# Ligand-dependent, palladium-catalyzed stereodivergent synthesis of chiral tetrahydroquinolines†

**DOI:** 10.1039/d2sc02771b

**Published:** 2022-06-20

**Authors:** Yue Wang, Er-Qing Li, Zheng Duan

**Affiliations:** College of Chemistry, Green Catalysis Center, International Phosphorus Laboratory, International Joint Research Laboratory for Functional Organophosphorus Materials of Henan Province, Zhengzhou University Zhengzhou 450001 P. R. China lierqing@zzu.edu.cn duanzheng@zzu.edu.cn

## Abstract

The most fundamental tasks in asymmetric synthesis are the development of fully stereodivergent strategies to access the full complement of stereoisomers of products bearing multiple stereocenters. Although great progress has been made in the past few decades, developing general and practical strategies that allow selective generation of any diastereomer of a reaction product bearing multiple stereocentres through switching distinct chiral catalysts is a significant challenge. Here, attaining precise switching of the product stereochemistry, we develop a novel *P*-chirogenic ligand, *i.e.*YuePhos, which can be easily derived from inexpensive and commercially available starting materials in four chemical operations. Through switching of three chiral ligands, an unprecedented ligand-dependent diastereodivergent Pd-catalyzed asymmetric intermolecular [4 + 2] cycloaddition reaction of vinyl benzoxazinanone with α-arylidene succinimides was developed. This novel method provides an efficient route for the stereodivergent synthesis of six stereoisomers of pyrrolidines bearing up to three adjacent stereocenters (one quaternary center). Despite the anticipated challenges associated with controlling stereoselectivity in such a complex system, the products are obtained in enantiomeric excesses ranging up to 98% ee. In addition, the synthetic utilities of optically active hexahydrocarbazoles are also shown.

## Abstract 2

An unprecedented ligand-dependent stereodivergent Pd-catalyzed asymmetric intermolecular [4 + 2] cycloaddition reaction of vinyl benzoxazinanone with α-aryliene succinimides was developed.

## Graphical abstract

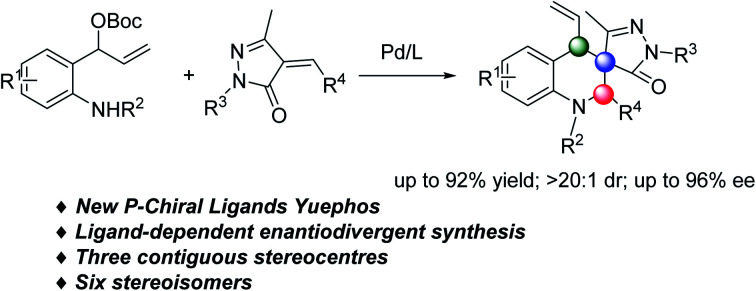

The chirality of a biologically active molecule can alter its physiological properties. Therefore, highly efficient access to and fully characterizing all possible stereoisomers of a chiral molecule is one of the fundamental challenges in organic synthesis, drug discovery and development processes. However, most asymmetric catalytic transformations afford products enantioselectively and diastereoselectively and only form one of the stereoisomers containing multiple stereocenters. Stereodivergent access to all possible stereoisomers of the products is incredibly difficult because diastereochemical preference is largely dominated by the inherent structural and stereoelectronic characteristics of substrates, while absolute conformation can be dictated by the choice of the chiral catalyst.^1^ In 2013, Carreira and co-workers addressed this limitation by introducing the concept of stereodivergent dual-catalytic synthesis, reporting the allylation of aldehydes in a diastereodivergent fashion by the synergistic reactivity of iridium and amine catalysts under acidic conditions.^2^ Soon after, Carreira,^3^ Zhang,^4^ Hartwig,^5^ Dong,^6^ Wang,^7^ Zi,^8^ Lee,^9^ and other groups^10^ reported using an appropriate combination of dual chiral catalysts in a series of elegant studies (Scheme 1A). Recently, chemists found, in some cases, that tuning non-chiral parameters, including solvents or additives, also controlled the stereochemical outcomes through subtle perturbation of the key diastereomeric transition states.^11^ In 2018, You and co-workers reported a solvent-controlled palladium-catalyzed enantioselective dearomative formal [3 + 2] cycloaddition, affording stereodivergent synthesis of two diastereomeric tetrahydrofuroindoles.^12^ However, a rapid and predictable way to access complete stereoisomers of products bearing multiple stereocentres (for example, three contiguous stereocentres) remains an unsolved challenge through switching of ligands. To the best of our knowledge, only two successful examples were reported by Buchwald and Zhang, in which eight stereoisomers were obtained through tuning catalysts and reactive substrates (Scheme 1B).^4*a*,13^

**Scheme 1:**
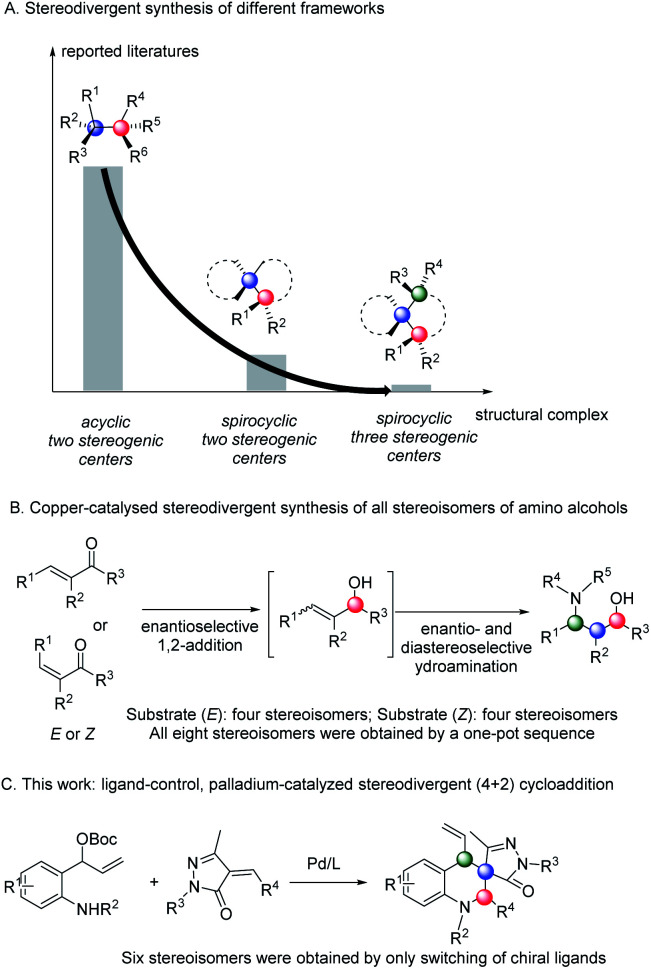
Strategy for stereodivergent synthesis of different stereoisomers.

In metal-catalyzed reactions, ligands can manipulate the reactivity and selectivity by affecting the steric and electronic properties of metal catalysts. Therefore, the design and development of new ligands to improve the utility, activity and selectivity of their related metal catalysts are greatly desired by organic chemists. Recently, our groups have synthesized a new and promising class of *P*-chiral ligands ZD-Phos (including Ganphos and Jiaphos), and their conformational rigidity and chemical robustness have endowed the structure and its variants with outstanding activity and selectivity as well as excellent stereocontrol features essential to asymmetric cycloaddition reactions.^14^ Inspired by these advances, we are interested in continuing the development of *P*-chiral ligands with new structural motifs in the search for new reactivity and selectivity to tackle current synthetic challenges. More recently, Sadphos has emerged as another superior chiral skeleton, owing to the pioneering contributions by Zhang.^15^ Thus its aminophosphine scaffold is envisaged to be introduced into our 1-phosphanorbornene framework (ZD-Phos).^16^ We aim to combine the advantages of the aforementioned two types of chiral motifs, thus developing a novel *P*,*P*-bidentate ligand. Thus the novel *P*-chiral ligands, called Yuephos, may show unique stereoselectivity in a metal-catalyzed asymmetric cycloaddition reaction (Fig. 1).

**Fig. 1:**
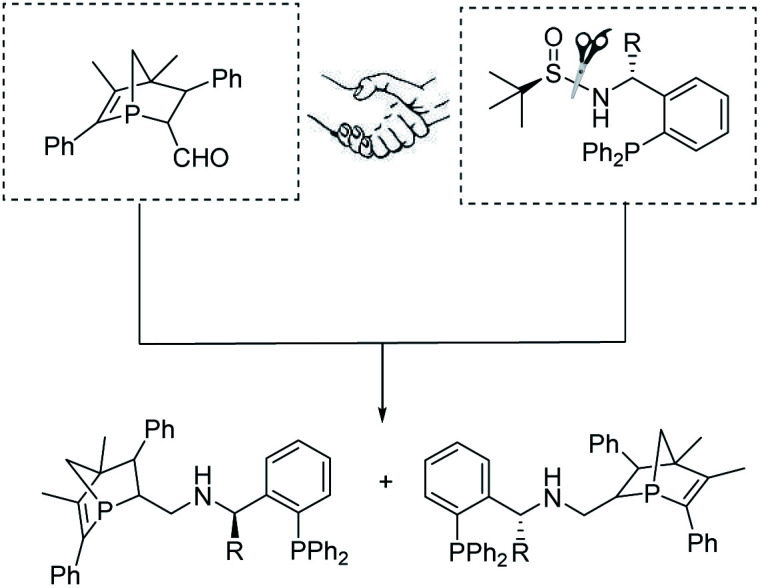
Design of the Yuephos framework.

Tetrahydroquinolines are important molecular skeletons that widely occur in natural molecules, pharmaceuticals, and functional materials. For this reason, realizing stereodivergent synthesis of all stereoisomers of fully substituted tetrahydroquinolines has been an important and challenging task in organic synthesis. However, to date, full control of absolute and relative stereochemical configuration of these molecules has remained an unmet synthetic challenge. Considering the potentiality of fully substituted chiral tetrahydroquinolines in drug discovery and stereodivergent synthesis,^17^ we envisioned that using our new palladium/ZD-Phos catalytic system may offer an efficient strategy for overcoming the challenges related to regio-, enantio-, and diastereo-selectivity. Herein, we report our studies on the unexplored stereodivergent synthesis of fully substituted tetrahydroquinolines through ligand-controlled, metal-catalyzed asymmetric annulation. Six possible stereoisomers bearing two tertiary and one quaternary stereocenters were easily synthesized in good yields with high enantio- and diastereo-selectivities from the same starting materials (Scheme 1C).

The new bisphosphorus ligands we report herein can be easily synthesized by a two-pot method with good yields (Scheme 2). Starting from the corresponding aldehyde^18^ and commercially available chiral amine, one-pot sequential reaction gave diastereomers Y1 and Y1′ with 1 : 1 dr, which could be straightforwardly separated by column chromatography. The subsequent reduction using Raney Ni produced the final Yuephos in good yields. The absolute configuration of Yue-1′ was established by single crystal X-ray diffraction.^19^ Importantly, the ligands Yuephos can remain stable in air and moisture for more than one year.

**Scheme 2:**
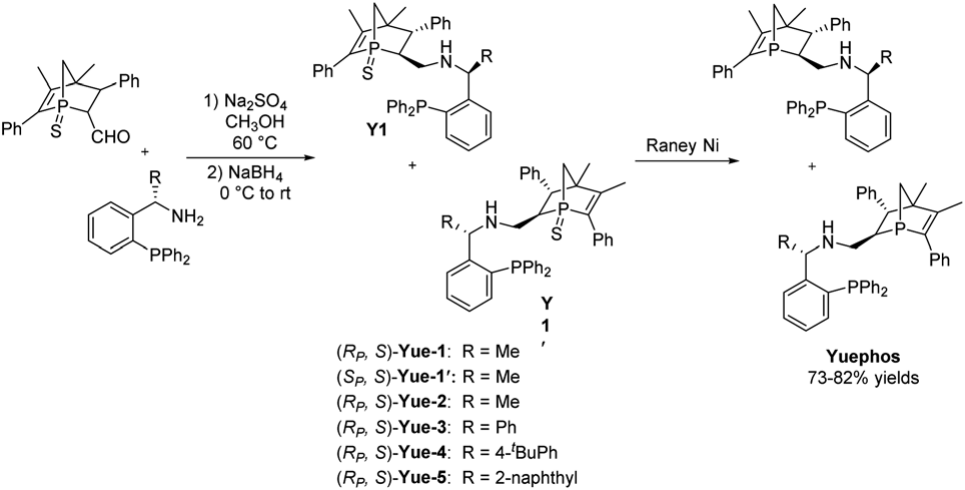
Synthesis of Yuephos ligands.

With new Yuephos ligands in hand, we began our study by choosing vinyl benzoxazinanone 1a with α-phenylidene succinimide 2a as the model substrate, combined with the Pd_2_dba_3_·CHCl_3_/L complex as the catalyst. Details of [Pd] source and solvent screening can be found in the ESI (Table S1 and S2†). Notably, using Pd_2_dba_3_·CHCl_3_/Yuephos as the catalyst in ethyl acetate, the reaction proceeded smoothly, affording the desired product 3a in 69% yield with 96% ee and >20 : 1 dr (entry 1). It should be noted that Yuephos ligands were found to be efficient for this reaction, and the product 3a was obtained in good enantioselectivity with seemingly irregular yields and diastereoselectivities (entries 2–6). Trost's ligand (L1) and chiral diphosphine ligand (L2) promoted the reaction with good diastereoselectivity but in a low yield and poor enantioselectivity (entries 7–8). However, (*R*)-SegPhos (L3) failed to afford the desired product (entry 9). To our surprise, when the phosphoramidite ligand (L4) was used, the diastereoselectivity was reversed compared to that in Yuephos (entry 10). Thus, a diastereodivergent phenomenon induced by the chiral ligand was discovered. To further improve the yield and selectivity, various solvents and [Pd] sources were screened (Table S3 and S4 in the ESI†), and an obvious improvement in the enantioselectivity and diastereoselectivity was observed when using DCM as the solvent (entries 10 *vs.* 11). The reaction enantioselectivity was further increased to 92% with good yield (85%) when the reaction temperature was reduced to −20 °C (entries 12–14).

With the optimal conditions established for (*S*, *R*, *S*)-3a (Table 1, entry 1) and (*S*, *S*, *S*)-4a (Table 1, entry 14), we turned our attention to exploring the scope with respect to vinyl benzoxazinanones by carrying out reactions with α-arylidene succinimides, and the results are summarized in Table 2. Using our new *P*-chiral ligand Yue-1 as a ligand, various groups on benzene rings of vinyl benzoxazinanones (including H, 6-OMe, 6-Me, 6-F, and 7-F) were first accommodated, leading to good yields with excellent diastereoselectivity and enantioselectivity (Table 2, 3a–3e). When more groups on vinyl benzoxazinanones and α-arylidene succinimides were simultaneously changed, the reaction could proceed smoothly, affording the corresponding products 3f and 3g in 53–74% yields with good enantioselectivity (Table 2, 3f and 3g). Attention was then paid to the groups on N1 of vinyl benzoxazinanones. To our delight, the N-CO_2_^i^Pr substituent was well tolerated, giving 3h in 77% yield with 91% ee, and acceptable diastereoselectivity (Table 2, 3h). The scope with respect to the α-arylidene succinimides turned out to be broad as well. Different substituted aryl groups (4-MeC_6_H_4_, 3-MeOC_6_H_4_, 2-MeC_6_H_4_, 4-FC_6_H_4_, 3-FC_6_H_4_, and 4-ClC_6_H_4_) were tolerated as shown in 3i–3n (Table 2, 3i–3n). α-Arylidene succinimide containing naphthalene was a suitable substrate giving the corresponding products 3o with well-controlled stereochemistry (>20 : 1 dr, 85% ee) (Table 2, 3o). The reaction could be extended to α-arylidene succinimides containing different N-protected groups (such as 4-ClC_6_H_4_, 4-MeC_6_H_4_, and 3,4-Me_2_C_6_H_3_), which provided the products with high levels of stereocontrol (Table 2, 3p–3r). The absolute configuration (*S*, *R*, *S*) of 3c was unambiguously assigned by single-crystal X-ray analysis.^20^

**Table 1:** Optimization of reaction conditions^a^

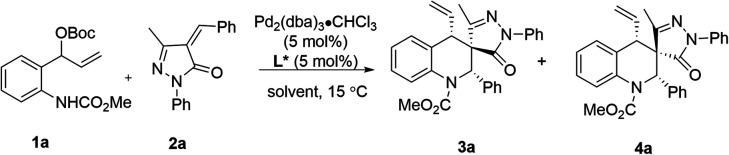					
Entry	Ligands	Solvent	Yield^b^ (%)	dr^c^ (3a : 4a)	ee^d^ (%)
1	Yue-1	EA	69	>20 : 1	96 (*S*, *R*, *S*)
2	Yue-1′	EA	64	4 : 1	33 (*S*, *R*, *S*)
3	Yue-2	EA	73	>20 : 1	95 (*R*, *S*, *R*)
4	Yue-3	EA	60	6 : 1	80 (*S*, *R*, *S*)
5	Yue-4	EA	44	3 : 1	85 (*S*, *R*, *S*)
6	Yue-5	EA	62	14 : 1	90 (*S*, *R*, *S*)
7	L1	EA	31	>20 : 1	14 (*S*, *R*, *S*)
8	L2	EA	42	>20 : 1	73 (*S*, *R*, *S*)
9	L3	EA	—	—	—
10^e^	L4	EA	64	1 : 15	77 (*S*, *S*, *S*)
11^e^	L4	DCM	89	<1 : 20	87 (*S*, *S*, *S*)
12^e^^,^^f^	L4	DCM	89	<1 : 20	86 (*S*, *S*, *S*)
13^e^^,^^g^	L4	DCM	87	<1 : 20	88 (*S*, *S*, *S*)
14^e^^,^^h^	L4	DCM	85	<1 : 20	92 (*S*, *S*, *S*)
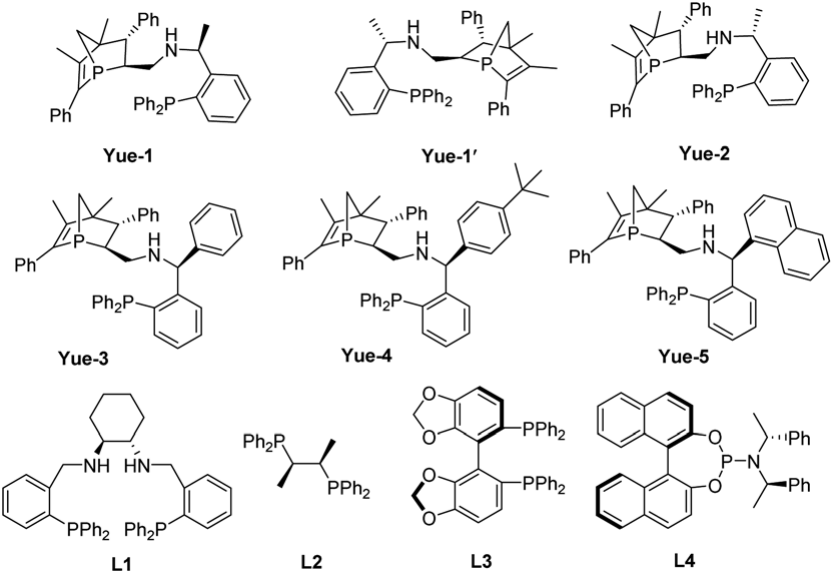					

a Unless otherwise stated, reactions were performed with 1a (60 mg, 0.2 mmol) and 2a (26 mg, 0.1 mmol), in 1.0 mL of solvent at 15 °C for 72 h, and EA = ethyl acetate; DCM = dichloromethane.

b Isolated yield after chromatography.

c The diastereomeric ratios were determined by column chromatography.

d Determined by HPLC analysis.

e L4 (10 mol%) was used, Cs_2_CO_3_ (2.0 equiv.).

f Reaction temperature: 0 °C.

g Reaction temperature: −10 °C.

h Reaction temperature: −20 °C.

**Table 2:** Scope of the substrates for the synthesis of (*S*, *R*, *S*)-3^a^

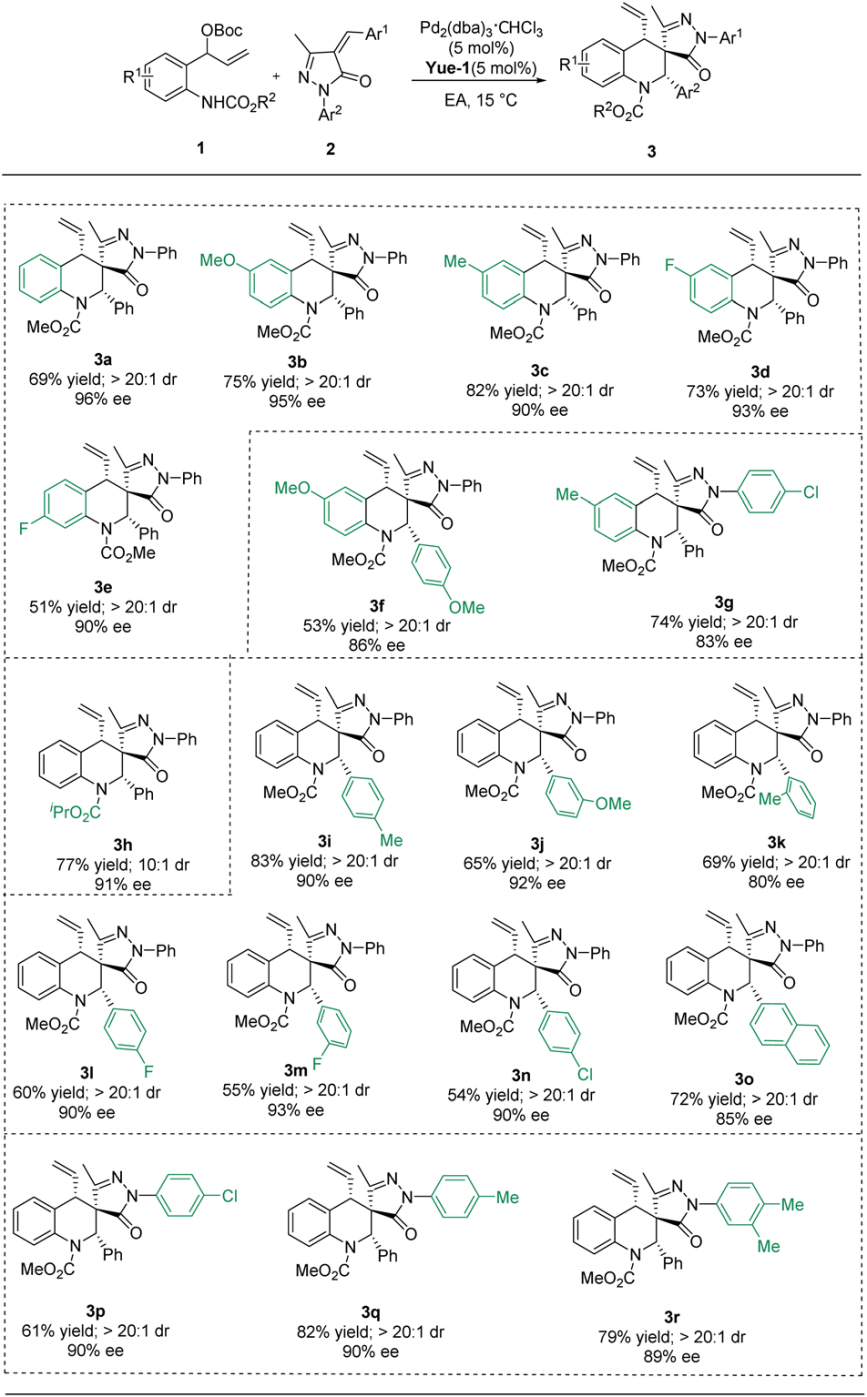

a Reaction conditions: see Table 1, entry 1. The yield is isolated yield. The reaction was performed for 72 h.

Compared with the Pd/Yue-1 complex, the reaction, using the Pd/L4 complex as the catalyst, proceeded smoothly to produce the diastereoisomer 4. As outlined in Table 3, most of these [4 + 2] cycloaddition reactions proceeded well, giving the corresponding (*S*, *S*, *S*) products in 42−92% yields with dr values ranging from 9 : 1 to >20 : 1 and ee values of up to 98%. Of particular note, (*S*, *S*, *S*)-4 and (*S*, *R*, *S*)-3 could be accessed with comparably high selectivities when using substrates containing H, 6-Me and 6-Br groups on benzene rings (Table 3, 4a–4e). Furthermore, the groups on N1 of vinyl benzoxazinanones have no apparent influence on the yield and stereoselectivity (Table 3, 4a*vs.*4f). Substrates 2 bearing either an electron-donating group (4-Me, 4-MeO, 3-MeO, 2-MeO, 4-EtO, and 3,4-OMe_2_) or electron-withdrawing group (4-NO_2_, 4-CN, 4-CF_3_, 4-F, 4-Cl, and 3-Cl) on aryl rings provided 4 with good to excellent yields (56–87%), good diastereoselectivity (10 : 1 to >20 : 1 dr), and good to excellent enantioselectivity (84–97% ee) (Table 3, 4g–4r). In addition, naphthyl and thienyl-substituted α-arylidene succinimides 2s and 2t reacted with 1a to give 4s and 4t in 66–88% yield with 9 : 1 to >20 : 1 dr and up to 92% ee (Table 3, 4s and 4t). Moreover, the Ar2 groups of α-arylidene succinimides were changed to 4-MeC_6_H_4_ and 4-ClC_6_H_4_, and the reaction still proceeded smoothly, affording the desired diastereomers (*S*, *S*, *S*) 4u and 4v with satisfactory yields and stereoselectivities (Table 3, 4u and 4v). Finally, the absolute configuration of 4a was determined to be (*S*, *S*, *S*) by X-ray crystallographic analysis of a single crystal of the enantiopure sample.^21^

**Table 3:** Scope of the substrates for the synthesis of (*S*, *S*, *S*)-4^aa^

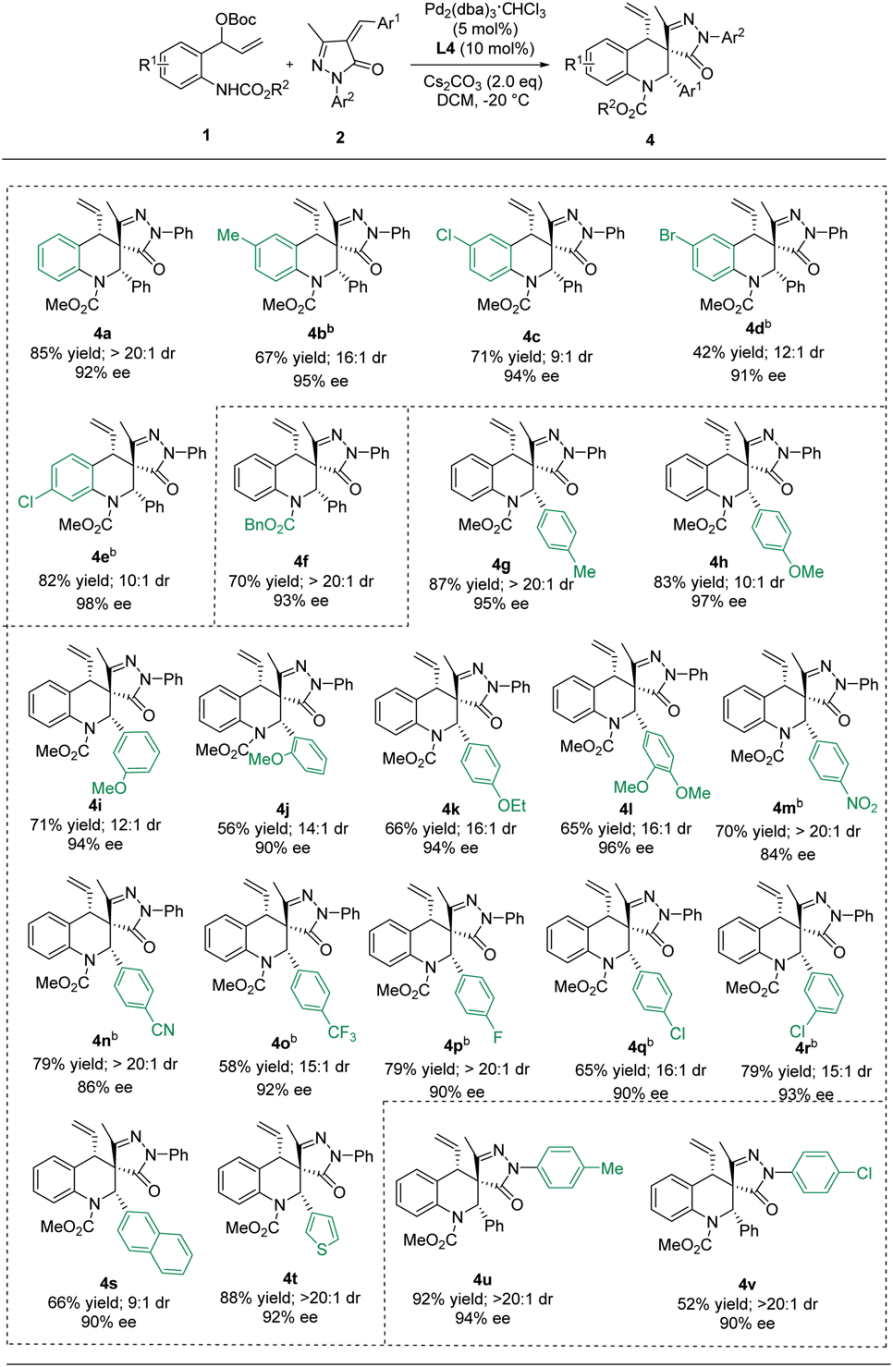

a Reaction conditions: see Table 1, entry 14. The yield is isolated yield. The reaction was performed for 72 h.

b Reaction temperature: −30 °C.

Subsequently, we wondered if these reaction conditions could be adapted to the stereodivergent construction of more diastereoisomers. By switching the absolute configuration of different ligands, four of the possible stereoisomers were successfully obtained. Encouraged by this result, some commercial ligands were first examined with optimal conditions; unfortunately, only diastereoisomers (*S*, *R*, *S*)-3a and (*S*, *S*, *S*)-4a were produced in all cases. Recently, we developed a new *P*-chiral ligand Mengphos, which has verified good performance in palladium catalysis. To our delighted, a new diastereoisomer (*R*, *R*, *S*)-5 was obtained in moderate enantioselectivity in the presence of Pd/Meng-1. Details of ligand and solvent screening can be found in the ESI (Table S5†). When the Pd/Meng-2 complex was used as the catalyst, its enantiomer (*S*, *S*, *R*)-5 was produced with moderate stereoselectivity (Scheme 3). Fortunately, six stereoisomers could be easily obtained after column chromatography, as confirmed by the high-performance liquid-chromatography traces. To the best of our knowledge, this is the first example of stereodivergent construction of six chiral tetrahydroquinolines containing three contiguous stereocenters by only switching chiral ligands (Scheme 3).

**Scheme 3:**
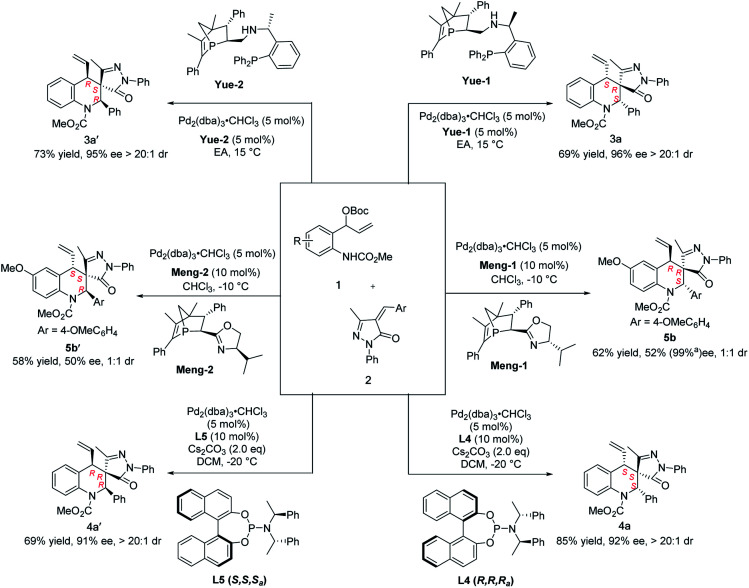
Synthesis of six stereoisomers by switching the chiral ligands. ^a^After recrystallization, the mother liquor was tested to get the relevant data.

To demonstrate the practicality of the reaction, a scale-up experiment was performed (Scheme 4). To our delight, the products (*S*, *R*, *S*)-3a and (*S*, *S*, *S*)-4a were obtained in 94% ee and 92% ee, respectively. Then, different transformations with regard to tetrahydroquinolines (*S*, *R*, *S*)-3a were conducted. At first, the hydrogenation of (*S*, *R*, *S*)-3a was conducted in the presence of Pd/C, furnishing the desired product 6 in 96% yield. In addition, the product (*S*, *R*, *S*)-3a could undergo selective hydroboration to give the *anti*-Markovnikov product 7 in 83% yield.

**Scheme 4:**
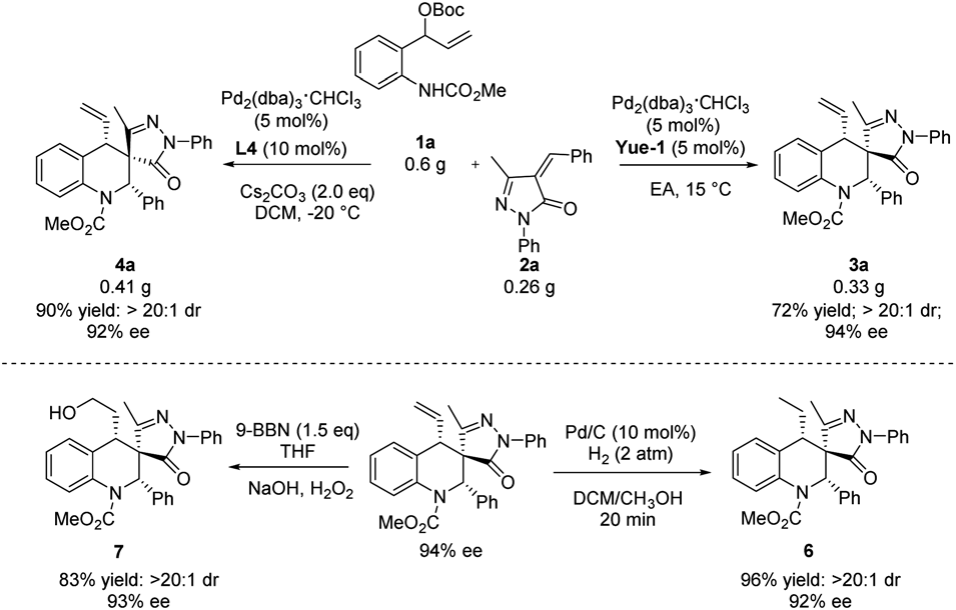
Scale-up experiment transformations of the multifunctional products.

## Conclusions

In summary, we developed new *P*-chiral ligands Yuephos, which showed good performance for highly enantioenriched synthesis of tetrahydroquinolines. This protocol assembled three contiguous stereocentres (one quaternary centre), using vinyl benzoxazinanones and α-arylidene succinimide substrates, in a highly selective, palladium-catalysed [4 + 2] cycloaddition reaction. Essential to our approach was highly effective ligand control, and by switching appropriate ligands, six diastereomers could be obtained with high diastereo- and enantio-selectivity in most cases. This method represents a rare example of the stereodivergent synthesis of spiro compounds from the same set of starting materials. We believed that this work might be generally applicable to the development of other enantio- and diastereo-divergent cycloaddition reactions.

## Data availability

Full experimental and characterization data is available in the ESI.†

## Author contributions

Z. D. and E.-Q. L. directed the project. Y. W. performed the experiments and analyzed the data.

## Conflicts of interest

There are no conflicts to declare.

## Supplementary Material

SC-013-D2SC02771B-s001

SC-013-D2SC02771B-s002
