# Supplementary material for: Ligand-dependent, palladium-catalyzed stereodivergent synthesis of chiral tetrahydroquinolines
Source: Chem Sci. 2022 Jun 20;13(27):8131–6. doi: 10.1039/d2sc02771b (PMC9278114; doi:10.1039/d2sc02771b)
Supplement: SC-013-D2SC02771B-s001 [file SC-013-D2SC02771B-s001.pdf]

Electronic Supplementary Material (ESI) for Chemical Science.
This journal is © The Royal Society of Chemistry 2022

Supporting Information

Ligand-Dependent, Palladium-Catalyzed Stereodivergent Synthesis of Chiral Tetrahydroquinolines

Yue Wang,[a] Er-Qing Li,[a*] and Zheng Duan[a*]
College of Chemistry, Green Catalysis Center, International Phosphorus Laboratory, International Joint Research Laboratory for Functional Organophosphorus Materials of Henan Province, Zhengzhou University, Zhengzhou 450001, P. R. China General Experimental Details.

1. General experimental details 2
2. General Procedure for Synthesis of Chiral Ligands Yue1-5 2
3. Optimization of the Reaction Conditions 7
   3.1 Optimization of reaction conditions with Yuephos ligands. 7
   3.2 Optimization of reaction conditions with P-N ligands. 8
   3.3 Optimization of reaction conditions with Mengphos ligands. 8
4. General Procedure for Reactions 9
5. Copies of ^{1}H NMR, ^{13}C NMR, ^{31}P NMR Spectra 36
6. Copies of HPLC Chromatograms 89
7. X-ray crystal structure 136

1. General experimental details

All reactions were performed under nitrogen using solvents dried by standard methods. NMR spectra were obtained using Bruker AV300 spectrometer. Chemical shifts are expressed in parts per million (ppm) downfield from internal TMS. HRMS spectra were obtained on an Agilent 1290-6540 UHPLC Q-Tof HR-MS spectrometer. X-ray crystallographic analyses were performed on an Oxford diffraction Gemini E diffractometer. Melting Point: heating rate: 4°C/min, the thermometer was not corrected. Enantiomer excesses were determined by chiral HPLC analysis on Chiralcel AD-H in comparison with the authentic racemates. Chiral HPLC analysis recorded on Shanghaiyice instruments and Equipment Co. Ltd. and Shimadzu LC-20A. Silica gel (200-300 mesh) was used for the chromatographic separations. All commercially available reagents were used without further purification. The compounds **1**[1] and **2**[2] were prepared according to literature methods.

2. General Procedure for Synthesis of Chiral Ligands Yue1-5

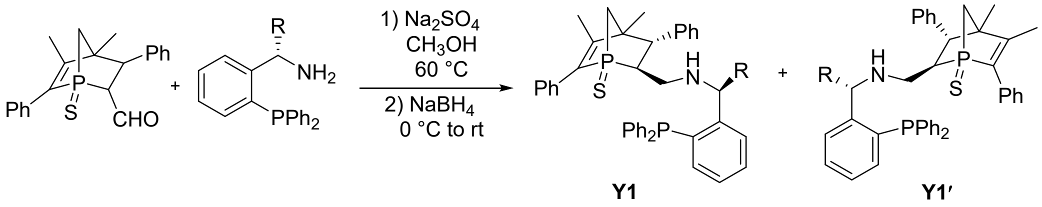

The corresponding aldehyde[3] and chiral imines[4] were prepared according to the literature methods: to a flask containing a solution of aldehyde (700 mg, 2.0 mmol) and corresponding chiral imines (3.0 mmol), then Na_2SO_4 (1 g) was added and the mixture was stirred at 60 °C. The progress of the reaction was followed by ^{31}P NMR. Upon reaction completion, the reaction mixture was cooled to 0 ˚C and $NaBH_4$ (227 mg, 6.0 mmol, 3 equiv.) was added. The reaction mixture was stirred at 0 ˚C for 30 min and then for 2 h at room temperature. When the reaction was completed the reaction mixture was quenched with H_2O and extracted with CH_2Cl_2, the combined

organic phases were dried over MgSO4 and the solvent was removed in vacuo. The residue was purified by silica gel chromatography (ethyl acetate /petroleum ether = 1:2) to afford the products **Y1** and **Y1'**.

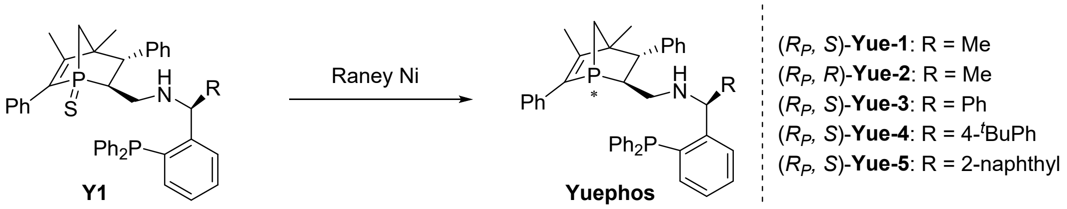

To a flame dried sealing tube, Raney nickel (20 equiv.) was added and washed 3 times with EtOH, 3 times with THF. A solution of **Y1** (1 mmol) in THF was added and the mixture was stirred at room temperature for 2 h. The progress of the reaction was followed by ^{31}P NMR. Upon reaction completion, the resulting suspension was filtered and obtained the desired **Yue-phoses**.

(*S*)-N-(((1*R*,2*R*,3*S*,4*S*)-4,5-dimethyl-3,6-diphenyl-1-phosphabicyclo[2.2.1]hept-5-en-2-yl)methyl)-1-(2-(diphenylphosphanyl)phenyl)ethan-1-amine (Yue-1).

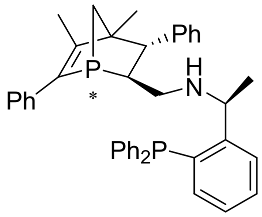

Purified by silica gel chromatography using PE/EA = 4:1, white solid (475 mg, 78% yield). [α]$_D$ = +142 (*c* = 0.1, CH_2Cl_2, 15.9 °C). MP: 72.2 – 74.3 °C. ^{31}P NMR (121 MHz，$CDCl_3$) δ -17.21，-17.55 ppm; ^{1}H NMR (300 MHz, $CDCl_3$) δ 7.55 – 7.51 (m, 1H), 7.41 (d, *J* = 7.7 Hz, 2H), 7.34 – 7.26 (m, 8H), 7.24 – 7.14 (m, 9H), 7.08 – 7.01 (m, 3H), 6.81 (ddd, *J* = 7.8, 4.3, 1.3 Hz, 1H), 4.53 – 4.46 (m, 1H), 2.65 – 2.42 (m, 3H), 2.37 (d, *J* = 5.4 Hz, 1H), 2.06 (s, 1H), 1.60 (dd, *J* = 11.5, 7.1 Hz, 1H), 1.43 – 1.36 (m, 4H), 1.22 (s, 3H), 0.91 (d, *J* = 6.4 Hz, 3H) ppm; ^{13}C NMR (75 MHz, $CDCl_3$) δ 152.2 (d, *J* = 1.1 Hz), 150.0 (d, *J* = 22.1 Hz), 141.8, 141.3 (d, *J* = 15.7 Hz), 138.9 (d, *J* = 20.7 Hz), 137.1 (d, *J* = 10.6 Hz), 136.7 (d, *J* = 10.2 Hz), 135.2 (d, *J* = 12.8 Hz), 134.3, 134.0, 133.1, 129.5, 128.8, 128.7, 128.7, 128.6, 128.6, 128.5, 128.5, 128.4, 128.3, 127.8, 126.9, 126.4, 126.4, 126.3, 126.3, 64.8 (d, *J* = 4.8 Hz), 57.5 (d, *J* = 2.3 Hz), 54.2 (d, *J* = 25.2 Hz), 52.4 (d, *J* = 17.5 Hz), 48.3 (d, *J* = 4.1 Hz), 46.9 (dd, *J* = 14.6, 2.3 Hz), 23.7, 20.7, 16.3 ppm. HRMS (ESI) calcd for $C_{41}H_{42}NP_2^+$ [M+H]$^+$: 610.2787, found 610.2794.

(*S*)-N-(((1*R*,2*R*,3*S*,4*R*)-4,5-dimethyl-3,6-diphenyl-1-phosphabicyclo[2.2.1]hept-5-en-2-yl)methyl)-1-(2-(diphenylphosphanyl)phenyl)ethan-1-amine (Yue-1').

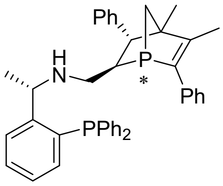

Purified by silica gel chromatography using PE/EA = 4:1, white solid (500 mg, 82% yield). $[\alpha]_D$ = -138 (*c* = 0.1, CH_2Cl_2, 15.1 °C). MP: 75.5 – 77.2 °C. ^{31}P NMR (121 MHz，$CDCl_3$) δ -17.42，-18.89 ppm; ^{1}H NMR (300 MHz, $CDCl_3$) δ 7.57 (dd, *J* = 7.8, 4.3 Hz, 1H), 7.39 – 7.35 (m, 5H), 7.31 – 7.15 (m, 14H), 7.10 (t, *J* = 7.6 Hz, 1H), 6.99 – 6.96 (m, 2H), 6.84 (dd, *J* = 7.7, 4.3 Hz, 1H), 4.61 (p, *J* = 6.7 Hz, 1H), 2.64 (ddd, *J* = 13.9, 11.4, 5.7 Hz, 1H), 2.42 – 2.32 (m, 2H), 2.29 – 2.24 (m, 1H), 1.72 (s, 1H), 1.61 – 1.53 (m, 1H), 1.42 (s, 3H), 1.41 – 1.34 (m, 1H), 1.20 (s, 3H), 1.12 (d, *J* = 6.4 Hz, 3H) ppm; ^{13}C NMR (75 MHz, $CDCl_3$) δ 152.3 (d, *J* = 1.0 Hz), 150.2 (d, *J* = 23.9 Hz), 141.1 (d, *J* = 15.4 Hz), 138.9 (d, *J* = 20.8 Hz), 137.1 (d, *J* = 10.4 Hz), 136.7 (d, *J* = 10.6 Hz), 135.2 (d, *J* = 13.0 Hz), 134.3, 134.2, 134.0, 133.9, 133.3, 129.5, 128.9, 128.7, 128.7, 128.6, 128.5, 128.5, 128.5, 128.3, 127.7, 126.9, 126.4, 126.3, 126.0 (d, *J* = 4.9 Hz), 64.5 (d, *J* = 4.8 Hz), 57.1 (d, *J* = 2.7 Hz), 55.1 (d, *J* = 25.2 Hz), 51.8 (d, *J* = 14.2 Hz), 48.3 (d, *J* = 4.0 Hz), 48.0 (d, *J* = 14.8 Hz), 24.0, 20.7, 16.3 ppm. HRMS (ESI) calcd for $C_{41}H_{42}NP_2^+$ $[M+H]^+$: 610.2787, found 610.2791.

(*R*)-N-(((1*R*,2*R*,3*S*,4*S*)-4,5-dimethyl-3,6-diphenyl-1-phosphabicyclo[2.2.1]hept-5-en-2-yl)methyl)-1-(2-(diphenylphosphanyl)phenyl)ethan-1-amine (Yue-2).

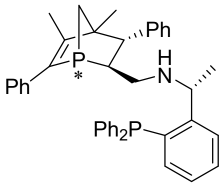

Purified by silica gel chromatography using PE/EA = 4:1, white solid (462 mg, 76% yield). $[\alpha]_D$ = -79 (*c* = 0.1, CH_2Cl_2, 16.2 °C). MP: 66.1 – 67.9 °C. ^{31}P NMR (121 MHz，$CDCl_3$) δ -17.16，-17.51 ppm; ^{1}H NMR (300 MHz, $CDCl_3$) δ 7.53 (dd, *J* = 7.9, 4.3 Hz, 1H), 7.41 (d, *J* = 7.7 Hz, 2H), 7.35 – 7.16 (m, 17H), 7.09 – 7.01 (m, 3H), 6.81 (dd, *J* = 7.8, 4.3 Hz, 1H), 4.51 (p, *J* = 6.6 Hz, 1H), 2.65 – 2.41 (m, 3H),, 2.37 (d, *J* = 5.5 Hz, 1H), 1.82 (s, 1H), 1.60 (dd, *J* = 11.5, 7.1 Hz, 1H), 1.43 – 1.36 (m, 4H), 1.22 (s, 3H), 0.92 (d, *J* = 6.4 Hz, 3H) ppm; ^{13}C NMR (75 MHz, $CDCl_3$) δ 152.2 (d, *J* = 1.1 Hz), 150.0 (d, *J* = 21.9 Hz), 141.2 (d, *J* = 15.4 Hz), 138.9 (d, *J* = 20.9 Hz), 137.1 (d, *J* = 10.7 Hz), 136.7 (d, *J* = 10.3 Hz), 135.2 (d, *J* = 12.9 Hz),134.3, 134.3, 134.0,

134.0, 133.1, 129.5, 128.8, 128.7, 128.7, 128.6, 128.5, 128.5, 128.5, 128.4, 128.2, 127.8, 126.8, 126.4, 126.4, 126.3, 64.8 (d, *J* = 4.8 Hz), 57.4 (d, *J* = 2.7 Hz), 54.2 (d, *J* = 25.3 Hz), 52.4 (d, *J* = 17.4 Hz), 48.3 (d, *J* = 3.9 Hz), 46.9 (dd, *J* = 14.8, 2.4 Hz), 23.7, 20.7, 16.2 ppm. HRMS (ESI) calcd for $C_{41}H_{42}NP_2^+$ $[M+H]^+$: 610.2787, found 610.2792.

(*S*)-N-(((1*R*,2*R*,3*S*,4*S*)-4,5-dimethyl-3,6-diphenyl-1-phosphabicyclo[2.2.1]hept-5-en-2-yl)methyl)-1-(2-(diphenylphosphanyl)phenyl)-1-phenylmethanamine (Yue-3).

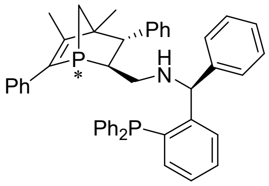

Purified by silica gel chromatography using PE/EA = 4:1, white solid (496 mg, 74% yield). $[\alpha]_D$ = +85 (*c* = 0.1, CH_2Cl_2, 16.5 °C). MP: 76.6 – 78.5 °C. ^{31}P NMR (121 MHz，$CDCl_3$) δ -16.45，-17.43 ppm; ^{1}H NMR (300 MHz, $CDCl_3$) δ 7.48 (ddd, *J* = 7.9, 4.3, 1.3 Hz, 1H), 7.39 – 7.35 (m, 4H), 7.25 – 7.15 (m, 15H), 7.13 – 7.04 (m, 6H), 6.98 (dd, *J* = 7.3, 2.1 Hz, 2H), 6.85 (ddd, *J* = 7.8, 4.2, 1.3 Hz, 1H), 5.64 (d, *J* = 7.9 Hz, 1H), 2.73 – 2.54 (m, 2H), 2.38 (d, *J* = 5.7 Hz, 1H), 2.26 – 2.19 (m, 1H), 1.72 (s, 1H), 1.59 (ddd, *J* = 11.6, 7.2, 1.6 Hz, 1H), 1.45 – 1.36 (m, 4H),1.19 (s, 3H) ppm; ^{13}C NMR (75 MHz, $CDCl_3$) δ 152.3 (d, *J* = 1.1 Hz), 148.3 (d, *J* = 22.9 Hz), 141.2 (d, *J* = 15.9 Hz), 138.9 (d, *J* = 20.7 Hz), 137.3 (d, *J* = 10.1 Hz), 136.7 (d, *J* = 10.5 Hz), 135.7 (d, *J* = 13.3 Hz), 134.1 (d, *J* = 1.6 Hz), 133.9 (d, *J* = 1.9 Hz), 133.8, 129.4, 128.9, 128.8, 128.7, 128.6, 128.5, 128.4, 128.3, 128.3, 128.2, 128.1, 127.8, 127.1, 126.6, 126.5, 126.4, 64.7 (d, *J* = 4.7 Hz), 63.4 (d, *J* = 23.2 Hz), 57.2 (d, *J* = 2.5 Hz), 52.5 (d, *J* = 15.6 Hz), 48.4 (d, *J* = 3.8 Hz), 47.3 (d, *J* = 15.0 Hz), 20.8, 16.3 ppm. HRMS (ESI) calcd for $C_{46}H_{44}NP_2^+$ $[M+H]^+$: 672.2943, found 672.2939.

(*S*)-1-(4-(tert-butyl)phenyl)-N-(((1*R*,2*R*,3*S*,4*S*)-4,5-dimethyl-3,6-diphenyl-1-phosphabicyclo[2.2.1]hept-5-en-2-yl)methyl)-1-(2-(diphenylphosphanyl)phenyl)methanamine (Yue-4).

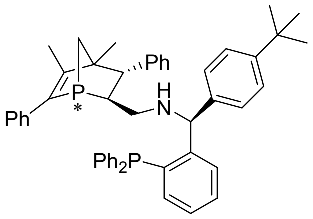

Purified by silica gel chromatography using PE/EA = 4:1, colourless oil (530 mg, 73% yield). $[\alpha]_D$ = +113 (c = 0.1, CH_2Cl_2, 17 °C). ^{31}P NMR (121 MHz，$CDCl_3$) δ -16.75，-17.36 ppm; ^{1}H NMR (300 MHz, $CDCl_3$) δ 7.59 – 7.54 (m, 1H), 7.40 – 7.35 (m, 4H), 7.24 – 7.17 (m, 13H), 7.09 – 6.97 (m, 9H), 6.84 (ddd, J = 7.8, 4.2, 1.4 Hz, 1H), 5.63 (d, J = 7.9 Hz, 1H), 2.75 – 2.57 (m, 2H), 2.41 (d, J = 5.8 Hz, 1H), 2.29 – 2.24 (m, 1H), 1.82 (s, 1H), 1.60 (ddd, J = 11.7, 7.1, 1.6 Hz, 1H), 1.47 – 1.43 (m, 1H), 1.41 (s, 3H), 1.22 (s, 9H), 1.20 (s, 3H) ppm; ^{13}C NMR (75 MHz, $CDCl_3$) δ 152.3 (d, J = 1.2 Hz), 149.1, 141.8, 141.2 (d, J = 15.6 Hz), 139.96 (d, J = 1.4 Hz), 138.89 (d, J = 21.0 Hz), 137.4 (d, J = 10.2 Hz), 136.6 (d, J = 10.4 Hz), 135.6 (d, J = 13.3 Hz), 134.1, 134.1, 133.8, 133.8, 133.8, 129.3, 128.9, 128.7, 128.7, 128.6, 128.5, 128.4, 128.4, 128.3, 128.3, 128.2, 127.9 (d, J = 5.1 Hz), 127.7, 127.4, 126.9, 126.4, 126.3, 126.3, 124.9, 64.7 (d, J = 4.8 Hz), 63.1 (d, J = 23.6 Hz), 57.2 (d, J = 2.8 Hz), 52.5 (d, J = 15.9 Hz), 48.4 (d, J = 4.0 Hz), 47.2 (d, J = 14.9 Hz), 34.3, 31.4, 20.7, 16.3 ppm.HRMS (ESI) calcd for $C_{50}H_{52}NP_2^+$ $[M+H]^+$: 728.3570, found 728.3577.

(*S*)-N-(((1*R*,2*R*,3*S*,4*S*)-4,5-dimethyl-3,6-diphenyl-1-phosphabicyclo[2.2.1]hept-5-en-2-yl)methyl)-1-(2-(diphenylphosphanyl)phenyl)-1-(naphthalen-1-yl)methanamine (Yue-5).

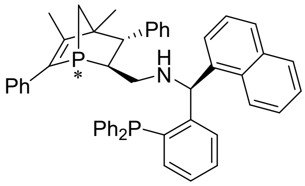

Purified by silica gel chromatography using PE/EA = 4:1, white solid (555 mg, 77% yield). $[\alpha]_D$ = +43 (c = 0.1, CH_2Cl_2, 16.8 °C). MP: 90.1 – 92 °C. ^{31}P NMR (121 MHz，$CDCl_3$) δ -17.13，-17.76 ppm; ^{1}H NMR (300 MHz, $CDCl_3$) δ 7.99 (d, J = 8.5 Hz, 1H), 7.90 (d, J = 7.2 Hz, 1H), 7.76 (d, J = 8.1 Hz, 1H), 7.68 (d, J = 8.2 Hz, 1H), 7.45 (t, J = 7.7 Hz, 1H), 7.38 – 7.30 (m, 5H), 7.28 – 6.99 (m, 19H), 6.92 (d, J = 6.8 Hz, 2H), 6.37 (d, J = 8.2 Hz, 1H), 2.84 (ddd, J = 15.0, 11.5, 5.7 Hz, 1H), 2.65 (q, J = 9.9 Hz, 1H), 2.32 (d, J = 5.6 Hz, 1H), 1.99 – 1.92 (m, 1H), 1.73 – 1.52 (m, 2H), 1.41 – 1.34 (m, 4H), 1.15 (s, 3H) ppm; ^{13}C NMR (75 MHz, $CDCl_3$) δ 152.4, 147.9 (d, J = 23.7 Hz), 141.7, 141.2 (d, J = 16.3 Hz), 138.9 (d, J = 20.8 Hz), 138.3, 136.9 (d, J = 9.3 Hz), 136.8 (d, J = 10.3 Hz), 136.1 (d, J = 13.1 Hz), 134.2, 134.1, 134.1, 134.0, 133.9, 133.8, 131.9, 129.3, 129.0, 128.9, 128.8, 128.7, 128.6, 128.5, 128.5, 128.4, 128.3, 127.7, 127.6, 127.3, 126.5, 126.4, 125.9, 125.5, 125.4 (d, J = 3.1 Hz), 125.3, 124.3 (d, J = 5.6 Hz), 64.6 (d, J = 4.7 Hz), 60.9 (d, J = 23.4 Hz), 57.2 (d, J = 2.5 Hz),

53.2 (d, J = 12.7 Hz), 48.32 (d, J = 4.7 Hz), 47.85 (d, J = 15.6 Hz), 20.7, 16.4 ppm. HRMS (ESI) calcd for $C_{50}H_{46}NP_2^+$ [M+H]$^+$: 722.3100, found 722.3109.

3. Optimization of the Reaction Conditions

3.1 Optimization of reaction conditions with Yuephos ligands.

Table S1. Screening of the solvents. [a]

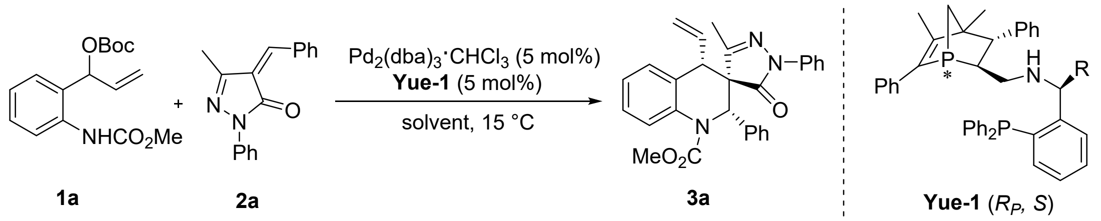

entry	Solvent	dr[b]	yield (%)[c]	ee (%)[d]
1	DCM	> 20:1	62	93
2	EA	> 20:1	69	96
3	THF	> 20:1	56	90
4	Toluene	> 20:1	56	86
5	MTBE	> 20:1	76	88

[a] Unless otherwise stated, reactions were performed with **1a** (60 mg, 0.2 mmol) and **2a** (26 mg, 0.1 mmol), in 1.0 mL of solvent at 15 °C for 72 h, MTBE = methyl tert-butyl ether. [b] The diastereomeric ratios were determined by column chromatography. [c] Isolated yield after chromatography. [d] Determined by HPLC analysis.

Table S2. Screening of the Pd catalysts. [a]

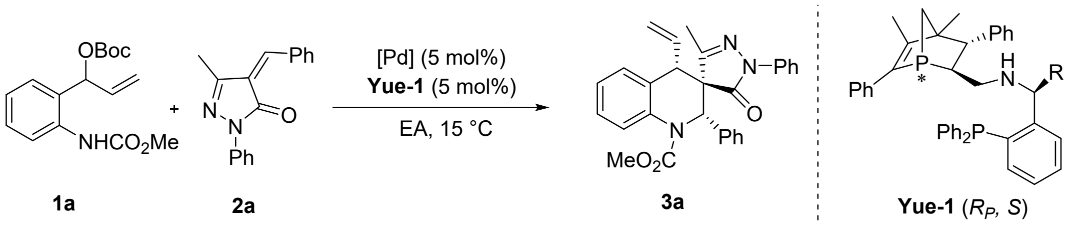

entry	Metal	dr[b]	yield (%)[c]	ee (%)[d]
1	$Pd_2(dba)_3$•$CHCl_3$	> 20:1	69	96
2	$[Pd(C_3H_5)Cl]_2$	-	-	-
3	$Pd(OAc)_2$	-	-	-

[a] Unless otherwise stated, reactions were performed with **1a** (60 mg, 0.2 mmol) and **2a** (26 mg, 0.1 mmol), in 1.0 mL of solvent at 15 °C for 72 h, MTBE = methyl tert-butyl ether. [b] The diastereomeric ratios were determined by column chromatography. [c] Isolated yield after chromatography. [d] Determined by HPLC analysis.

3.2 Optimization of reaction conditions with P-N ligands.

Table S3. Screening of the solvents. [a]

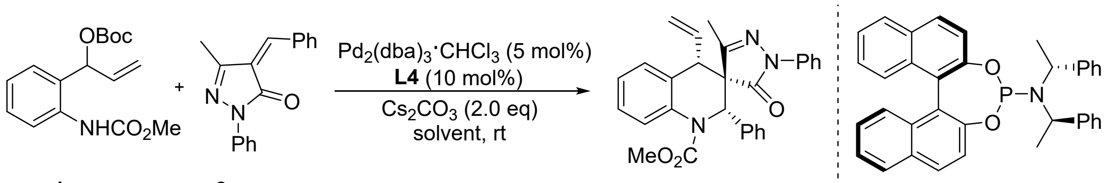

entry	solvent	dr[b]	yield (%)[c]	ee (%)[d]
1	DCM	> 20:1	89	87
2	EA	15:1	64	77
3	Toluene	10:1	44	75
4	THF	> 20:1	44	80

[a] Unless otherwise stated, reactions were performed with **1a** (60 mg, 0.2 mmol) and **2a** (26 mg, 0.1 mmol), in 1.0 mL of solvent at 25 °C for 6 h. [b] The diastereomeric ratios were determined by column chromatography. [c] Isolated yield after chromatography. [d] Determined by HPLC analysis.

Table S4. Screening of the Pd catalysts. [a]

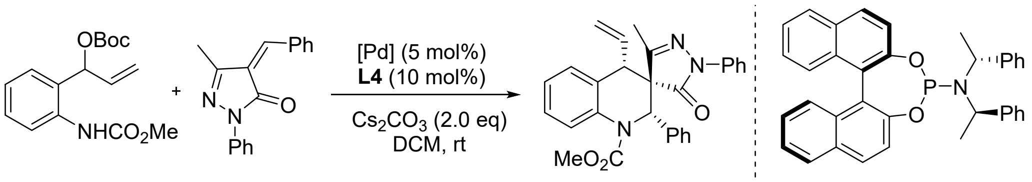

entry	metal	dr[b]	yield (%)[c]	ee (%)[d]
1	$Pd_2(dba)_3 \cdot CHCl_3$	> 20:1	89	87
2	$Pd_2(dba)_3$	> 20:1	80	87
3	$[Pd(C_3H_5)Cl]_2$	> 20:1	42	33
4	$Pd(OAc)_2$	> 20:1	44	89
5	$Pd(dba)_2$	> 20:1	64	88

[a] Unless otherwise stated, reactions were performed with **1a** (60 mg, 0.2 mmol) and **2a** (26 mg, 0.1 mmol), in 1.0 mL of solvent at 25 °C for 6 h. [b] The diastereomeric ratios were determined by column chromatography. [c] Isolated yield after chromatography. [d] Determined by HPLC analysis.

3.3 Optimization of reaction conditions with Mengphos ligands.

Table S5. Screening of the ligands, solvents and temperature. [a]

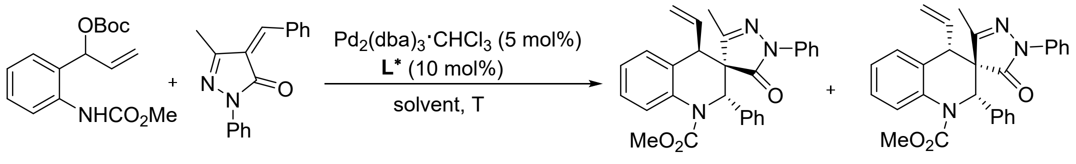

1a **2a** **5a** **4a**

entry	solvent	ligand	T (°C)	yield (%)[b]	dr (**5a**:**4a**)[c]	ee (%)[d]
1	DCM	**Meng-1**	rt	60	1:2	11
2	DCE	**Meng-1**	rt	51	1:1	7
3	$CHCl_3$	**Meng-1**	rt	80	1:1	30
4	EA	**Meng-1**	rt	trace	-	-
5	THF	**Meng-1**	rt	trace	-	-
6	$CHCl_3$	**Meng-2**	rt	89	1:1	-28
7	$CHCl_3$	**Meng-3**	rt	89	1:1	30
8	$CHCl_3$	**Meng-4**	rt	60	1:1.25	26
9	$CHCl_3$	**L5**	rt	-	-	-
10[e]	$CHCl_3$	**L6**	rt	82	-	-
11	$CHCl_3$	**Meng-1**	0	56	1.25:1	32
12[f]	$CHCl_3$	**Meng-1**	-10	62	1:1	52

[a] Unless otherwise stated, reactions were performed with **1a** (60 mg, 0.2 mmol) and **2a** (26 mg, 0.1 mmol), in 1.0 mL of solvent at 25 °C for 6 h. [b] Isolated yield after chromatography. [c] The diastereomeric ratios were determined by column chromatography. [d] Determined by HPLC analysis. [e] Only **4a** was obtained. [f] Reaction was performed with **1b** (63 mg, 0.2 mmol) and **2b** (29 mg, 0.1 mmol).

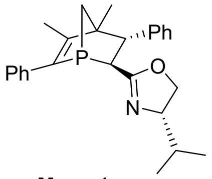

Meng-1

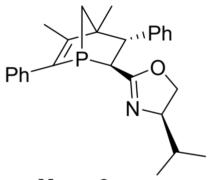

Meng-2

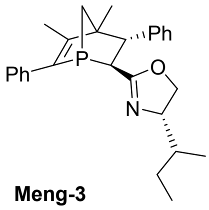

Meng-3

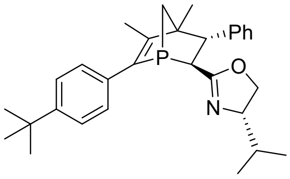

Meng-4

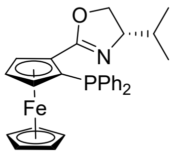

L5

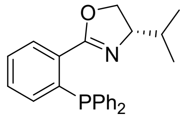

L6

4. General Procedure for Reactions

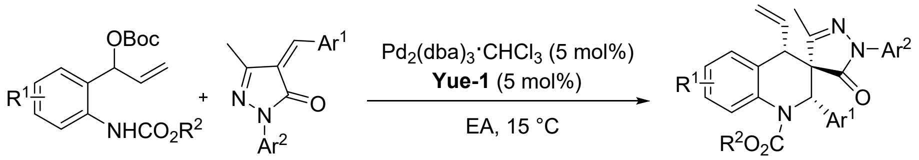

1 **2** **3**

To a dry flask filled with nitrogen were added $Pd_2(dba)_3$•$CHCl_3$ (0.005 mmol) and ligand **Yue-1** (0.005 mmol), then 1 mL EA was added. This solution was stirred at room temperature for 0.5 h. Then, allyl carbonate **1** (0.2 mmol) and alkylidene pyrazolone **2** (0.1 mmol) were added subsequently. The reaction mixture was stirred at 15 °C for 72 h and monitored by TLC (ethyl acetate /petroleum ether). After complete conversion, the product **3** was obtained by chromatography on silica gel (ethyl acetate /petroleum ether).

Methyl (2'*S*,4*R*,4'*S*)-3-methyl-5-oxo-1,2'-diphenyl-4'-vinyl-1,5-dihydro-2'*H*-spiro[pyrazole-4,3'-quinoline]-1'(4'*H*)-carboxylate (3a).

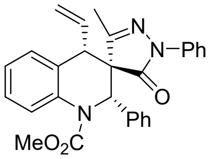

Purified by silica gel chromatography using PE/EA = 4:1, white solid (31 mg, 69% yield). $[\alpha]_D$ = +54 (*c* = 0.1, CH_2Cl_2, 30.1 °C). MP: 176.4 – 177.9 °C. ^{1}H NMR (300 MHz, $CDCl_3$) δ 7.83 (d, *J* = 8.0 Hz, 1H), 7.76 (d, *J* = 8.1 Hz, 2H), 7.48 – 7.37 (m, 3H), 7.23 – 7.18 (m, 6H), 7.11 – 7.08 (m, 2H), 6.01 (s, 1H), 5.52 – 5.48 (m, 2H), 5.39 – 5.35 (m, 1H), 3.99 – 3.96 (m, 1H), 3.71 (s, 3H), 0.97 (s, 3H) ppm; ^{13}C NMR (75 MHz, $CDCl_3$) δ 172.6, 159.3, 155.1, 138.7, 137.8, 137.4, 131.8, 130.3, 128.9, 128.6, 128.0, 127.8, 126.1, 125.7, 125.5, 125.3, 124.5, 123.3, 119.4, 67.6, 63.7, 53.4, 47.3, 16.9 ppm. HRMS (ESI) calcd for $C_{28}H_{26}N_3O_3^+$ $[M+H]^+$: 452.1969, found 452.1942. HPLC: The product was analyzed by HPLC to determine the enantiomeric excess: 96% ee (Chiralpak AD-H, *n*-hexane / *i*-propanol = 90:10, flow rate 1.0 mL/min, λ = 254 nm); tr = 8.98 and 25.36 min.

Using (***R**$_P$*, ***R***)-**Yue-2** as ligand:

3a': Purified by silica gel chromatography using PE/EA = 4:1, white solid (33 mg, 73% yield). $[\alpha]_D$ = -33 (*c* = 0.1, CH_2Cl_2, 26.4 °C). HPLC: The product was analyzed by HPLC to determine the enantiomeric excess: 95% ee (Chiralpak AD-H, *n*-hexane / *i*-propanol = 90:10, flow rate 1.0 mL/min, λ = 254 nm); tr = 8.70 and 22.48 min.

Methyl(2'*S*,4*R*,4'*S*)-6'-methoxy-3-methyl-5-oxo-1,2'-diphenyl-4'-vinyl-1,5-dihydro-2'*H*-spiro[pyrazole-4,3'-quinoline]-1'(4'*H*)-carboxylate (3b).

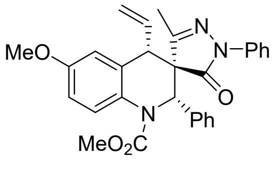

Purified by silica gel chromatography using PE/EA = 4:1, colourless oil(36 mg, 75% yield). $[\alpha]_D$ = +50 (*c* = 0.1, CH_2Cl_2, 30.5 °C). MP: 176.4 – 177.9 °C. ^1H NMR (300 MHz, $CDCl_3$) δ 7.75 (t, *J* = 9.3 Hz, 3H), 7.40 (t, *J* = 7.8 Hz, 2H), 7.23 – 7.18 (m, 4H), 7.10 – 7.08 (m, 2H), 6.97 (dd, *J* = 8.8, 2.8 Hz, 1H), 6.75 (d, *J* = 2.9 Hz, 1H), 6.00 (s, 1H), 5.50 – 5.45 (m, 2H), 5.39 – 5.35 (m, 1H), 3.97 – 3.94 (m, 1H), 3.82 (s, 3H), 3.70 (s, 3H), 1.02 (s, 3H) ppm; ^{13}C NMR (75 MHz, $CDCl_3$) δ 172.7, 159.4, 157.1, 155.2, 138.8, 137.4, 133.3, 130.7, 130.1, 128.9, 128.6, 127.8, 126.7, 125.5, 124.5, 123.6, 119.3, 112.7, 112.2, 67.6, 63.6, 55.6, 53.4, 47.4, 16.9 ppm. HRMS (ESI) calcd for $C_{29}H_{28}N_3O_4^+$ $[M+H]^+$: 482.2074, found 482.2073. HPLC: The product was analyzed by HPLC to determine the enantiomeric excess: 95% ee (Chiralpak AD-H, *n*-hexane / *i*-propanol = 85:15, flow rate 1.0 mL/min, λ = 254 nm); tr = 8.03 and 23.10 min.

Methyl(2'*S*,4*R*,4'*S*)-3,6'-dimethyl-5-oxo-1,2'-diphenyl-4'-vinyl-1,5-dihydro-2'*H*-spiro[pyrazole-4,3'-quinoline]-1'(4'*H*)-carboxylate (3c).

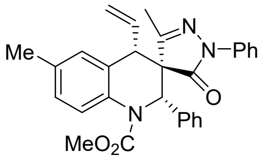

Purified by silica gel chromatography using PE/EA = 4:1, colourless oil (37.4 mg, 82% yield). $[\alpha]_D$ = +40 (*c* = 0.1, CH_2Cl_2, 27.8 °C). ^1H NMR (300 MHz, $CDCl_3$) δ 7.78 – 7.75 (m, 2H), 7.70 (d, *J* = 8.2 Hz, 1H), 7.39 (t, *J* = 8.0 Hz, 2H), 7.26 – 7.15 (m, 5H), 7.11 – 7.07 (m, 2H), 2.1 Hz, 2H), 7.00 (s, 1H), 6.00 (s, 1H), 5.51 – 5.43 (m, 2H), 5.38 – 5.34 (m, 1H), 3.96 – 3.93 (m, 1H), 3.70 (s, 3H), 2.36 (s, 3H), 0.99 (s, 3H) ppm; ^{13}C NMR (75 MHz, $CDCl_3$) δ 172.7, 159.4, 155.1, 138.8, 137.5, 135.2, 135.2, 131.6, 130.4, 128.9, 128.9, 128.6, 127.8, 126.6, 125.5, 124.5, 123.2, 119.4, 67.7, 63.7, 53.4, 47.3, 21.2, 17.0 ppm. HRMS (ESI) calcd for $C_{29}H_{28}N_3O_3^+$ $[M+H]^+$: 466.2125, found 466.2114. HPLC: The product was analyzed by HPLC to determine the enantiomeric excess: 90% ee (Chiralpak AD-H, *n*-hexane / *i*-propanol = 85:15, flow rate 1.0 mL/min, λ = 254 nm); tr = 5.67 and 15.75 min.

Methyl(2'*S*,4*R*,4'*S*)-6'-fluoro-3-methyl-5-oxo-1,2'-diphenyl-4'-vinyl-1,5-dihydro-2'*H*-spiro[pyrazole-4,3'-quinoline]-1'(4'*H*)-carboxylate (3d).

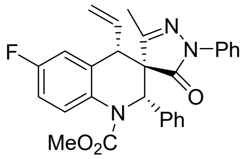

Purified by silica gel chromatography using PE/EA = 4:1, white solid (34 mg, 73% yield). $[\alpha]_D$ = +21 (*c* = 0.1, CH_2Cl_2, 28 °C). MP: 175.6 – 176.8 °C. ^1H NMR (300

MHz, $CDCl_3$) δ 7.82 (dd, J = 8.9, 5.0 Hz, 1H), 7.76 (d, J = 8.1 Hz, 1H), 7.40 (t, J = 7.9 Hz, 1H), 7.24 – 7.12 (m, 5H), 7.07 (dd, J = 7.2, 2.5 Hz, 2H), 6.94 (dd, J = 9.1, 2.9 Hz, 1H), 6.00 (s, 1H), 5.55 – 5.38 (m, 3H), 3.96 – 3.94 (m, 1H), 3.71 (s, 2H), 1.03 (s, 2H) ppm; ^{13}C NMR (75 MHz, $CDCl_3$) δ 172.4, 15.8, 155.0, 138.5, 137.3, 134.0 (d, J = 7.4 Hz), 133.7 (d, J = 3.1 Hz), 129.6, 128.9, 128.7, 127.9, 127.2, 125.6, 124.4, 124.1, 119.3, 114.8 (d, J = 22.7 Hz), 113.2 (d, J = 24.2 Hz), 67.4, 63.6, 53.5, 47.1, 17.0 ppm. HRMS (ESI) calcd for $C_{28}H_{25}FN_3O_3^+$ $[M+H]^+$: 470.1874, found 470.1856. HPLC: The product was analyzed by HPLC to determine the enantiomeric excess: 93% ee (Chiralpak AD-H, *n*-hexane / *i*-propanol = 85:15, flow rate 1.0 mL/min, λ = 254 nm); tr = 7.00 and 20.89 min.

Methyl(2'*S*,4*R*,4'*S*)-7'-fluoro-3-methyl-5-oxo-1,2'-diphenyl-4'-vinyl-1,5-dihydro-2'*H*-spiro[pyrazole-4,3'-quinoline]-1'(4'*H*)-carboxylate (3e).

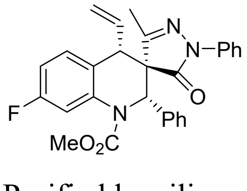

Purified by silica gel chromatography using PE/EA = 4:1, colourless oil (24 mg, 51% yield). $[\alpha]_D$ = +32 (c = 0.1, CH_2Cl_2, 30.5 °C). ^1H NMR (300 MHz, $CDCl_3$) δ 7.77 – 7.73 (m, 2H), 7.64 (dd, J = 10.1, 2.6 Hz, 1H), 7.42 – 7.37 (m, 2H), 7.24 – 7.15 (m, 5H), 7.08 – 7.05 (m, 2H), 6.94 (td, J = 8.4, 2.6 Hz, 1H), 5.97 (s, 1H), 5.53 – 5.43 (m, 2H), 5.40 – 5.36 (m, 1H), 3.94 – 3.91 (m, 1H), 3.71 (s, 3H), 1.05 (s, 3H) ppm; ^{13}C NMR (75 MHz, $CDCl_3$) δ 172.4, 162.2 (d, J = 246.0 Hz), 160.5, 158.9, 154.7, 138.9 (d, J = 10.8 Hz), 138.4, 137.3, 130.1, 128.9, 128.7, 127.9, 127.3 (d, J = 3.1 Hz), 127.1 (d, J = 9.3 Hz), 125.6, 124.3, 123.6, 119.4, 113.2 (d, J = 25.3 Hz), 112.2 (d, J = 21.5 Hz), 67.3, 63.8, 53.6, 46.8, 17.2 ppm. HRMS (ESI) calcd for $C_{28}H_{25}FN_3O_3^+$ $[M+H]^+$: 470.1874, found 470.1885. HPLC: The product was analyzed by HPLC to determine the enantiomeric excess: 90% ee (Chiralpak AD-H, *n*-hexane / *i*-propanol = 85:15, flow rate 1.0 mL/min, λ = 254 nm); tr = 5.71 and 15.24 min.

Methyl(2'*S*,4*R*,4'*S*)-6'-methoxy-2'-(4-methoxyphenyl)-3-methyl-5-oxo-1-phenyl-4'-vinyl-1,5-dihydro-2'*H*-spiro[pyrazole-4,3'-quinoline]-1'(4'*H*)-carboxylate (3f).

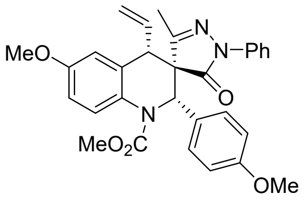

Purified by silica gel chromatography using PE/EA = 4:1, colourless oil (27 mg, 53% yield). $[\alpha]_D$ = +26 (*c* = 0.1, CH_2Cl_2, 30.8 °C). ^1H NMR (300 MHz, $CDCl_3$) δ 7.79 – 7.76 (m, 2H), 7.71 (d, *J* = 8.8 Hz, 1H), 7.40 (t, *J* = 7.9 Hz, 2H), 7.21 (t, *J* = 7.4 Hz, 1H), 7.02 – 6.94 (m, 3H), 6.75 – 6.70 (m, 3H), 5.94 (s, 1H), 5.51 – 5.42 (m, 2H), 5.36 (dd, *J* = 7.1, 4.9 Hz, 1H), 3.95 – 3.92 (m, 1H), 3.82 (s, 3H), 3.72 (s, 3H), 3.71 (s, 3H), 1.05 (s, 3H) ppm; ^{13}C NMR (75 MHz, $CDCl_3$) δ 172.7, 159.5, 158.9, 157.1, 155.2, 137.5, 133.4, 130.8, 130.7, 130.1, 128.9, 126.7, 125.6, 125.5, 123.5, 119.3, 113.9, 112.7, 112.1, 67.6, 63.2, 55.6, 55.1, 53.4, 47.3, 17.0 ppm. HRMS (ESI) calcd for $C_{30}H_{30}N_3O_5^+$ $[M+H]^+$: 512.2180, found 512.2189. HPLC: The product was analyzed by HPLC to determine the enantiomeric excess: 86% ee (Chiralpak AD-H, *n*-hexane / *i*-propanol = 85:15, flow rate 1.0 mL/min, λ = 254 nm); tr = 9.25 and 17.21 min.

Methyl(2'*S*,4*R*,4'*S*)-1-(4-chlorophenyl)-3,6'-dimethyl-5-oxo-2'-phenyl-4'-vinyl-1,5-dihydro-2'*H*-spiro[pyrazole-4,3'-quinoline]-1'(4'*H*)-carboxylate (3g).

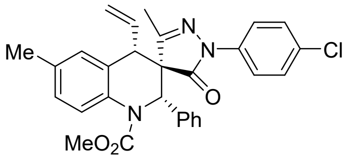

Purified by silica gel chromatography using PE/EA = 4:1, white solid (37 mg, 74% yield). $[\alpha]_D$ = +29 (*c* = 0.1, CH_2Cl_2, 29 °C). MP: 163.1 – 164.6 °C. ^1H NMR (300 MHz, $CDCl_3$) δ 7.78 – 7.73 (m, 2H), 7.70 (d, *J* = 8.2 Hz, 1H), 7.37 – 7.32 (m, 2H), 7.27 – 7.23 (m, 1H), 7.21 – 7.15 (m, 3H), 7.06 (dd, *J* = 7.1, 2.6 Hz, 2H), 6.99 (d, *J* = 1.9 Hz, 1H), 5.98 (s, 1H), 5.59 – 5.41 (m, 2H), 5.37 – 5.33 (m, 1H), 3.95 – 3.92 (m, 1H), 3.70 (s, 3H), 2.37 (s, 3H), 0.98 (s, 3H) ppm; ^{13}C NMR (75 MHz, $CDCl_3$) δ 172.7, 159.8, 155.1, 138.7, 136.0, 135.2, 135.1, 131.4, 130.6, 130.3, 128.9, 128.6, 128.6, 127.8, 126.6, 125.5, 124.4, 123.2, 120.2, 67.8, 63.7, 53.4, 47.3, 21.2, 17.0 ppm. HRMS (ESI) calcd for $C_{29}H_{27}ClN_3O_3^+$ $[M+H]^+$: 500.1735, found 500.1697. HPLC: The product was analyzed by HPLC to determine the enantiomeric excess: 83% ee (Chiralpak AD-H, *n*-hexane / *i*-propanol = 90:10, flow rate 1.0 mL/min, λ = 254 nm); tr = 7.21 and 34.99 min.

Isopropyl(2'*S*,4*R*,4'*S*)-3-methyl-5-oxo-1,2'-diphenyl-4'-vinyl-1,5-dihydro-2'*H*-spiro[pyrazole-4,3'-quinoline]-1'(4'*H*)-carboxylate (3h).

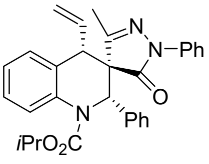

Purified by silica gel chromatography using PE/EA = 4:1, white solid (37 mg, 77% yield). $[\alpha]_D$ = +24 (*c* = 0.1, CH_2Cl_2, 28.8 °C). MP: 150.2 – 151.5 °C. ^{1}H NMR (300 MHz, $CDCl_3$) δ 7.93 (d, *J* = 8.1 Hz, 1H), 7.78 – 7.72 (m, 2H), 7.46 – 7.36 (m, 3H), 7.23 – 7.15 (m, 6H), 7.12 – 7.07 (m, 2H), 5.93 (s, 1H), 5.50 (td, *J* = 5.8, 2.5 Hz, 2H), 5.37 (dd, *J* = 7.1, 4.8 Hz, 1H), 4.91 (p, *J* = 6.2 Hz, 1H), 4.00 (dd, *J* = 5.7, 3.3 Hz, 1H), 1.23 (d, *J* = 6.2 Hz, 3H), 1.04 (s, 3H), 0.89 (d, *J* = 6.2 Hz, 3H) ppm; ^{13}C NMR (75 MHz, $CDCl_3$) δ 172.6, 159.3, 153.9, 139.3, 137.9, 137.4, 131.2, 130.5, 128.9, 128.5, 127.9, 127.6, 126.1, 125.5, 125.3, 124.9, 124.5, 123.2, 119.4,70.3, 67.2, 63.8, 47.2, 22.0, 21.5, 17.0 ppm. HRMS (ESI) calcd for $C_{30}H_{30}N_3O_3^+$ $[M+H]^+$: 480.2282, found 480.2254. HPLC: The product was analyzed by HPLC to determine the enantiomeric excess: 91% ee (Chiralpak AD-H, *n*-hexane / *i*-propanol = 93:7, flow rate 1.0 mL/min, λ = 254 nm); tr = 6.99 and 11.74 min.

Methyl(2'*S*,4*R*,4'*S*)-3-methyl-5-oxo-1-phenyl-2'-(p-tolyl)-4'-vinyl-1,5-dihydro-2'*H*-spiro[pyrazole-4,3'-quinoline]-1'(4'*H*)-carboxylate (3i).

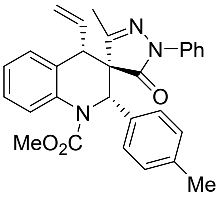

Purified by silica gel chromatography using PE/EA = 4:1, white solid (39 mg, 83% yield). $[\alpha]_D$ = +34 (*c* = 0.1, CH_2Cl_2, 30.1 °C). MP: 133.4 – 135.5 °C. ^{1}H NMR (300 MHz, $CDCl_3$) δ 7.84 – 7.76 (m, 3H), 7.47 – 7.37 (m, 3H), 7.24 – 7.18 (m, 3H), 6.98 – 6.95 (m, 4H), 5.98 (s, 1H), 5.44 – 5.58 (m, 2H), 5.44 – 5.24 (m, 1H), 3.98 – 3.95 (m, 1H), 3.71 (s, 3H), 2.24 (s, 3H), 0.98 (s, 3H) ppm; ^{13}C NMR (75 MHz, $CDCl_3$) δ 172.7, 159..5, 155.7, 137.8, 137.5, 137.4, 135.7, 131.8, 130.3, 129.3, 128.9, 128.0, 128.0, 126.1, 125.7, 125.5, 125.3, 124.3, 123.3, 119.4, 67.7, 63.5, 53.4, 47.3, 21.1, 17.0 ppm. HRMS (ESI) calcd for $C_{29}H_{28}N_3O_3^+$ $[M+H]^+$: 466.2125, found 466.2086. HPLC: The product was analyzed by HPLC to determine the enantiomeric excess: 90% ee (Chiralpak AD-H, *n*-hexane / *i*-propanol = 90:10, flow rate 1.0 mL/min, λ = 254 nm); tr = 8.02 and 15.99 min.

Methyl(2'*S*,4*R*,4'*S*)-2'-(3-methoxyphenyl)-3-methyl-5-oxo-1-phenyl-4'-vinyl-1,5-dihydro-2'*H*-spiro[pyrazole-4,3'-quinoline]-1'(4'*H*)-carboxylate (3j).

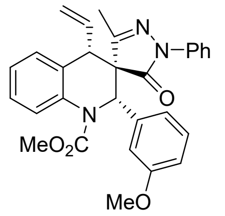

Purified by silica gel chromatography using PE/EA = 4:1, white solid (31 mg, 65% yield). $[\alpha]_D$ = +11 (c = 0.1, CH_2Cl_2, 27.5 °C). MP: 120.3 – 122.2 °C. ^1H NMR (300 MHz, $CDCl_3$) δ 7.83 – 7.77 (m, 3H), 7.47 – 7.36 (m, 3H), 7.22 – 7.18 (m, 3H), 7.11 (t, J = 7.9 Hz, 1H), 6.70 (dd, J = 8.1, 2.2 Hz, 2H), 6.60 (t, J = 2.1 Hz, 1H), 5.98 (s, 1H), 5.52 – 5.48 (m, 2H), 5.39 – 5.35 (m, 1H), 3.98 – 3.95 (m, 1H), 3.72 (s, 3H), 3.55 (s, 3H), 0.99 (s, 3H) ppm; ^{13}C NMR (75 MHz, $CDCl_3$) δ 172.7, 159.7, 159.4, 155.0, 140.4, 137.7, 137.4, 131.7, 130.3, 129.7, 128.9, 128.0, 126.1, 125.7, 125.5, 125.3, 123.3, 119.2, 116.6, 109.8, 67.6, 63.7, 54.9, 53.5, 47.2, 16.9 ppm. HRMS (ESI) calcd for $C_{29}H_{28}N_3O_4^+$ $[M+H]^+$: 482.2074, found 482.2088. HPLC: The product was analyzed by HPLC to determine the enantiomeric excess: 92% ee (Chiralpak AD-H, *n*-hexane / *i*-propanol = 90:10, flow rate 1.0 mL/min, λ = 254 nm); tr = 10.89 and 25.56 min.

Methyl(2'*S*,4*R*,4'*S*)-3-methyl-5-oxo-1-phenyl-2'-(o-tolyl)-4'-vinyl-1,5-dihydro-2'*H*-spiro[pyrazole-4,3'-quinoline]-1'(4'*H*)-carboxylate (3k).

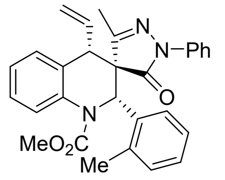

Purified by silica gel chromatography using PE/EA = 4:1, colourless oil (32 mg, 69% yield). $[\alpha]_D$ = +18 (c = 0.1, CH_2Cl_2, 30.9 °C). ^1H NMR (300 MHz, $CDCl_3$) δ 7.80 – 7.75 (m, 3H), 7.47 (dt, J = 8.5, 4.4 Hz, 1H), 7.38 (t, J = 7.9 Hz, 2H), 7.32 – 7.24 (m, 3H), 7.22 – 7.16 (m, 1H), 7.08 – 6.98 (m, 3H), 6.21 (s, 1H), 5.60 – 5.42 (m, 2H), 5.36 (dd, J = 8.8, 3.0 Hz, 1H), 3.98 (d, J = 8.4 Hz, 1H), 3.71 (s, 3H), 2.20 (s, 3H), 1.18 (s, 3H) ppm; ^{13}C NMR (75 MHz, $CDCl_3$) δ 172.5, 159.4, 154.8, 138.5, 137.4, 135.2, 131.9, 131.1, 130.0, 128.9, 128.0, 127.7, 126.1, 125.6, 125.4, 125.4, 125.3, 123.7, 119.0, 67.4, 60.9, 53.4, 47.5, 19.2, 17.6 ppm. HRMS (ESI) calcd for $C_{29}H_{28}N_3O_3^+$ $[M+H]^+$: 466.2125, found 466.2132. HPLC: The product was analyzed by HPLC to determine the enantiomeric excess: 80% ee (Chiralpak AD-H, *n*-hexane / *i*-propanol = 90:10, flow rate 1.0 mL/min, λ = 254 nm); tr = 6.19 and 11.69 min.

Methyl(2'*S*,4*R*,4'*S*)-2'-(4-fluorophenyl)-3-methyl-5-oxo-1-phenyl-4'-vinyl-1,5-dihydro-2'*H*-spiro[pyrazole-4,3'-quinoline]-1'(4'*H*)-carboxylate (3l).

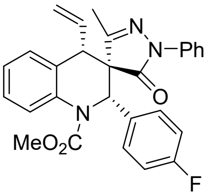

Purified by silica gel chromatography using PE/EA = 4:1, white solid (28.4 mg, 60% yield). $[\alpha]_D$ = +34 (*c* = 0.1, CH_2Cl_2, 27.3 °C). MP: 165.1 – 166.5 °C. ^1H NMR (300 MHz, $CDCl_3$) δ 7.81 – 7.74 (m, 3H), 7.48 – 7.37 (m, 3H), 7.25 – 7.19 (m, 3H), 7.08 (dd, *J* = 8.6, 5.2 Hz, 2H), 6.89 (t, *J* = 8.6 Hz, 2H), 5.96 (s, 1H), 5.49 (td, *J* = 5.8, 2.6 Hz, 2H), 5.38 (dd, *J* = 7.1, 4.8 Hz, 1H), 3.97 (dd, *J* = 5.6, 3.3 Hz, 1H), 3.72 (s, 3H), 0.99 (s, 3H) ppm; ^{13}C NMR (75 MHz, $CDCl_3$) δ 172.4, 163.7, 159.1, 155.0, 137.6, 137.3, 134.6 (d, *J* = 3.3 Hz), 131.6, 130.2, 128.9, 128.1, 126.2, 126.1, 125.5, 123.4, 119.3, 115.8, 115.5, 67.5, 63.3, 53.5, 47.2, 17.0 ppm. HRMS (ESI) calcd for $C_{28}H_{25}FN_3O_3^+$ $[M+H]^+$: 470.1874, found 470.1874. HPLC: The product was analyzed by HPLC to determine the enantiomeric excess: 90% ee (Chiralpak AD-H, *n*-hexane / *i*-propanol = 85:15, flow rate 1.0 mL/min, λ = 254 nm); tr = 6.51 and 13.94 min.

Methyl(2'*S*,4*R*,4'*S*)-2'-(3-fluorophenyl)-3-methyl-5-oxo-1-phenyl-4'-vinyl-1,5-dihydro-2'*H*-spiro[pyrazole-4,3'-quinoline]-1'(4'*H*)-carboxylate (3m).

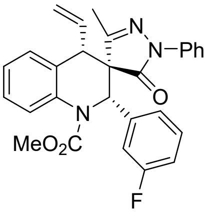

Purified by silica gel chromatography using PE/EA = 4:1, colourless oil (25.9 mg, 55% yield). $[\alpha]_D$ = +50 (*c* = 0.1, CH_2Cl_2, 30.5 °C). ^1H NMR (300 MHz, $CDCl_3$) δ 7.81 (d, *J* = 8.2 Hz, 1H), 7.75 (d, *J* = 8.1 Hz, 2H), 7.48 – 7.38 (m, 3H), 7.26 – 7.16 (m, 4H), 6.91 – 6.84 (m, 3H), 5.98 (s, 1H), 5.52 – 5.48 (m, 2H), 5.40 – 5.37 (m, 1H), 3.98 – 3.95 (m, 1H), 3.73 (s, 3H), 1.00 (s, 3H) ppm; ^{13}C NMR (75 MHz, $CDCl_3$) δ 172.4, 158.9, 155.0, 137.4, 137.3, 131.5, 130.4 (d, *J* = 8.1 Hz), 130.1, 128.9, 128.2, 126.2, 125.7, 125.7, 125.5, 123.5, 120.4 (d, *J* = 3.1 Hz),119.4, 118.8, 114.8 (d, *J* = 21.4 Hz), 111.6 (d, *J* = 23.0 Hz), 67.8, 63.3, 53.6, 47.3, 16.9 ppm. HRMS (ESI) calcd for $C_{28}H_{25}FN_3O_3^+$ $[M+H]^+$: 470.1874, found 470.1874. HPLC: The product was analyzed by HPLC to determine the enantiomeric excess: 93% ee (Chiralpak AD-H, *n*-hexane / *i*-propanol = 85:15, flow rate 1.0 mL/min, λ = 254 nm); tr = 6.96 and 13.49 min.

Methyl(2'*S*,4*R*,4'*S*)-2'-(4-chlorophenyl)-3-methyl-5-oxo-1-phenyl-4'-vinyl-1,5-dihydro-2'*H*-spiro[pyrazole-4,3'-quinoline]-1'(4'*H*)-carboxylate (3n).

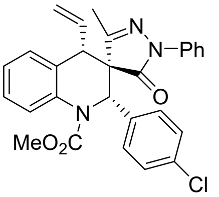

Purified by silica gel chromatography using PE/EA = 4:1, colourless oil (26.3 mg, 54% yield). $[\alpha]_D$ = +13 (*c* = 0.1, CH_2Cl_2, 30.4 °C). ^{1}H NMR (300 MHz, $CDCl_3$) δ 7.81 – 7.74 (m, 3H), 7.48 – 7.37 (m, 3H), 7.24 – 7.16 (m, 6H), 7.04 (d, *J* = 8.4 Hz, 2H), 5.95 (s, 1H), 5.49 (td, *J* = 5.7, 2.5 Hz, 2H), 5.38 (dd, *J* = 7.1, 4.8 Hz, 1H), 3.97 (dd, *J* = 5.7, 3.3 Hz, 1H), 3.72 (s, 3H), 0.99 (s, 3H) ppm; ^{13}C NMR (75 MHz, $CDCl_3$) δ 172.4, 158.9, 155.0, 137.5, 137.4, 137.3, 133.5, 131.5, 130.1, 128.9, 128.1, 126.2, 125.9, 125.7, 125.6, 125.5, 123.5, 119.3, 67.4, 63.3, 53.5, 47.2, 17.0 ppm. HRMS (ESI) calcd for $C_{28}H_{25}ClN_3O_3^+$ $[M+H]^+$: 486.1579, found 486.1588. HPLC: The product was analyzed by HPLC to determine the enantiomeric excess: 90% ee (Chiralpak AD-H, *n*-hexane / *i*-propanol = 90:10, flow rate 1.0 mL/min, λ = 254 nm); tr = 8.44 and 17.03 min.

Mmethyl(2'*S*,4*R*,4'*S*)-3-methyl-2'-(naphthalen-2-yl)-5-oxo-1-phenyl-4'-vinyl-1,5-dihydro-2'*H*-spiro[pyrazole-4,3'-quinoline]-1'(4'*H*)-carboxylate (3o).

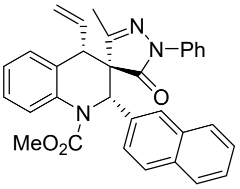

Purified by silica gel chromatography using PE/EA = 4:1, colourless oil (36.2 mg, 72% yield). $[\alpha]_D$ = +62 (*c* = 0.1, CH_2Cl_2, 29.9 °C). ^{1}H NMR (300 MHz, $CDCl_3$) δ 7.94 (d, *J* = 8.0 Hz, 1H), 7.75 – 7.72 (m, 3H), 7.69 – 7.63 (m, 2H), 7.57 (d, *J* = 1.8 Hz, 1H), 7.52 – 7.46 (m, 1H), 7.44 – 7.37 (m, 4H), 7.25 – 7.15 (m, 4H), 6.17 (s, 1H), 5.54 – 5.45 (m, 2H), 5.39 (dd, *J* = 7.1, 4.7 Hz, 1H), 4.04 (dd, *J* = 5.8, 3.3 Hz, 1H), 3.69 (s, 3H), 0.95 (s, 3H) ppm; ^{13}C NMR (75 MHz, $CDCl_3$) δ 172.7, 159.3, 155.1, 137.9, 137.4, 133.1, 132.9, 131.8, 130.3, 128.9, 128.7, 128.1, 127.9, 127.7, 126.4, 126.2, 126.1, 125.7, 125.6, 125.4, 123.4, 123.3, 122.6, 119.5, 67.7, 63.8, 53.5, 47.3, 17.0 ppm. HRMS (ESI) calcd for $C_{32}H_{28}N_3O_3^+$ $[M+H]^+$: 502.2125, found 502.2142. HPLC: The product was analyzed by HPLC to determine the enantiomeric excess: 85% ee (Chiralpak AD-H, *n*-hexane / *i*-propanol = 85:15, flow rate 1.0 mL/min, λ = 254 nm); tr = 9.20 and 18.64 min.

Methyl(2'*S*,4*R*,4'*S*)-1-(4-chlorophenyl)-3-methyl-5-oxo-2'-phenyl-4'-vinyl-1,5-dihydro-2'*H*-spiro[pyrazole-4,3'-quinoline]-1'(4'*H*)-carboxylate (3p).

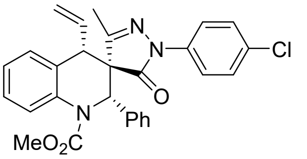

Purified by silica gel chromatography using PE/EA = 4:1, white solid (30 mg, 61% yield). $[\alpha]_D$ = +13 (*c* = 0.1, CH_2Cl_2, 27.7 °C). MP: 159.7 – 161.2 °C. ^1H NMR (300 MHz, $CDCl_3$) δ 7.83 (d, *J* = 8.0 Hz, 1H), 7.78 – 7.73 (m, 2H), 7.45 (ddd, *J* = 9.9, 6.4, 2.2 Hz, 1H), 7.36 – 7.34 (m, 2H), 7.23 – 7.16 (m, 5H), 7.07 (dd, *J* = 7.0, 2.6 Hz, 2H), 5.99 (s, 1H), 5.48 (td, *J* = 5.8, 4.9, 2.8 Hz, 2H), 5.40 – 5.29 (m, 1H), 4.05 – 3.92 (m, 1H), 3.71 (s, 3H), 0.97 (s, 3H) ppm; ^{13}C NMR (75 MHz, $CDCl_3$) δ 172.6, 159.6, 155.0, 138.6, 137.7, 136.0, 131.6, 130.6, 130.2, 128.9, 128.7, 128.1, 127.9, 126.1, 125.7, 125.4, 124.4, 123.4, 120.2, 67.7, 63.7, 53.5, 47.3, 16.9 ppm. HRMS (ESI) calcd for $C_{28}H_{25}ClN_3O_3^+$ $[M+H]^+$: 486.1579, found 486.1565. HPLC: The product was analyzed by HPLC to determine the enantiomeric excess: 90% ee (Chiralpak AD-H, *n*-hexane / *i*-propanol = 85:15, flow rate 1.0 mL/min, λ = 254 nm); tr = 7.64 and 30.34 min.

Methyl(2'*S*,4*R*,4'*S*)-3-methyl-5-oxo-2'-phenyl-1-(p-tolyl)-4'-vinyl-1,5-dihydro-2'*H*-spiro[pyrazole-4,3'-quinoline]-1'(4'*H*)-carboxylate (3q).

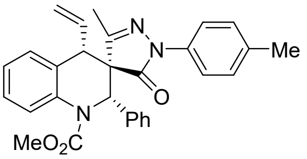

Purified by silica gel chromatography using PE/EA = 4:1, white solid (38.2 mg, 82% yield). $[\alpha]_D$ = +31 (*c* = 0.1, CH_2Cl_2, 28.5 °C). MP: 185.9 – 187.3 °C. ^1H NMR (300 MHz, $CDCl_3$) δ 7.83 (d, *J* = 8.0 Hz, 1H), 7.63 (d, *J* = 8.5 Hz, 2H), 7.47 – 7.41 (m, 1H), 7.23 – 7.17 (m, 7H), 7.10 – 7.07 (m, 2H), 6.00 (s, 1H), 5.52 – 5.44 (m, 2H), 5.39 – 5.35 (m, 1H), 3.98 – 3.95 (m, 1H), 3.71 (s, 3H), 2.35 (s, 3H), 0.96 (s, 3H) ppm; ^{13}C NMR (75 MHz, $CDCl_3$) δ 172.4, 159.1, 155.1, 138.8, 137.8, 135.3, 135.0, 131.8, 130.3, 129.4, 128.6, 128.0, 127.8, 126.1, 125.7, 125.3, 124.5, 123.2, 119.4, 67.5, 63.7, 53.4, 47.2, 21.0, 16.9 ppm. HRMS (ESI) calcd for $C_{29}H_{28}N_3O_3^+$ $[M+H]^+$: 466.2125, found 466.2108. HPLC: The product was analyzed by HPLC to determine the enantiomeric excess: 90% ee (Chiralpak AD-H, *n*-hexane / *i*-propanol = 85:15, flow rate 1.0 mL/min, λ = 254 nm); tr = 9.56 and 48.00 min.

Methyl(2'*S*,4*R*,4'*S*)-1-(3,4-dimethylphenyl)-3-methyl-5-oxo-2'-phenyl-4'-vinyl-1,5-dihydro-2'*H*-spiro[pyrazole-4,3'-quinoline]-1'(4'*H*)-carboxylate (3r).

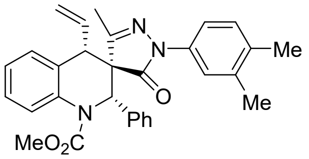

Purified by silica gel chromatography using PE/EA = 3:1, white solid (38.1 mg, 79% yield). $[\alpha]_D$ = +19 (c = 0.1, CH_2Cl_2, 29.1 °C). MP: 162.2 – 163.4 °C. ^1H NMR (300 MHz, $CDCl_3$) δ 7.83 (d, J = 8.0 Hz, 1H), 7.53 – 7.41 (m, 3H), 7.22 – 7.08 (m, 8H), 6.00 (s, 1H), 5.54 – 5.44 (m, 2H), 5.39 – 5.35 (m, 1H), 3.98 – 3.95 (m, 1H), 3.71 (s, 3H), 2.28 (s, 3H), 2.26 (s, 3H), 0.96 (s, 3H) ppm; ^{13}C NMR (75 MHz, $CDCl_3$) δ 172.4, 159.1, 155.1, 138.8, 137.8, 137.2, 135.3, 134.1, 131.8, 130.3, 129.9, 128.6, 128.0, 127.8, 126.1, 125.7, 125.3, 124.5, 123.3, 120.5, 117.0, 67.5, 63.7, 53.4, 47.2, 20.0, 19.3, 16.9 ppm. HRMS (ESI) calcd for $C_{30}H_{30}N_3O_3^+$ $[M+H]^+$: 480.2282, found 480.2254. HPLC: The product was analyzed by HPLC to determine the enantiomeric excess: 89% ee (Chiralpak AD-H, *n*-hexane / *i*-propanol = 85:15, flow rate 1.0 mL/min, λ = 254 nm); tr = 5.67 and 18.44 min.

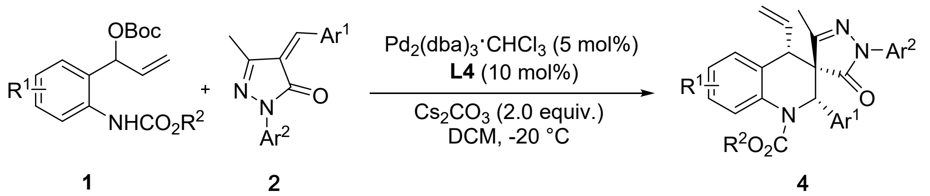

To a dry flask filled with nitrogen were added $Pd_2(dba)_3$•$CHCl_3$ (0.005 mmol) and ligand **L4** (0.01 mmol), then 1 mL DCM was added. This solution was stirred at room temperature for 0.5 h. Then, allyl carbonate **1** (0.2 mmol), alkylidene pyrazolone **2** (0.1 mmol) and Cs_2CO_3 (65.2 mg, 0.2 mmol, 2.0 equiv.) were added subsequently. The reaction mixture was stirred at -20 °C for 72 h and monitored by TLC. After complete conversion, the product **4** was obtained by chromatography on silica gel.

Methyl(2'*S*,4*S*,4'*S*)-3-methyl-5-oxo-1,2'-diphenyl-4'-vinyl-1,5-dihydro-2'*H*-spiro[pyrazole-4,3'-quinoline]-1'(4'*H*)-carboxylate (4a).

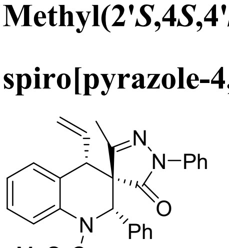

Purified by silica gel chromatography using PE/EA = 4:1, white solid (38.4 mg, 85% yield). Yield: 85%. $[\alpha]_D$ = +94 (c = 0.1, CH_2Cl_2, 28.6 °C). MP: 82.6 – 83.9 °C. ^1H

NMR (300 MHz, $CDCl_3$) δ 7.70 (dd, J = 8.1, 1.2 Hz, 1H), 7.46 – 7.41 (m, 1H), 7.25 – 7.09 (m, 11H), 7.03 – 6.98 (m, 1H), 5.72 (s, 1H), 5.62 (dt, J = 17.3, 9.6 Hz, 1H), 5.47 – 5.41 (m, 2H), 3.91 (d, J = 9.6 Hz, 1H), 3.73 (s, 3H), 2.43 (s, 3H) ppm; ^{13}C NMR (75 MHz, $CDCl_3$) δ 169.9, 157.8, 155.2, 137.7, 137.5, 136.9, 131.6, 130.4, 128.4, 128.3, 127.9, 127.7, 126.7, 126.0, 125.1, 122.0, 119.3, 65.2, 61.7, 53.4, 46.6, 14.3 ppm. HRMS (ESI) calcd for $C_{28}H_{26}N_3O_3^+$ $[M+H]^+$: 452.1969, found 452.1992. HPLC: The product was analyzed by HPLC to determine the enantiomeric excess: 92% ee (Chiralpak AD-H, *n*-hexane / *i*-propanol = 93:7, flow rate 1.0 mL/min, λ = 254 nm); tr = 8.62 and 17.19 min.

Using (***S***$_a$, ***S*,*S***)-**L1** as ligand:

4a': Purified by silica gel chromatography using PE/EA = 4:1, white solid (31 mg, 69% yield). $[\alpha]_D$ = -147 (c = 0.1, CH_2Cl_2, 26.6 °C). HPLC: The product was analyzed by HPLC to determine the enantiomeric excess: 91% ee (Chiralpak AD-H, *n*-hexane / *i*-propanol = 93:7, flow rate 1.0 mL/min, λ = 254 nm); tr = 8.63 and16.88 min.

Methyl(22'*S*,4*S*,4'*S*)-6'-methoxy-3-methyl-5-oxo-1,2'-diphenyl-4'-vinyl-1,5-dihydro-2'*H*-spiro[pyrazole-4,3'-quinoline]-1'(4'*H*)-carboxylate (4b).

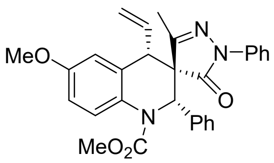

Purified by silica gel chromatography using PE/EA = 3:1, colourless oil (32.3 mg, 67% yield). $[\alpha]_D$ = +110 (c = 0.1,, CH_2Cl_2, 30.1 °C). ^1H NMR (300 MHz, $CDCl_3$) δ 7.62 (d, J = 8.8 Hz, 1H), 7.27 – 7.23 (m, 3H), 7.19 – 7.11 (m, 6H), 7.06 – 7.01 (m, 1H), 6.97 (dd, J = 8.8, 2.9 Hz, 1H), 6.71 – 6.70 (m, 1H), 5.74 (s, 1H), 5.62 (dt, J = 17.5, 9.3 Hz, 1H), 5.46 (dq, J = 13.0, 1.9 Hz, 2H), 3.90 (d, J = 9.5 Hz, 1H), 3.81 (s, 3H), 3.75 (s, 3H), 2.45 (s, 3H) ppm; ^{13}C NMR (75 MHz, $CDCl_3$) δ 169.9, 157.9, 156.9, 155.4, 137.8, 136.9, 131.9, 131.5, 130.5, 128.4, 128.3, 127.9, 126.0, 125.9, 125.1, 122.2, 119.3, 112.9, 112.3, 65.1, 61.4, 55.3, 53.4, 46.6, 14.3 ppm. HRMS (ESI) calcd for $C_{29}H_{28}N_3O_4^+$ $[M+H]^+$: 482.2074, found 482.2078. HPLC: The product was analyzed by HPLC to determine the enantiomeric excess: 95% ee (Chiralpak AD-H, *n*-hexane / *i*-propanol = 90:10, flow rate 1.0 mL/min, λ = 254 nm); tr = 6.91 and 7.80 min.

Methyl(2'*S*,4*S*,4'*S*)-6'-chloro-3-methyl-5-oxo-1,2'-diphenyl-4'-vinyl-1,5-dihydro-2'*H*-spiro[pyrazole-4,3'-quinoline]-1'(4'*H*)-carboxylate (4c).

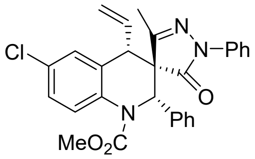

Purified by silica gel chromatography using PE/EA = 4:1, colourless oil (34.5 mg, 71% yield). $[\alpha]_D$ = +163 (*c* = 0.1, CH_2Cl_2, 30.1 °C). ^{1}H NMR (300 MHz, $CDCl_3$) δ 7.64 (d, *J* = 8.6 Hz, 1H), 7.38 (dd, *J* = 8.6, 2.4 Hz, 1H), 7.22 – 7.10 (m, 10H), 7.05 – 7.00 (m, 1H), 5.68 (s, 1H), 5.63 – 5.42 (m, 3H), 3.88 (d, *J* = 8.9 Hz, 1H), 3.73 (s, 3H), 2.42 (s, 3H) ppm; ^{13}C NMR (75 MHz, $CDCl_3$) δ 169.8, 157.5, 154.9, 137.3, 136.7, 136.1, 132.2, 130.9, 130.6, 128.5, 128.3, 128.1, 127.8, 126.9, 126.1, 126.0, 125.3, 122.7, 119.4, 64.8, 61.7, 53.5, 46.3, 14.3 ppm. HRMS (ESI) calcd for $C_{28}H_{25}ClN_3O_3^+$ $[M+H]^+$: 486.1579, found 486.1552. HPLC: The product was analyzed by HPLC to determine the enantiomeric excess: 94% ee (Chiralpak AD-H, *n*-hexane / *i*-propanol = 90:10, flow rate 1.0 mL/min, λ = 254 nm); tr = 6.66 and 7.83 min.

Methyl(*2'S,4S,4'S*)-6'-bromo-3-methyl-5-oxo-1,2'-diphenyl-4'-vinyl-1,5-dihydro-2'*H*-spiro[pyrazole-4,3'-quinoline]-1'(4'*H*)-carboxylate (4d).

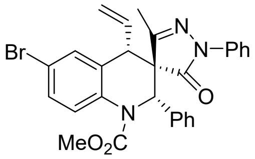

Purified by silica gel chromatography using PE/EA = 4:1, colourless oil (22.1 mg, 42% yield). $[\alpha]_D$ = +143 (*c* = 0.1, CH_2Cl_2, 31.0 °C). ^{1}H NMR (300 MHz, $CDCl_3$) δ 7.61 – 7.52 (m, 2H), 7.25 – 7.11 (m, 10H), 7.06 – 7.00 (m, 1H), 5.67 (s, 1H), 5.60 – 5.43 (m, 3H), 3.88 (d, *J* = 8.9 Hz, 1H), 3.73 (s, 3H), 2.42 (s, 3H) ppm; ^{13}C NMR (75 MHz, $CDCl_3$) δ 169.8, 157.5, 154.8, 137.3, 136.7, 132.5, 130.9, 130.7, 129.7, 128.5, 128.3, 128.1, 126.4, 125.9, 125.3, 122.8, 119.4, 118.5, 64.8, 61.7, 53.5, 46.3, 14.3 ppm. HRMS (ESI) calcd for $C_{28}H_{25}BrN_3O_3^+$ $[M+H]^+$: 530.1074, found 530.1076. HPLC: The product was analyzed by HPLC to determine the enantiomeric excess: 91% ee (Chiralpak AD-H, *n*-hexane / *i*-propanol = 90:10, flow rate 1.0 mL/min, λ = 254 nm); tr = 8.58 and 10.41 min.

Methyl(2'*S*,4*S*,4'*S*)-7'-chloro-3-methyl-5-oxo-1,2'-diphenyl-4'-vinyl-1,5-dihydro-2'*H*-spiro[pyrazole-4,3'-quinoline]-1'(4'*H*)-carboxylate (4e).

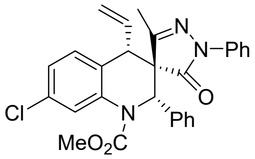

Purified by silica gel chromatography using PE/EA = 4:1, colourless oil (40.1 mg, 82% yield). $[\alpha]_D$ = +167 (*c* = 0.1, CH_2Cl_2, 30.1 °C). ^{1}H NMR (300 MHz, $CDCl_3$) δ 7.73 (d, *J* = 2.0 Hz, 1H), 7.23 – 7.10 (m, 10H), 7.06 – 6.99 (m, 2H), 5.67 (s, 1H), 5.58 (dt, *J* = 18.1, 9.1 Hz, 1H), 5.44 (dq, *J* = 12.6, 2.1 Hz, 2H), 3.86 (d, *J* = 9.3 Hz, 1H), 3.74 (s, 3H), 2.42 (s, 3H) ppm; ^{13}C NMR (75 MHz, $CDCl_3$) δ 169.9, 157.6, 154.8, 138.5, 137.3, 136.8, 133.2, 131.2, 128.8,128.5, 128.4, 128.1, 127.6, 126.0, 125.3, 125.2, 125.1, 122.5, 119.4, 64.9, 61.8, 53.6, 46.2, 14.3 ppm. HRMS (ESI) calcd for $C_{28}H_{25}ClN_3O_3^+$ $[M+H]^+$: 486.1579, found 486.1586. HPLC: The product was analyzed by HPLC to determine the enantiomeric excess: 98% ee (Chiralpak AD-H, *n*-hexane / *i*-propanol = 90:10, flow rate 1.0 mL/min, λ = 254 nm); tr = 5.59 and 11.66 min.

Benzyl(2'*S*,4*S*,4'*S*)-3-methyl-5-oxo-1,2'-diphenyl-4'-vinyl-1,5-dihydro-2'*H*-spiro[pyrazole-4,3'-quinoline]-1'(4'*H*)-carboxylate (4f).

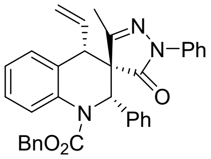

Purified by silica gel chromatography using PE/EA = 3:1, colourless oil (37 mg, 70% yield). $[\alpha]_D$ = +187 (*c* = 0.1, CH_2Cl_2, 30.0 °C). ^{1}H NMR (300 MHz, $CDCl_3$) δ 7.75 (d, *J* = 8.0 Hz, 1H),, 7.41 (td, *J* = 7.7, 1.7 Hz, 1H), 7.27 – 7.22 (m, 5H), 7.19 – 7.08 (m, 11H), 7.03 – 6.97 (m, 1H), 5.71 (s, 1H), 5.61 (dt, *J* = 17.2, 9.6 Hz, 1H), 5.46 – 5.40 (m, 2H), 5.17 (s, 2H), 3.92 (d, *J* = 9.6 Hz, 1H), 2.40 (s, 3H) ppm; ^{13}C NMR (75 MHz, $CDCl_3$) δ 169.9, 157.7, 154.5, 137.8, 137.5, 136.9, 135.8, 131.7, 130.1, 128.4, 128.4, 128.3, 128.0, 127.9, 127.7, 127.6, 126.7, 126.1, 125.1, 125.0, 125.0, 122.0, 119.3, 68.0, 65.0, 61.8, 46.6, 14.3 ppm. HRMS (ESI) calcd for $C_{34}H_{29}N_3NaO_3^+$ $[M+H]^+$: 550.2101, found 550.2139. HPLC: The product was analyzed by HPLC to determine

the enantiomeric excess: 93% ee (Chiralpak AD-H, *n*-hexane / *i*-propanol = 90:10, flow rate 1.0 mL/min, λ = 254 nm); tr = 8.47 and 23.67 min.

Methyl(2'*S*,4*S*,4'*S*)-3-methyl-5-oxo-1-phenyl-2'-(p-tolyl)-4'-vinyl-1,5-dihydro-2'*H*-spiro[pyrazole-4,3'-quinoline]-1'(4'*H*)-carboxylate (4g).

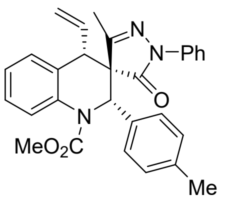

Purified by silica gel chromatography using PE/EA = 4:1, white solid (41 mg, 87% yield). $[\alpha]_D$ = +122 (*c* = 0.1, CH_2Cl_2, 29.0 °C). MP: 78.3 – 79.6 °C. ^1H NMR (300 MHz, $CDCl_3$) δ 7.68 (d, J = 8.0 Hz, 1H), 7.42 (t, J = 7.7 Hz, 1H), 7.28 – 7.12 (m, 6H), 7.05 – 6.94 (m, 5H), 5.68 (s, 1H), 5.64 – 5.55 (m, 1H), 5.46 – 5.40 (m, 2H), 3.90 (d, J = 9.7 Hz, 1H), 3.73 (s, 3H), 2.41 (s, 3H), 2.16 (s, 3H) ppm; ^{13}C NMR (75 MHz, $CDCl_3$) δ 170.0, 157.9, 155.2, 137.5, 137.0, 134.7, 131.7, 130.4, 128.9, 128.4, 127.6, 126.7, 126.0, 125.1, 125.0, 121.9, 119.3, 65.2, 61.6, 53.4, 46.7, 21.0, 14.3 ppm. HRMS (ESI) calcd for $C_{29}H_{28}N_3O_4^+$ $[M+H]^+$: 452.1969, found 452.1992. HPLC: The product was analyzed by HPLC to determine the enantiomeric excess: 95% ee (Chiralpak AD-H, *n*-hexane / *i*-propanol = 90:10, flow rate 1.0 mL/min, λ = 254 nm); tr = 6.58 and 13.44 min.

Methyl(2'*S*,4*S*,4'*S*)-2'-(4-methoxyphenyl)-3-methyl-5-oxo-1-phenyl-4'-vinyl-1,5-dihydro-2'*H*-spiro[pyrazole-4,3'-quinoline]-1'(4'*H*)-carboxylate (4h).

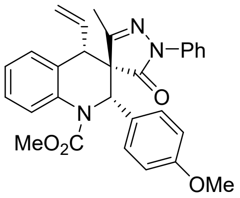

Purified by silica gel chromatography using PE/EA = 3:1, white solid (40 mg, 83% yield). $[\alpha]_D$ = +104 (*c* = 0.1, CH_2Cl_2, 28.8 °C). MP: 163.5 – 165.2 °C. ^1H NMR (300 MHz, $CDCl_3$) δ 7.66 (dd, J = 8.1, 1.2 Hz, 1H), 7.45 – 7.40 (m, 1H), 7.31 – 7.28 (m, 2H), 7.24 – 7.12 (m, 4H), 7.10 – 6.99 (m, 3H), 6.70 – 6.68 (m, 2H), 5.67 (s, 1H), 5.65 – 5.56 (m, 1H), 5.46 – 5.40 (m, 2H), 3.90 (d, J = 9.7 Hz, 1H), 3.73 (s, 3H), 3.64 (s, 3H), 2.41 (s, 3H) ppm; ^{13}C NMR (75 MHz, $CDCl_3$) δ 170.0, 159.0, 157.9, 155.2,

137.5, 137.0, 131.7, 130.4, 129.8, 128.4, 127.6, 127.4, 126.7, 125.1, 125.0, 121.9, 119.3, 113.7, 65.3, 61.4, 55.2, 53.4, 46.6, 14.3 ppm. HRMS (ESI) calcd for $C_{29}H_{28}N_3O_4^+$ [M+H]$^+$: 482.2074, found 482.2089. HPLC: The product was analyzed by HPLC to determine the enantiomeric excess: 97% ee (Chiralpak AD-H, *n*-hexane / *i*-propanol = 90:10, flow rate 1.0 mL/min, λ = 254 nm); tr = 9.91 and 25.75 min.

Methyl(2'*S*,4*S*,4'*S*)-2'-(3-methoxyphenyl)-3-methyl-5-oxo-1-phenyl-4'-vinyl-1,5-dihydro-2'*H*-spiro[pyrazole-4,3'-quinoline]-1'(4'*H*)-carboxylate (4i).

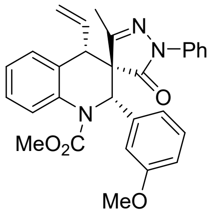

Purified by silica gel chromatography using PE/EA = 3:1, white solid (34.2 mg, 71% yield). $[\alpha]_D$ = +154 (*c* = 0.1, CH_2Cl_2, 23.0 °C). MP: 128.5 – 129.6 °C. ^{1}H NMR (300 MHz, $CDCl_3$) δ 7.68 (dd, *J* = 8.1, 1.2 Hz, 1H), 7.42 (td, *J* = 7.9, 1.4 Hz, 1H), 7.32 – 7.28 (m, 2H), 7.21 – 6.99 (m, 6H), 6.75 – 6.72 (m, 1H), 6.69 (t, *J* = 2.1 Hz, 1H), 6.64 – 6.60 (m, 1H), 5.70 (s, 1H), 5.62 (dt, *J* = 17.3, 9.6 Hz, 1H), 5.43 (dq, *J* = 12.5, 1.8 Hz, 2H), 3.89 (d, *J* = 9.7 Hz, 1H), 3.74 (s, 3H), 3.63 (s, 3H), 2.42 (s, 3H) ppm; ^{13}C NMR (75 MHz, $CDCl_3$) δ 169.8, 159.4, 157.8, 155.2, 139.3, 137.5, 137.0, 131.6, 130.4, 129.3, 128.4, 127.7, 126.7, 125.1, 125.0, 125.0, 122.0, 119.2, 118.3, 113.3, 111.9, 65.1, 61.6, 55.1, 53.4, 46.7, 14.3 ppm. HRMS (ESI) calcd for $C_{29}H_{28}N_3O_4^+$ [M+H]$^+$: 482.2074, found 482.2049. HPLC: The product was analyzed by HPLC to determine the enantiomeric excess: 94% ee (Chiralpak AD-H, *n*-hexane / *i*-propanol = 90:10, flow rate 1.0 mL/min, λ = 254 nm); tr = 8.22 and 15.44 min.

Methyl(2'*S*,4*S*,4'*S*)-2'-(2-methoxyphenyl)-3-methyl-5-oxo-1-phenyl-4'-vinyl-1,5-dihydro-2'*H*-spiro[pyrazole-4,3'-quinoline]-1'(4'*H*)-carboxylate (4j).

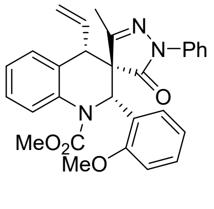

Purified by silica gel chromatography using PE/EA = 3:1, colourless oil (26.9 mg, 56% yield). $[\alpha]_D$ = +189 (c = 0.1, CH_2Cl_2, 31.0 °C). ^1H NMR (300 MHz, $CDCl_3$) δ 7.68 (d, J = 8.0 Hz, 1H), 7.43 (ddd, J = 8.8, 7.4, 1.6 Hz, 1H), 7.33 – 7.29 (m, 3H), 7.21 – 7.13 (m, 4H), 7.07 – 6.98 (m, 2H), 6.81 (td, J = 7.6, 1.1 Hz, 1H), 6.66 (dd, J = 8.3, 1.1 Hz, 1H), 6.26 (s, 1H), 5.61 (dt, J = 17.3, 9.6 Hz, 1H), 5.46 – 5.39 (m, 2H), 3.89 (d, J = 9.7 Hz, 1H), 3.74 (s, 3H), 3.71 (s, 3H), 2.38 (s, 3H) ppm; ^{13}C NMR (75 MHz, $CDCl_3$) δ 170.0, 159.6, 155.8, 155.1, 137.9, 137.2, 131.7, 130.9, 128.7, 128.4, 128.2, 127.5, 126.8, 126.7, 125.1, 124.8, 121.9, 120.5, 119.1, 109.9, 64.6, 55.6, 54.9, 53.4, 46.8, 14.3 ppm. HRMS (ESI) calcd for $C_{29}H_{28}N_3O_4^+$ $[M+H]^+$: 482.2074, found 482.2078. HPLC: The product was analyzed by HPLC to determine the enantiomeric excess: 90% ee (Chiralpak AD-H, *n*-hexane / *i*-propanol = 93:7 flow rate 1.0 mL/min, λ = 254 nm); tr = 7.89 and 10.45 min.

Methyl(2'*S*,4*S*,4'*S*)-2'-(4-ethoxyphenyl)-3-methyl-5-oxo-1-phenyl-4'-vinyl-1,5-dihydro-2'*H*-spiro[pyrazole-4,3'-quinoline]-1'(4'*H*)-carboxylate (4k).

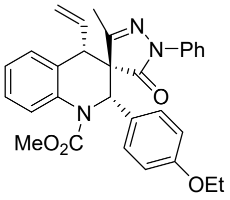

Purified by silica gel chromatography using PE/EA = 3:1, colourless oil (32.4 mg, 66% yield). $[\alpha]_D$ = +108 (c = 0.1, CH_2Cl_2, 29.9 °C). ^1H NMR (300 MHz, $CDCl_3$) δ 7.67 (dd, J = 8.0, 1.2 Hz, 1H), 7.45 – 7.39 (m, 1H), 7.31 – 7.28 (m, 2H), 7.23 – 7.12 (m, 5H), 7.08 – 6.99 (m, 3H), 6.68 (d, J = 8.7 Hz, 2H), 5.68– 5.56 (m, 2H), 5.46 – 5.40 (m, 2H), 3.91 – 3.83 (m, 3H), 3.73 (s, 3H), 2.41 (s, 3H), 1.28 (t, J = 6.9 Hz, 3H) ppm; ^{13}C NMR (75 MHz, $CDCl_3$) δ 170.0, 158.3, 157.9, 155.2, 137.5, 137.0, 131.7, 130.4, 129.6, 128.4, 127.6, 127.4, 126.7, 125.1, 121.9, 119.3, 114.3, 65.3, 63.3, 61.4, 53.4, 46.6, 14.7, 14.3 ppm. HRMS (ESI) calcd for $C_{30}H_{30}N_3O_4^+$ $[M+H]^+$: 496.2231, found 496.2210. HPLC: The product was analyzed by HPLC to determine the enantiomeric excess: 94% ee (Chiralpak AD-H, *n*-hexane / *i*-propanol = 90:10 flow rate 1.0 mL/min, λ = 254 nm); tr = 10.36 and 32.75 min.

Methyl(2'*S*,4*S*,4'*S*)-2'-(3,4-dimethoxyphenyl)-3-methyl-5-oxo-1-phenyl-4'-vinyl-1,5-dihydro-2'*H*-spiro[pyrazole-4,3'-quinoline]-1'(4'*H*)-carboxylate (4l).

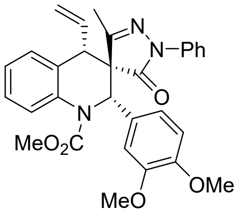

Purified by silica gel chromatography using PE/EA = 2:1, colourless oil (33.2 mg, 65% yield). $[\alpha]_D$ = +112 (*c* = 0.1, CH_2Cl_2, 30.0 °C). ^{1}H NMR (300 MHz, $CDCl_3$) δ 7.65 (dd, *J* = 8.0, 1.2 Hz, 1H), 7.45 – 7.40 (m, 1H), 7.35 – 7.32 (m, 2H), 7.23 – 7.13 (m, 4H), 7.05 – 6.99 (m, 1H), 6.74 (dd, *J* = 8.2, 2.1 Hz, 1H), 6.67 – 6.64 ((m, 2H), 5.69 (s, 1H), 5.65 – 5.56 (m, 1H), 5.47 – 5.40 (m, 2H), 3.89 (d, *J* = 9.7 Hz, 1H), 3.75 (s, 3H), 3.72 (s, 3H), 3.69 (s, 3H), 2.42 (s, 3H) ppm; ^{13}C NMR (75 MHz, $CDCl_3$) δ 170.0, 158.0, 155.2, 148.6, 148.4, 137.5, 137.0, 131.6, 130.6, 130.2, 128.5, 127.6, 126.7, 125.2, 125.0, 125.0, 122.0, 119.0, 118.6, 110.7, 109.5, 65.4, 61.5, 55.8, 55.7, 53.4, 46.7, 14.3 ppm. HRMS (ESI) calcd for $C_{30}H_{30}N_3O_5^+$ $[M+H]^+$: 512.2180, found 512.2147. HPLC: The product was analyzed by HPLC to determine the enantiomeric excess: 96% ee (Chiralpak AD-H, *n*-hexane / *i*-propanol = 85:15 flow rate 1.0 mL/min, λ = 254 nm); tr = 10.36 and 32.75 min.

Methyl(2'*S*,4*S*,4'*S*)-3-methyl-2'-(4-nitrophenyl)-5-oxo-1-phenyl-4'-vinyl-1,5-dihydro-2'*H*-spiro[pyrazole-4,3'-quinoline]-1'(4'*H*)-carboxylate (4m).

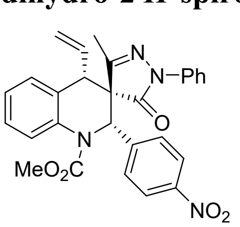

Purified by silica gel chromatography using PE/EA = 4:1, colourless oil (35 mg, 70% yield). $[\alpha]_D$ = +70 (*c* = 0.1, CH_2Cl_2, 29.3 °C). ^{1}H NMR (300 MHz, $CDCl_3$) δ 8.04 (d, *J* = 8.8 Hz, 2H), 7.68 (dd, *J* = 8.0, 1.2 Hz, 1H), 7.50 – 7.44 (m, 1H), 7.34 – 7.23 (m, 6H), 7.19 – 7.13 (m, 3H), 7.06 – 7.00 (m, 1H), 5.78 (s, 1H), 5.61 (dt, *J* = 18.1, 9.1 Hz, 1H), 5.48 (dq, *J* = 13.1, 2.0 Hz, 2H), 3.94 (d, *J* = 9.4 Hz, 1H), 3.76 (s, 3H), 2.46 (s, 3H) ppm; ^{13}C NMR (75 MHz, $CDCl_3$) δ 169.4, 157.4, 155.2, 147.3, 145.3, 136.9,

136.6, 131.0, 129.8, 128.6, 128.0, 127.2, 126.9, 125.5, 125.4, 124.9, 123.6, 122.6, 118.8, 64.8, 61.1, 53.7, 46.7, 14.3 ppm. HRMS (ESI) calcd for $C_{28}H_{25}N_4O_5^+$ [M+H]$^+$: 497.1819, found 497.1822. HPLC: The product was analyzed by HPLC to determine the enantiomeric excess: 84% ee (Chiralpak AD-H, *n*-hexane / *i*-propanol = 80:20 flow rate 1.0 mL/min, λ = 254 nm); tr = 9.16 and 27.69 min.

Methyl(2'*S*,4*S*,4'*S*)-2'-(4-cyanophenyl)-3-methyl-5-oxo-1-phenyl-4'-vinyl-1,5-dihydro-2'*H*-spiro[pyrazole-4,3'-quinoline]-1'(4'*H*)-carboxylate (4n).

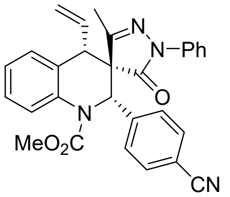

Purified by silica gel chromatography using PE/EA = 4:1, white solid (38 mg, 79% yield). [α]$_D$ = +96 (*c* = 0.1, CH_2Cl_2, 29.9 °C). MP: 87.9 – 89.5 °C. ^{1}H NMR (300 MHz, $CDCl_3$) *δ* 7.68 – 7.65 (m, 1H), 7.49 – 7.43 (m, 3H), 7.27 – 7.24 (m, 5H), 7.22 – 7.14 (m, 3H), 7.08 – 7.03 (m, 1H), 5.73 (s, 1H), 5.67 – 5.55 (m, 1H), 5.49 – 5.43 (m, 2H), 3.92 (d, *J* = 9.4 Hz, 1H), 3.76 (s, 3H), 2.44 (s, 3H) ppm; ^{13}C NMR (75 MHz, $CDCl_3$) *δ* 169.5, 157.5, 155.2, 143.3, 136.9, 136.6, 132.1, 131.1, 129.9, 128.6, 127.9, 127.0, 123.9, 125.5, 124.9, 122.5, 118.9, 118.4, 111.8, 64.9, 61.2, 53.6, 46.6, 14.3 ppm. HRMS (ESI) calcd for $C_{29}H_{25}N_4O_3^+$ [M+H]$^+$: 477.1921, found 477.1907. HPLC: The product was analyzed by HPLC to determine the enantiomeric excess: 86% ee (Chiralpak AD-H, *n*-hexane / *i*-propanol = 85:15, flow rate 1.0 mL/min, λ = 254 nm); tr = 8.71 and 27.78 min.

Methyl(2'*S*,4*S*,4'*S*)-3-methyl-5-oxo-1-phenyl-2'-(4-(trifluoromethyl)phenyl)-4'-vinyl-1,5-dihydro-2'*H*-spiro[pyrazole-4,3'-quinoline]-1'(4'*H*)-carboxylate (4o).

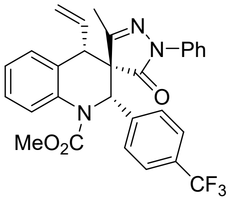

Purified by silica gel chromatography using PE/EA = 4:1, white solid (30.1 mg, 58% yield). [α]$_D$ = +84 (*c* = 0.1, CH_2Cl_2, 29.0 °C). MP: 76.5 – 78.4 °C. ^{1}H NMR (300 MHz, $CDCl_3$) *δ* 7.69 – 7.66 (m, 1H), 7.44 (d, *J* = 8.0 Hz, 3H), 7.28 – 7.23 (m, 3H), 7.21 –

7.12 (m, 5H), 7.06 – 7.00 (m, 1H), 5.75 (s, 1H), 5.68 – 5.56 (m, 1H), 5.50 – 5.43 (m, 2H), 3.93 (d, *J* = 9.6 Hz, 1H), 3.75 (s, 3H), 2.44 (s, 3H) ppm; ^{13}C NMR (75 MHz, $CDCl_3$) δ 169.7, 157.6, 155.2, 148.3, 141.9, 137.1, 136.6, 131.3, 130.1,130.1(d, *J* = 32.5 Hz), 129.8, 128.5, 127.8, 126.8, 126.6, 125.5, 125.4, 125.3, 125.0, 122.3, 119.3, 64.9, 61.2, 53.6, 46.6, 14.3 ppm. HRMS (ESI) calcd for $C_{29}H_{25}F_3N_3O_3^+$ $[M+H]^+$: 520.1843, found 520.1863. HPLC: The product was analyzed by HPLC to determine the enantiomeric excess: 92% ee (Chiralpak AD-H, *n*-hexane / *i*-propanol = 90:10, flow rate 1.0 mL/min, λ = 254 nm); tr = 8.49 and 11.39 min.

Methyl(2'*S*,4*S*,4'*S*)-2'-(4-fluorophenyl)-3-methyl-5-oxo-1-phenyl-4'-vinyl-1,5-dihydro-2'*H*-spiro[pyrazole-4,3'-quinoline]-1'(4'*H*)-carboxylate (4p).

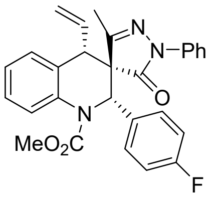

Purified by silica gel chromatography using PE/EA = 4:1, white solid (37.2 mg, 79% yield). $[\alpha]_D$ = +131 (*c* = 0.1, CH_2Cl_2, 29.6 °C). MP: 105.6 – 107.4 °C. ^{1}H NMR (300 MHz, $CDCl_3$) δ 7.66 (dd, *J* = 8.1, 1.2 Hz, 1H), 7.44 (dddd, *J* = 8.1, 7.3, 1.7, 0.8 Hz, 1H), 7.30 – 7.27 (m, 2H), 7.22 – 7.11 (m, 6H), 7.06 – 7.00 (m, 1H), 6.89 – 6.83 (m, 2H), 5.69 (s, 1H), 5.64 – 5.55 (m, 1H), 5.44 (dq, *J* = 12.7, 1.8 Hz, 2H), 3.91 (d, *J* = 9.6 Hz, 1H), 3.74 (s, 3H), 2.42 (s, 3H) ppm; ^{13}C NMR (75 MHz, $CDCl_3$) δ 169.8, 157.7, 155.2, 137.3, 136.8, 133.6, 133.5, 131.5, 130.3, 128.5, 128.0, 127.9, 127.7, 126.8, 125.2, 125.2, 125.0, 122.1, 119.1, 115.4, 115.1, 65.2, 61.1, 53.5, 46.6, 14.3 ppm. HRMS (ESI) calcd for $C_{28}H_{25}FN_3O_3^+$ $[M+H]^+$: 470.1874, found 470.1887. HPLC: The product was analyzed by HPLC to determine the enantiomeric excess: 90% ee (Chiralpak AD-H, *n*-hexane / *i*-propanol = 90:10, flow rate 1.0 mL/min, λ = 254 nm); tr = 6.29 and 13.30 min.

Methyl(2'*S*,4*S*,4'*S*)-2'-(4-chlorophenyl)-3-methyl-5-oxo-1-phenyl-4'-vinyl-1,5-dihydro-2'*H*-spiro[pyrazole-4,3'-quinoline]-1'(4'*H*)-carboxylate (4q).

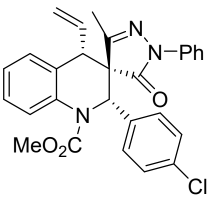

Purified by silica gel chromatography using PE/EA = 4:1, white solid (31.7 mg, 65% yield). $[\alpha]_D$ = +114 (*c* = 0.1, CH_2Cl_2, 29.8 °C). MP: 177.4 – 178.6 °C. ^{1}H NMR (300 MHz, $CDCl_3$) δ 7.66 (dd, *J* = 8.0, 1.3 Hz, 1H), 7.44 (ddd, *J* = 8.8, 7.7, 1.4 Hz, 1H), 7.30 – 7.26 (m, 2H), 7.22 – 7.01 (m, 9H), 5.67 (s, 1H), 5.64 – 5.55 (m, 1H), 5.45 (dq, *J* = 13.1, 1.8 Hz, 2H), 3.90 (d, *J* = 9.5 Hz, 1H), 3.74 (s, 3H), 2.42 (s, 3H) ppm; ^{13}C NMR (75 MHz, $CDCl_3$) δ 169.7, 157.6, 155.1, 137.2, 136.8, 136.4, 133.7, 131.4, 130.2, 128.5, 127.8, 127.6, 126.8, 125.3, 125.0, 122.2, 119.2, 65.0, 61.1, 53.5, 46.7, 14.3 ppm. HRMS (ESI) calcd for $C_{28}H_{25}ClN_3O_3^+$ $[M+H]^+$: 486.1579, found 486.1572. HPLC: The product was analyzed by HPLC to determine the enantiomeric excess: 90% ee (Chiralpak AD-H, *n*-hexane / *i*-propanol = 90:10, flow rate 1.0 mL/min, λ = 254 nm); tr = 7.97 and 14.89 min.

Methyl(2'*S*,4*S*,4'*S*)-2'-(3-chlorophenyl)-3-methyl-5-oxo-1-phenyl-4'-vinyl-1,5-dihydro-2'*H*-spiro[pyrazole-4,3'-quinoline]-1'(4'*H*)-carboxylate (4r).

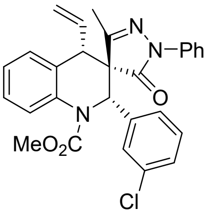

Purified by silica gel chromatography using PE/EA = 4:1, white solid (38.4 mg, 79% yield). $[\alpha]_D$ = +112 (*c* = 0.1, CH_2Cl_2, 29.1 °C). MP: 75.4 – 76.9 °C. ^{1}H NMR (300 MHz, $CDCl_3$) δ 7.68 (dd, *J* = 8.1, 1.2 Hz, 1H), 7.47 – 7.42 (m, 1H), 7.33 – 7.29 (m, 2H), 7.23 – 7.01 (m, 9H), 5.67 (s, 1H), 5.64 – 5.56 (m, 1H), 5.45 (dq, *J* = 13.6, 1.9 Hz, 2H), 3.90 (d, *J* = 9.6 Hz, 1H), 3.75 (s, 3H), 2.42 (s, 3H) ppm; ^{13}C NMR (75 MHz, $CDCl_3$) δ 169.6, 157.6, 155.2, 139.8, 137.2, 136.8, 134.1, 131.4, 130.1, 129.6, 128.5, 128.2, 127.8, 126.7, 126.2, 125.3, 125.2, 125.1, 124.4, 122.2, 119.1, 64.9, 61.1, 53.5, 46.6, 14.3 ppm. HRMS (ESI) calcd for $C_{28}H_{25}ClN_3O_3^+$ $[M+H]^+$: 486.1579, found 486.1595. HPLC: The product was analyzed by HPLC to determine the enantiomeric

excess: 93% ee (Chiralpak AD-H, *n*-hexane / *i*-propanol = 90:10, flow rate 1.0 mL/min, λ = 254 nm); tr = 6.04 and 12.16 min.

Methyl(2'*S*,4*S*,4'*S*)-3-methyl-2'-(naphthalen-2-yl)-5-oxo-1-phenyl-4'-vinyl-1,5-dihydro-2'*H*-spiro[pyrazole-4,3'-quinoline]-1'(4'*H*)-carboxylate (4s).

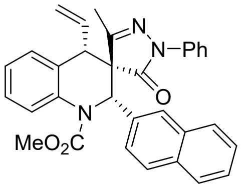

Purified by silica gel chromatography using PE/EA = 3:1, white solid (33 mg, 66% yield). $[\alpha]_D$ = +95 (*c*= 0.1, CH_2Cl_2, 30.0 °C). MP: 108.8 – 110.2 °C. ^{1}H NMR (300 MHz, $CDCl_3$) δ 7.77 (dd, *J* = 8.1, 1.2 Hz, 1H), 7.73 – 7.70 (m, 1H), 7.67 – 7.61 (m, 3H), 7.51 – 7.45 (m, 1H), 7.39 – 7.32 (m, 2H), 7.29 – 7.21 (m, 3H), 7.18 – 7.13 (m, 3H), 7.05 – 6.99 (m, 2H), 6.94 – 6.88 (m, 1H), 5.89 (s, 1H), 5.63 (dt, *J* = 16.9, 9.6 Hz, 1H), 5.49 – 5.42 (m, 2H), 3.96 (d, *J* = 9.6 Hz, 1H), 3.71 (s, 3H), 2.49 (s, 3H) ppm; ^{13}C NMR (75 MHz, $CDCl_3$) δ 169.9, 157.9, 155.3, 137.6, 136.8, 135.3, 132.9, 132.8, 131.5, 130.4, 128.3, 128.2, 128.1, 127.8, 127.5, 126.7, 126.0, 126.0, 125.4, 125.2, 125.0, 124.1, 122.1, 119.2, 65.2, 61.9, 53.4, 46.9, 14.4 ppm. HRMS (ESI) calcd for $C_{32}H_{28}N_3O_3^+$ [M+H]$^+$: 502.2125, found 502.2086. HPLC: The product was analyzed by HPLC to determine the enantiomeric excess: 90% ee (Chiralpak AD-H, *n*-hexane / *i*-propanol = 87:13, flow rate 1.0 mL/min, λ = 254 nm); tr = 9.82 and 12.28 min.

Methyl(2'*R*,4*S*,4'*S*)-3-methyl-5-oxo-1-phenyl-2'-(thiophen-3-yl)-4'-vinyl-1,5-dihydro-2'*H*-spiro[pyrazole-4,3'-quinoline]-1'(4'*H*)-carboxylate (4t).

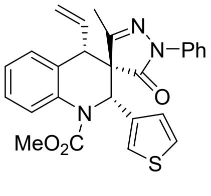

Purified by silica gel chromatography using PE/EA = 4:1, white solid (40 mg, 88% yield). $[\alpha]_D$ = +123 (*c* = 0.1, CH_2Cl_2, 30.1 °C). MP: 161.2 – 162.5 °C. ^{1}H NMR (300 MHz, $CDCl_3$) δ 7.62 (dd, *J* = 8.0, 1.2 Hz, 1H), 7.44 – 7.41 (m, 1H), 7.36 – 7.33 (m, 2H), 7.21 – 7.12 (m, 4H), 7.10 – 7.01 (m, 3H), 6.83 (dd, *J* = 4.9, 1.5 Hz, 1H), 5.86 (s, 1H), 5.63 (dt, *J* = 17.2, 9.6 Hz, 1H), 5.42 (dq, *J* = 13.0, 1.6 Hz, 2H), 3.87 (d, *J* = 9.7

Hz, 1H), 3.75 (s, 3H), 2.39 (s, 3H) ppm; ^{13}C NMR (75 MHz, $CDCl_3$) δ 170.0, 158.1, 155.2, 138.7, 137.1, 137.1, 131.5, 130.6, 128.5, 127.7, 126.7, 125.9, 125.5, 125.3, 125.2, 125.1, 122.0, 121.6, 119.3, 65.0, 58.1, 53.5, 46.5, 14.2 ppm. HRMS (ESI) calcd for $C_{26}H_{24}N_3O_3S^+$ $[M+H]^+$: 458.1533, found 458.1541. HPLC: The product was analyzed by HPLC to determine the enantiomeric excess: 92% ee (Chiralpak AD-H, *n*-hexane / *i*-propanol = 90:10, flow rate 1.0 mL/min, λ = 254 nm); tr = 8.34 and 11.38 min.

Methyl(2'*S*,4*S*,4'*S*)-3-methyl-5-oxo-2'-phenyl-1-(p-tolyl)-4'-vinyl-1,5-dihydro-2'*H*-spiro[pyrazole-4,3'-quinoline]-1'(4'*H*)-carboxylate (4u).

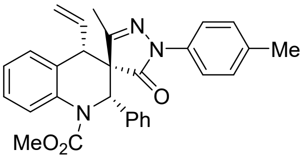

Purified by silica gel chromatography using PE/EA = 4:1, white solid (73 mg, 92% yield). $[\alpha]_D$ = +192 (*c* = 0.1, CH_2Cl_2, 30.0 °C). MP: 149.3 – 150.2 °C. ^1H NMR (300 MHz, $CDCl_3$) δ 7.69 (dd, *J* = 8.0, 1.1 Hz, 1H), 7.45 – 7.40 (m, 1H), 7.20 – 7.08 (m, 9H), 6.93 (d, *J* = 8.3 Hz, 2H), 5.71 (s, 1H), 5.61 (dt, *J* = 17.2, 9.6 Hz, 1H), 5.46 – 5.40 (m, 2H), 3.90 (d, *J* = 9.7 Hz, 1H), 3.73 (s, 3H), 2.42 (s, 3H), 2.20 (s, 3H) ppm; ^{13}C NMR (75 MHz, $CDCl_3$) δ 169.7, 157.6, 155.2, 137.7, 137.5, 134.8, 134.5, 131.7, 130.4, 128.9, 128.2, 127.9, 127.7, 126.7, 126.0, 125.1, 125.1, 121.9, 119.3, 65.1, 61.7, 53.4, 46.6, 20.9, 14.3 ppm. HRMS (ESI) calcd for $C_{29}H_{28}N_3O_3^+$ $[M+H]^+$: 466.2125, found 466.2126. HPLC: The product was analyzed by HPLC to determine the enantiomeric excess: 94% ee (Chiralpak AD-H, *n*-hexane / *i*-propanol = 90:10, flow rate 1.0 mL/min, λ = 254 nm); tr = 7.32 and 10.32 min.

Methyl(2'*S*,4*S*,4'*S*)-1-(4-chlorophenyl)-3-methyl-5-oxo-2'-phenyl-4'-vinyl-1,5-dihydro-2'*H*-spiro[pyrazole-4,3'-quinoline]-1'(4'*H*)-carboxylate (4v).

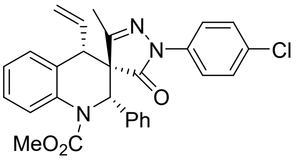

Purified by silica gel chromatography using PE/EA = 4:1, colourless oil (25 mg, 52% yield). $[\alpha]_D$ = +135 (*c* = 0.1, CH_2Cl_2, 30.1 °C). ^1H NMR (300 MHz, $CDCl_3$) δ 7.70 (d,

J = 8.0 Hz, 1H), 7.44 (t, J = 7.4 Hz, 1H), 7.25 – 7.08 (m, 11H), 5.71 (s, 1H), 5.60 (dt, J = 17.1, 9.4 Hz, 1H), 5.47 – 5.41 (m, 2H), 3.91 (d, J = 9.5 Hz, 1H), 3.74 (s, 3H), 2.43 (s, 3H) ppm; ^{13}C NMR (75 MHz, $CDCl_3$) δ 169.9, 158.2, 155.1, 137.5, 137.5, 135.5, 131.5, 130.2, 130.1, 128.4, 128.3, 128.0, 127.7, 126.7, 126.0, 125.1, 125.1, 122.1, 120.1, 65.3, 61.7, 53.4, 46.5, 14.3 ppm. HRMS (ESI) calcd for $C_{28}H_{25}ClN_3O_3^+$ $[M+H]^+$: 486.1579, found 486.1583. HPLC: The product was analyzed by HPLC to determine the enantiomeric excess: 90% ee (Chiralpak AD-H, *n*-hexane / *i*-propanol = 90:10, flow rate 1.0 mL/min, λ = 254 nm); tr = 7.43 and 12.55 min.

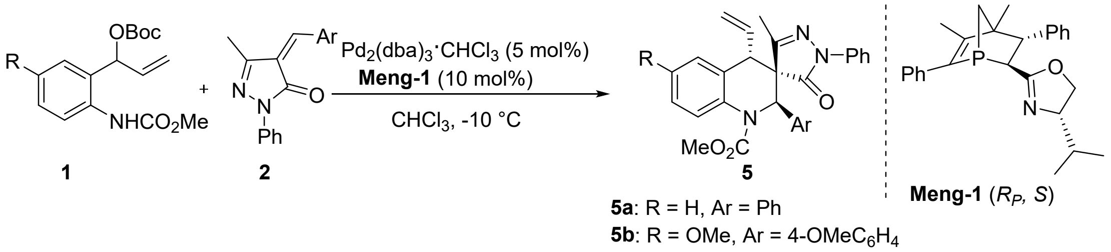

To a dry flask filled with nitrogen were added $Pd_2(dba)_3$•$CHCl_3$ (0.005 mmol) and ligand **Meng-1** (0.01 mmol), then 1 mL $CHCl_3$ was added. This solution was stirred at room temperature for 0.5 h. Then, allyl carbonate **1** (0.2 mmol), alkylidene pyrazolone **2** (0.1 mmol) were added subsequently. The reaction mixture was stirred at -10 °C for 48 h and monitored by TLC (ethyl acetate /petroleum ether). After complete conversion, the product **5** was obtained by chromatography on silica gel.

Methyl(2'S,4R,4'R)-3-methyl-5-oxo-1,2'-diphenyl-4'-vinyl-1,5-dihydro-2'H-spiro[pyrazole-4,3'-quinoline]-1'(4'H)-carboxylate (5a)

Purified by silica gel chromatography using PE/EA = 4:1, white solid (32 mg, 62% yield). $[\alpha]_D$ = +112 (c = 0.1, CH_2Cl_2, 27.3 °C). ^{1}H NMR (300 MHz, $CDCl_3$) δ 7.87 – 7.79 (m, 3H), 7.45 – 7.37 (m, 3H), 7.22 – 7.17 (m, 6H), 7.12 – 7.09 (m, 2H), 6.30 (ddd, J = 17.1, 10.2, 7.0 Hz, 1H), 5.89 (s, 1H), 5.28 (dt, J = 10.2, 1.2 Hz, 1H), 5.01 (dt, J = 17.1, 1.3 Hz, 1H), 3.71 (s, 3H), 3.60 (d, J = 7.0 Hz, 1H), 1.10 (s, 3H) ppm; ^{13}C NMR (75 MHz, $CDCl_3$) δ 171.6, 160.7, 155.3, 138.5, 137.7, 137.4, 132.4, 129.7, 128.9, 128.8, 128.6, 128.2, 128.1, 126.1, 125.6, 125.3, 119.4, 119.0, 63.2, 60.2, 53.5, 46.8, 16.6 ppm. HRMS (ESI) calcd for $C_{28}H_{26}N_3O_3^+$ $[M+H]^+$: 452.1969, found 452.2003. HPLC: The product was analyzed by HPLC to determine the enantiomeric excess: 32% ee (Chiralpak AD-H, *n*-hexane / *i*-propanol = 90:10, flow rate 1.0 mL/min, λ = 254 nm); tr = 16.61 and 19.22 min.

Using ($\boldsymbol{R_P}$, ***R***)-**Meng-2** as ligand:

5a': Purified by silica gel chromatography using PE/EA = 4:1, white solid (38 mg, 80% yield). $[\alpha]_D$ = -99 (*c* = 0.1, CH_2Cl_2, 27.5 °C). HPLC: The product was analyzed by HPLC to determine the enantiomeric excess: 28% ee (Chiralpak AD-H, *n*-hexane / *i*-propanol = 90:10, flow rate 1.0 mL/min, λ = 254 nm); tr = 17.41 and 19.87 min.

Methyl(2'*S*,4*R*,4'*R*)-6'-methoxy-2'-(4-methoxyphenyl)-3-methyl-5-oxo-1-phenyl-4'-vinyl-1,5-dihydro-2'*H*-spiro[pyrazole-4,3'-quinoline]-1'(4'*H*)-carboxylate(5b).

Purified by silica gel chromatography using PE/EA = 4:1, yellow oil (32 mg, 62% yield). $[\alpha]_D$ = +87 (*c* = 0.1, CH_2Cl_2, 26.8 °C). ^{1}H NMR (300 MHz, $CDCl_3$) *δ* 7.87 (d, *J* = 8.1 Hz, 2H), 7.67 (d, *J* = 9.0 Hz, 1H), 7.39 (t, *J* = 7.8 Hz, 2H), 7.19 (t, *J* = 7.4 Hz, 1H), 7.01 (d, *J* = 8.4 Hz, 2H), 6.95 (dd, *J* = 9.0, 3.0 Hz, 1H), 6.74 (dd, *J* = 5.8, 2.8 Hz, 3H), 6.20 (ddd, *J* = 17.3, 10.2, 7.4 Hz, 1H), 5.27 (d, *J* = 10.1 Hz, 1H), 5.05 (d, *J* = 17.0 Hz, 1H), 3.82 (s, 3H), 3.73 (s, 3H), 3.71 (s, 3H), 3.56 (d, *J* = 7.3 Hz, 1H), 1.20 (s, 3H) ppm; ^{13}C NMR (75 MHz, $CDCl_3$) *δ* 171.7, 160.7, 159.2, 156.9, 155.5, 137.8, 132.6, 130.6, 130.5, 130.4, 128.8, 127.2, 127.1, 125.2, 119., 119.0, 114.1, 113.7, 113.3, 62.7, 59.5, 55.6, 55.2, 53.5, 46.5 ppm. HPLC: The product was analyzed by HPLC to determine the enantiomeric excess: 50% ee (Chiralpak AD-H, *n*-hexane / *i*-propanol = 85:15, flow rate 1.0 mL/min, λ = 254 nm); tr = 18.79 and 24.00 min.

Using ($\boldsymbol{R_P}$, ***R***)-**Meng-2** as ligand:

5b': Purified by silica gel chromatography using PE/EA = 4:1, yellow oil (30 mg, 58% yield). $[\alpha]_D$ = -69 (*c* = 0.1, CH_2Cl_2, 27 °C). HRMS (ESI) calcd for $C_{30}H_{30}N_3O_5^+$ $[M+H]^+$: 512.2180, found 512.2186. HPLC: The product was analyzed by HPLC to determine the enantiomeric excess: 52% ee (Chiralpak AD-H, *n*-hexane / *i*-propanol = 85:15, flow rate 1.0 mL/min, λ = 254 nm); tr = 16.43 and 21.03 min.

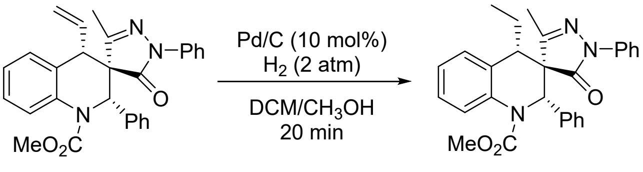

3a
ee = 94%

6
96% yield
92% ee

The compound **3a** (0.1 mmol, 45.1 mg), Pd/C (10%, 0.1 mmol, 10.6mg, 1.0 equiv) was dissolved in 3 mL of DCM and 3 mL of MeOH. Under H_2 at two atmosphere, the solution was stirred at room temperature for 20 min. After complete conversion, the

mixture was concentrated and the product **6** was obtained by chromatography on silica gel.

Methyl(2'*S*,4*R*,4'*S*)-4'-ethyl-3-methyl-5-oxo-1,2'-diphenyl-1,5-dihydro-2'*H*-spiro[pyrazole-4,3'-quinoline]-1'(4'*H*)-carboxylate (6)

Purified by silica gel chromatography using PE/EA = 4:1, colorless oil (43 mg, 96% yield). $[\alpha]_D$ = +71 (*c* = 0.1, CH_2Cl_2, 17 °C). ^1H NMR (300 MHz, $CDCl_3$) δ 7.89 (d, *J* = 8.1 Hz, 2H), 7.84 (d, *J* = 8.2 Hz, 1H), 7.50 – 7.42 (m, 3H), 7.35 – 7.25 (m, 3H), 7.22 – 7.17 (m, 3H), 7.09 – 7.06 (m, 2H), 6.04 (s, 1H), 3.74 (s, 3H), 3.24 (d, *J* = 10.4 Hz, 1H), 1.72 (ddd, *J* = 14.1, 10.6, 7.0 Hz, 1H), 1.55 – 1.46 (m, 1H), 1.18 (t, *J* = 7.3 Hz, 3H), 0.88 (s, 3H) ppm; ^{13}C NMR (75 MHz, $CDCl_3$) δ 173.5, 160.4, 155.0, 139.0, 138.8, 137.5, 132.3, 129.0, 128.6, 127.8, 127.7, 126.0, 125.5, 124.4, 119.1, 69.0, 65.0, 53.4, 43.8, 19.5, 16.8, 13.1 ppm. HRMS (ESI) calcd for $C_{28}H_{28}N_3O_3^+$ $[M+H]^+$: 454.2125, found 454.2132. HPLC: The product was analyzed by HPLC to determine the enantiomeric excess: 92% ee (Chiralpak AD-H, *n*-hexane / *i*-propanol = 85:15, flow rate 1.0 mL/min, λ = 254 nm); tr = 6.78 and 17.59 min.

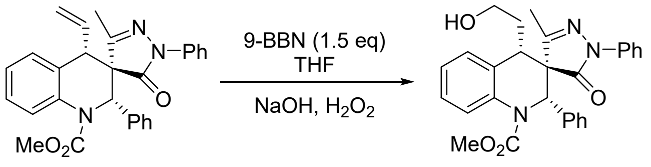

3a ee = 94% **7** 83% yield 93% ee

To a dry flask filled with nitrogen was added 3 ml of 9-BBN (0.5 M in THF) solution at 0 °C, a solution of the compound **3a** (0.2 M in THF, 0.1 mmol, 45.1 mg) was added dropwise. This solution was stirred at room temperature for 24 h. Then, quenched with 2.0 mL of 2 N aqueous NaOH solution and 0.60 mL of 30% aqueous H_2O_2 solution. After stirring for 30 min, the reaction mixture was extracted with EtOAc, and the combined organic phases were dried over MgSO4 and the solvent was removed in vacuo. The residue was purified by silica gel chromatography to afford the product **7**.

Methyl(2'*S*,4*R*,4'*S*)-4'-(2-hydroxyethyl)-3-methyl-5-oxo-1,2'-diphenyl-1,5-dihydro-2'*H*-spiro[pyrazole-4,3'-quinoline]-1'(4'*H*)-carboxylate(7).

Purified by silica gel chromatography using PE/EA = 2:1, colorless oil (39 mg, 83% yield). $[\alpha]_D$ = +59 (*c* = 0.1, CH_2Cl_2, 16.3 °C). ^1H NMR (300 MHz, $CDCl_3$) *δ* 7.86 (d, *J* = 8.0 Hz, 2H), 7.81 (d, *J* = 8.0 Hz, 1H), 7.48 – 7.39 (m, 3H), 7.31 – 7.26 (m, 2H), 7.22 – 7.15 (m, 4H), 7.04 (dd, *J* = 6.9, 2.8 Hz, 2H), 6.01 (s, 1H), 3.86 – 3.73 (m, 2H), 3.71 (s, 3H), 3.47 (dd, *J* = 9.9, 3.0 Hz, 1H), 2.10 – 1.98 (m, 2H), 1.60 – 1.49 (m, 1H), 0.88 (s, 3H) ppm; ^{13}C NMR (75 MHz, $CDCl_3$) *δ* 173.6, 160.5, 155.1, 138.8, 138.6, 137.2, 132.1, 129.0, 128.6, 127.9, 126.1, 125.8, 125.7, 125.2, 124.4, 119.2, 68.7, 65.1, 60.4, 53.5, 38.0, 29.5, 16.8, ppm. HRMS (ESI) calcd for $C_{28}H_{28}N_3O_4^+$ $[M+H]^+$: 470.2074, found 470.2078. HPLC: The product was analyzed by HPLC to determine the enantiomeric excess: 93% ee (Chiralpak AD-H, *n*-hexane / *i*-propanol = 80:20, flow rate 1.0 mL/min, λ = 254 nm); tr = 9.67 and 23.32 min.

1. X. Liang, T.-Y. Zhang, X.-Y. Zeng, Y. Zheng, K. Wei and Y.-R. Yang. *J. Am. Chem. Soc.* **2017**, *139*, 3364-3367.
2. Y.-C. Gong, Y. Wang, E.-Q. Li, H. Cui, Z. Duan. Adv. Synth. Catal. **2019**, *361*, 1389-1393.
3. S. Jia, M. Ma, E.-Q. Li, Z. Duan and F. Mathey. Org. Lett. **2021**, *23*, 3337-3342.
4. T. Zhou, T. Xia, Z. Liu, L. Liu, J. Zhang. Adv. Synth. Catal. **2018**, *360*, 4475-4479.

5. Copies of ^{1}H NMR, ^{13}C NMR, ^{31}P NMR Spectra

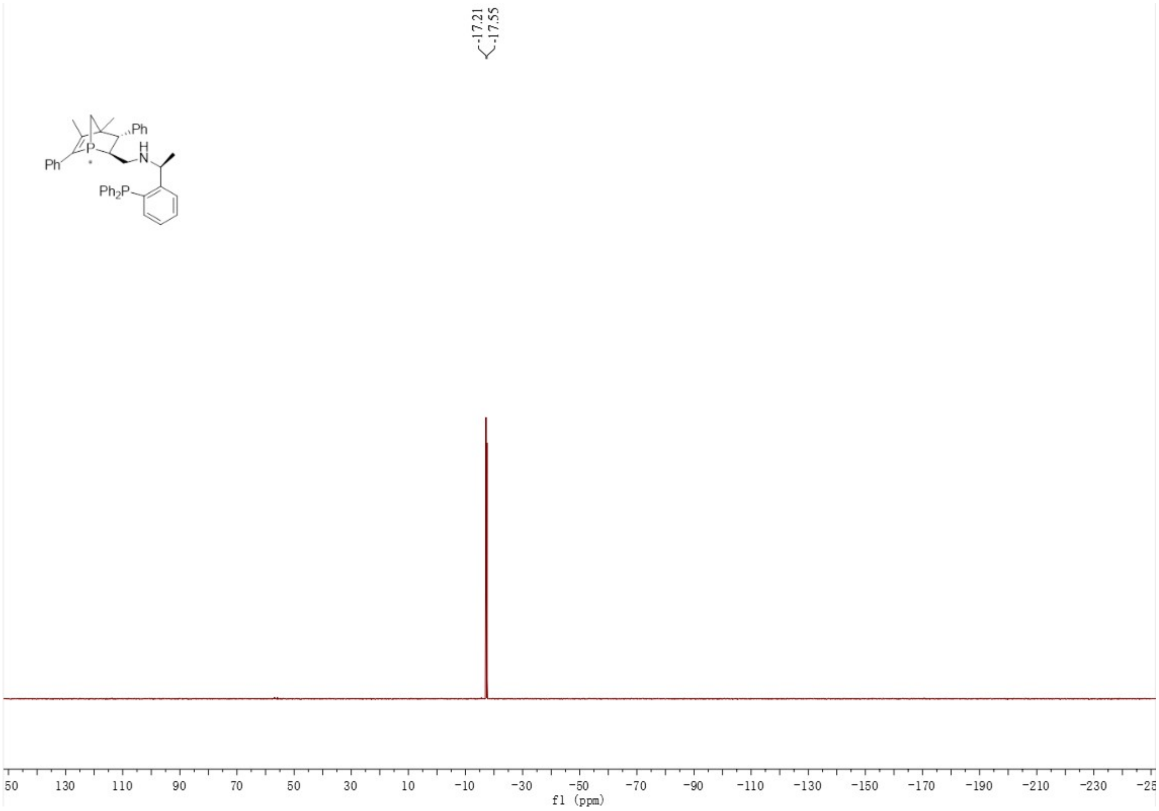

^{31}P ($CDCl_3$, 121 MHz) NMR of compound **Yue-1**

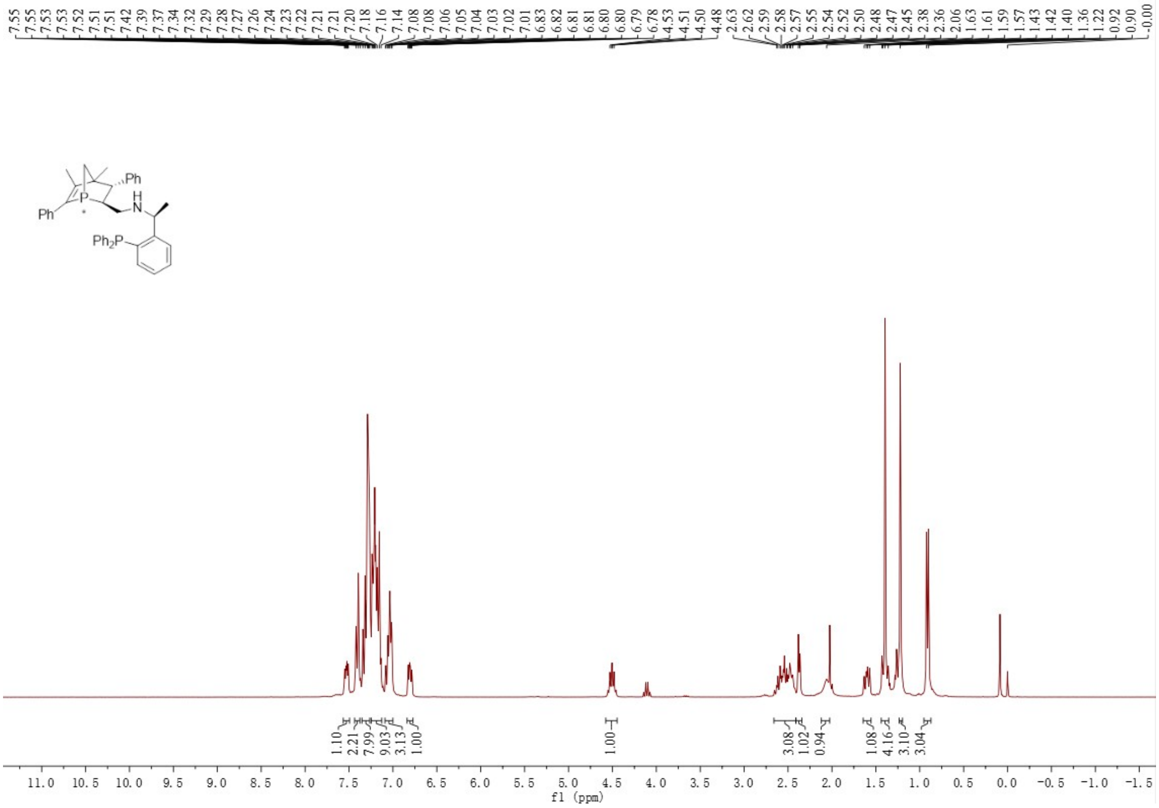

^{1}H ($CDCl_3$, 300 MHz) NMR of compound **Yue-1**

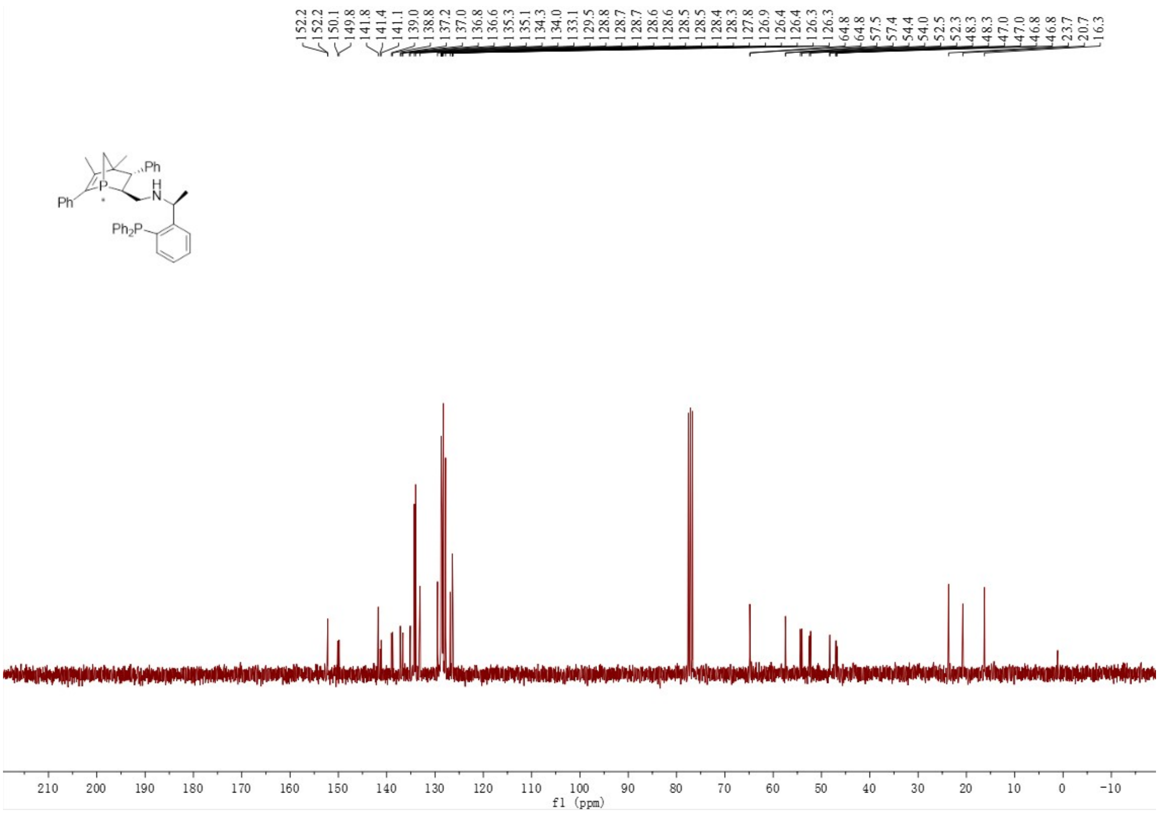

^{13}C ($CDCl_3$, 75 MHz) NMR of compound **Yue-1**

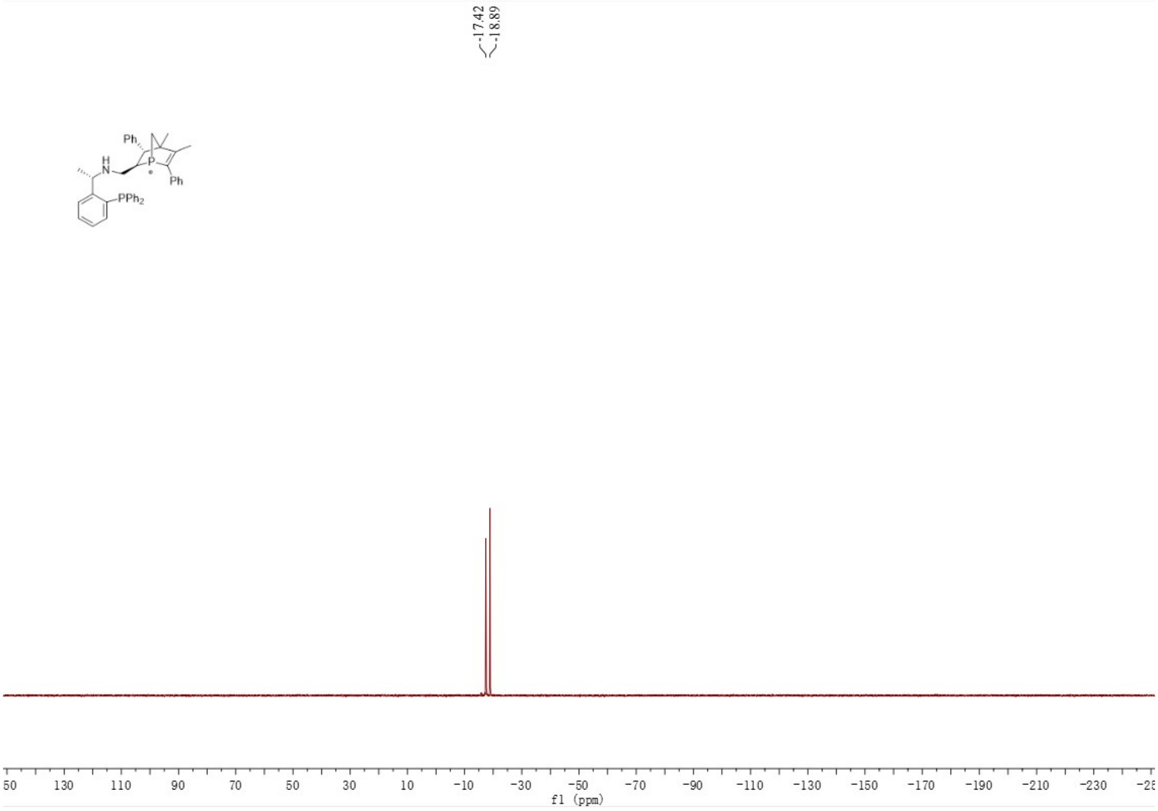

^{31}P ($CDCl_3$, 121 MHz) NMR of compound **Yue-1'**

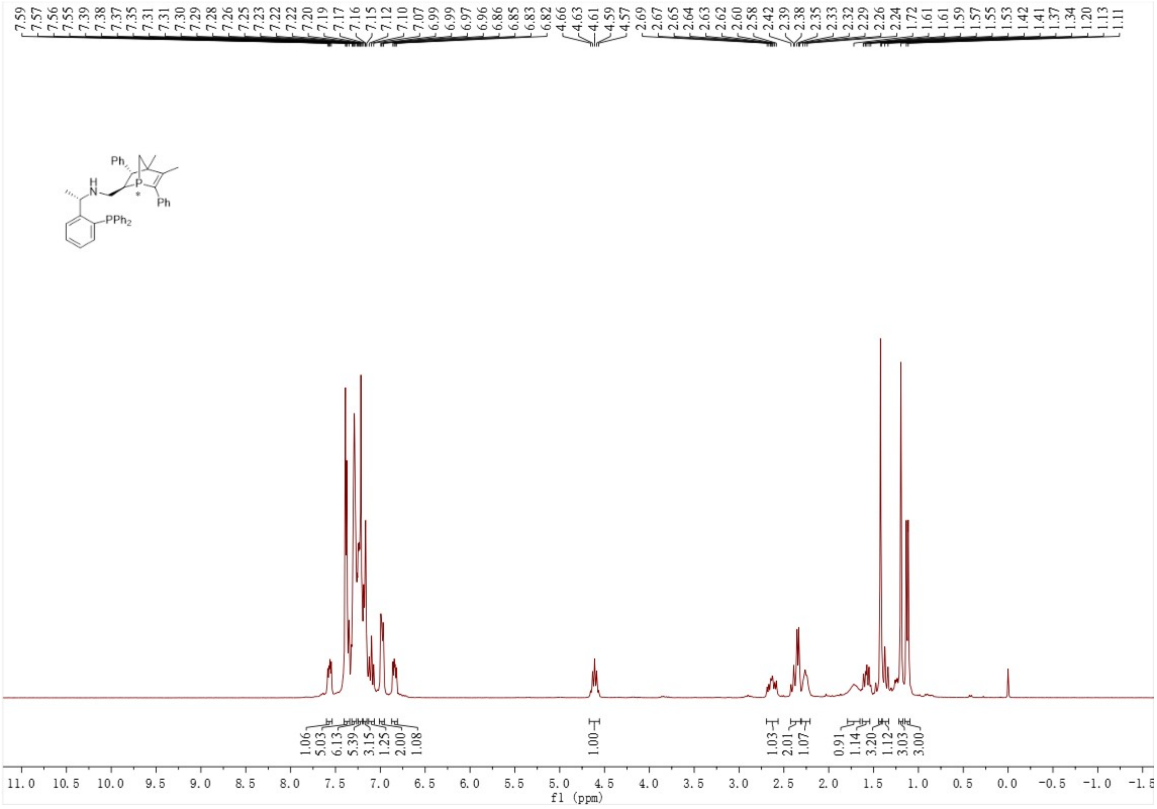

^{1}H ($CDCl_3$, 300 MHz) NMR of compound **Yue-1'**

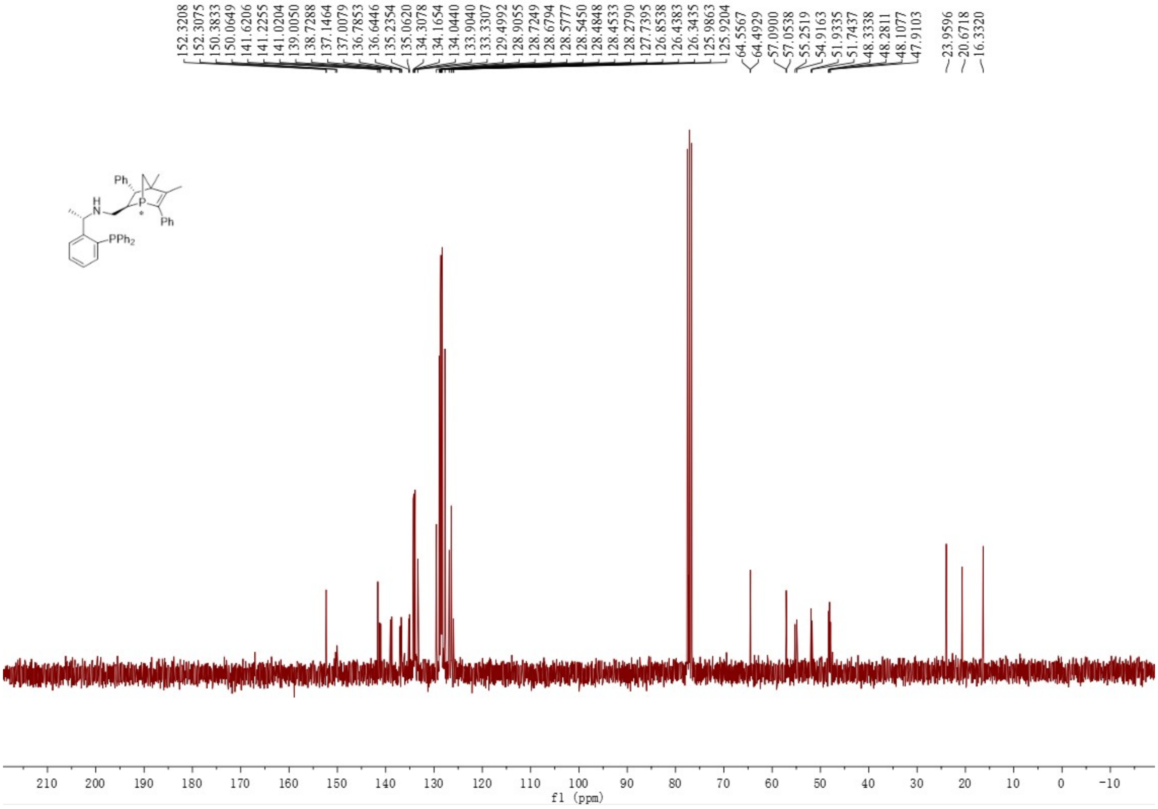

^{13}C ($CDCl_3$, 75 MHz) NMR of compound **Yue-1'**

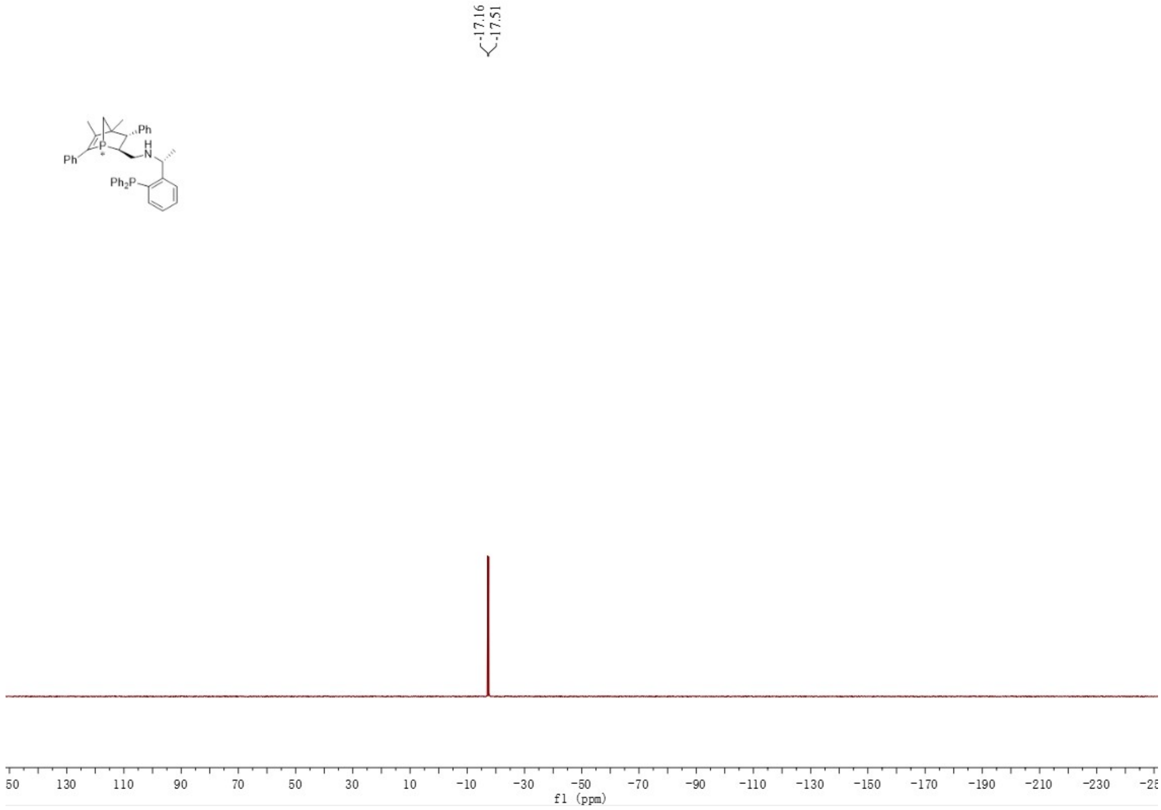

^{31}P ($CDCl_3$, 121 MHz) NMR of compound **Yue-2**

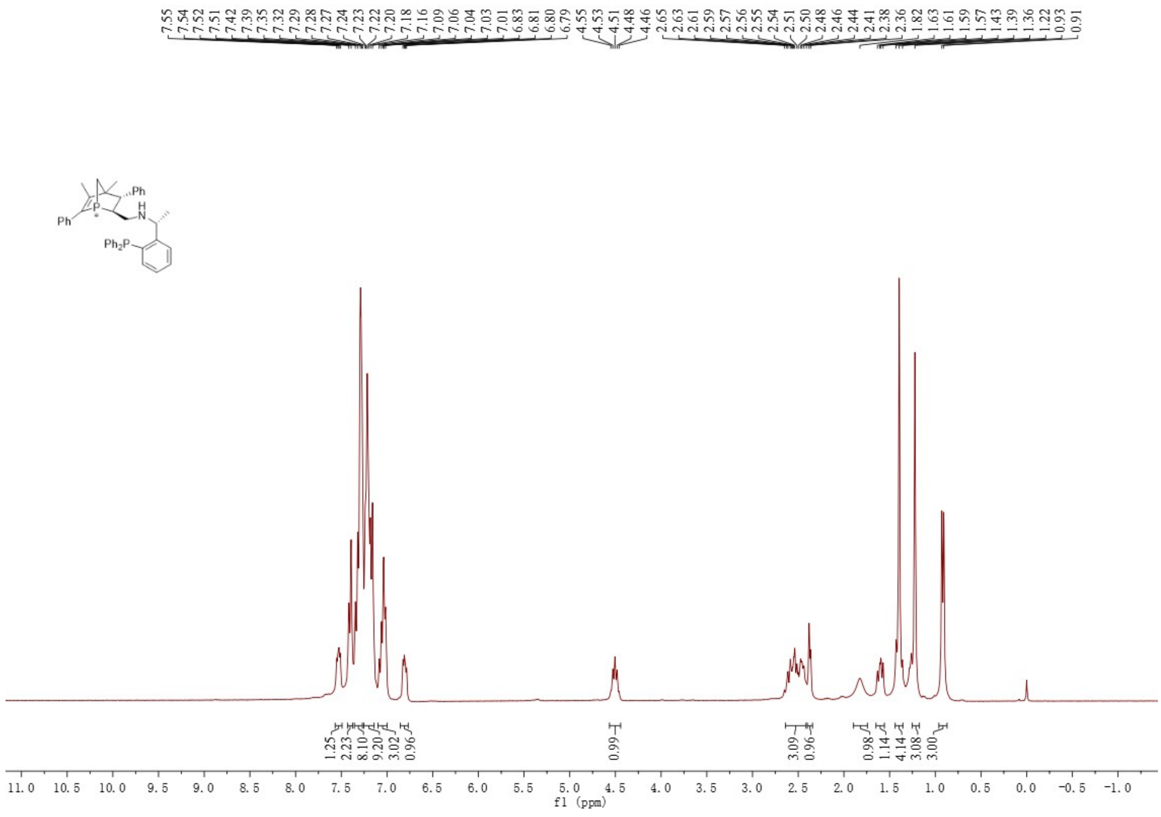

^{1}H ($CDCl_3$, 300 MHz) NMR of compound **Yue-2**

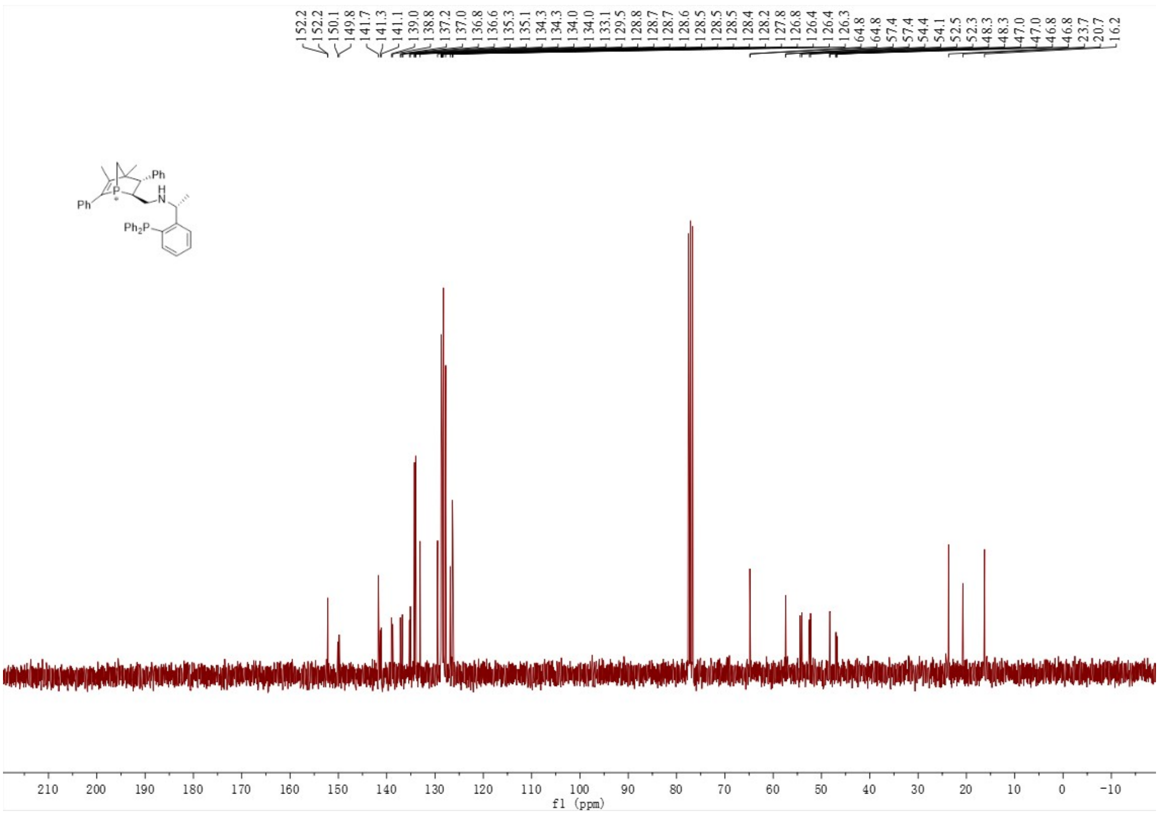

^{13}C ($CDCl_3$, 75 MHz) NMR of compound **Yue-2**

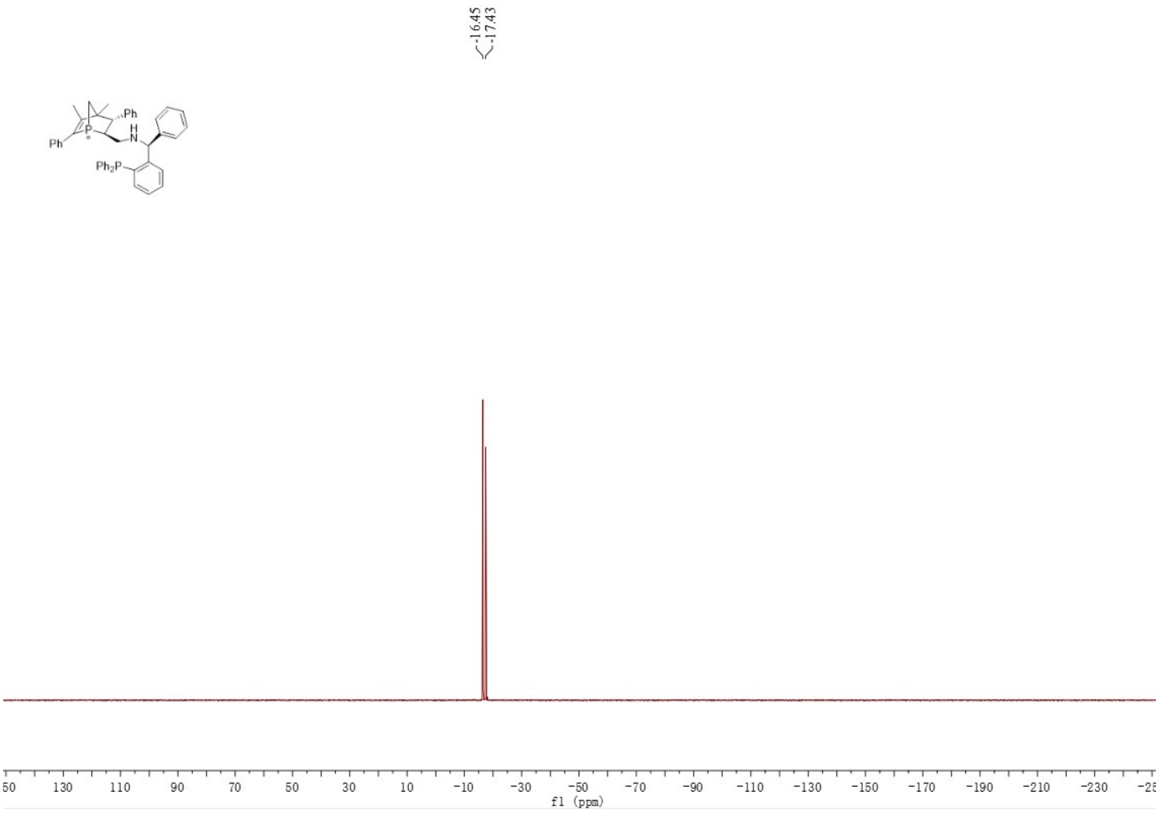

^{31}P ($CDCl_3$, 121 MHz) NMR of compound **Yue-3**

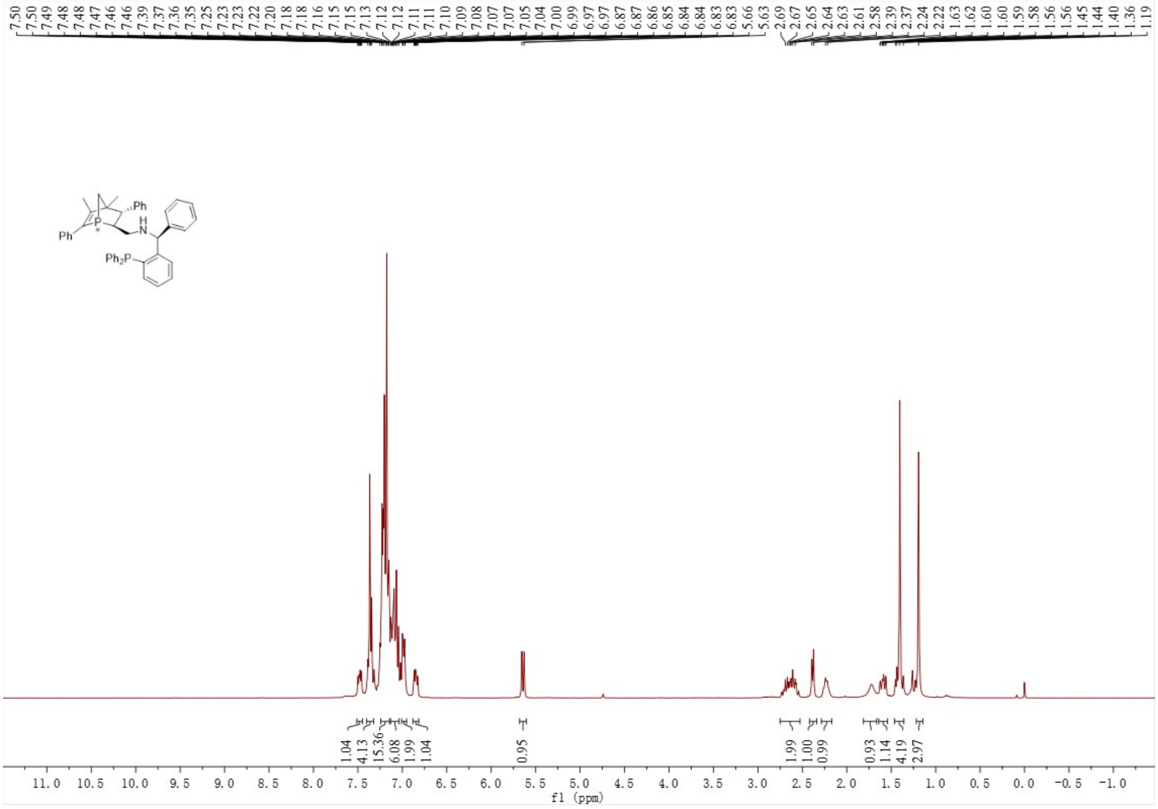

^{1}H ($CDCl_3$, 300 MHz) NMR of compound **Yue-3**

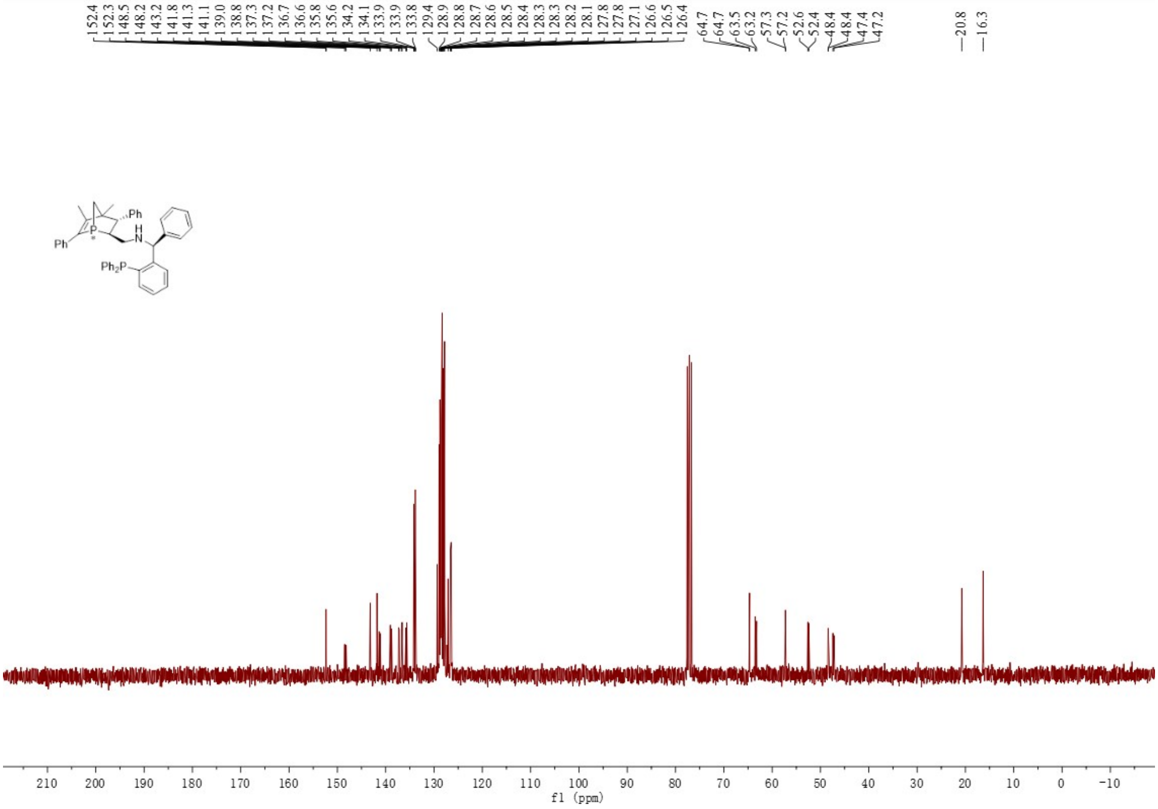

^{13}C ($CDCl_3$, 75 MHz) NMR of compound **Yue-3**

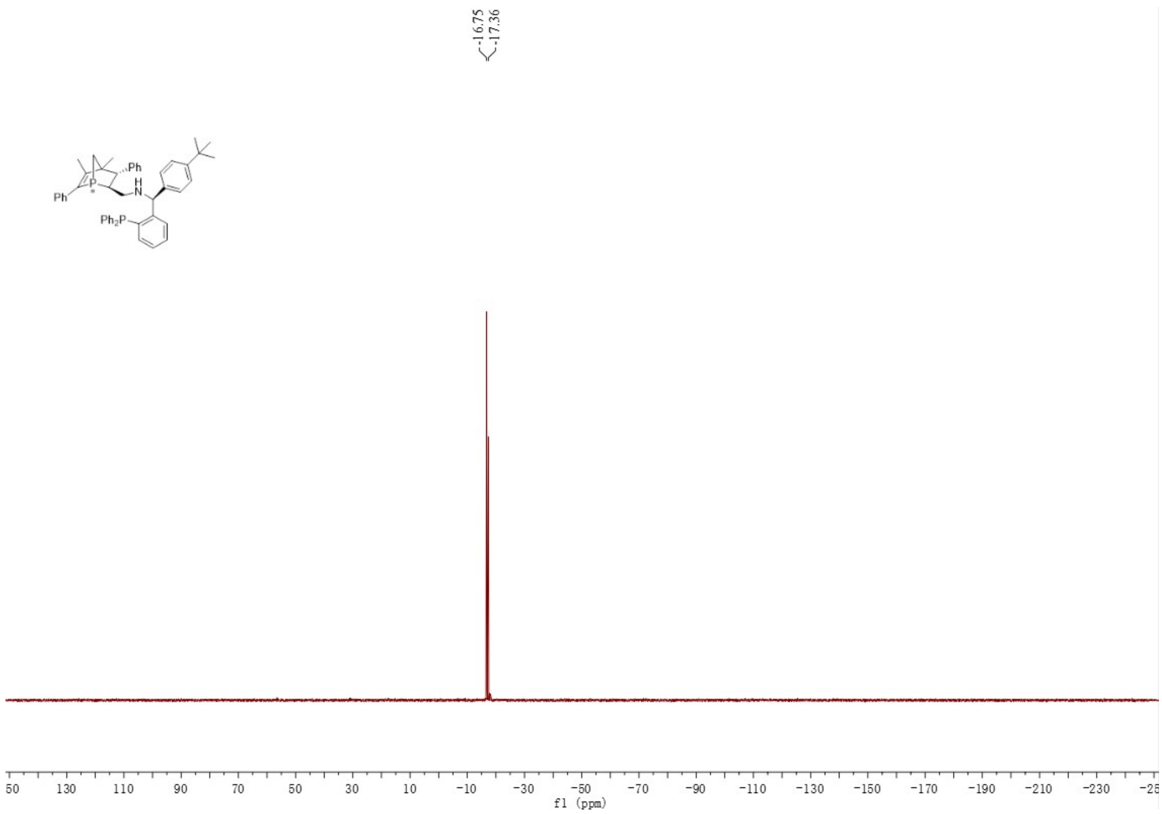

^{31}P ($CDCl_3$, 121 MHz) NMR of compound **Yue-4**

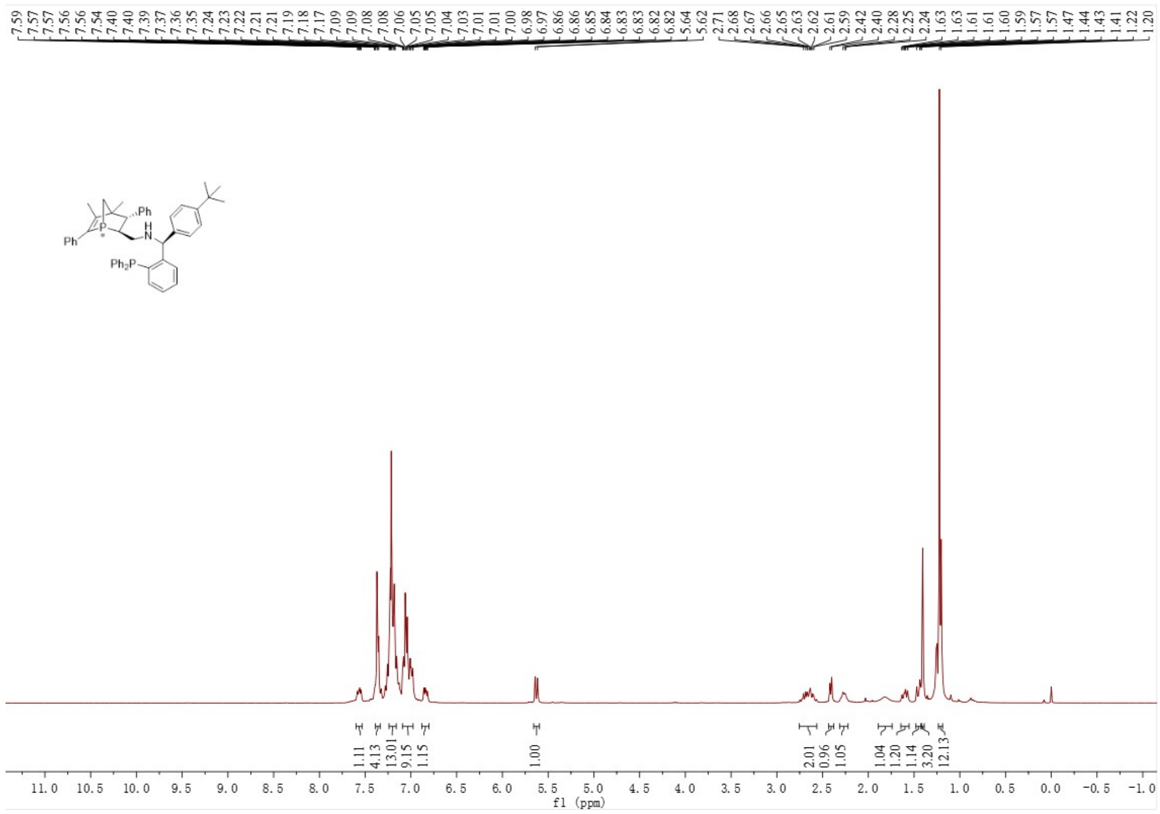

^{1}H ($CDCl_3$, 300 MHz) NMR of compound **Yue-4**

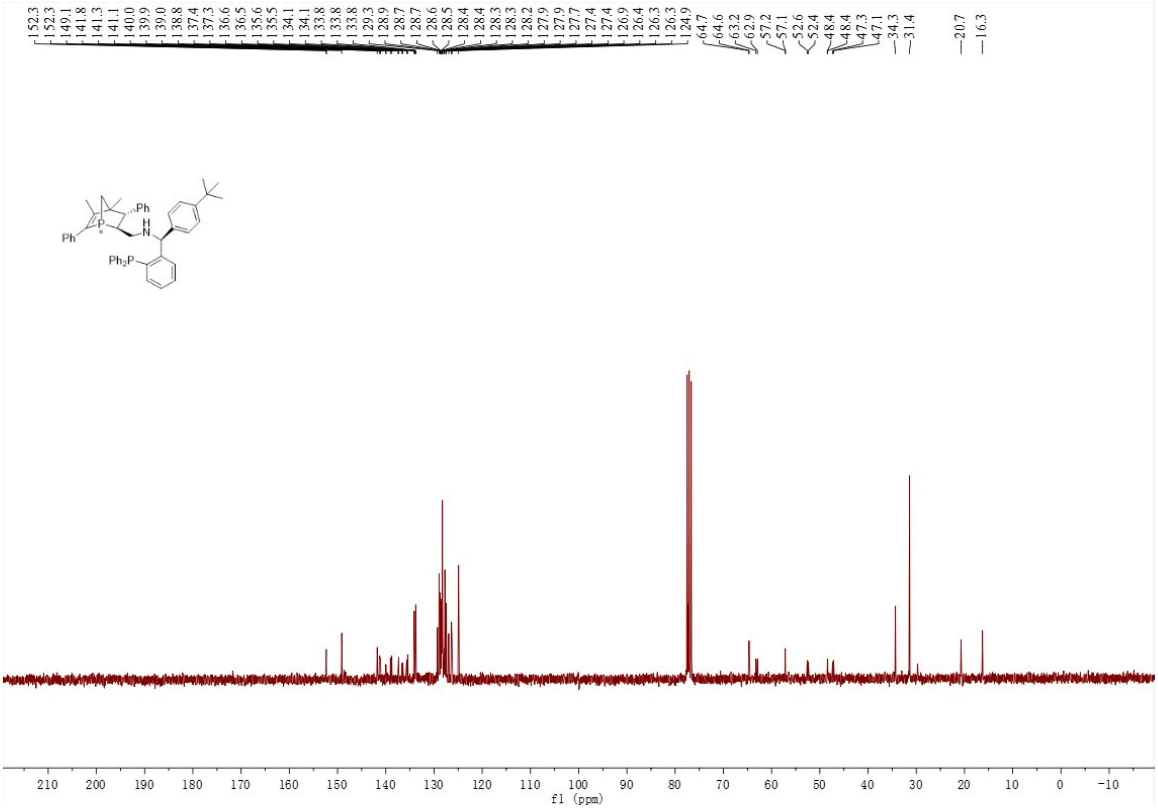

^{13}C ($CDCl_3$, 75 MHz) NMR of compound **Yue-4**

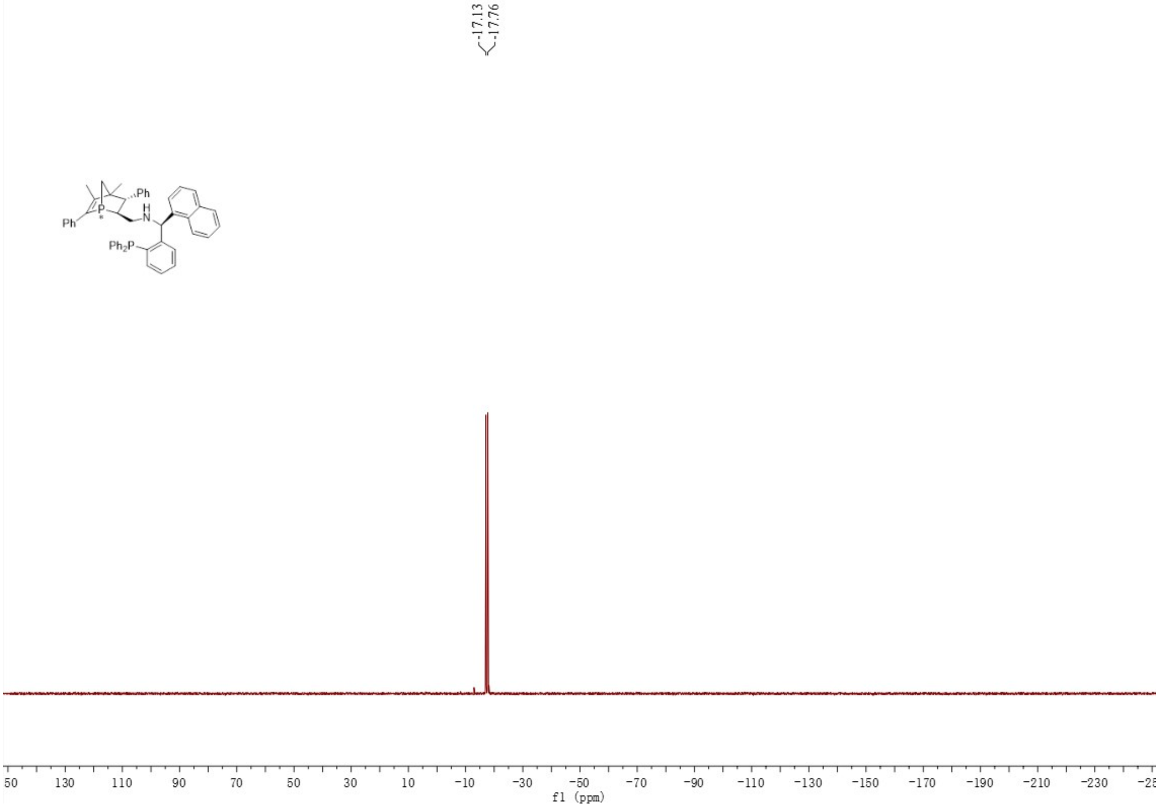

^{31}P ($CDCl_3$, 121 MHz) NMR of compound **Yue-5**

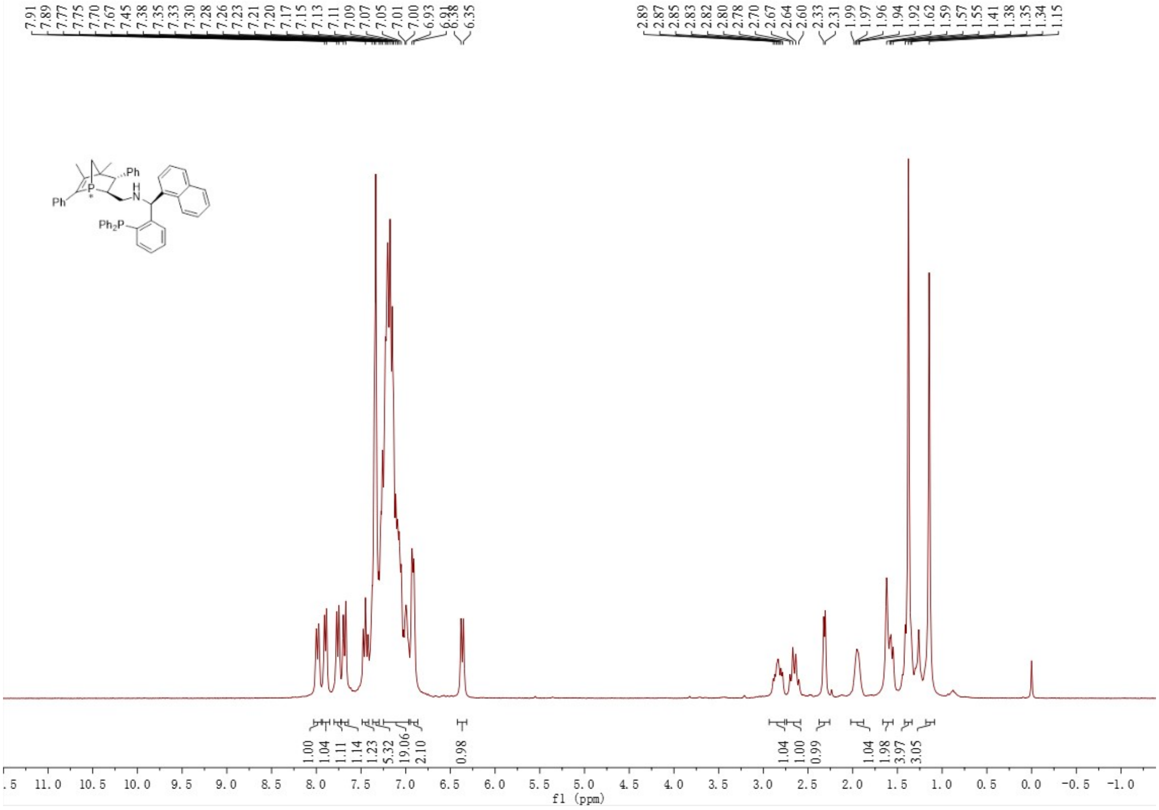

^{1}H ($CDCl_3$, 300 MHz) NMR of compound **Yue-5**

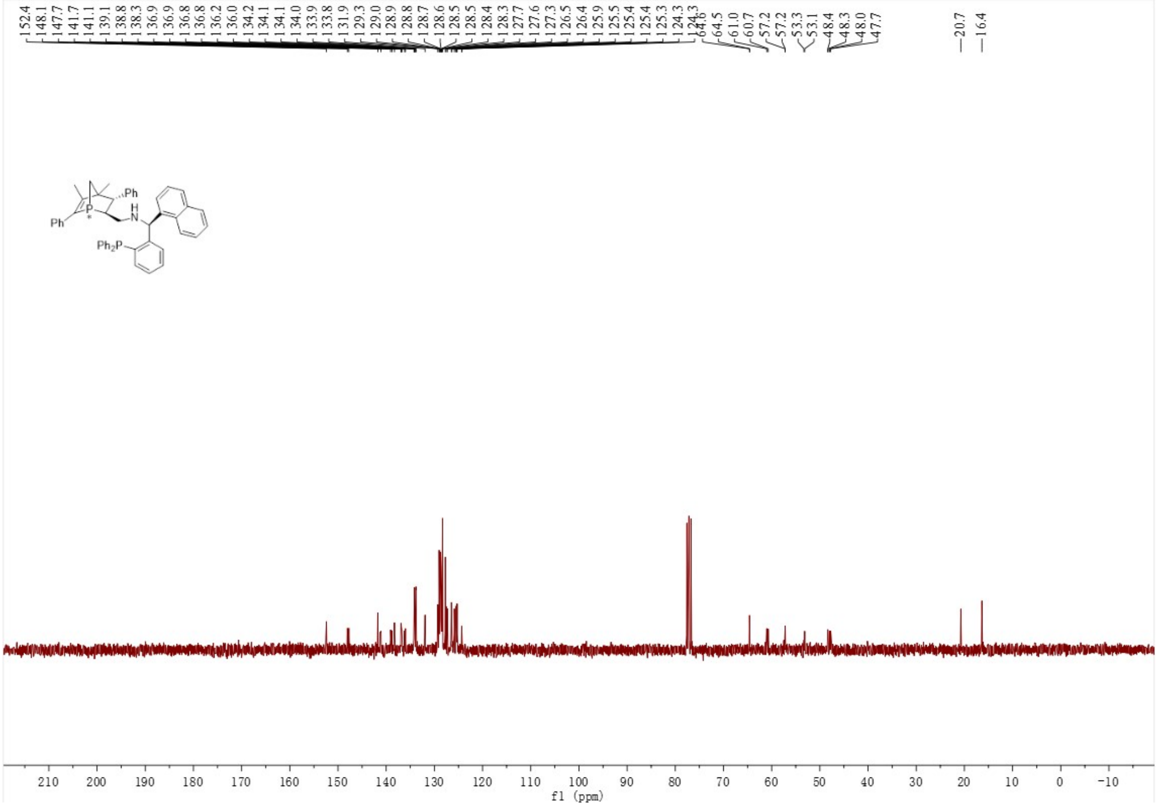

^{13}C ($CDCl_3$, 75 MHz) NMR of compound **Yue-5**

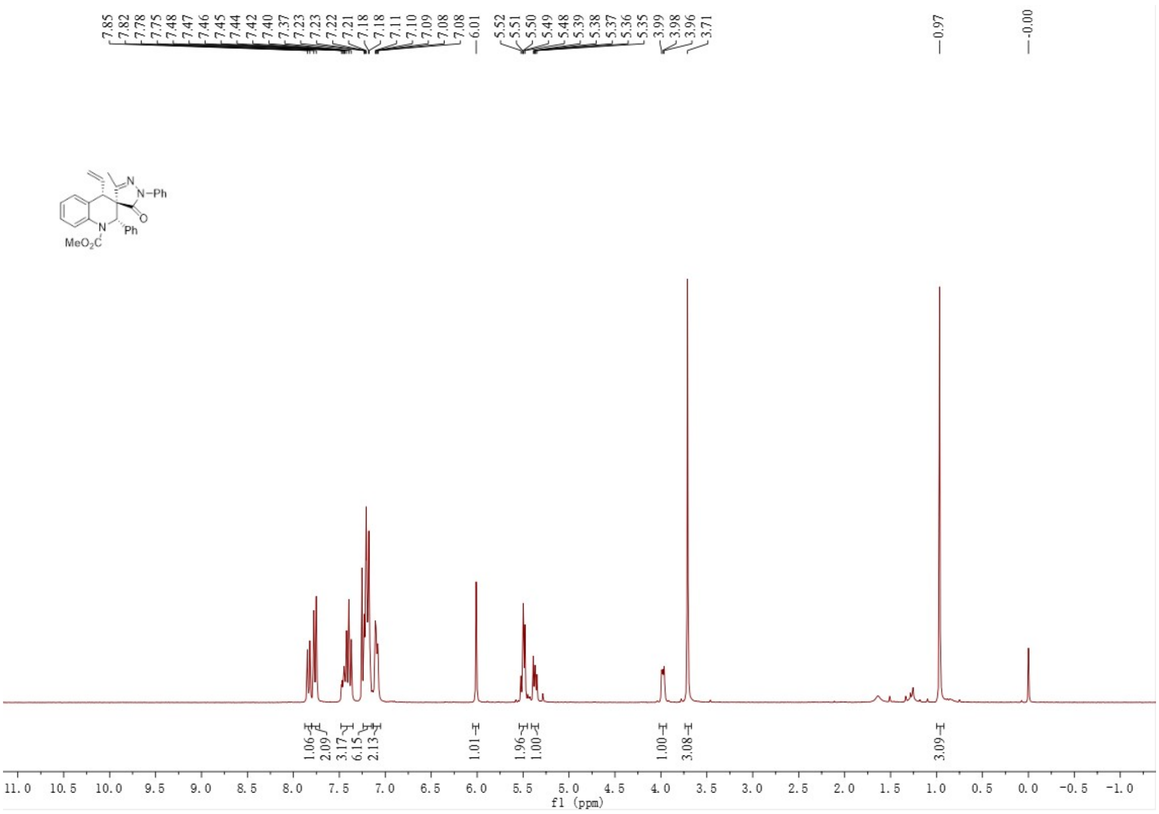

^{1}H ($CDCl_3$, 300 MHz) NMR of compound **3a**

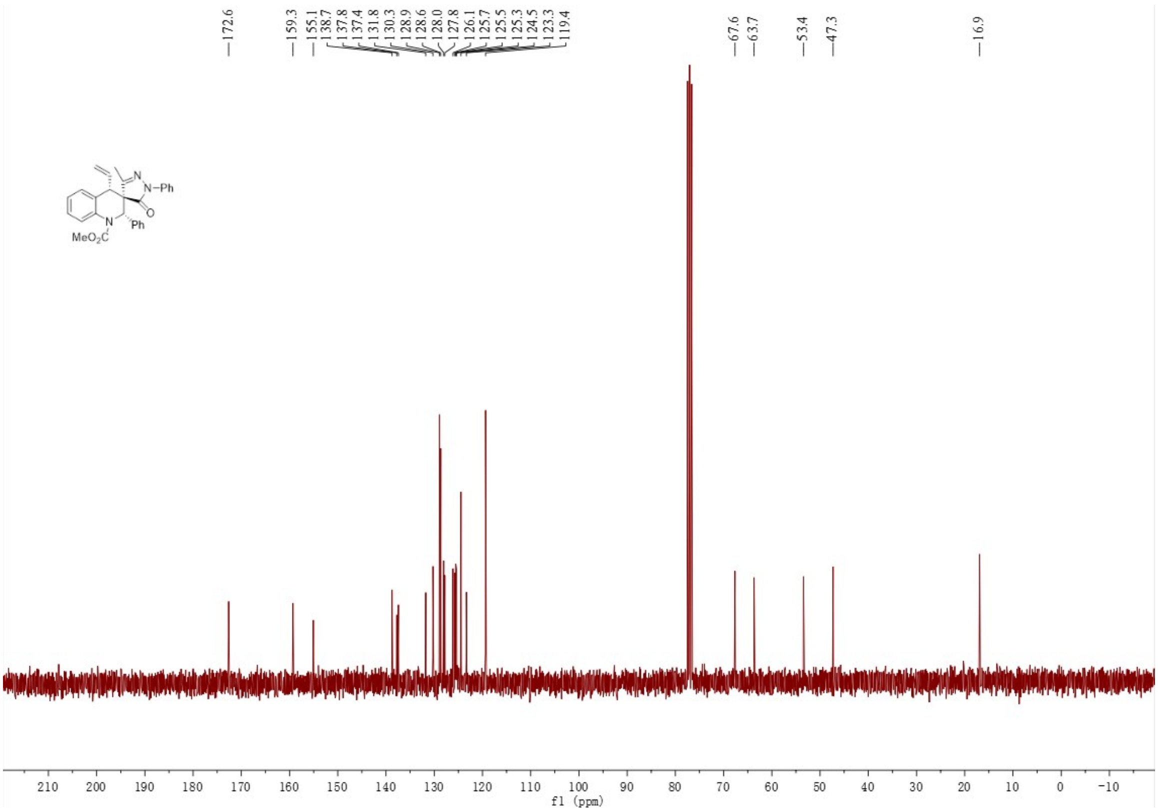

^{13}C ($CDCl_3$, 75 MHz) NMR of compound **3a**

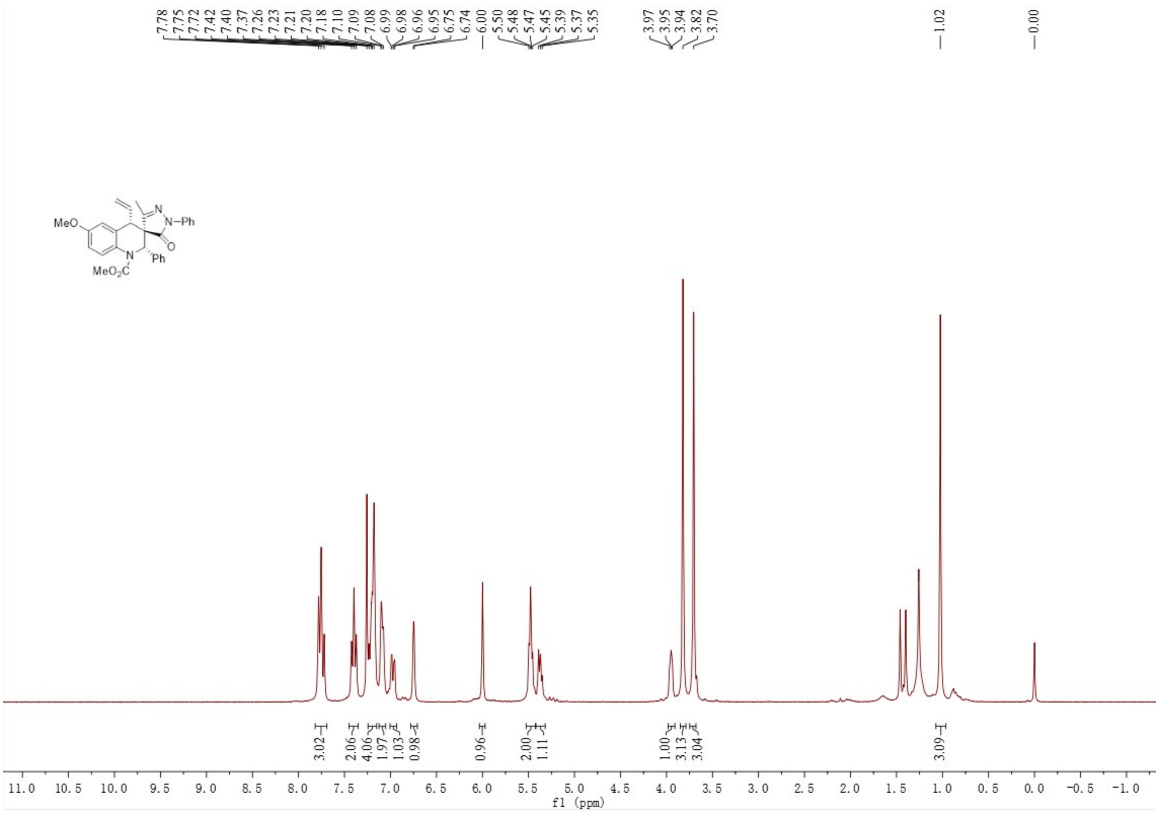

^{1}H ($CDCl_3$, 300 MHz) NMR of compound **3b**

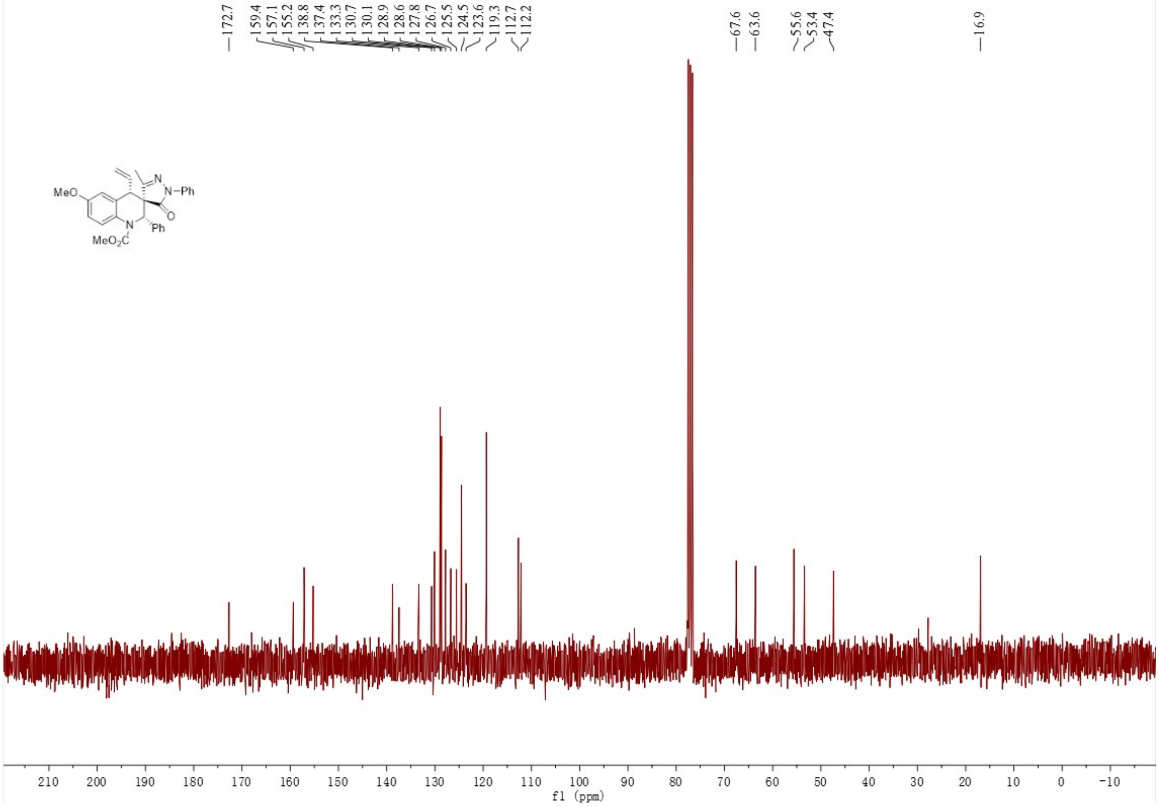

^{13}C ($CDCl_3$, 75 MHz) NMR of compound **3b**

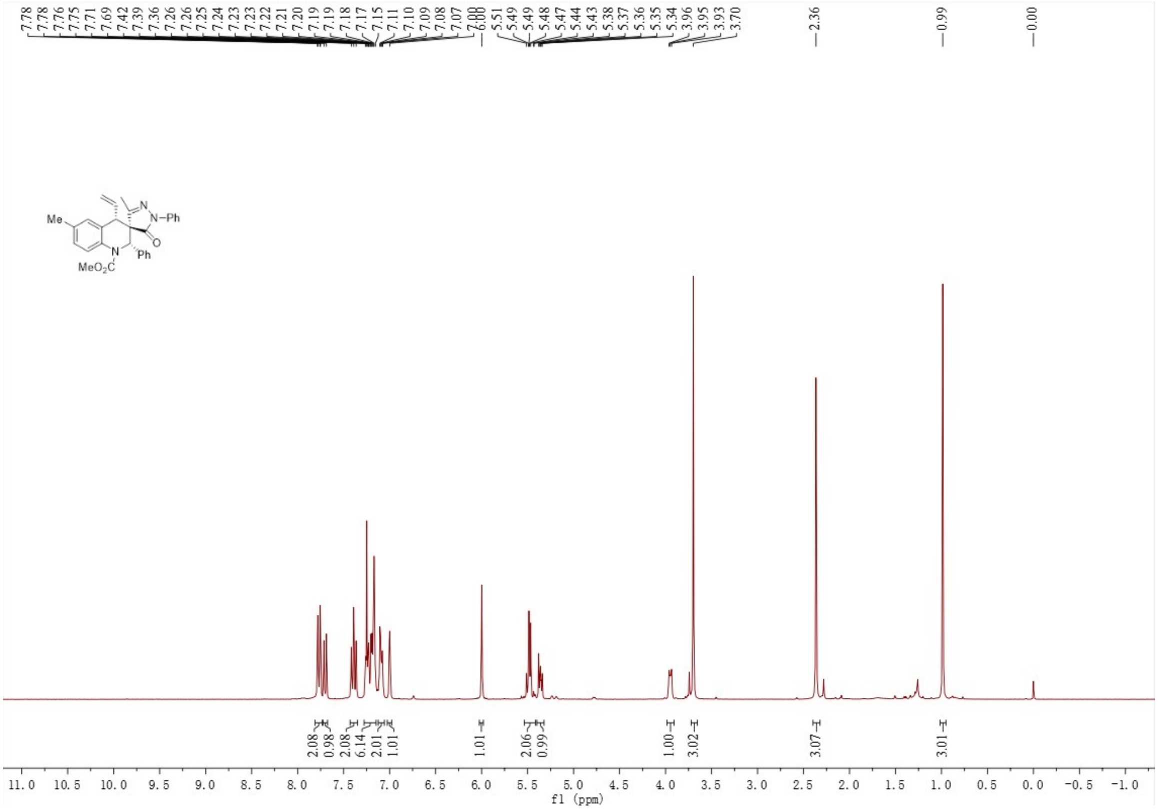

^{1}H ($CDCl_3$, 300 MHz) NMR of compound **3c**

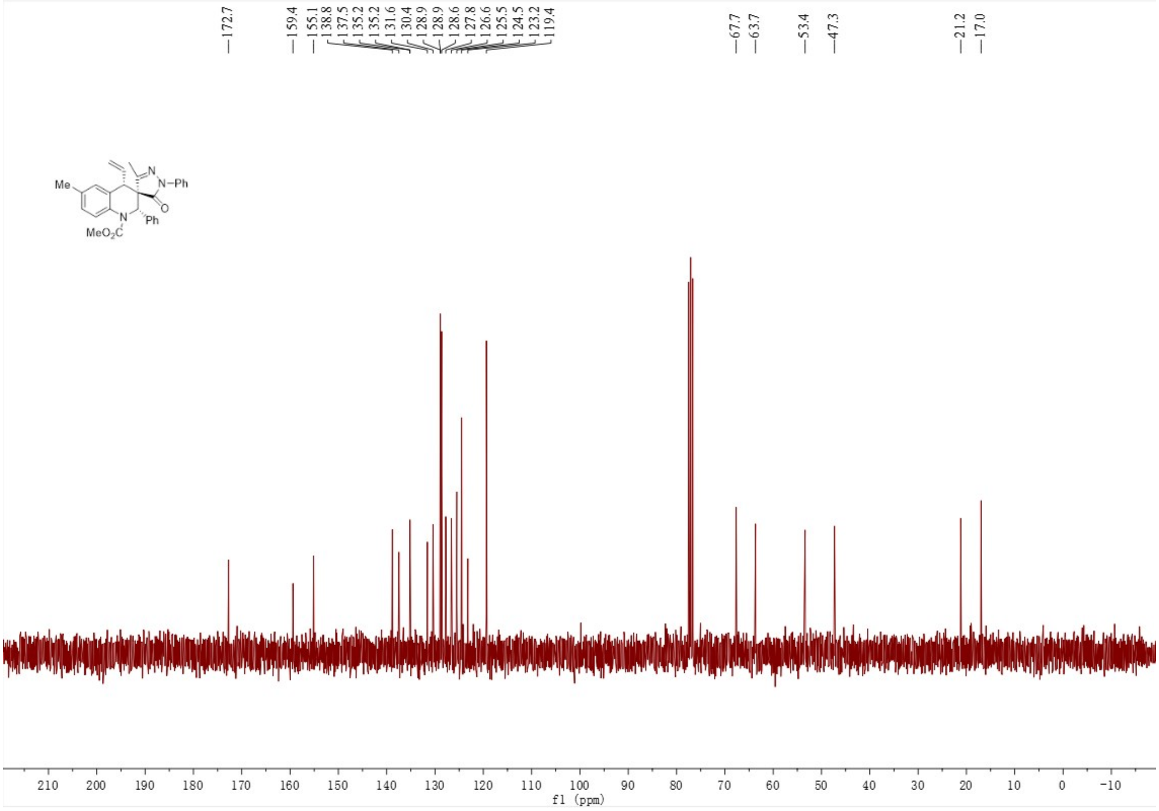

^{13}C ($CDCl_3$, 75 MHz) NMR of compound **3c**

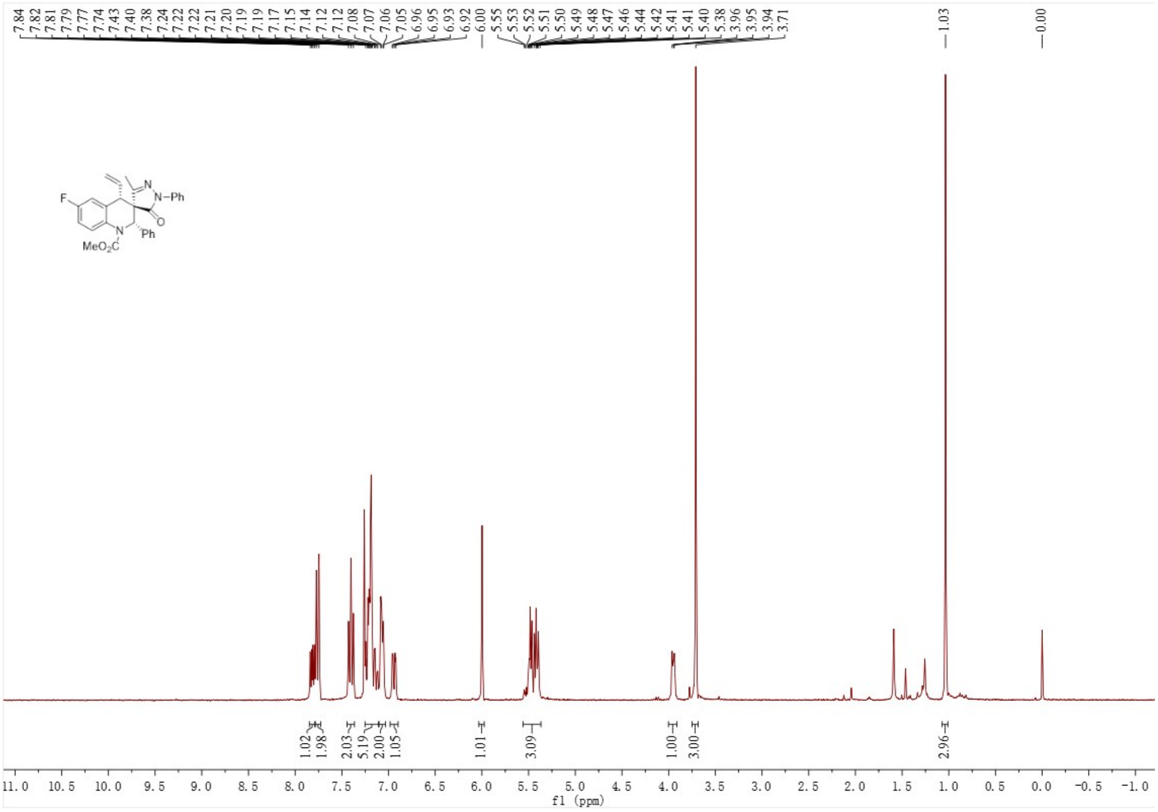

^{1}H ($CDCl_3$, 300 MHz) NMR of compound **3d**

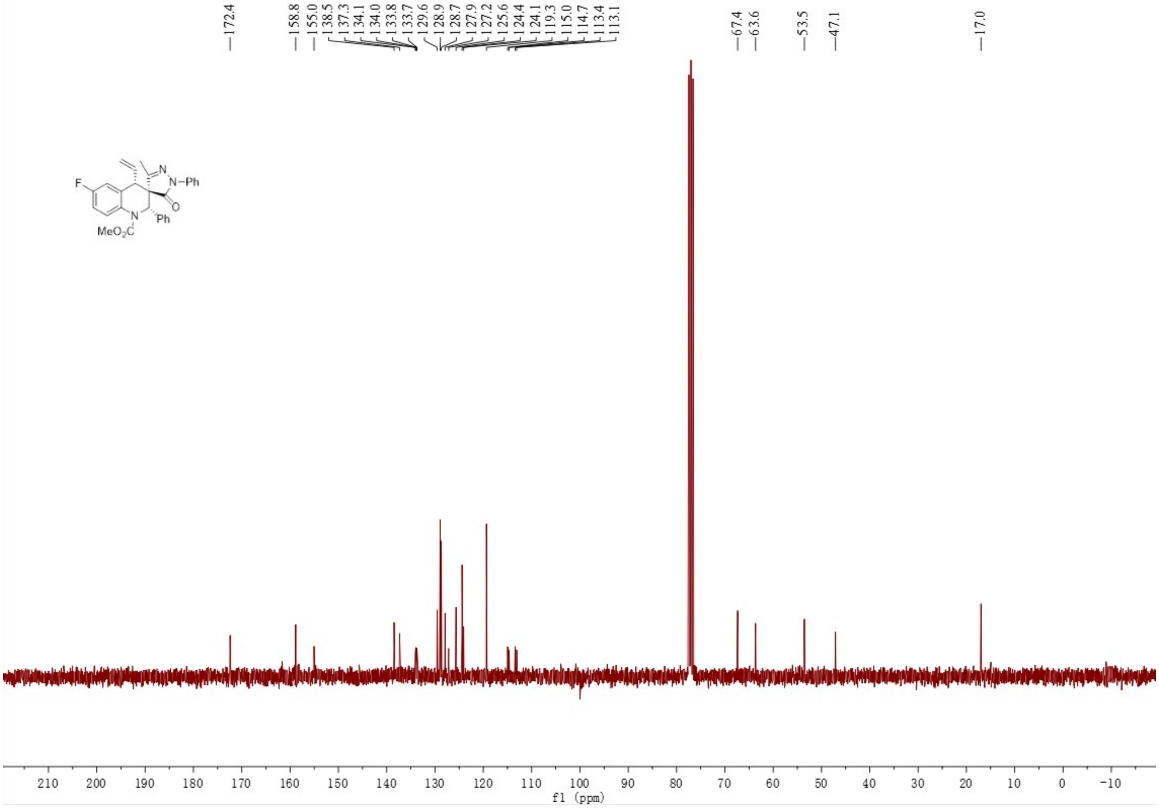

^{13}C ($CDCl_3$, 75 MHz) NMR of compound **3d**

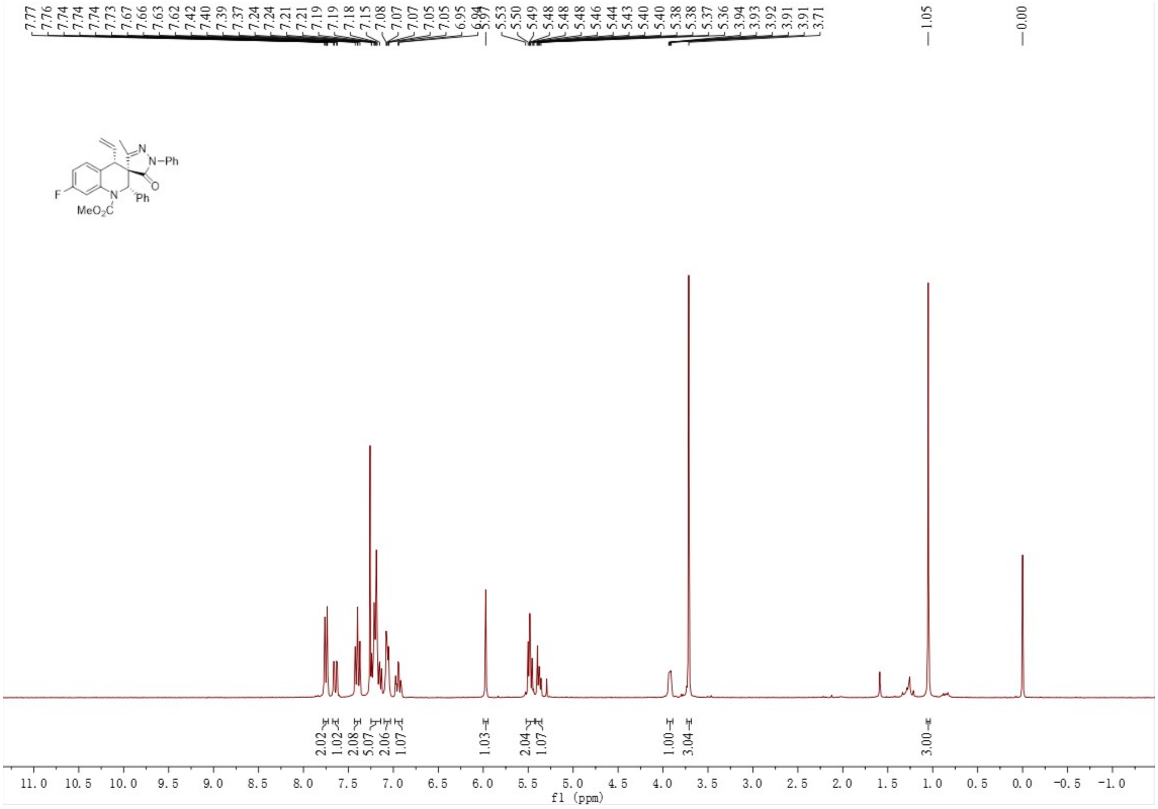

^{1}H (CDCl$_3$, 300 MHz) NMR of compound **3e**

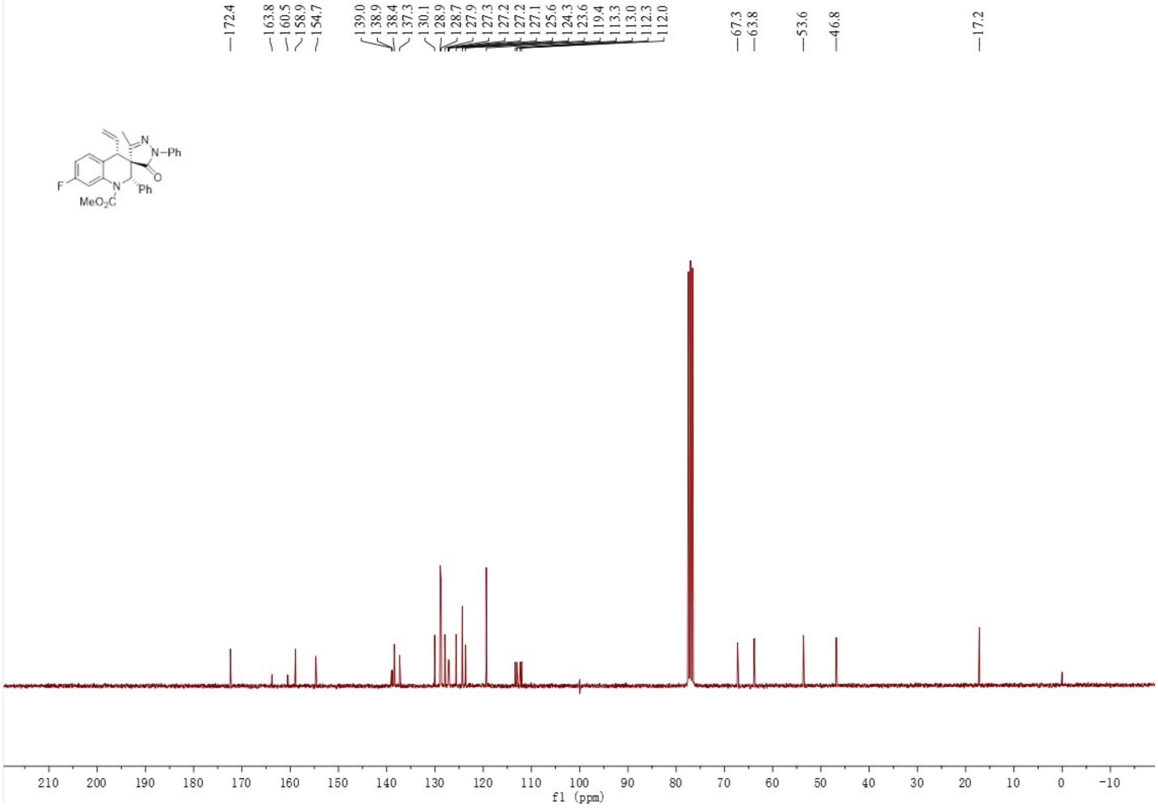

^{13}C (CDCl$_3$, 75 MHz) NMR of compound **3e**

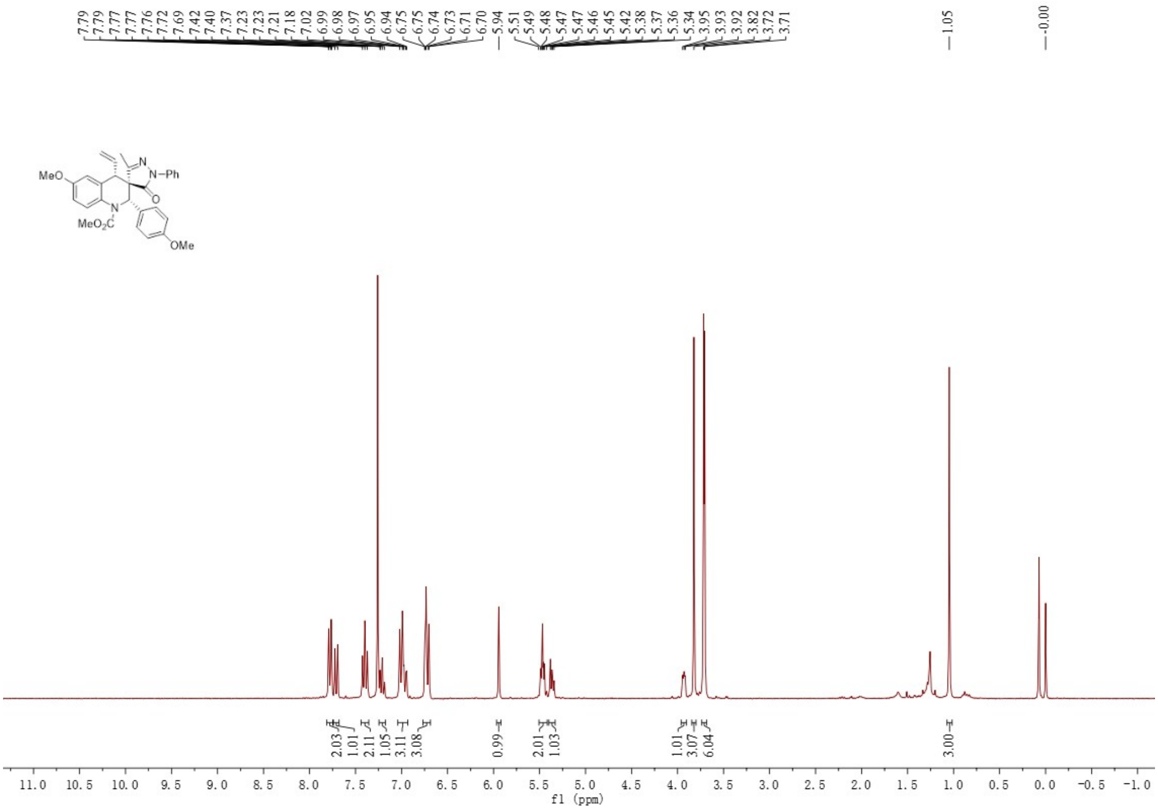

^{1}H ($CDCl_3$, 300 MHz) NMR of compound **3f**

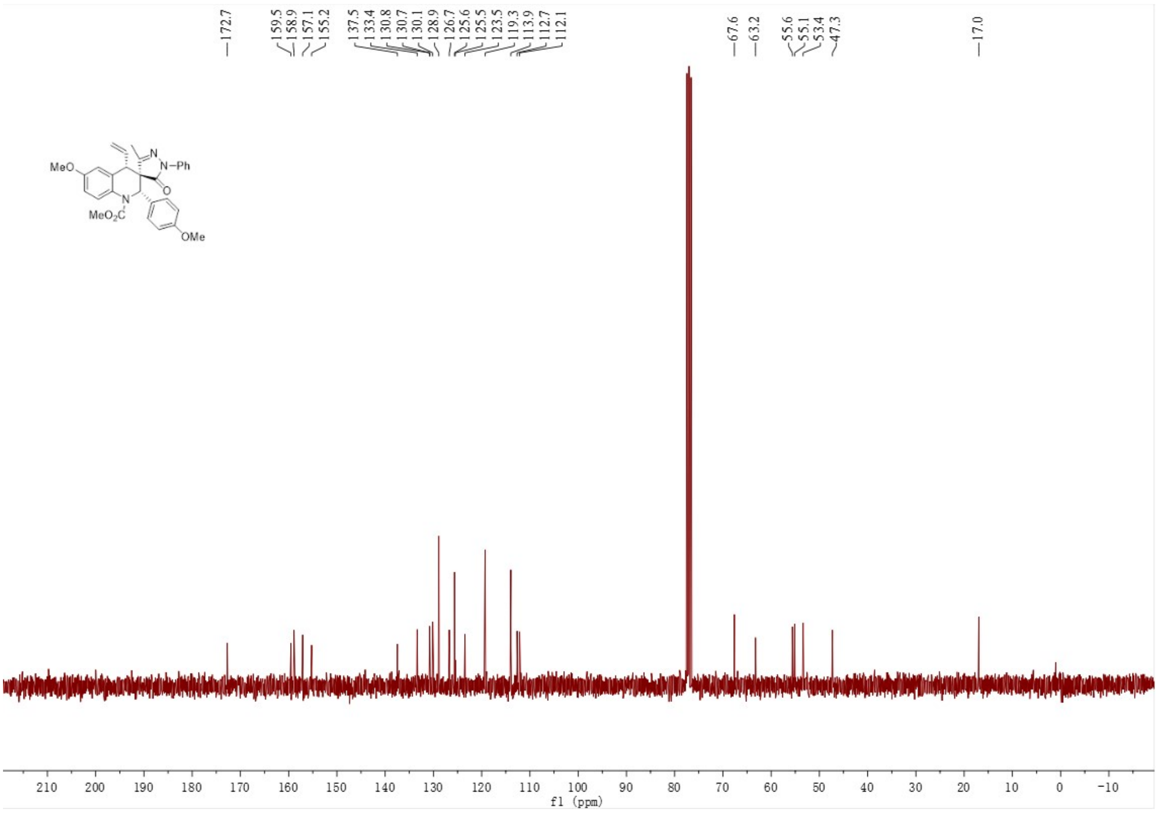

^{13}C ($CDCl_3$, 75 MHz) NMR of compound **3f**

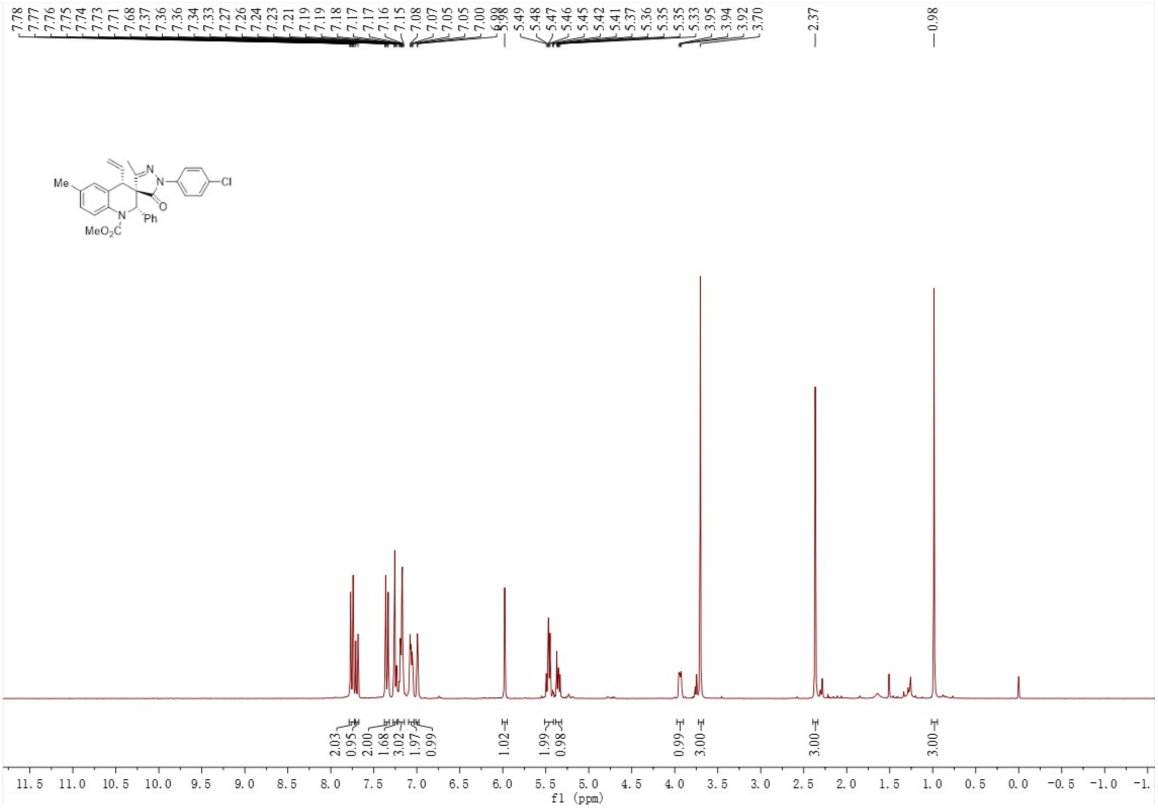

^{1}H (CDCl$_3$, 300 MHz) NMR of compound **3g**

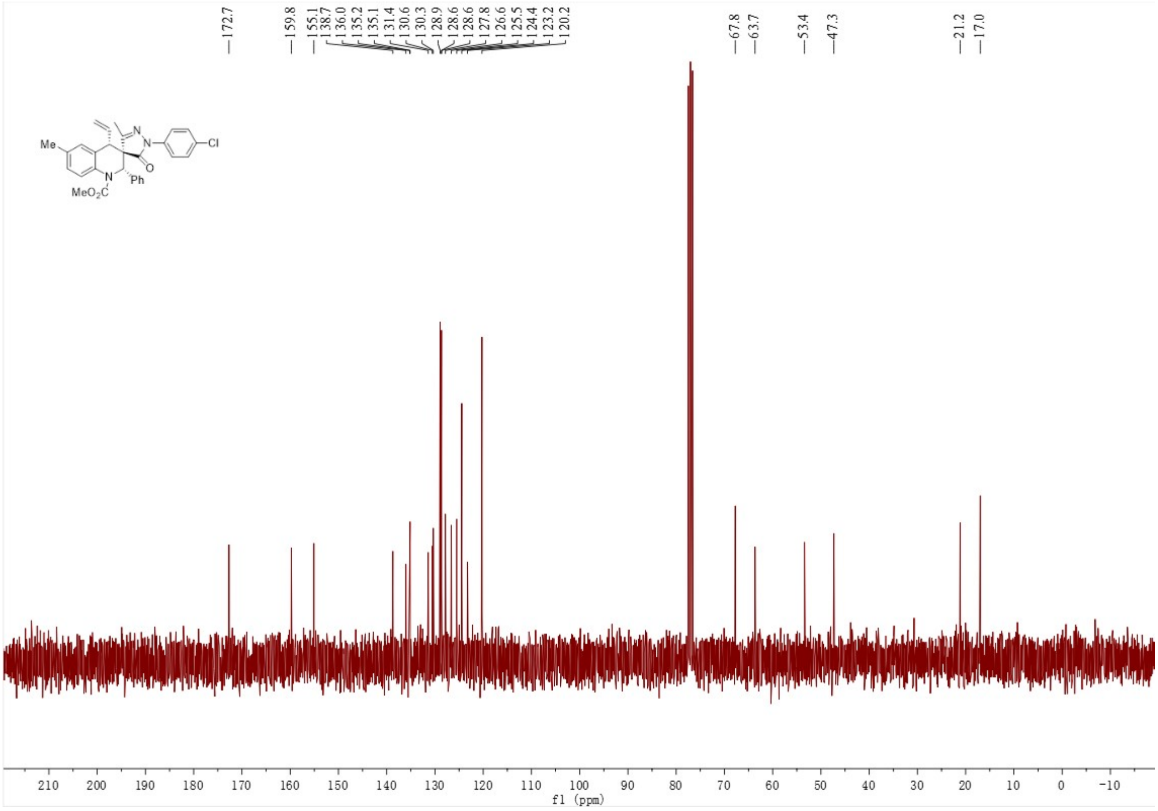

^{13}C (CDCl$_3$, 75 MHz) NMR of compound **3g**

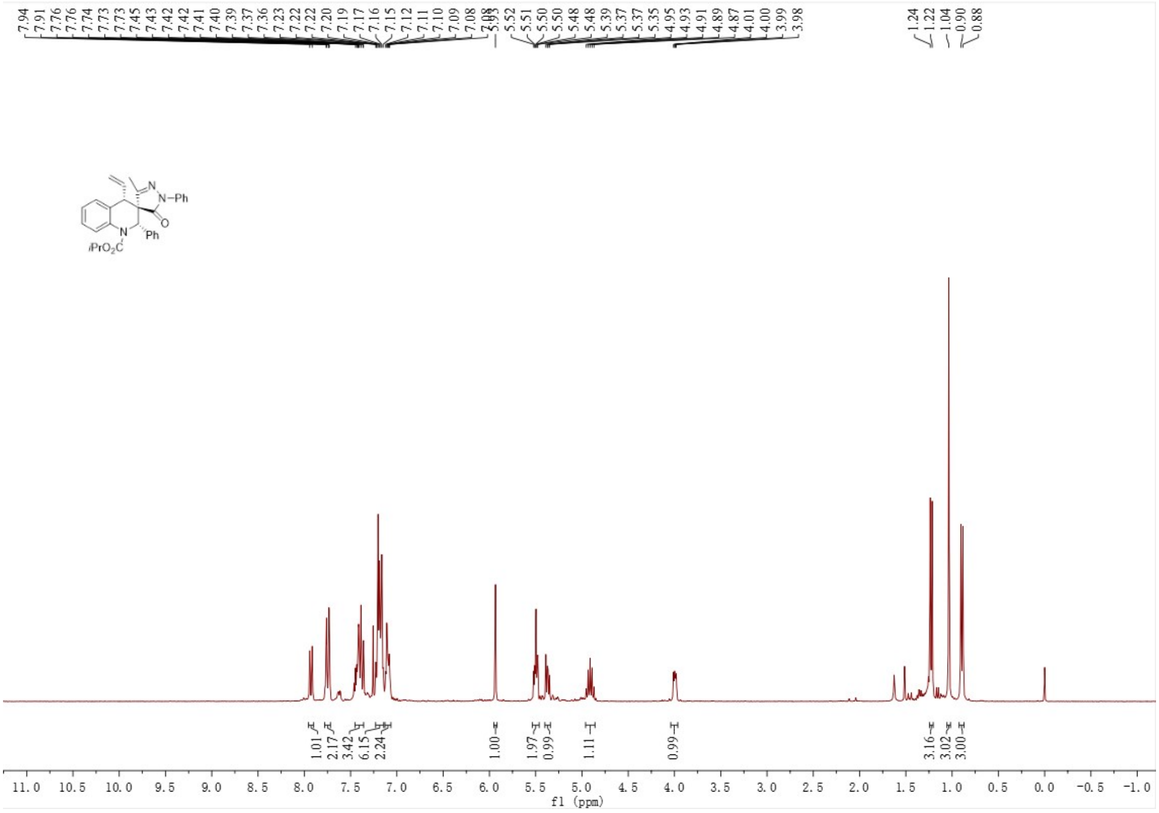

^{1}H ($CDCl_3$, 300 MHz) NMR of compound **3h**

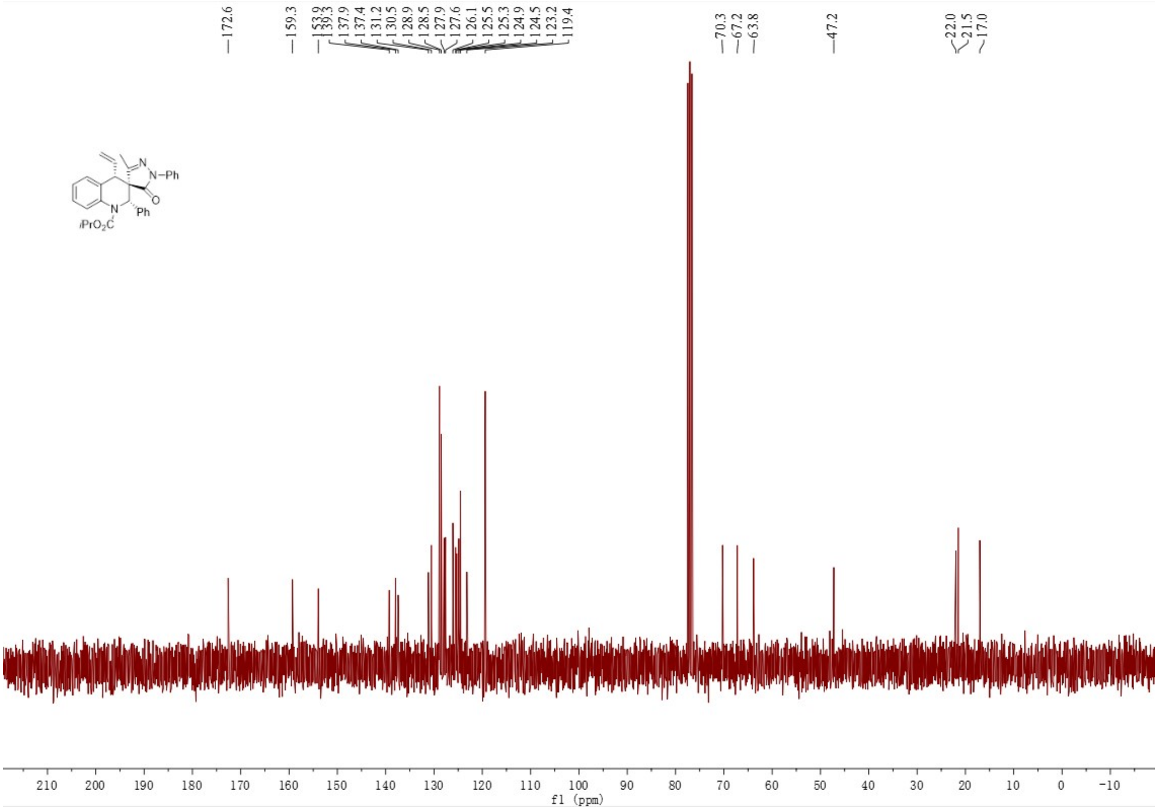

^{13}C ($CDCl_3$, 75 MHz) NMR of compound **3h**

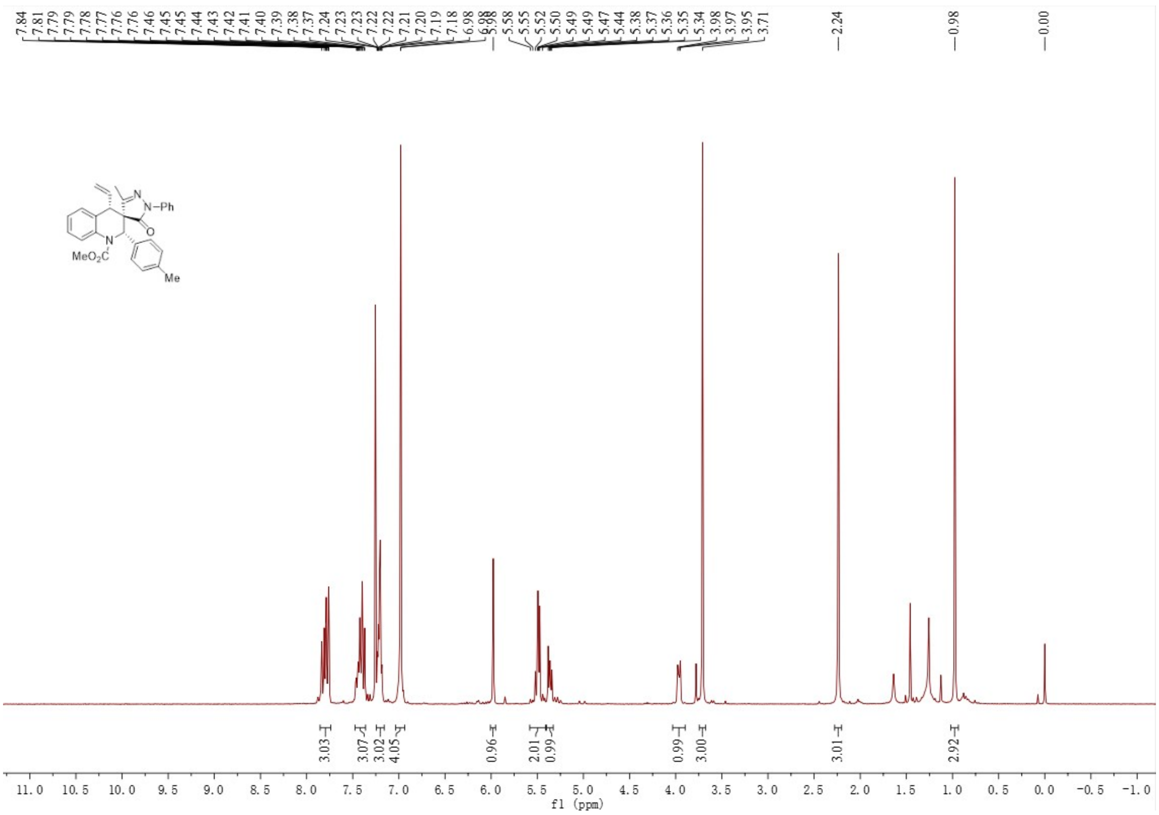

^{1}H ($CDCl_3$, 300 MHz) NMR of compound **3i**

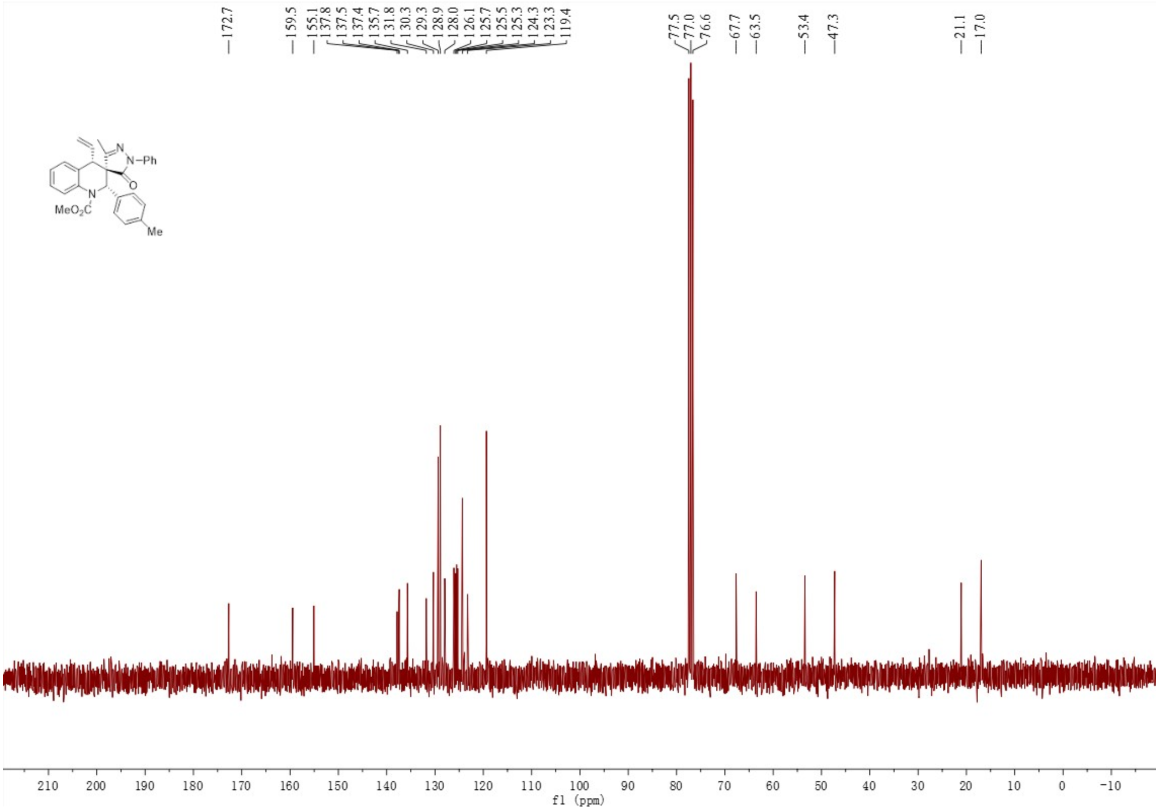

^{13}C ($CDCl_3$, 75 MHz) NMR of compound **3i**

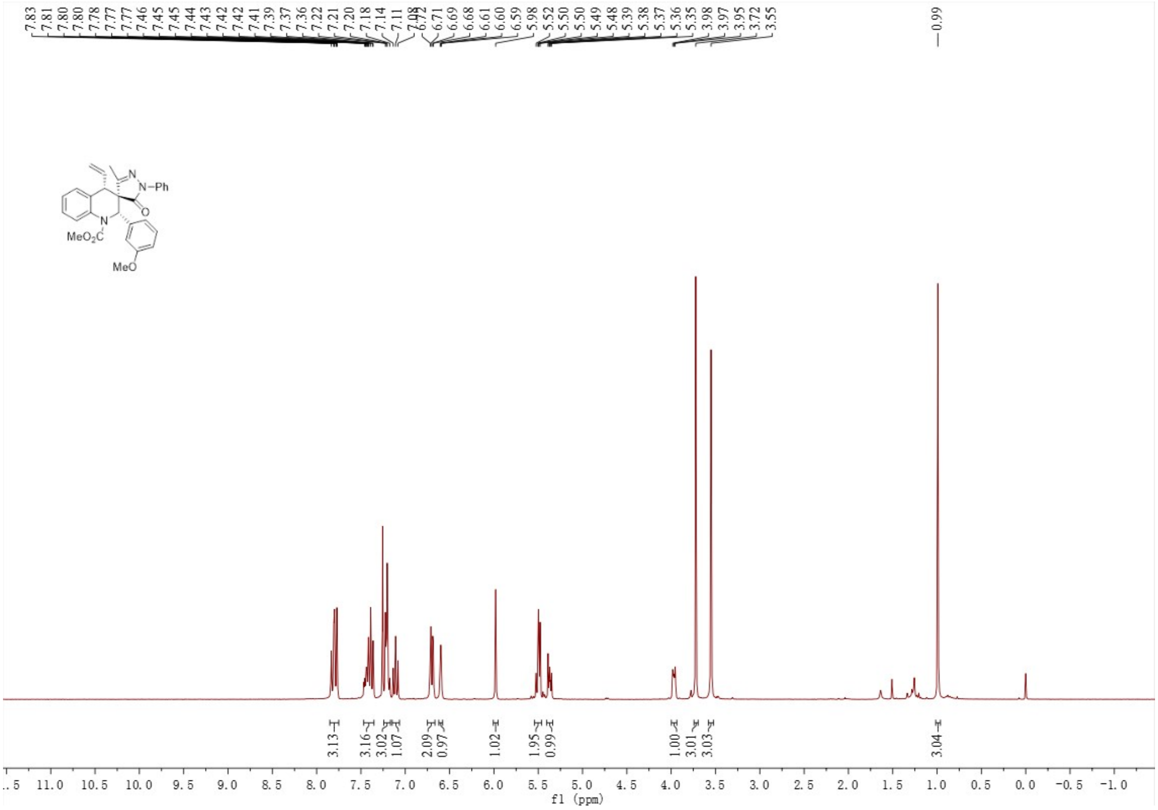

^{1}H ($CDCl_3$, 300 MHz) NMR of compound **3j**

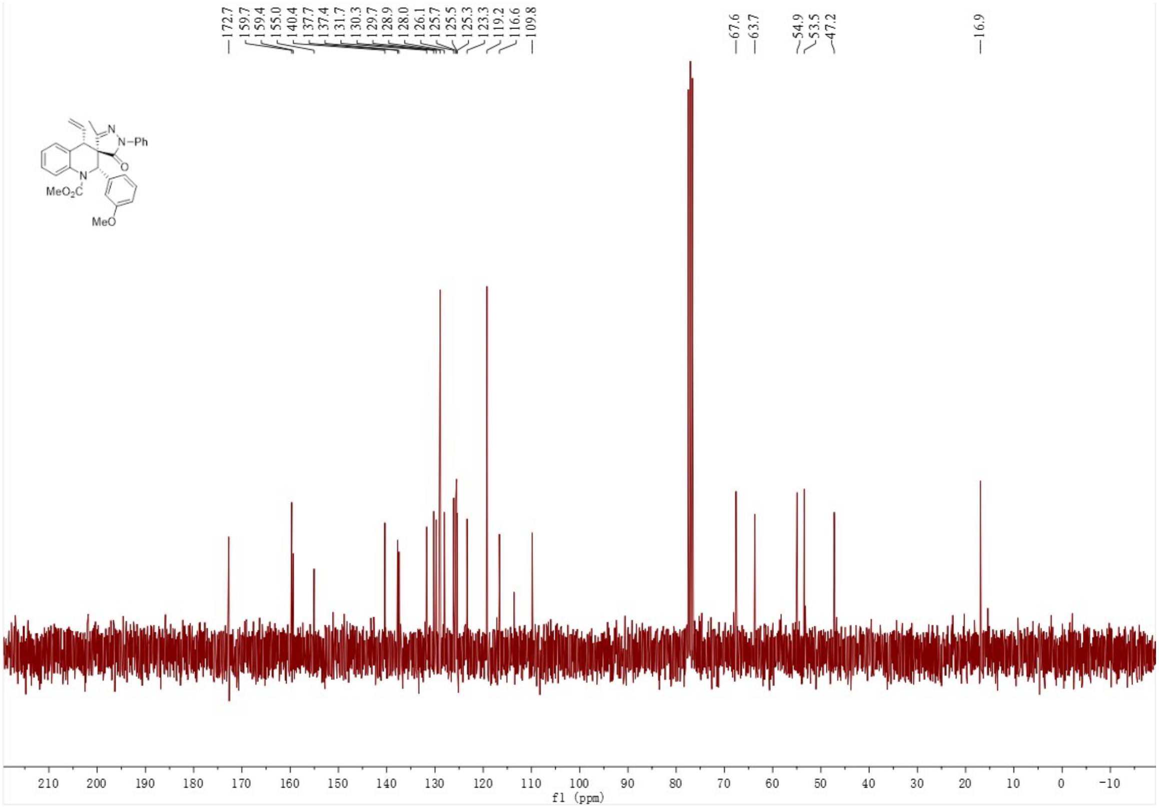

^{13}C ($CDCl_3$, 75 MHz) NMR of compound **3j**

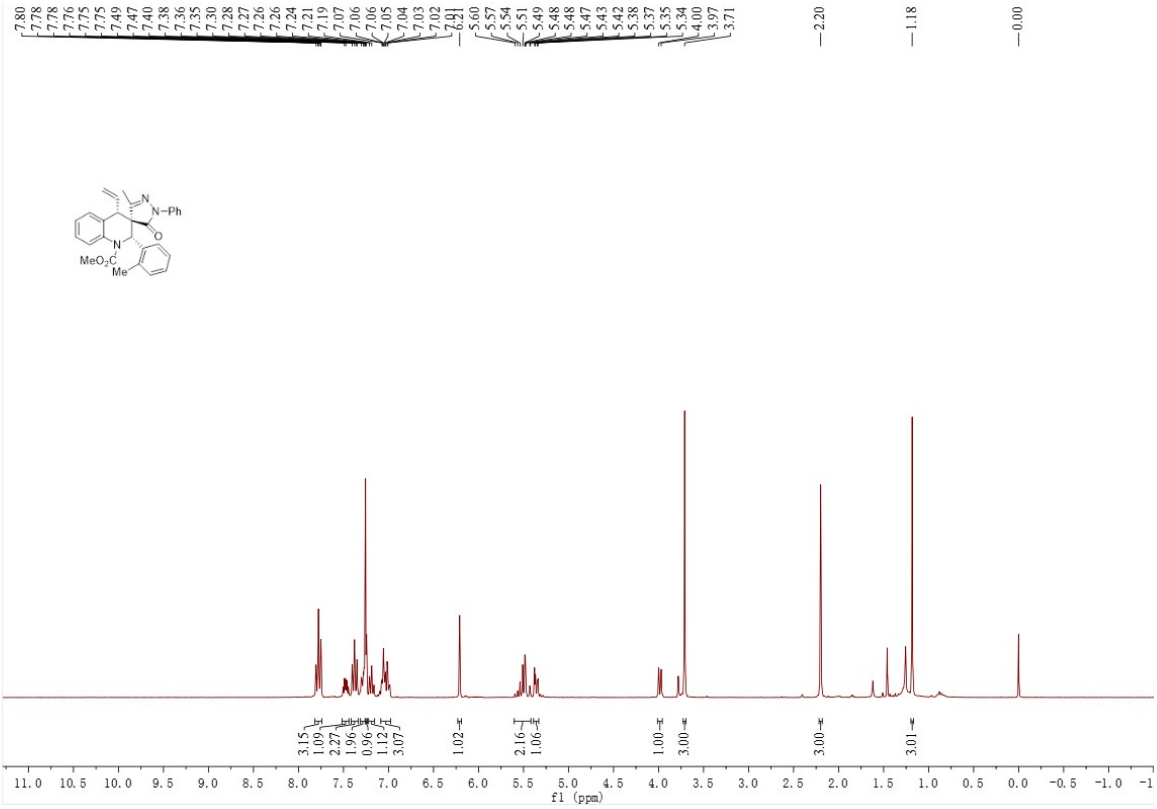

^{1}H ($CDCl_3$, 300 MHz) NMR of compound **3k**

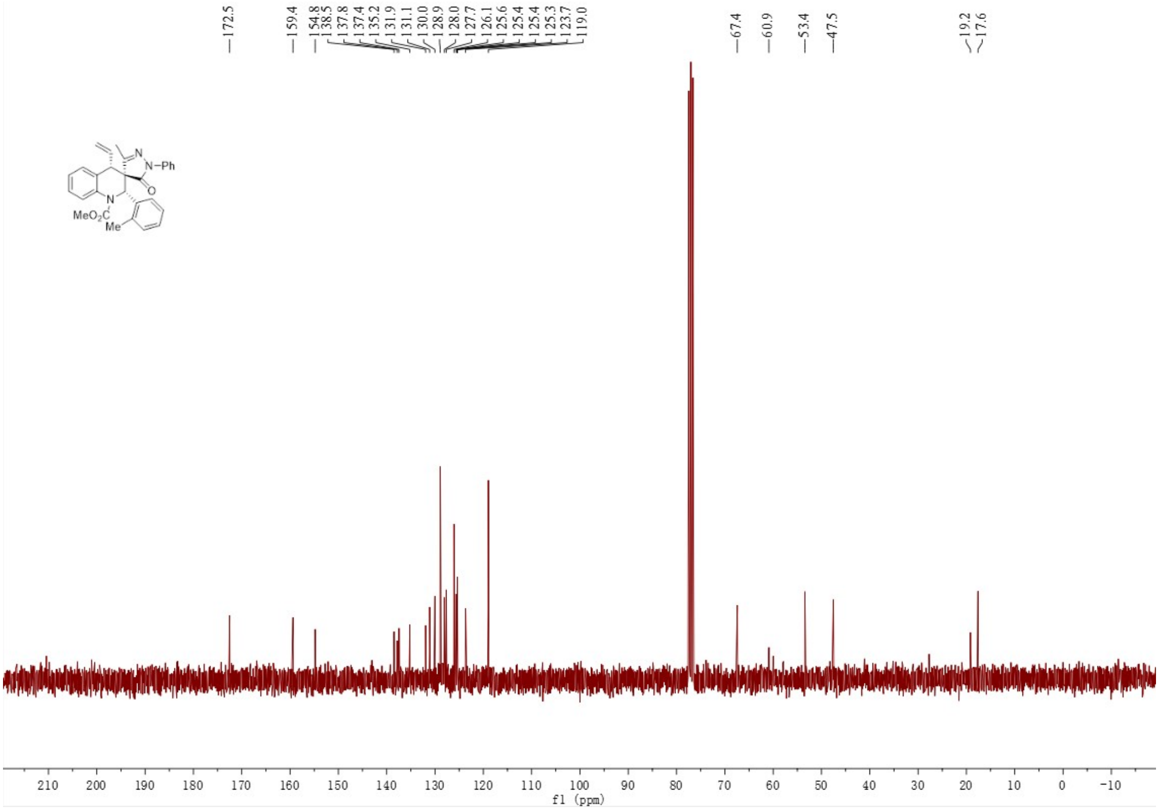

^{13}C ($CDCl_3$, 75 MHz) NMR of compound **3k**

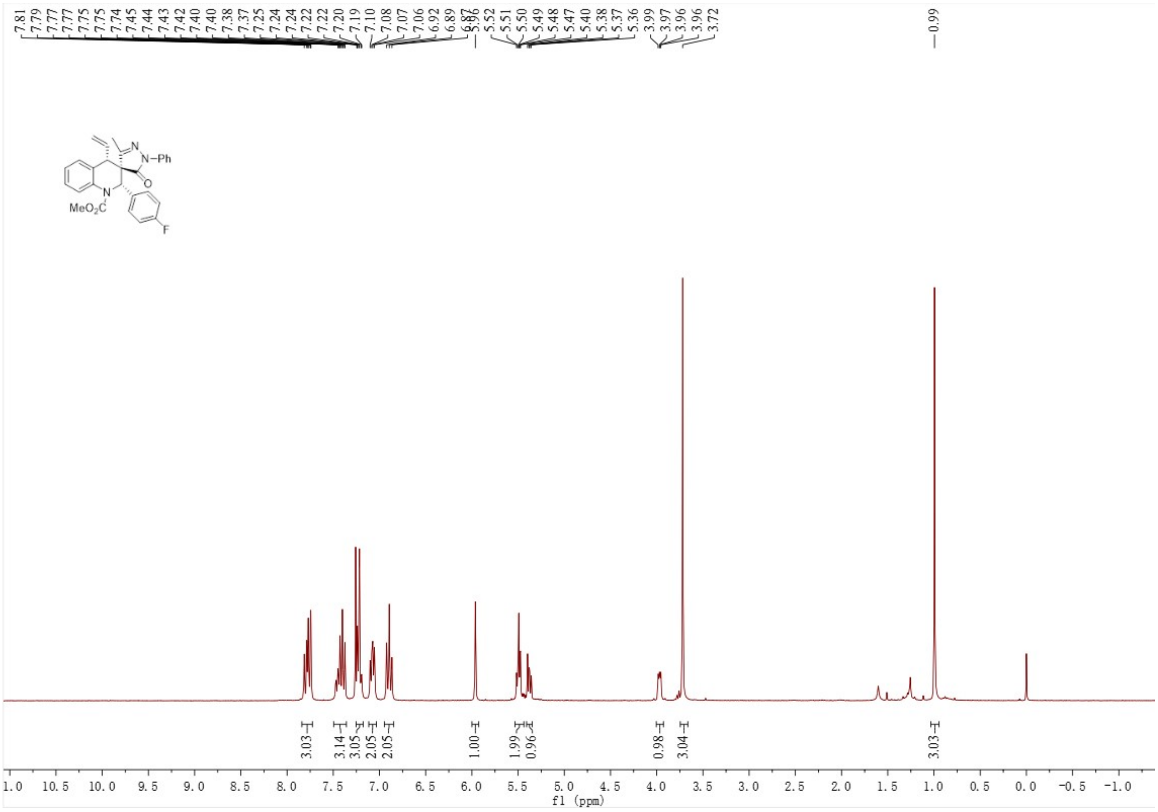

^{1}H ($CDCl_3$, 300 MHz) NMR of compound **3l**

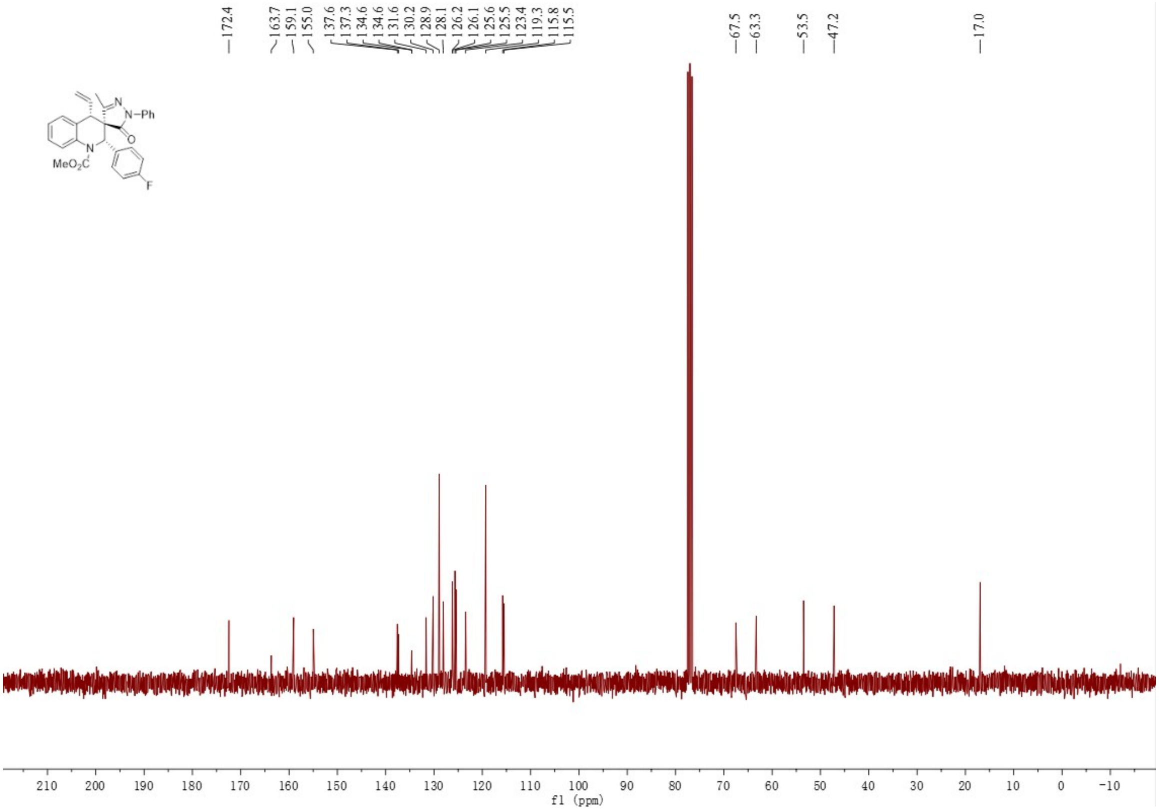

^{13}C ($CDCl_3$, 75 MHz) NMR of compound **3l**

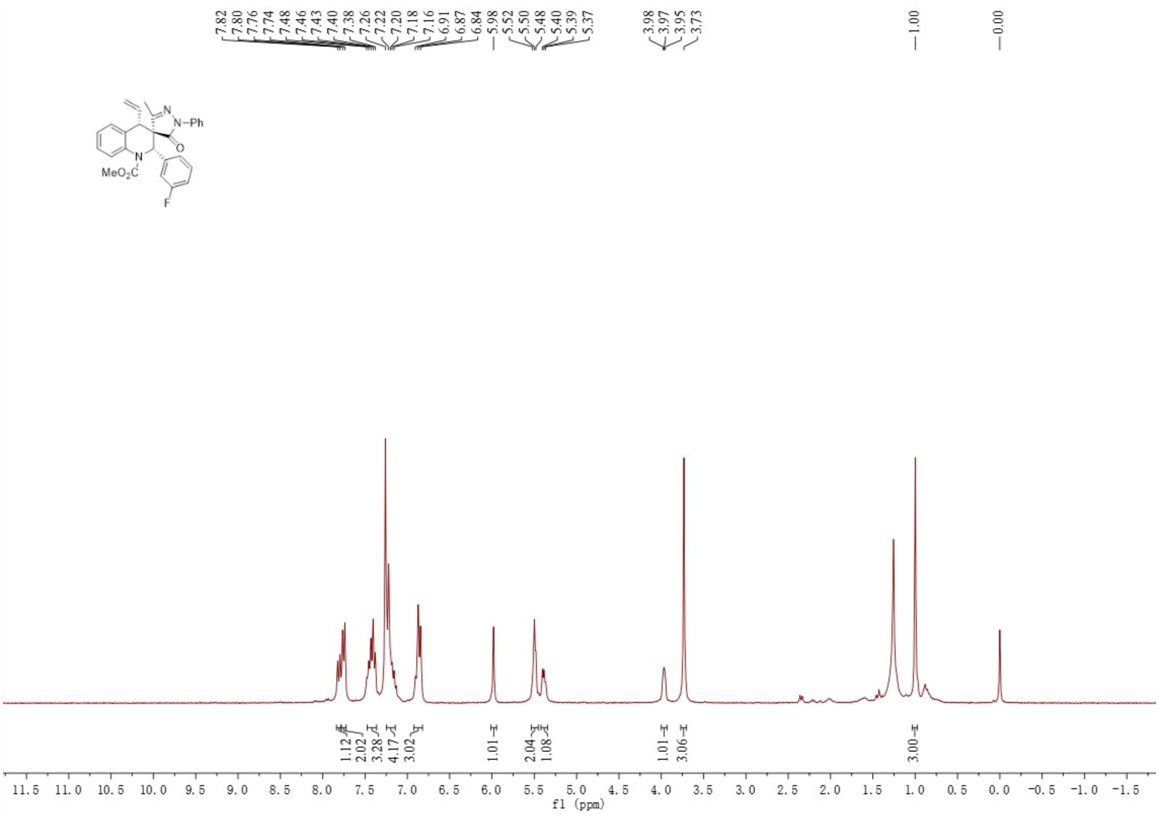

^{1}H ($CDCl_3$, 300 MHz) NMR of compound **3m**

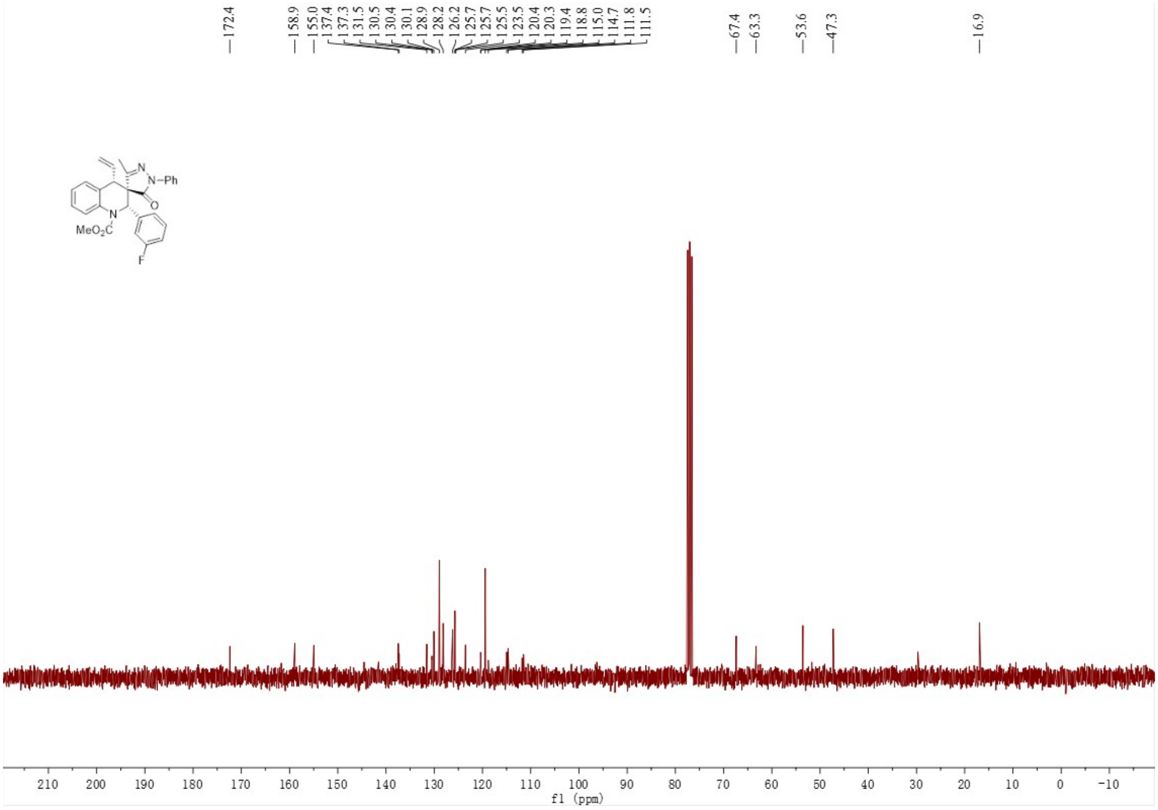

^{13}C ($CDCl_3$, 75 MHz) NMR of compound **3m**

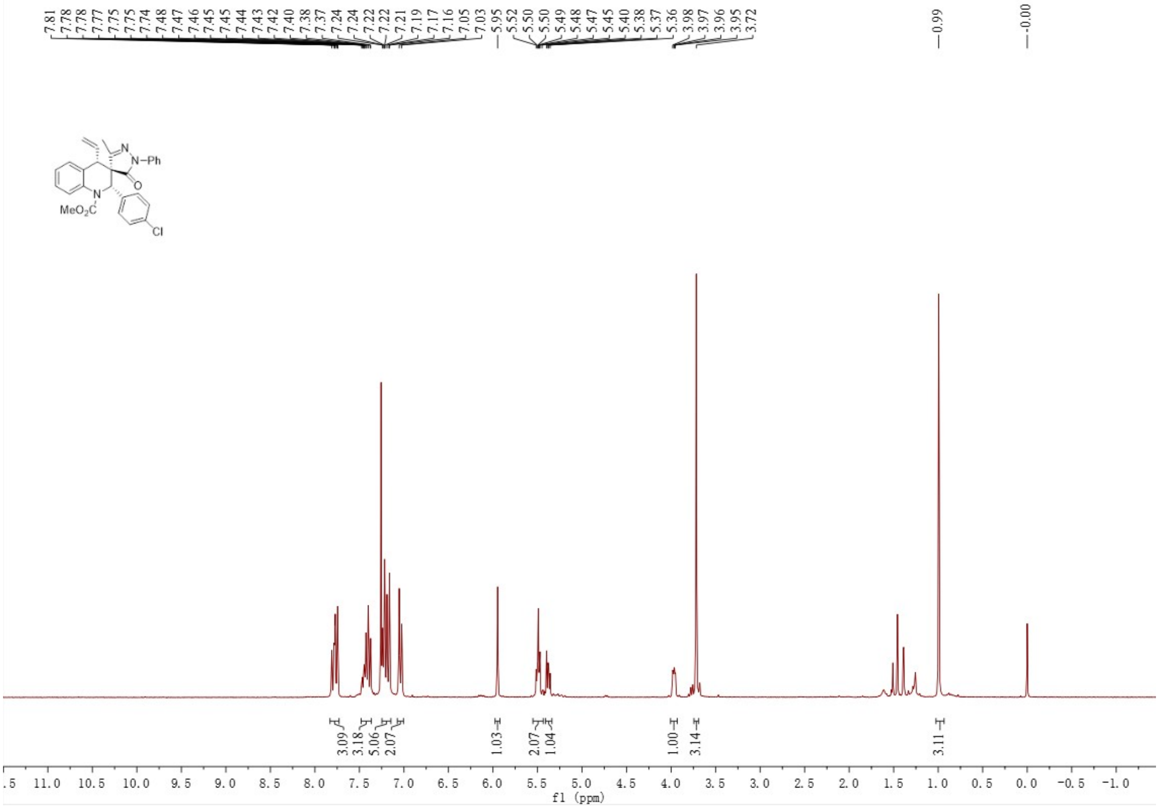

^{1}H ($CDCl_3$, 300 MHz) NMR of compound **3n**

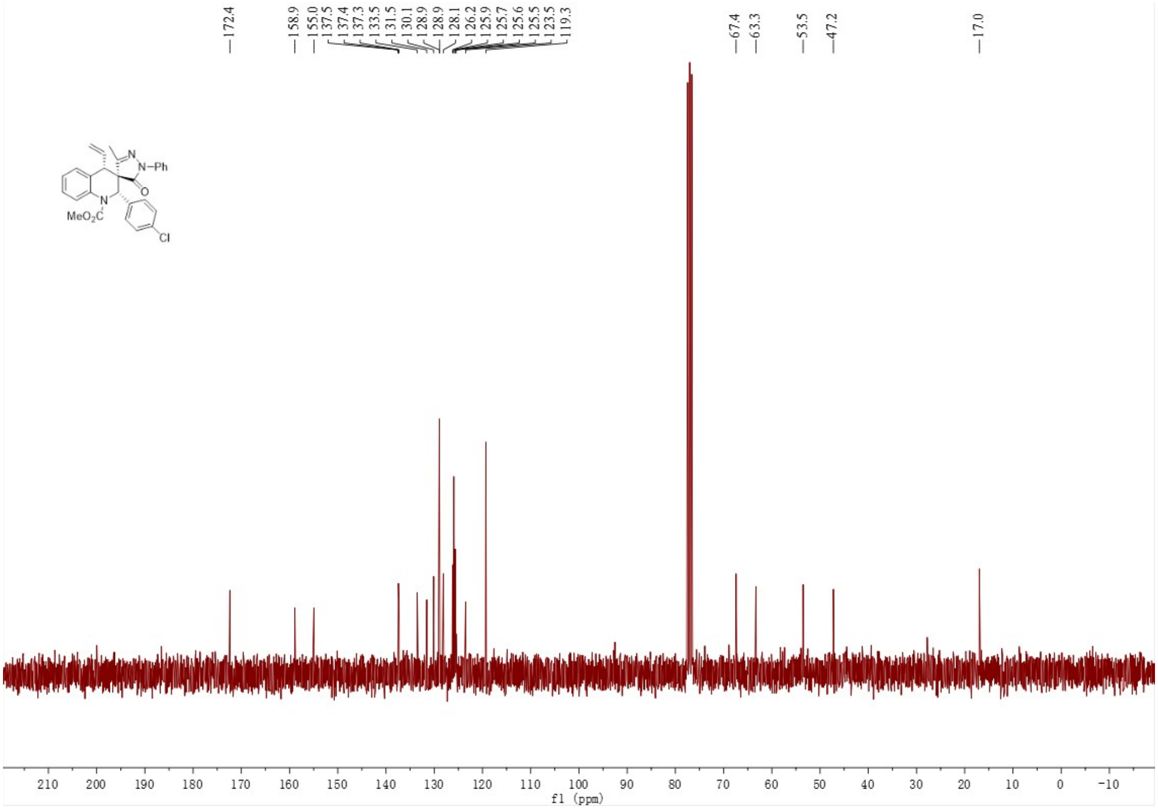

^{13}C ($CDCl_3$, 75 MHz) NMR of compound **3n**

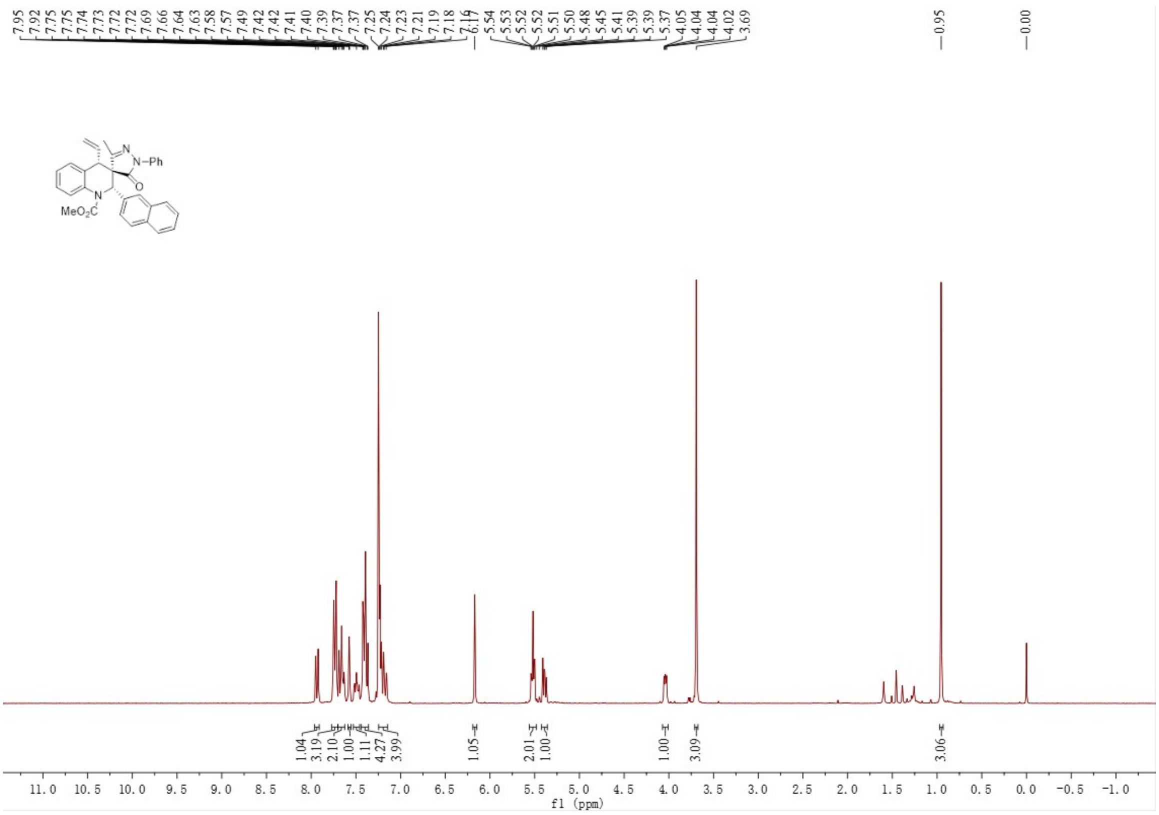

^{1}H ($CDCl_3$, 300 MHz) NMR of compound **3o**

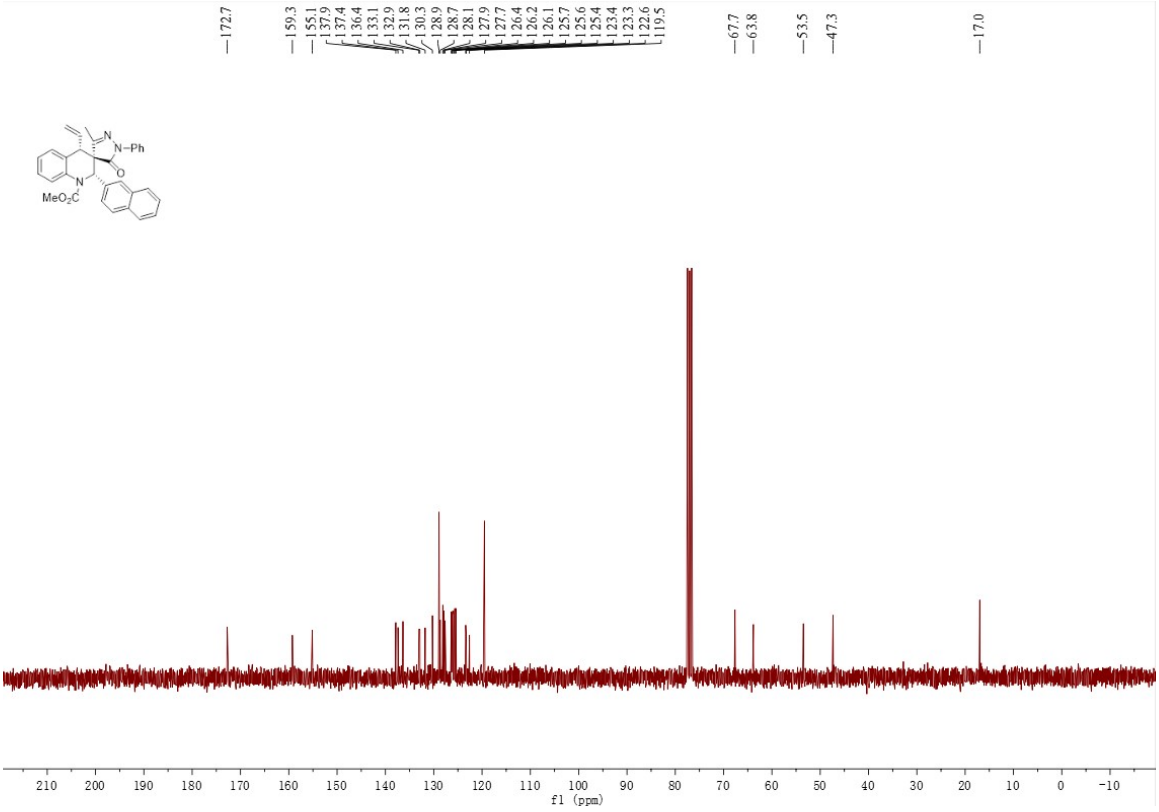

^{13}C ($CDCl_3$, 75 MHz) NMR of compound **3o**

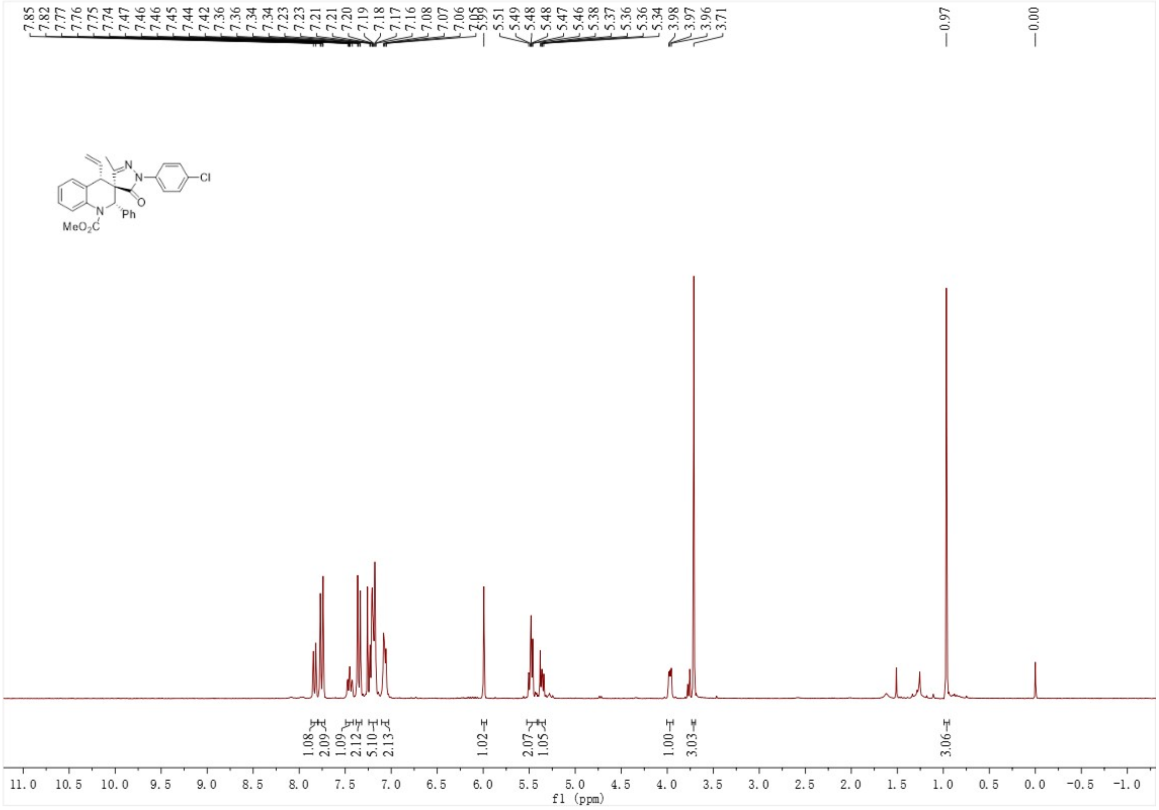

^{1}H ($CDCl_3$, 300 MHz) NMR of compound **3p**

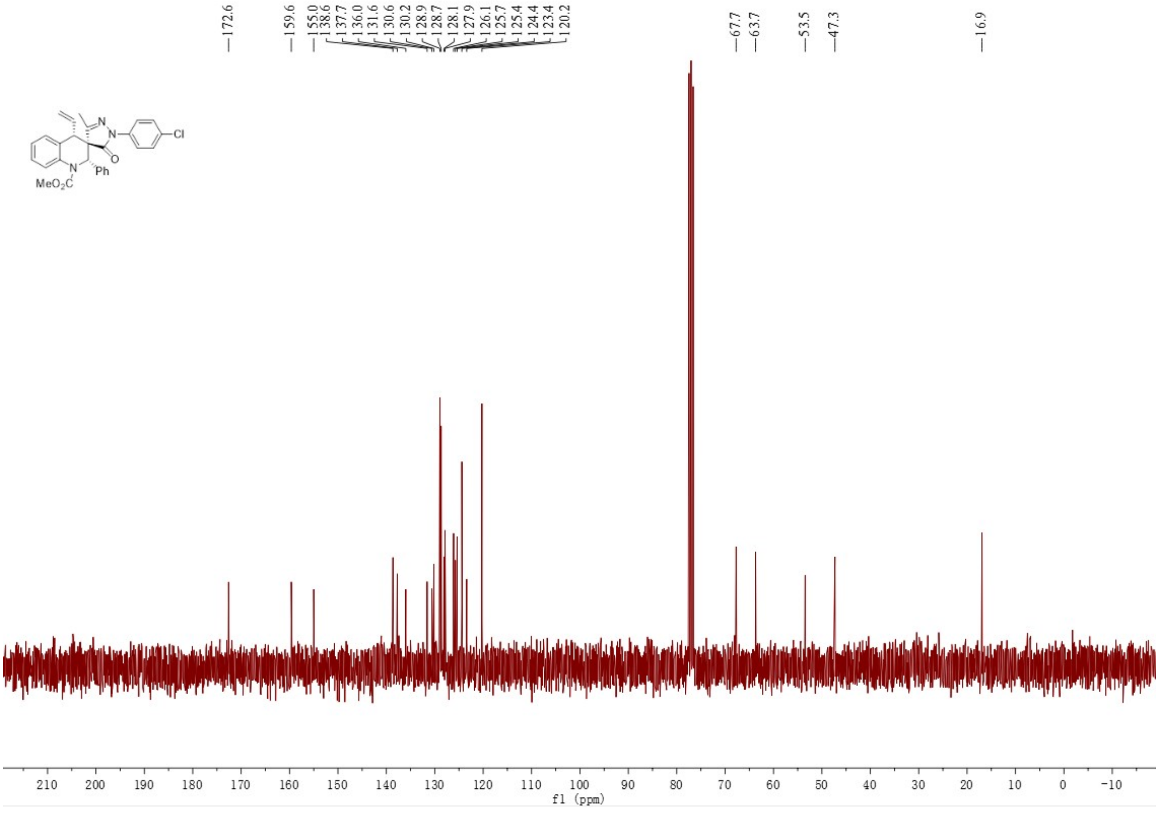

^{13}C ($CDCl_3$, 75 MHz) NMR of compound **3p**

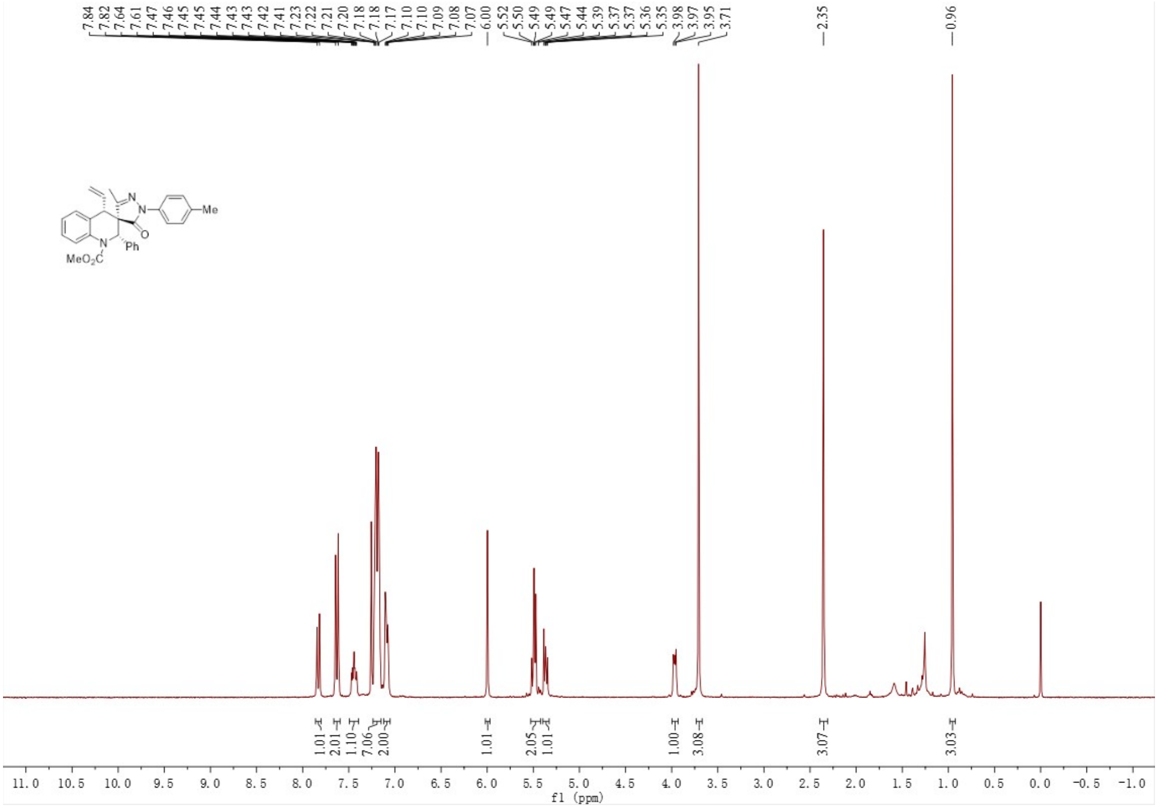

^{1}H ($CDCl_3$, 300 MHz) NMR of compound **3q**

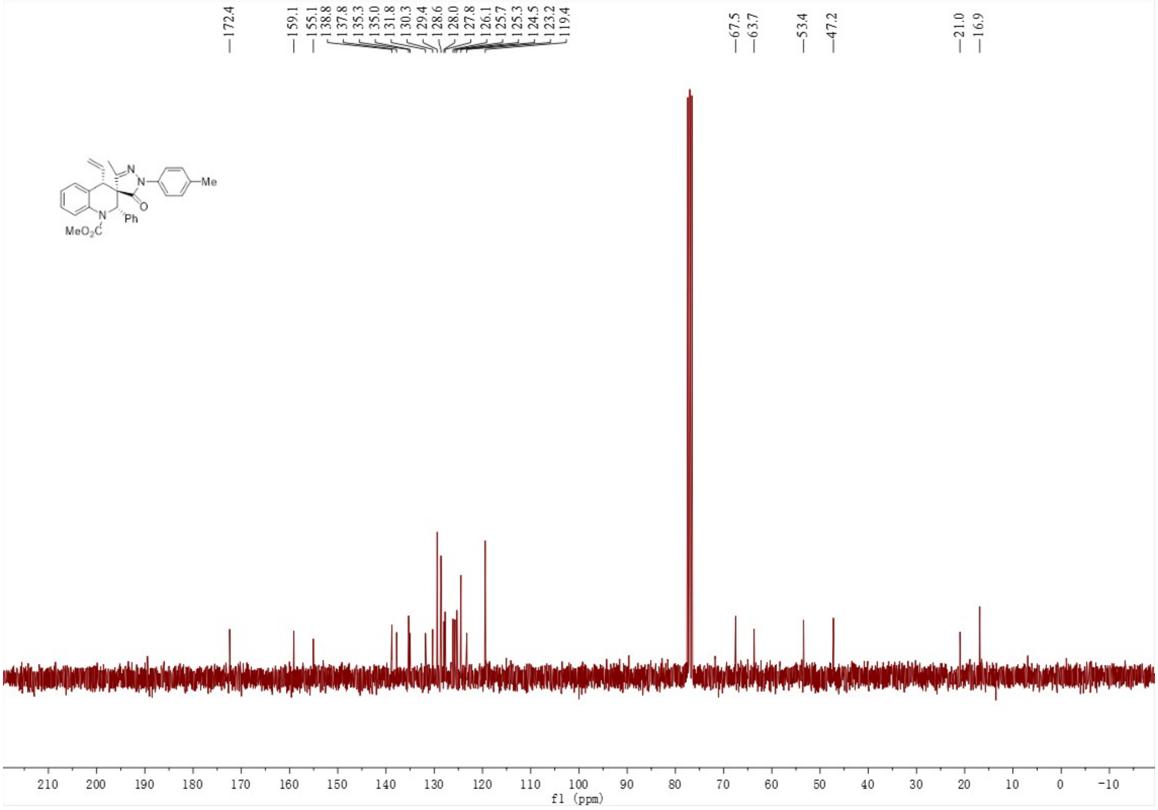

^{13}C ($CDCl_3$, 75 MHz) NMR of compound **3q**

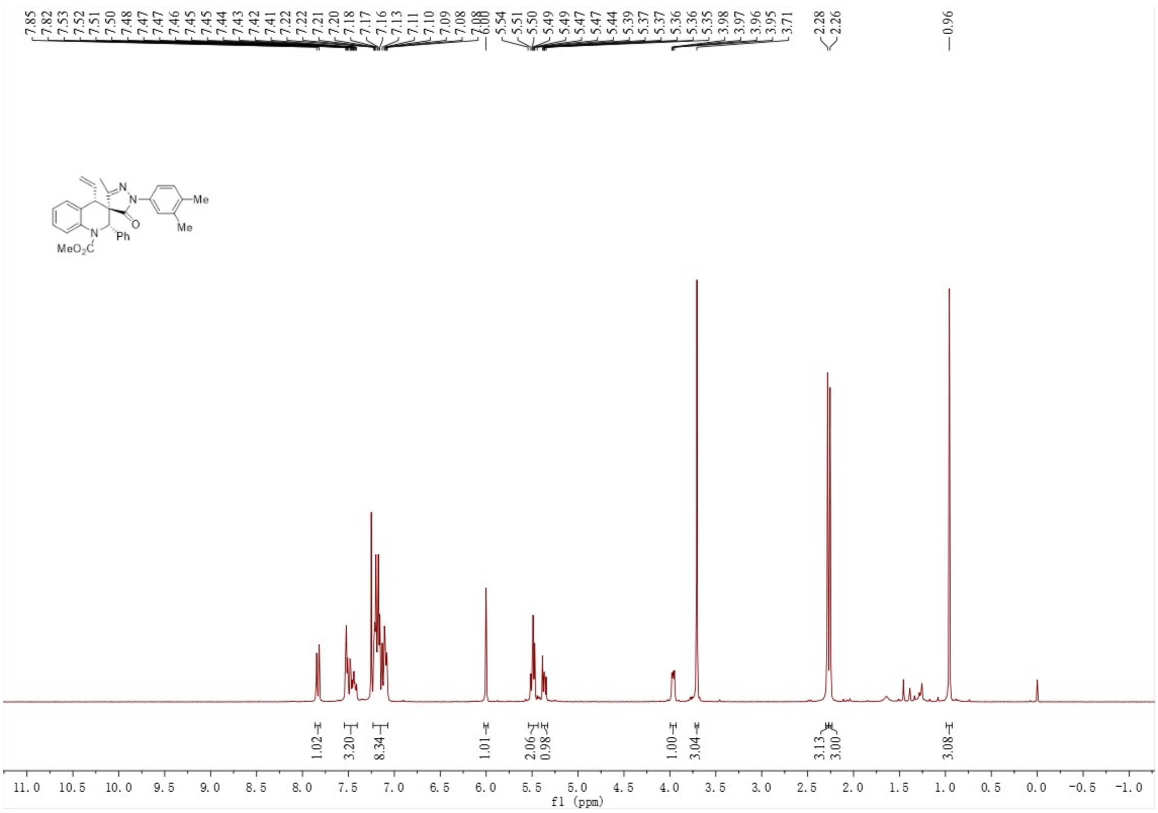

^{1}H ($CDCl_3$, 300 MHz) NMR of compound **3r**

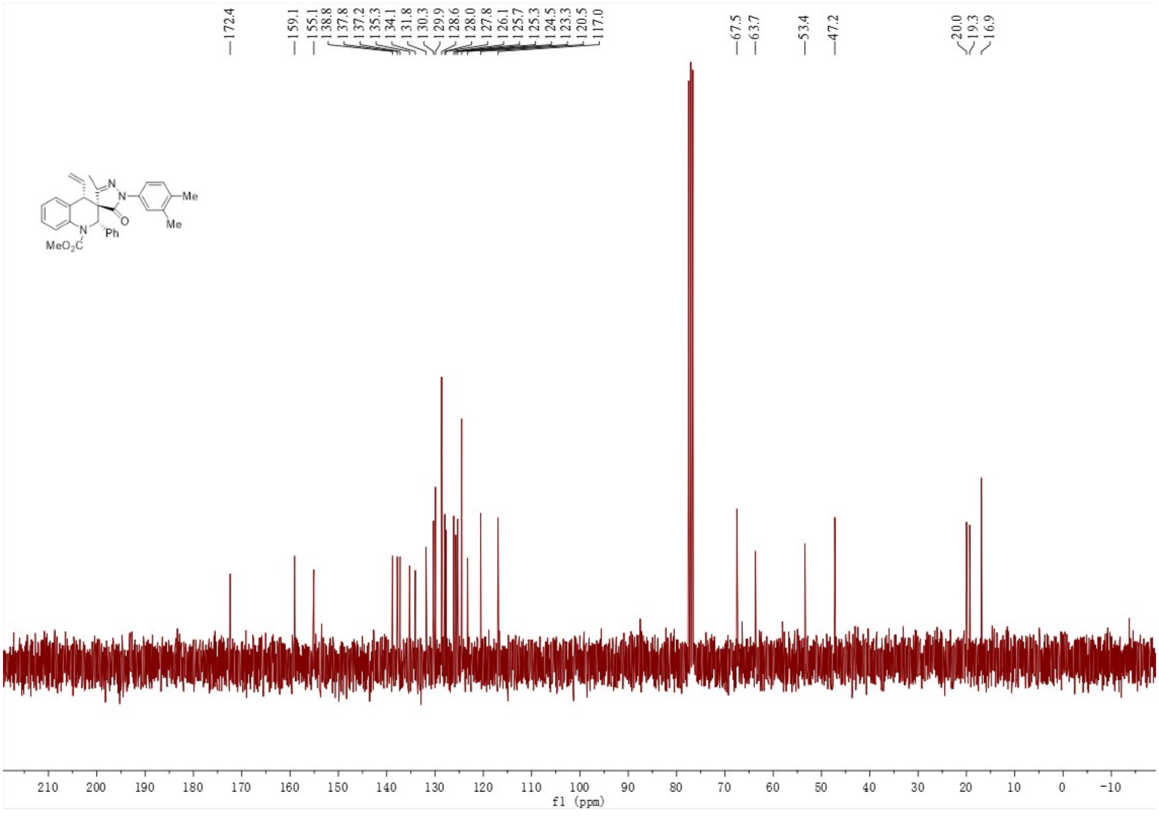

^{13}C ($CDCl_3$, 75 MHz) NMR of compound **3r**

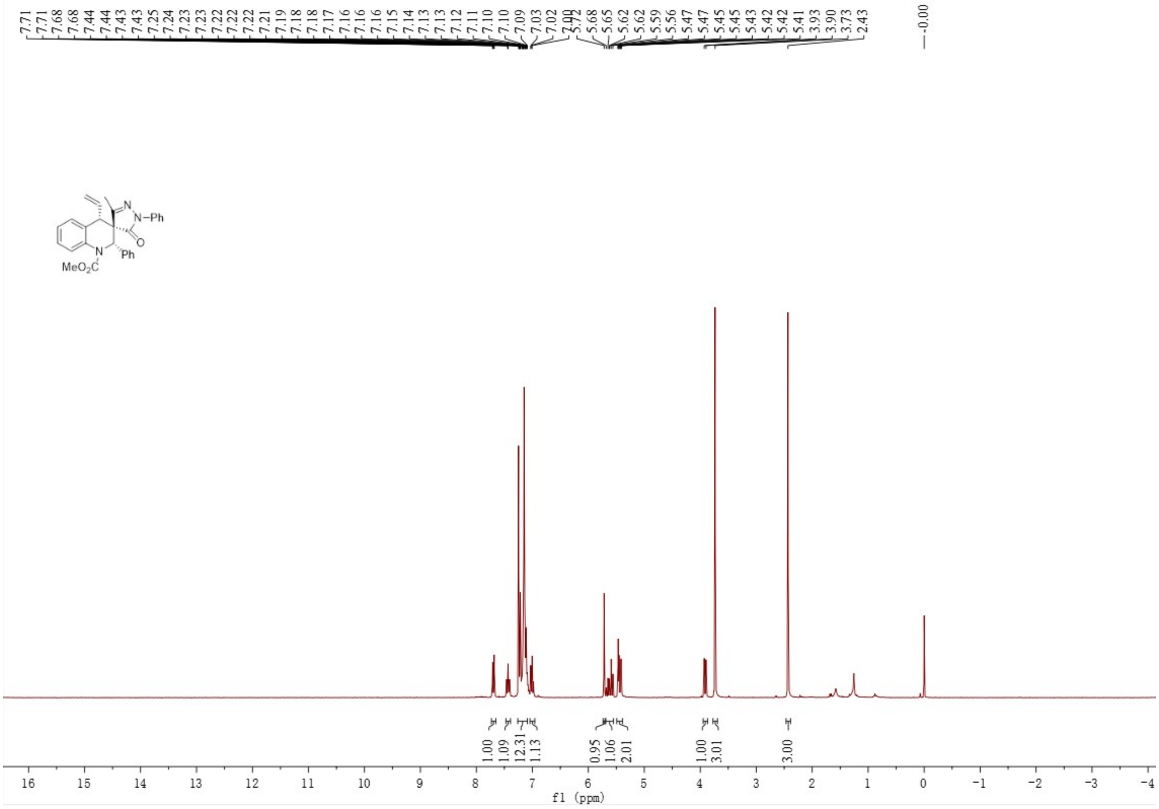

^{1}H ($CDCl_3$, 300 MHz) NMR of compound **4a**

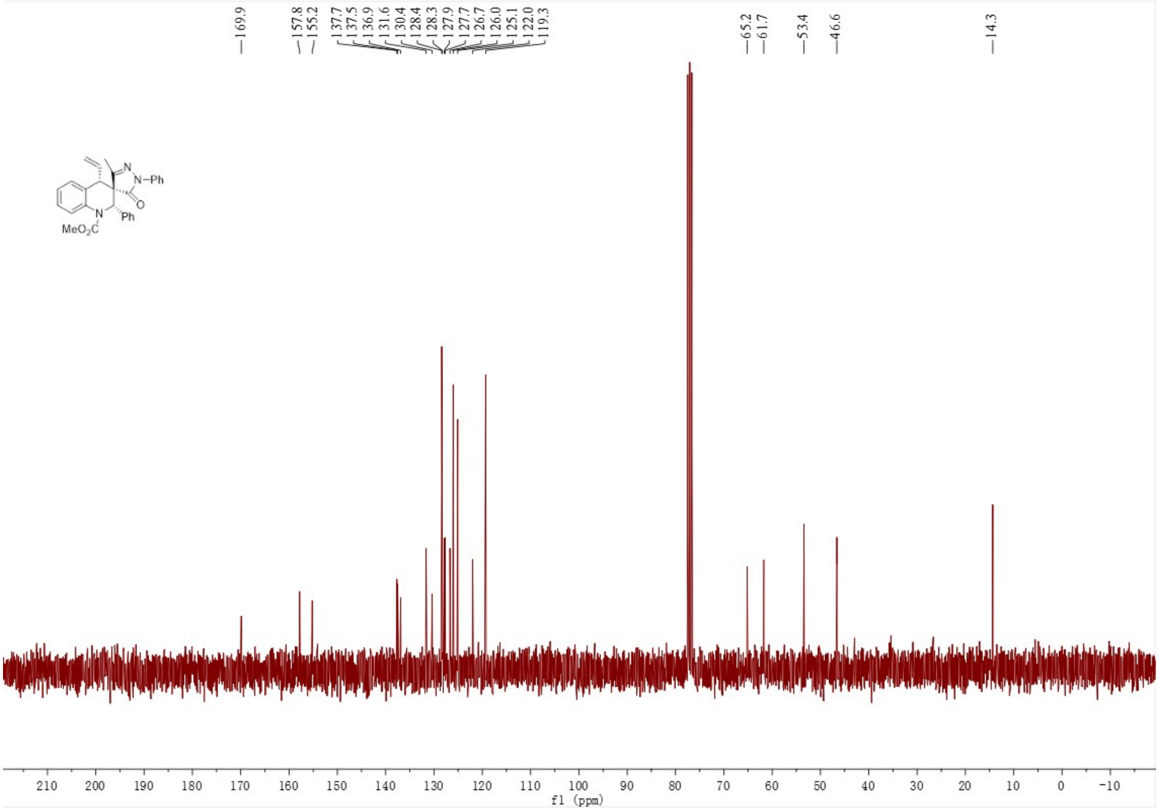

^{13}C ($CDCl_3$, 75 MHz) NMR of compound **4a**

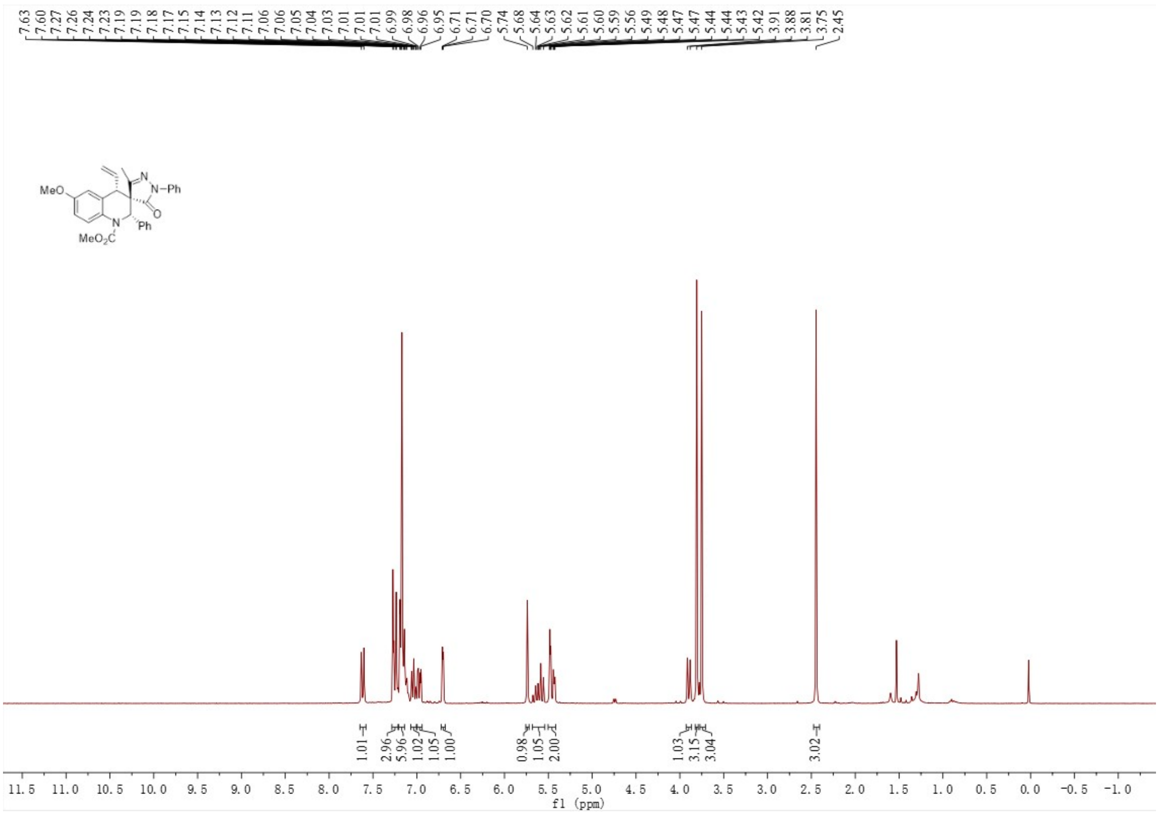

^{1}H ($CDCl_3$, 300 MHz) NMR of compound **4b**

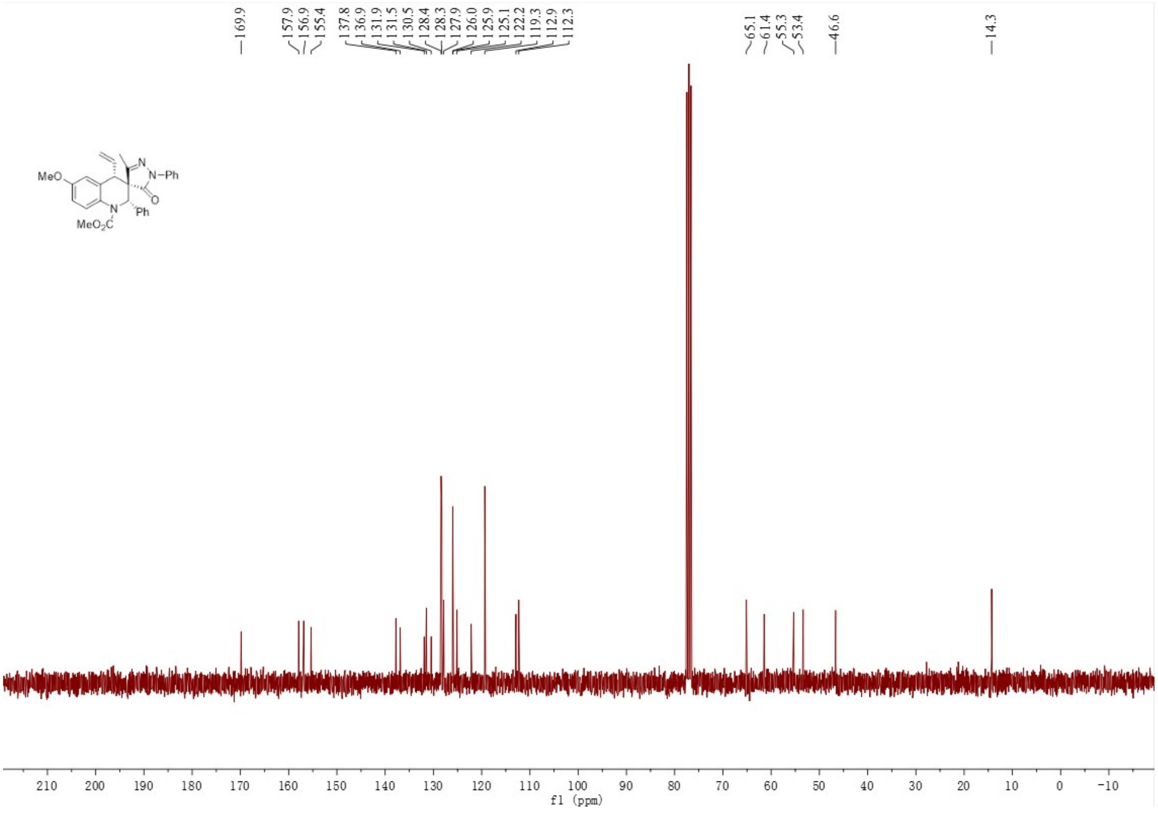

^{13}C ($CDCl_3$, 75 MHz) NMR of compound **4b**

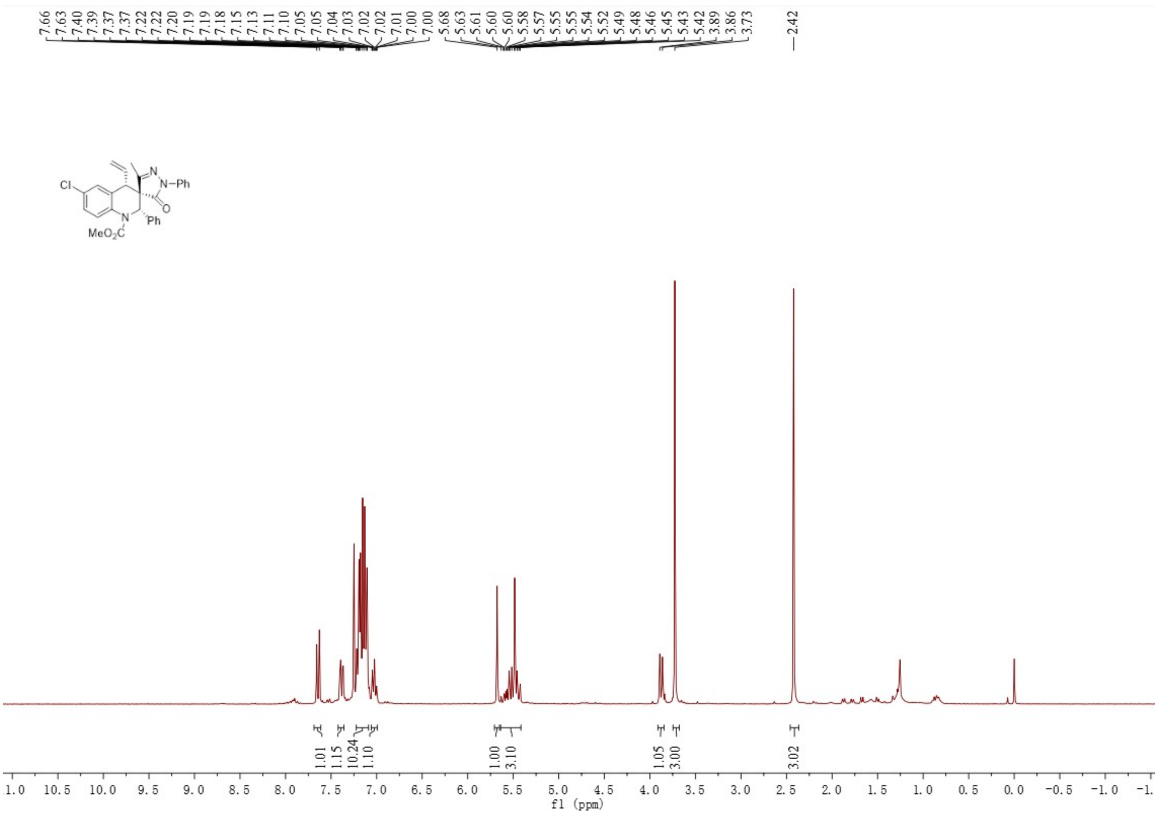

^{1}H ($CDCl_3$, 300 MHz) NMR of compound **4c**

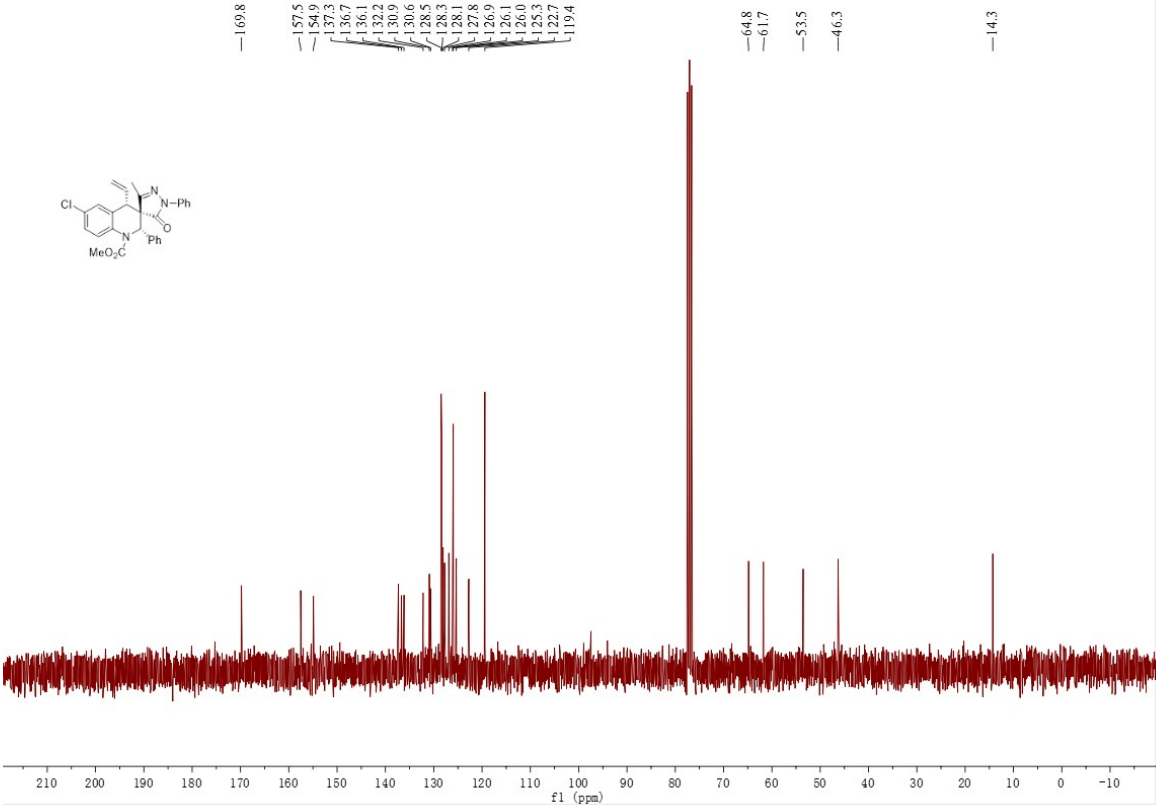

^{13}C ($CDCl_3$, 75 MHz) NMR of compound **4c**

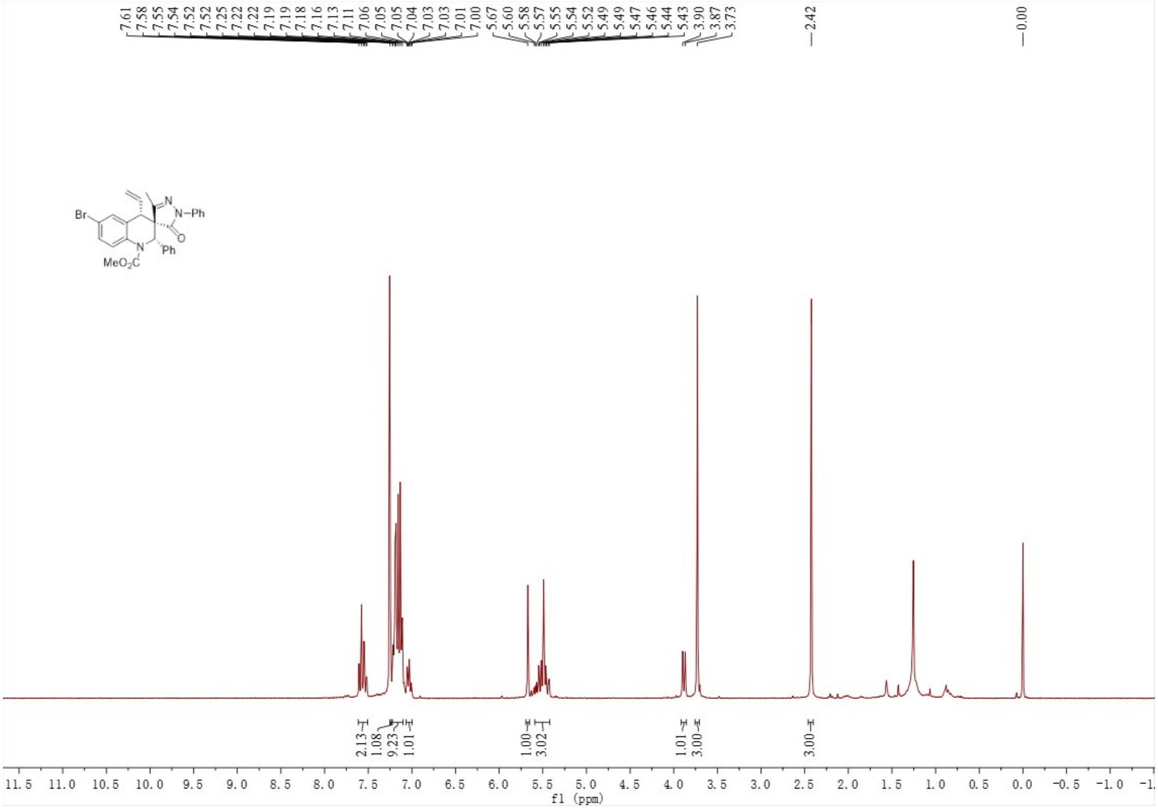

^1H ($CDCl_3$, 300 MHz) NMR of compound **4d**

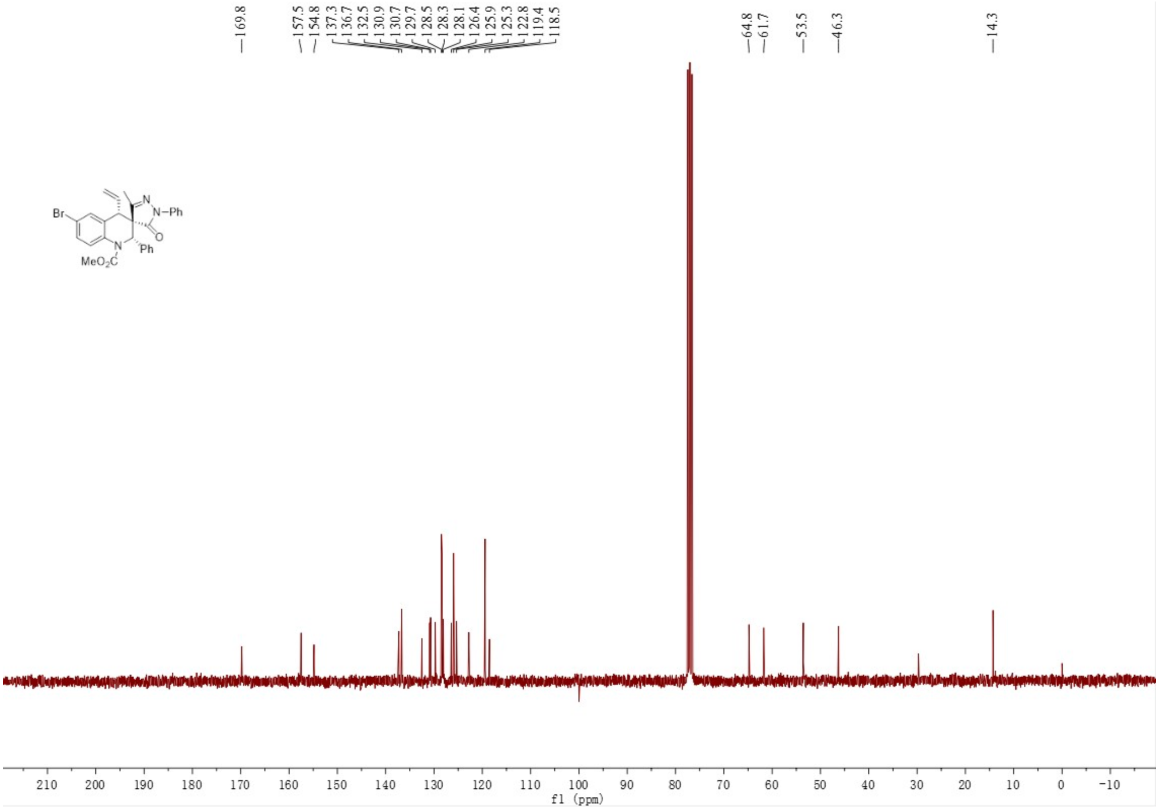

^{13}C ($CDCl_3$, 75 MHz) NMR of compound **4d**

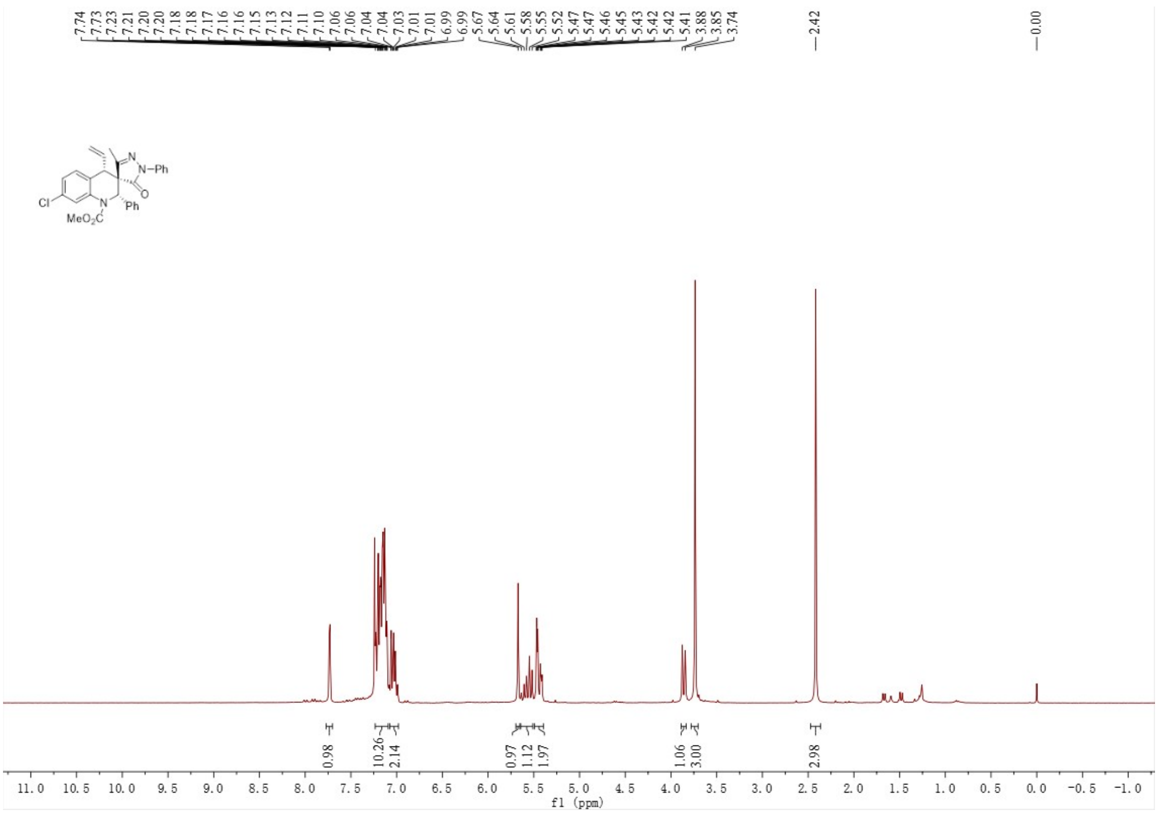

^{1}H ($CDCl_3$, 300 MHz) NMR of compound **4e**

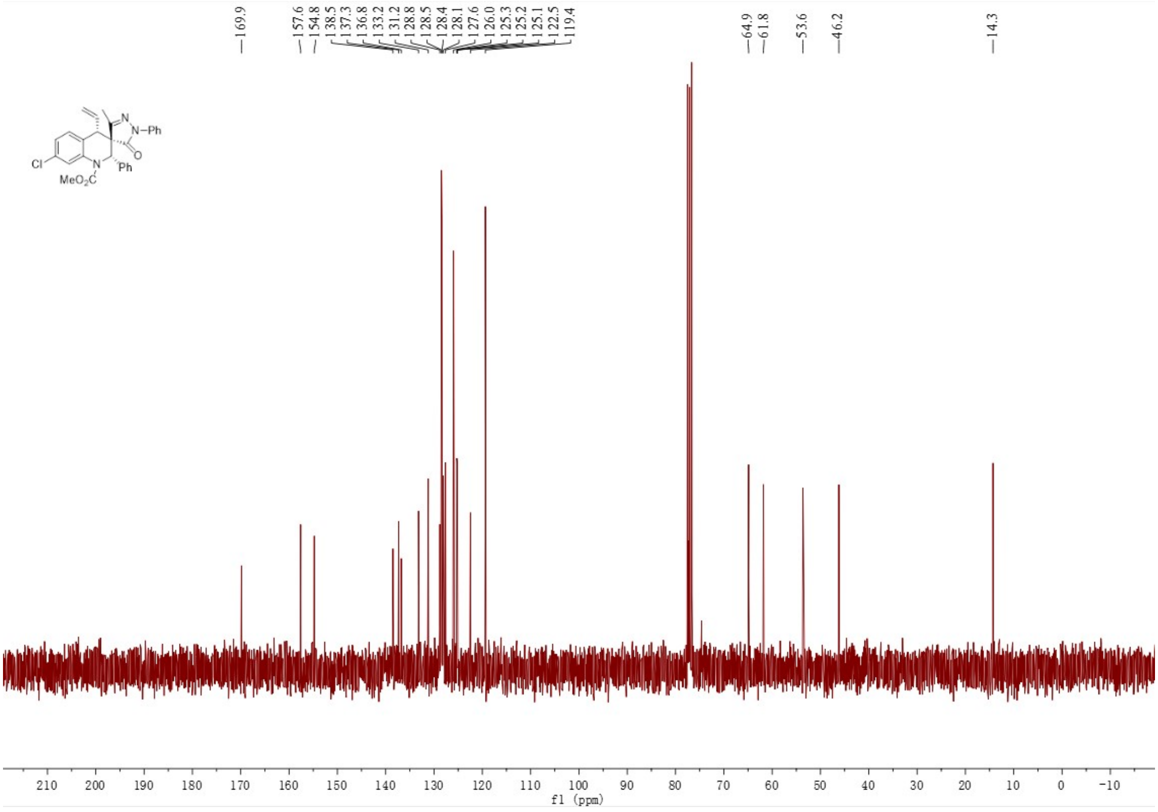

^{13}C ($CDCl_3$, 75 MHz) NMR of compound **4e**

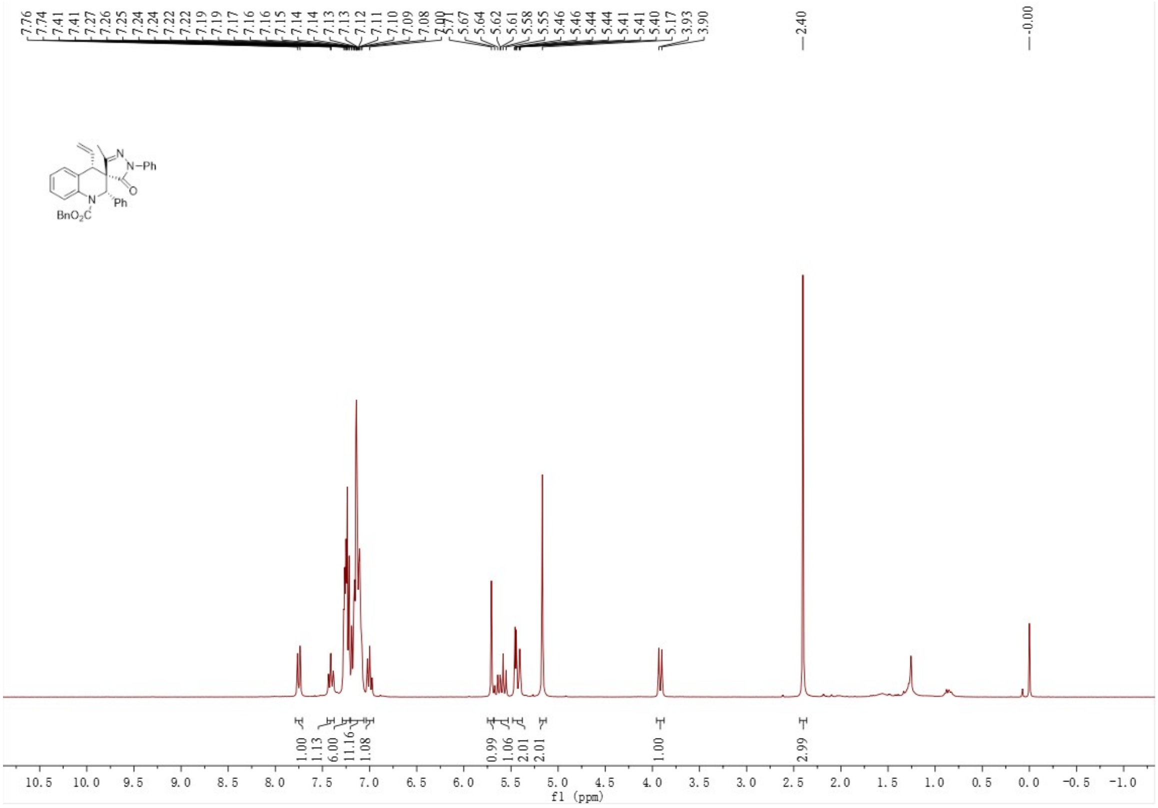

^{1}H ($CDCl_3$, 300 MHz) NMR of compound **4f**

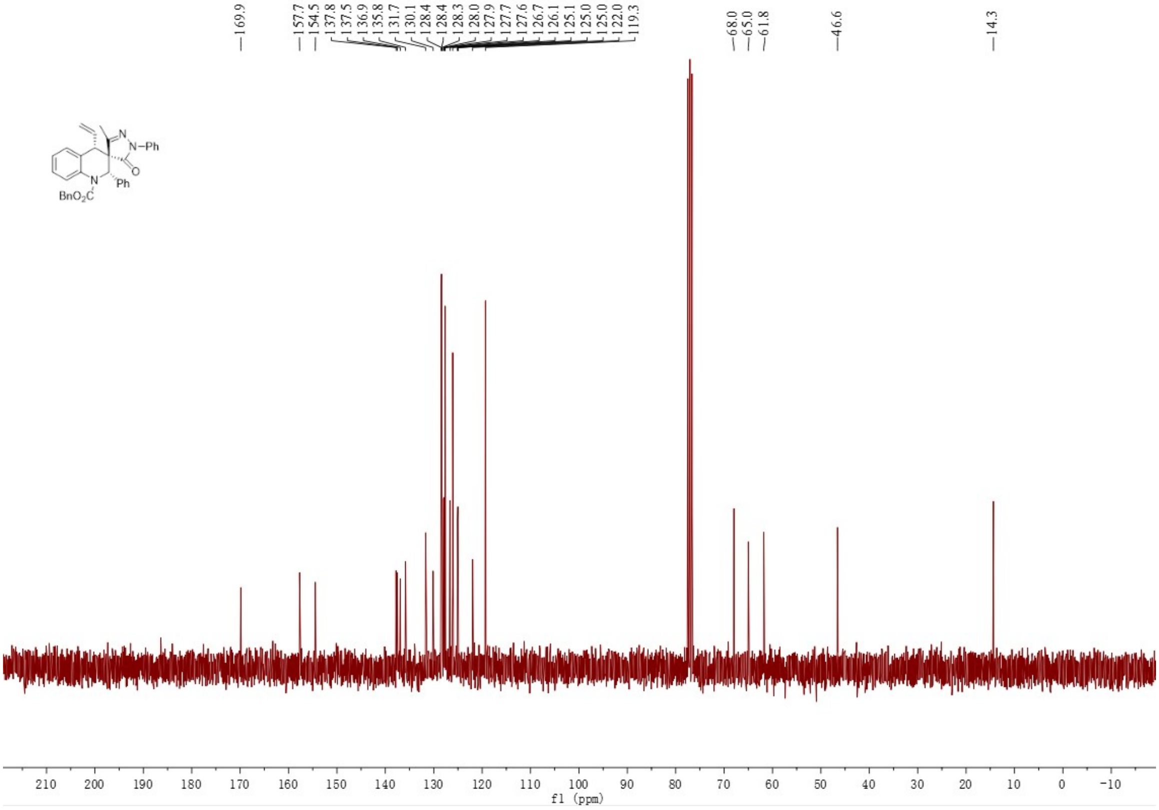

^{13}C ($CDCl_3$, 75 MHz) NMR of compound **4f**

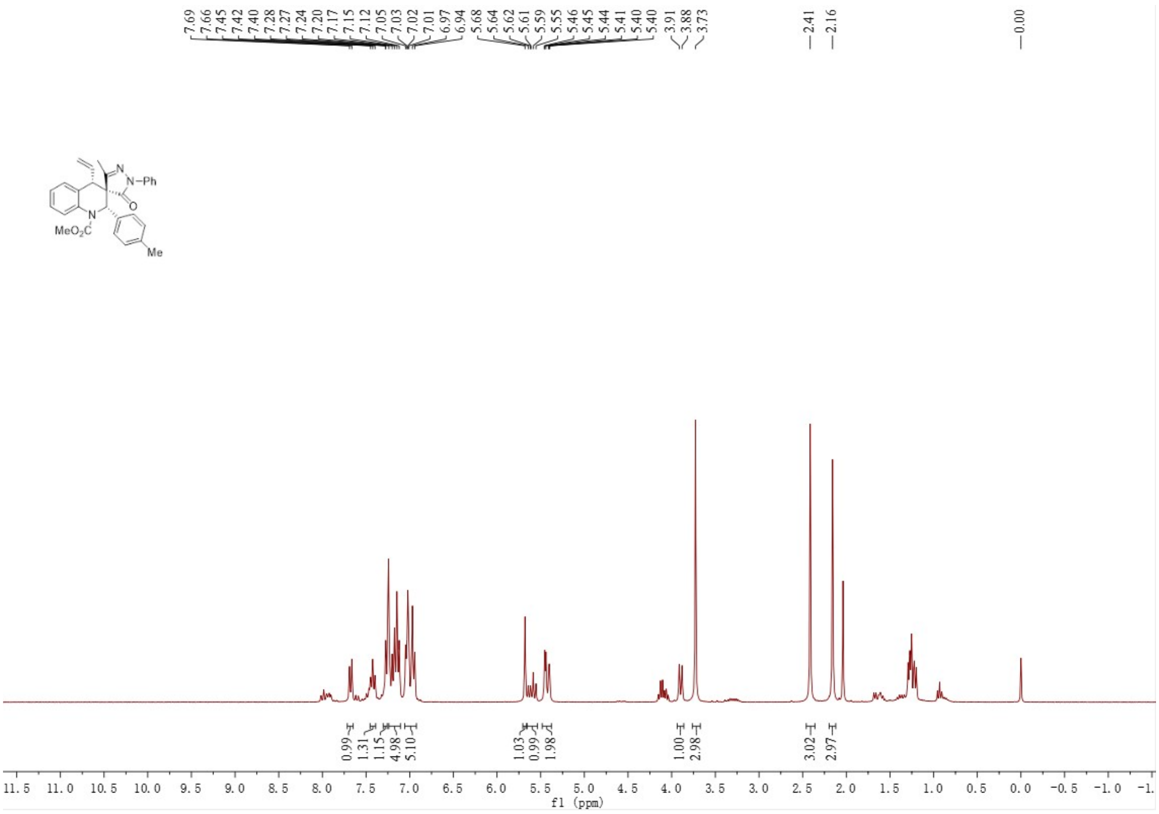

^{1}H ($CDCl_3$, 300 MHz) NMR of compound **4g**

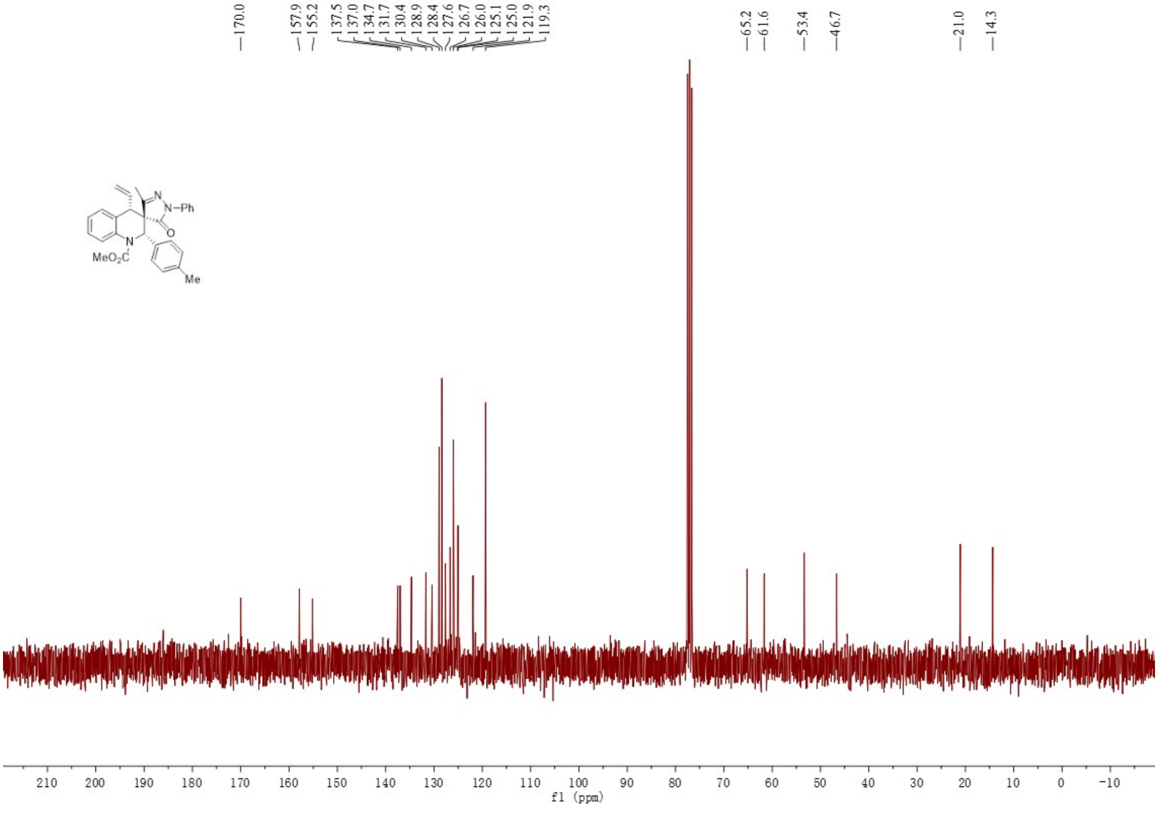

^{13}C ($CDCl_3$, 75 MHz) NMR of compound **4g**

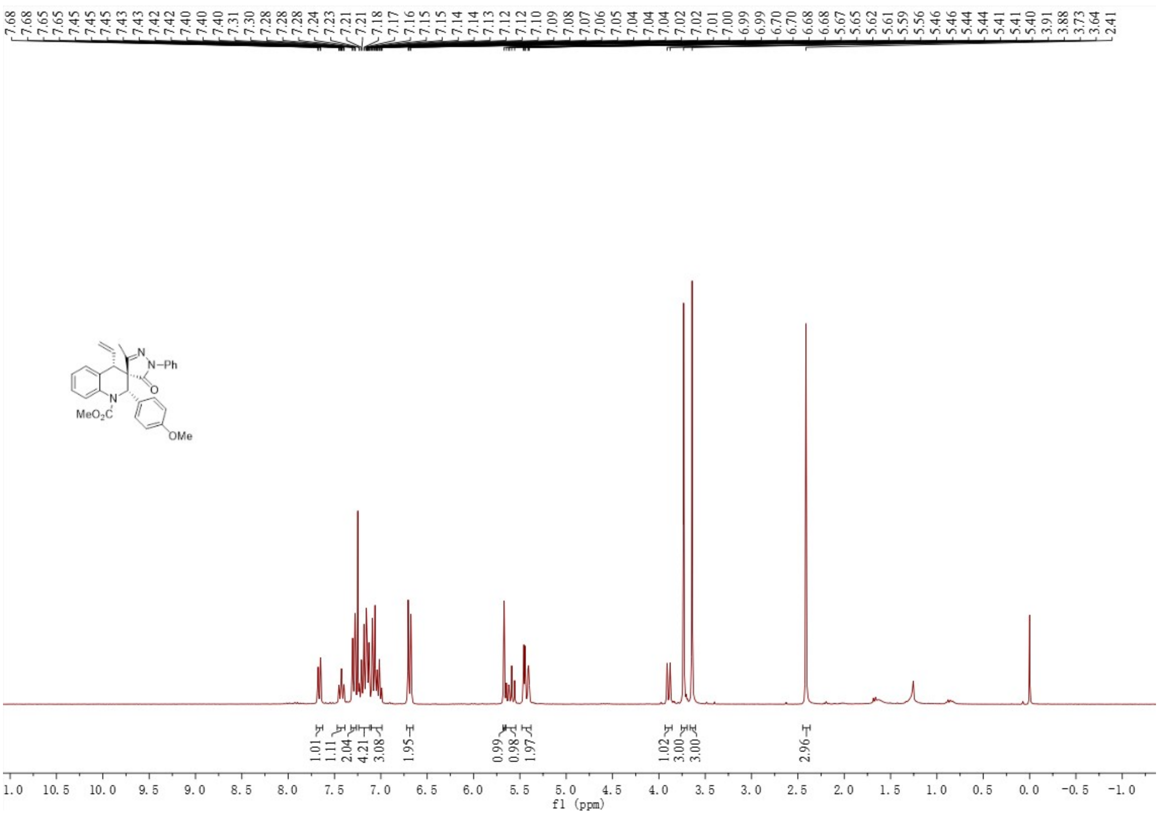

^{1}H ($CDCl_3$, 300 MHz) NMR of compound **4h**

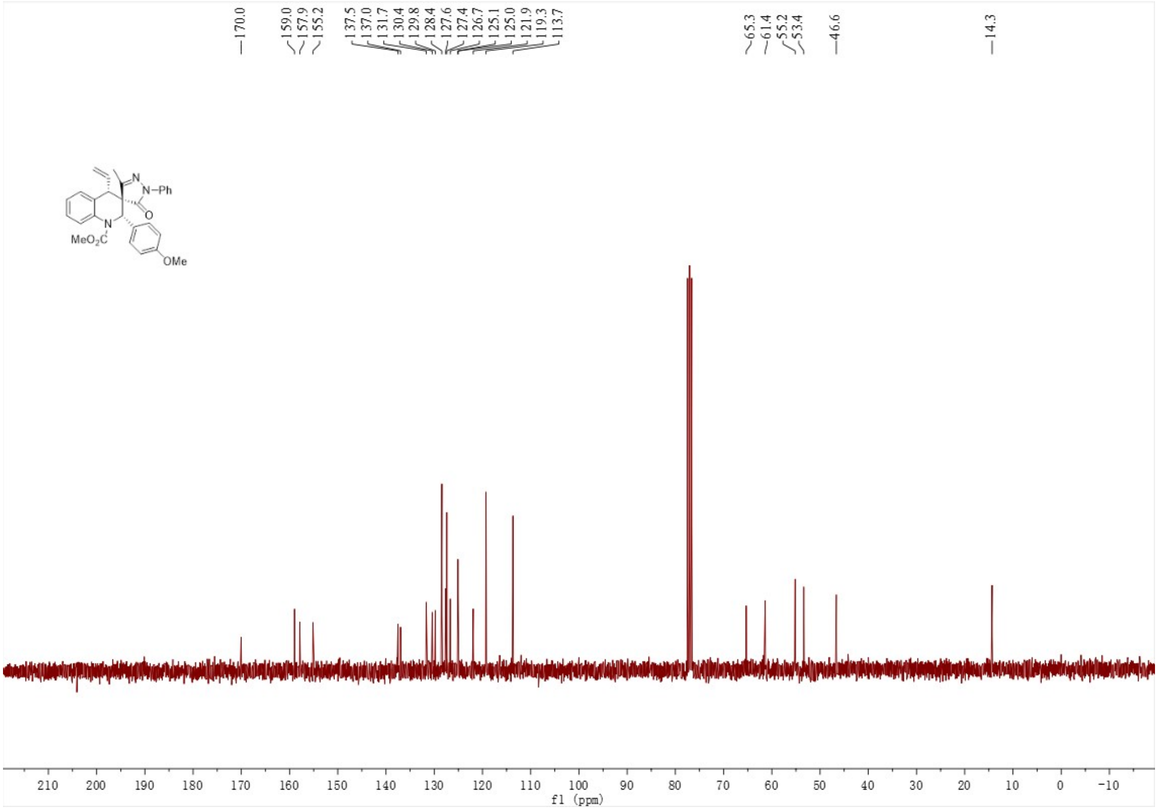

^{13}C ($CDCl_3$, 75 MHz) NMR of compound **4h**

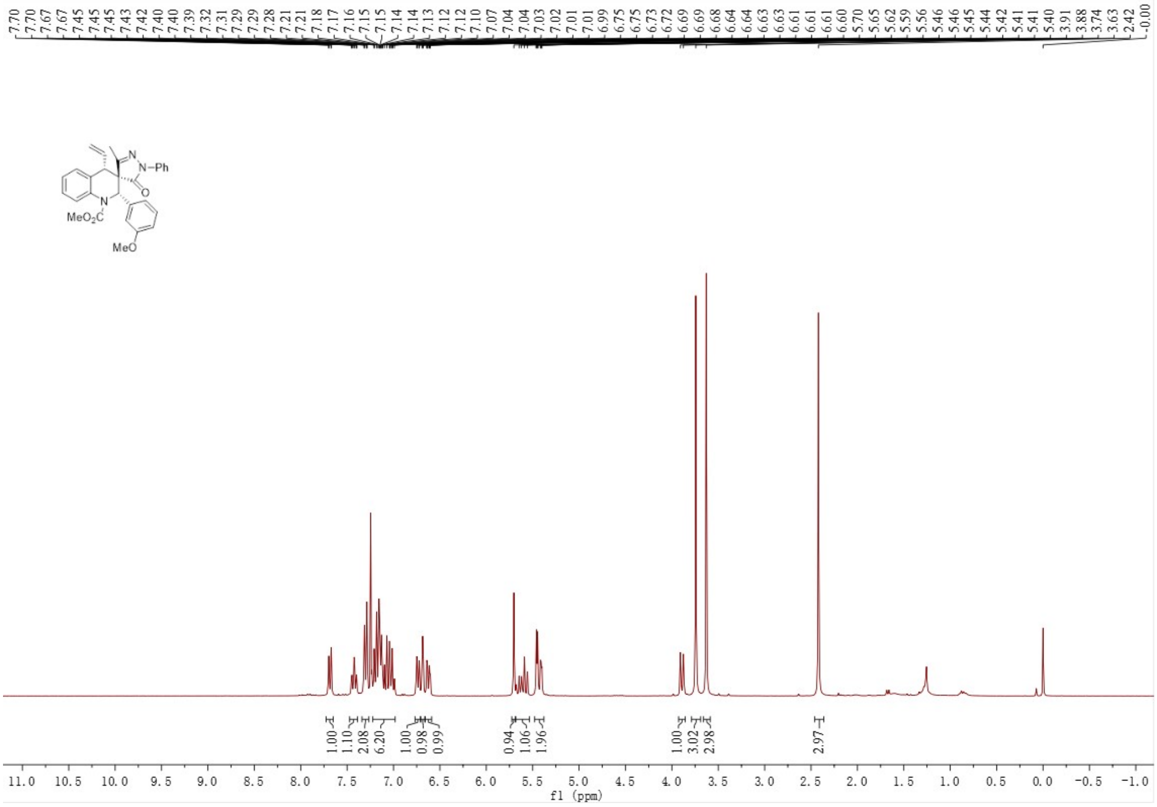

^{1}H ($CDCl_3$, 300 MHz) NMR of compound **4i**

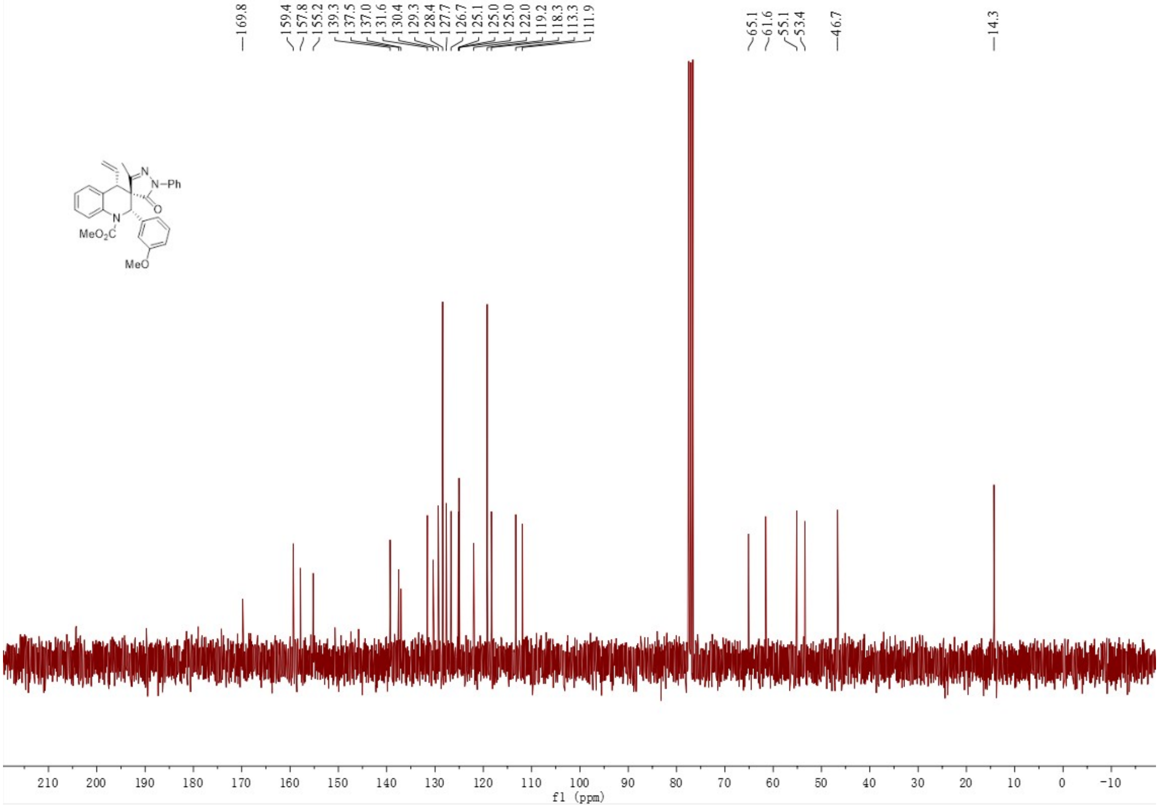

^{13}C ($CDCl_3$, 75 MHz) NMR of compound **4i**

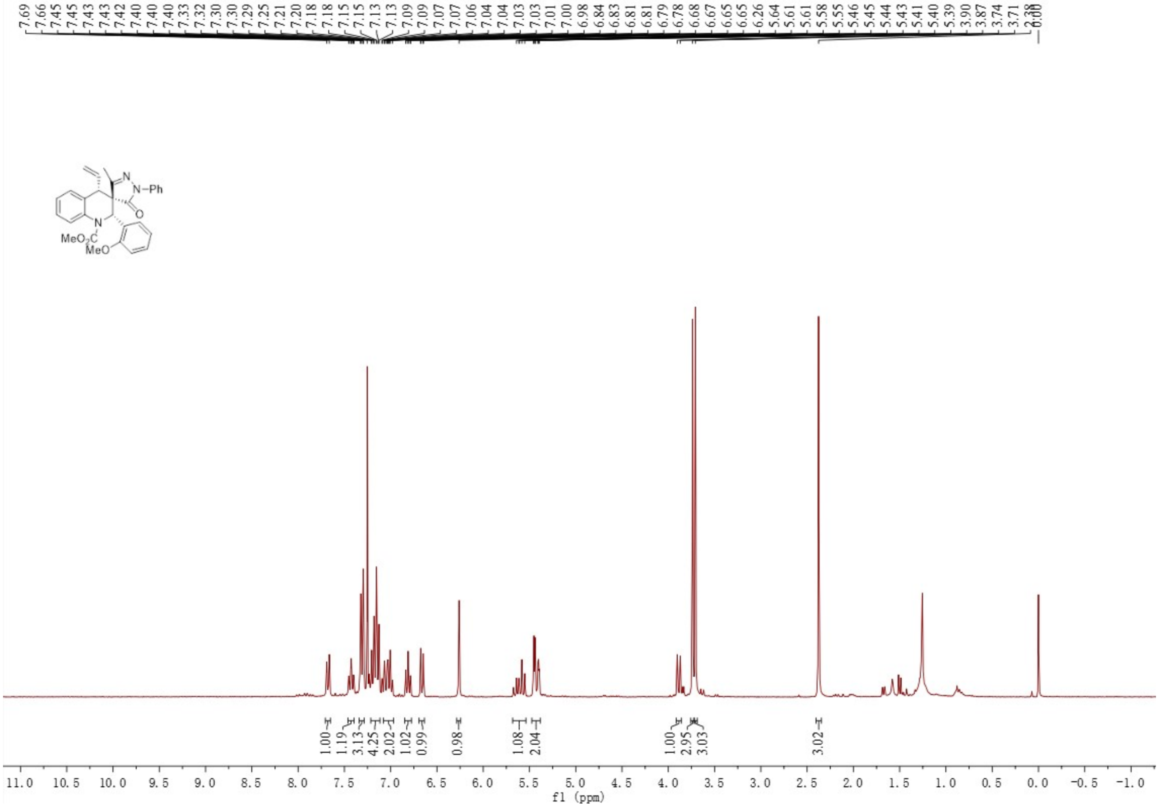

^{1}H ($CDCl_3$, 300 MHz) NMR of compound **4j**

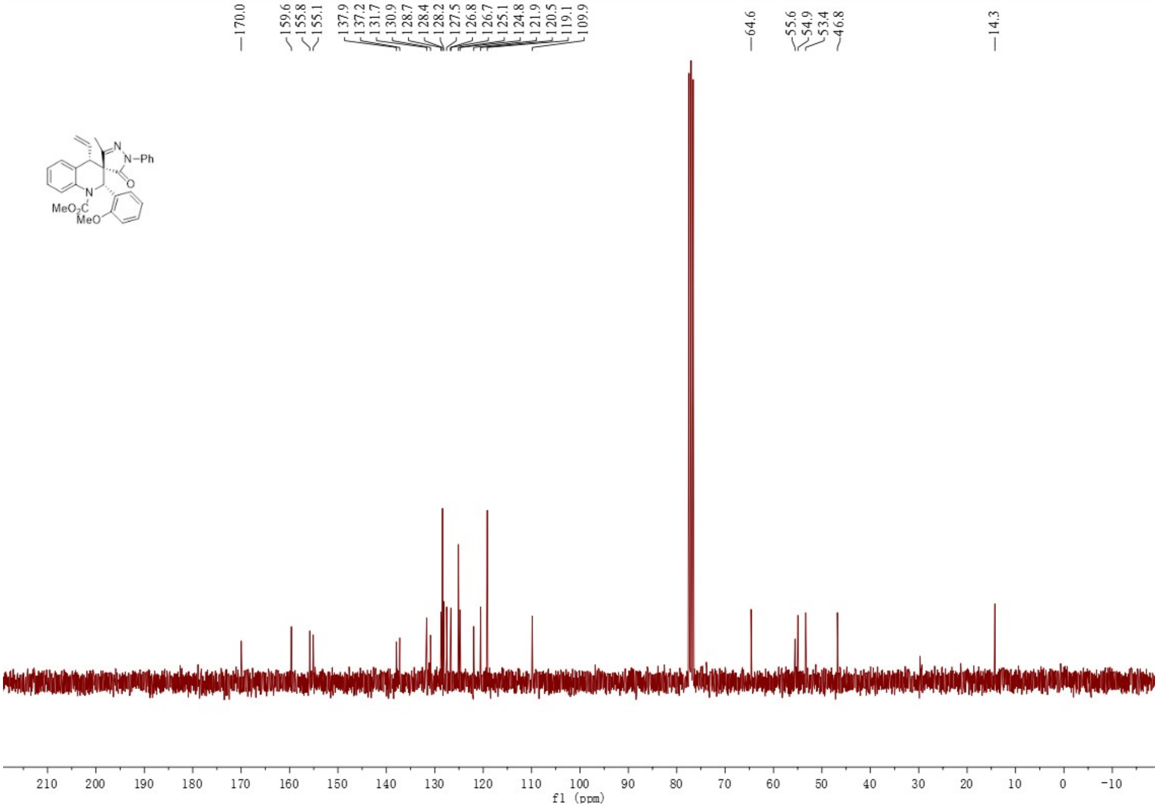

^{13}C ($CDCl_3$, 75 MHz) NMR of compound **4j**

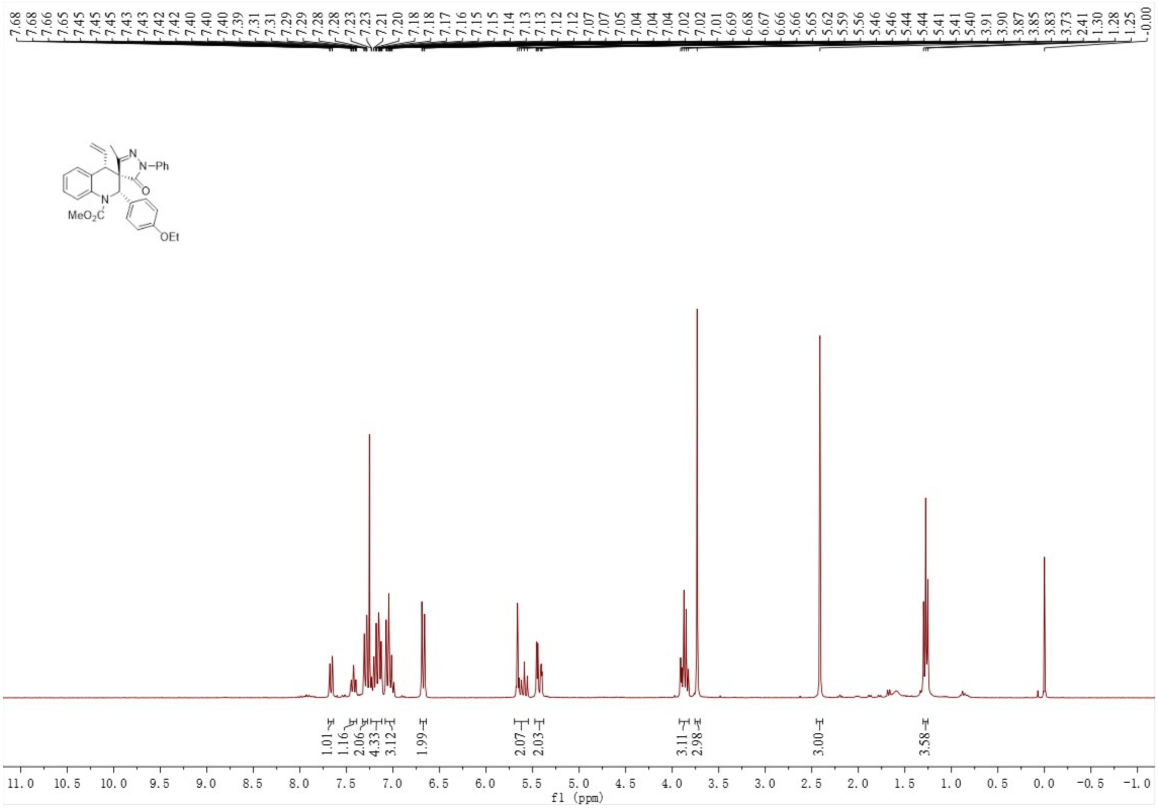

^{1}H ($CDCl_3$, 300 MHz) NMR of compound **4k**

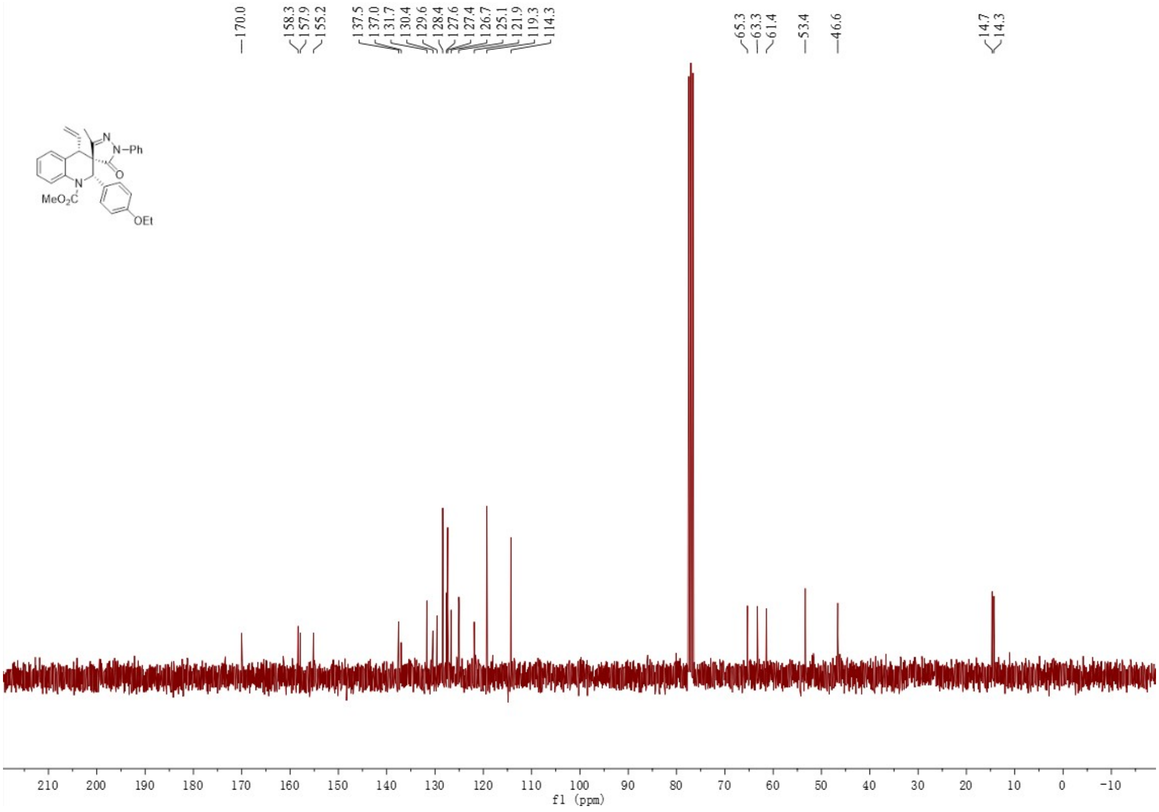

^{13}C ($CDCl_3$, 75 MHz) NMR of compound **4k**

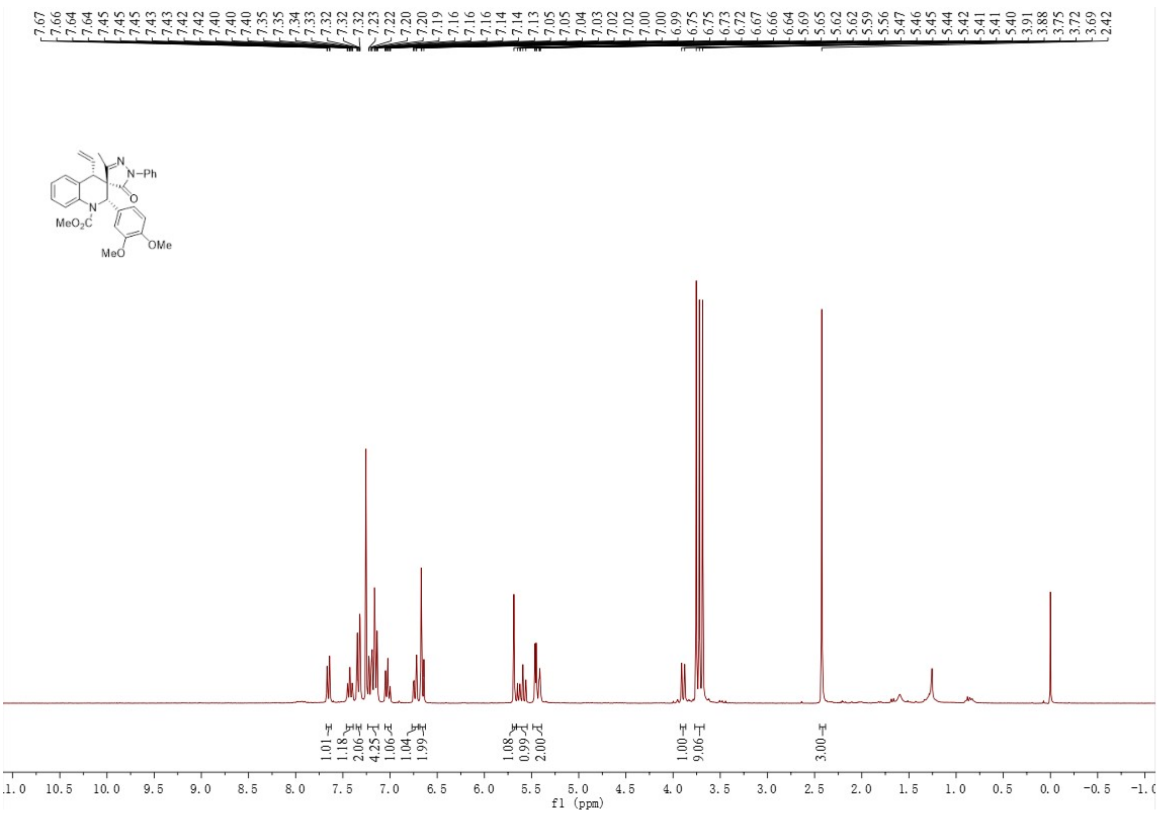

^{1}H ($CDCl_3$, 300 MHz) NMR of compound **4l**

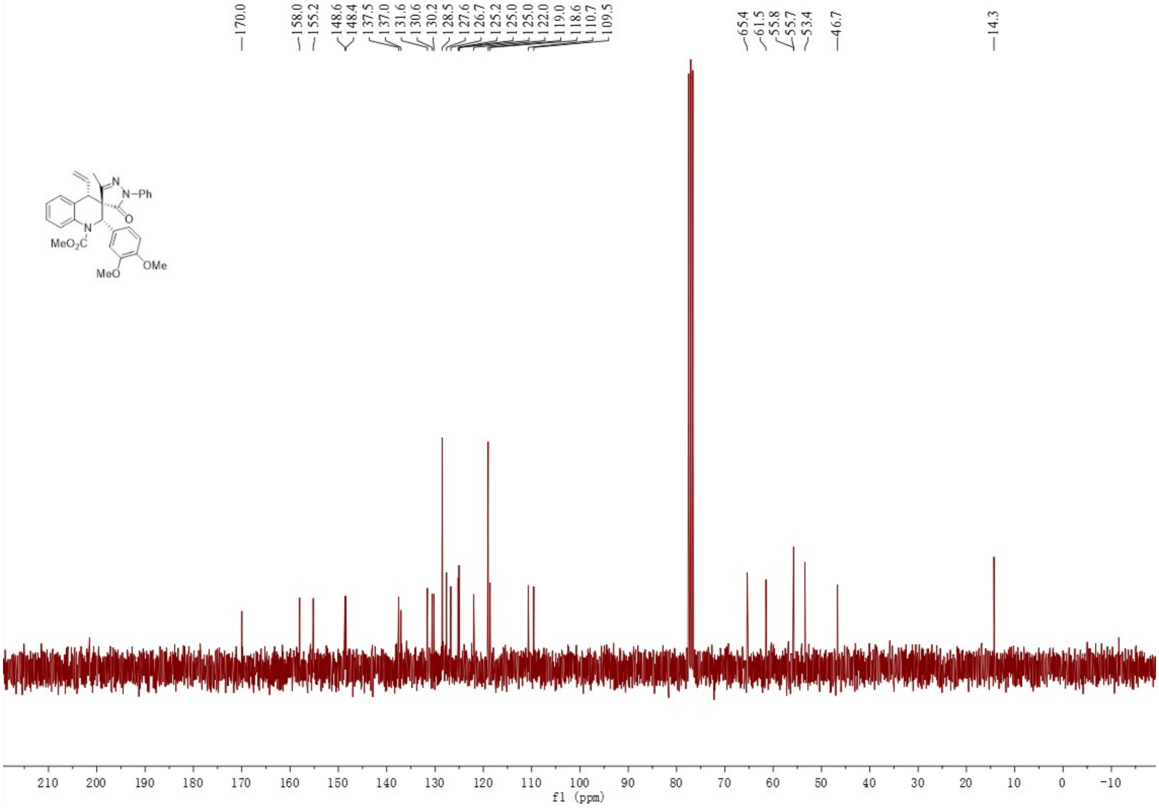

^{13}C ($CDCl_3$, 75 MHz) NMR of compound **4l**

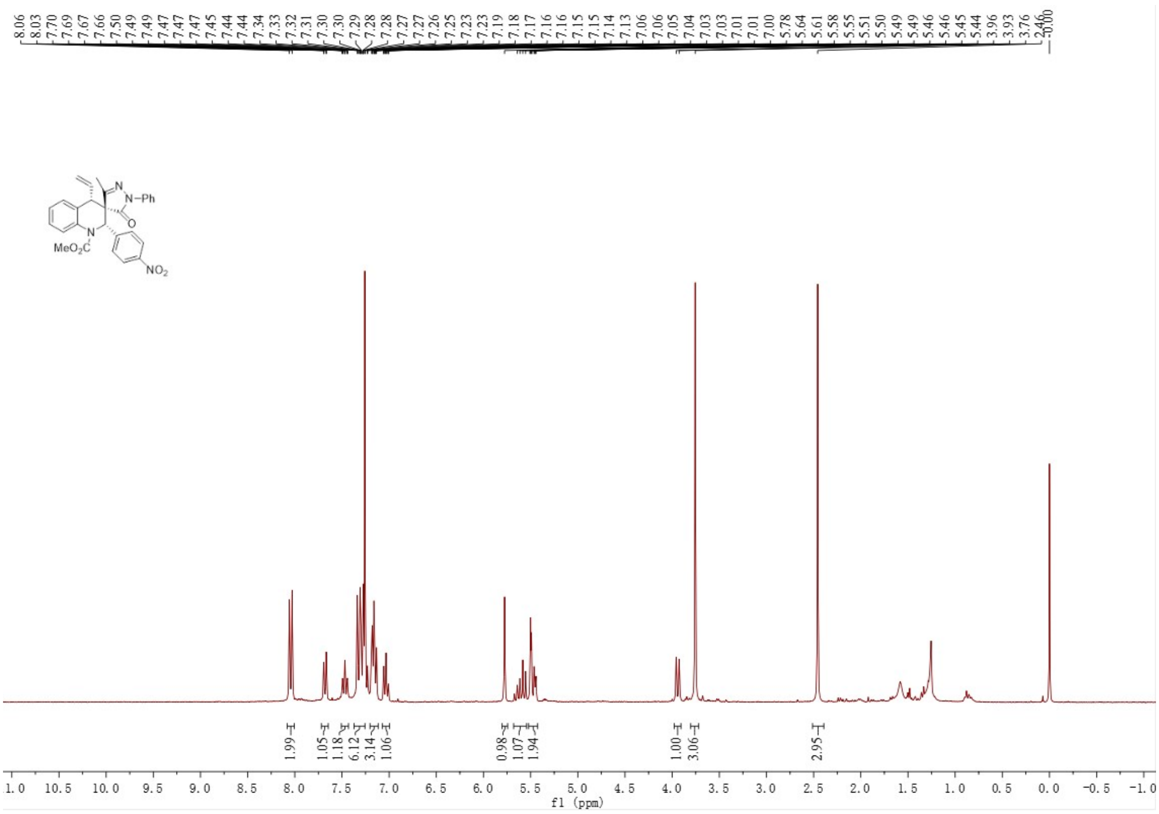

^{1}H ($CDCl_3$, 300 MHz) NMR of compound **4m**

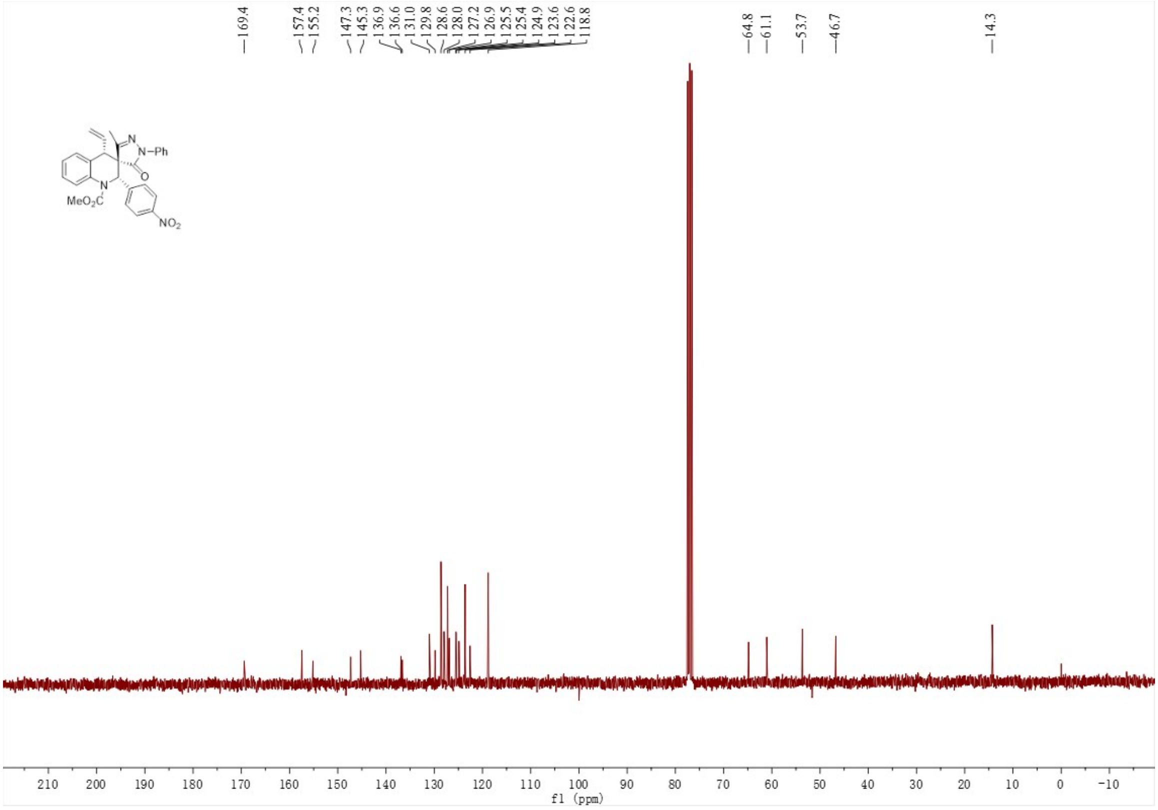

^{13}C ($CDCl_3$, 75 MHz) NMR of compound **4m**

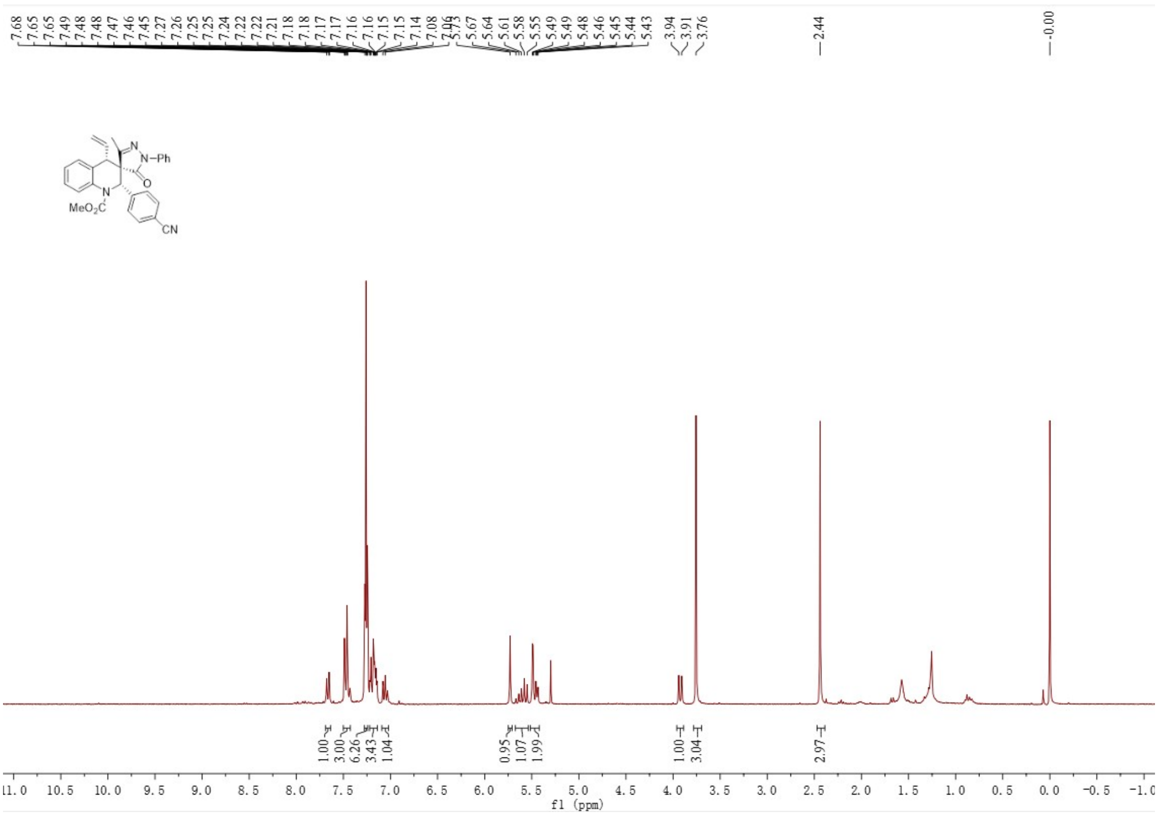

^{1}H (CDCl$_3$, 300 MHz) NMR of compound **4n**

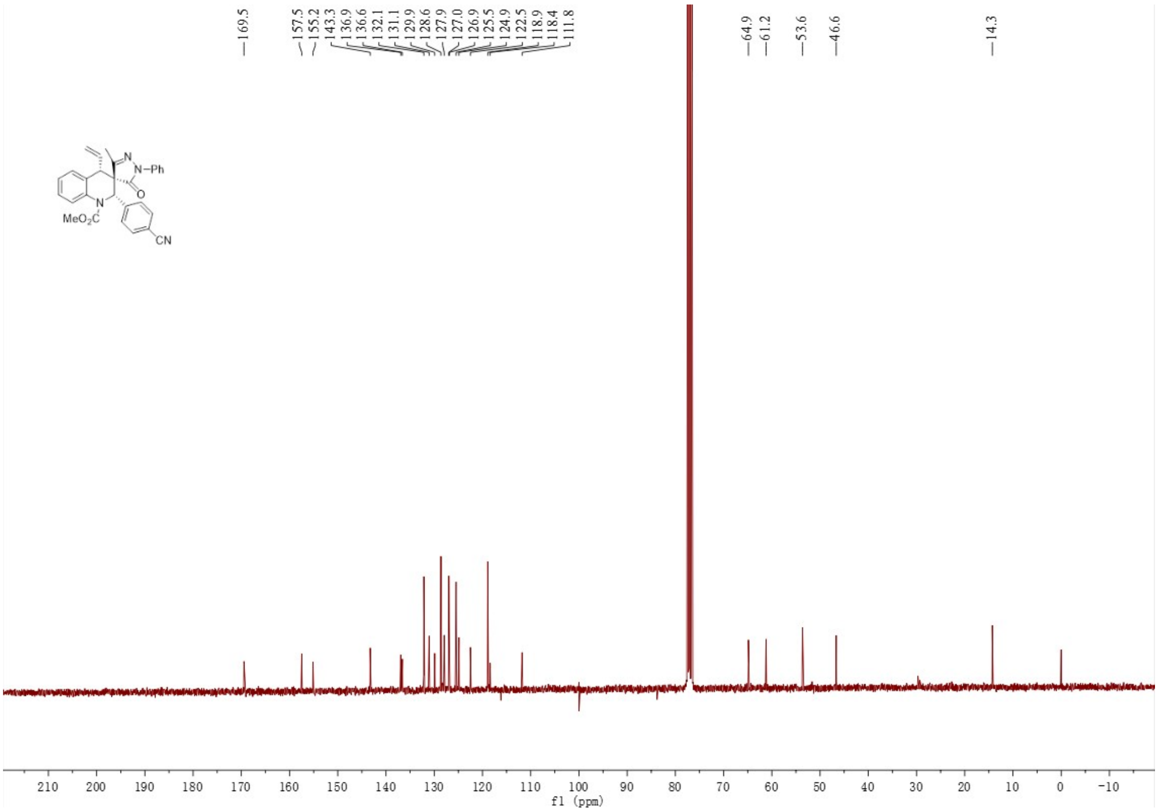

^{13}C (CDCl$_3$, 75 MHz) NMR of compound **4n**

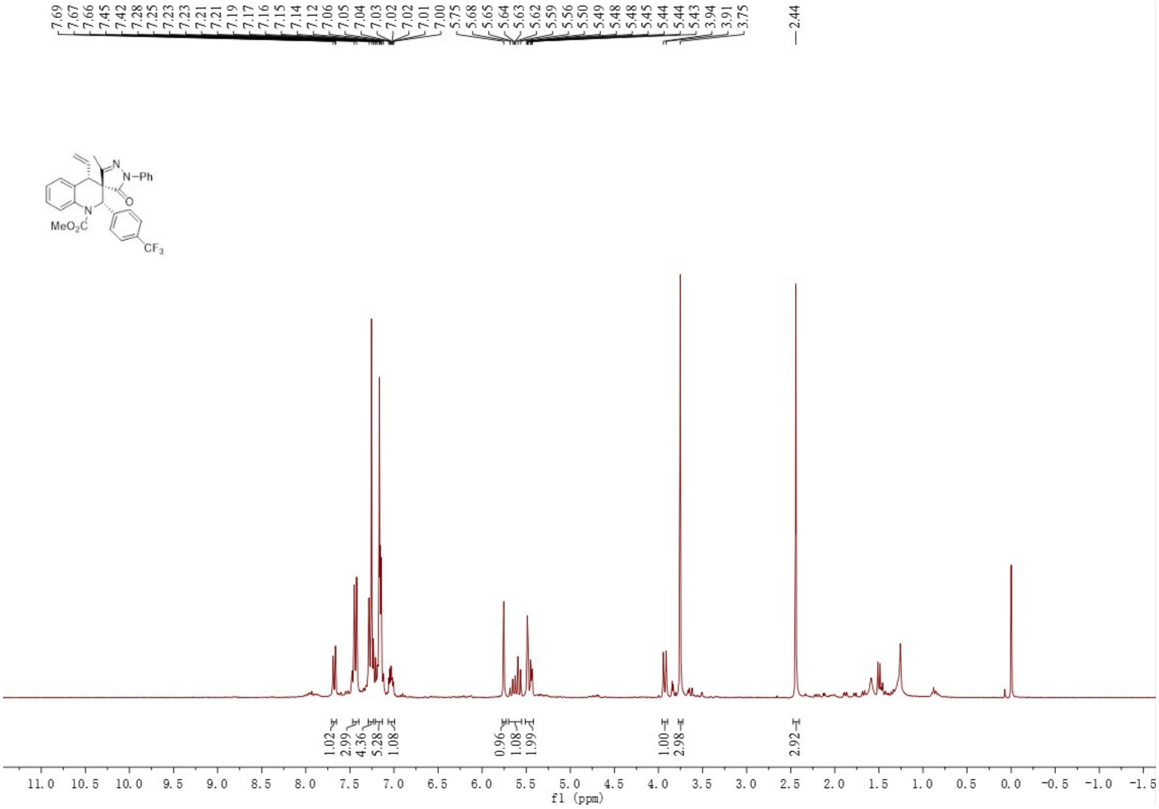

^{1}H ($CDCl_3$, 300 MHz) NMR of compound **4o**

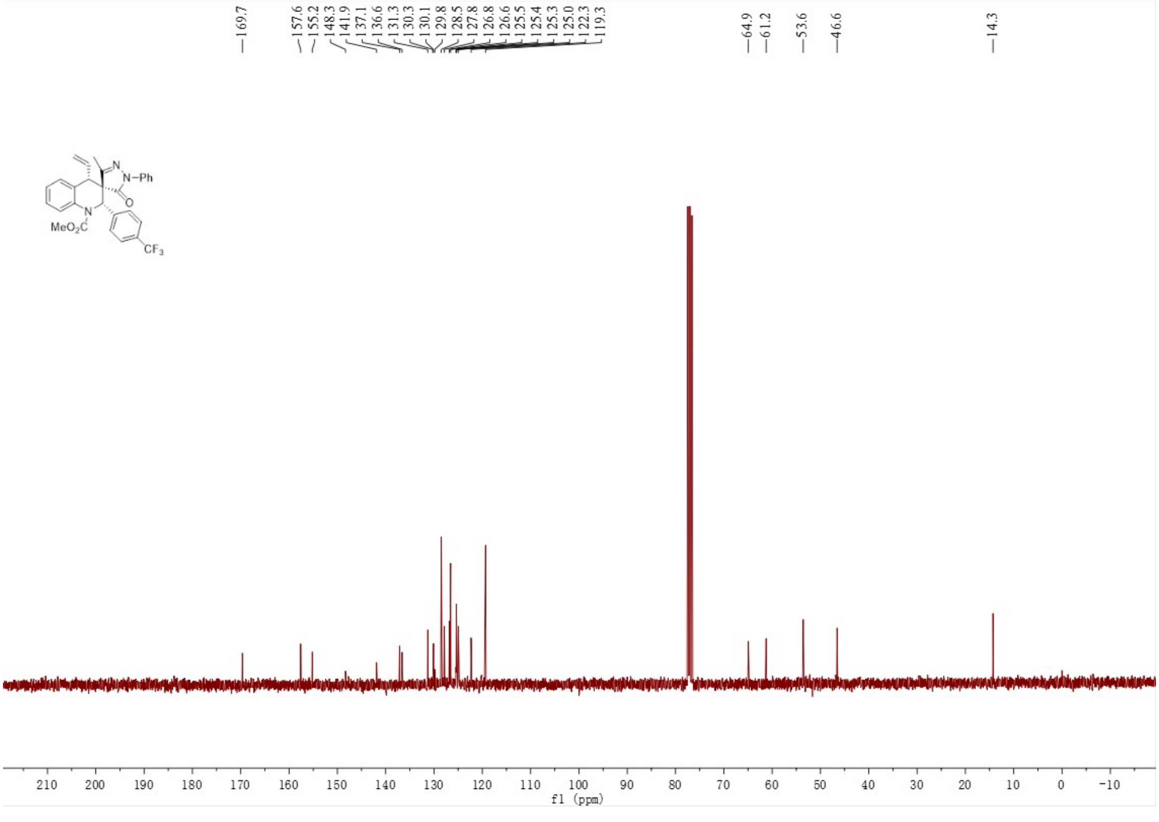

^{13}C ($CDCl_3$, 75 MHz) NMR of compound **4o**

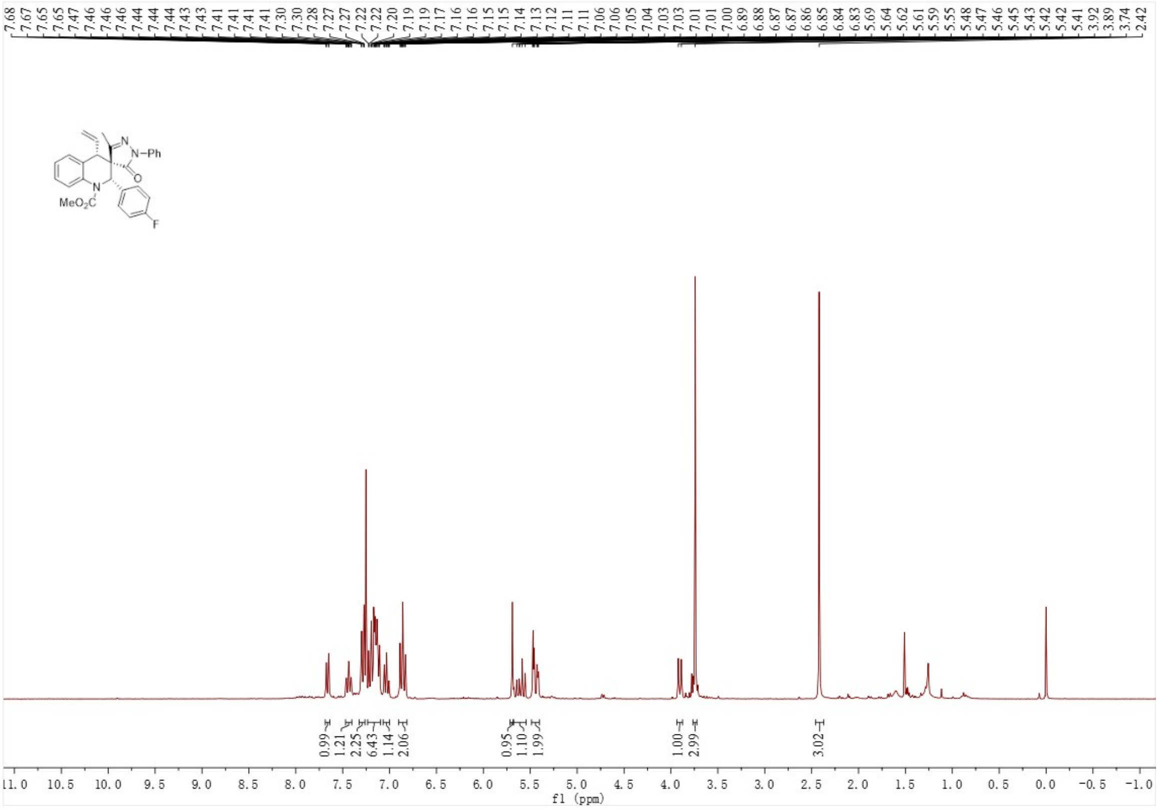

^{1}H ($CDCl_3$, 300 MHz) NMR of compound **4p**

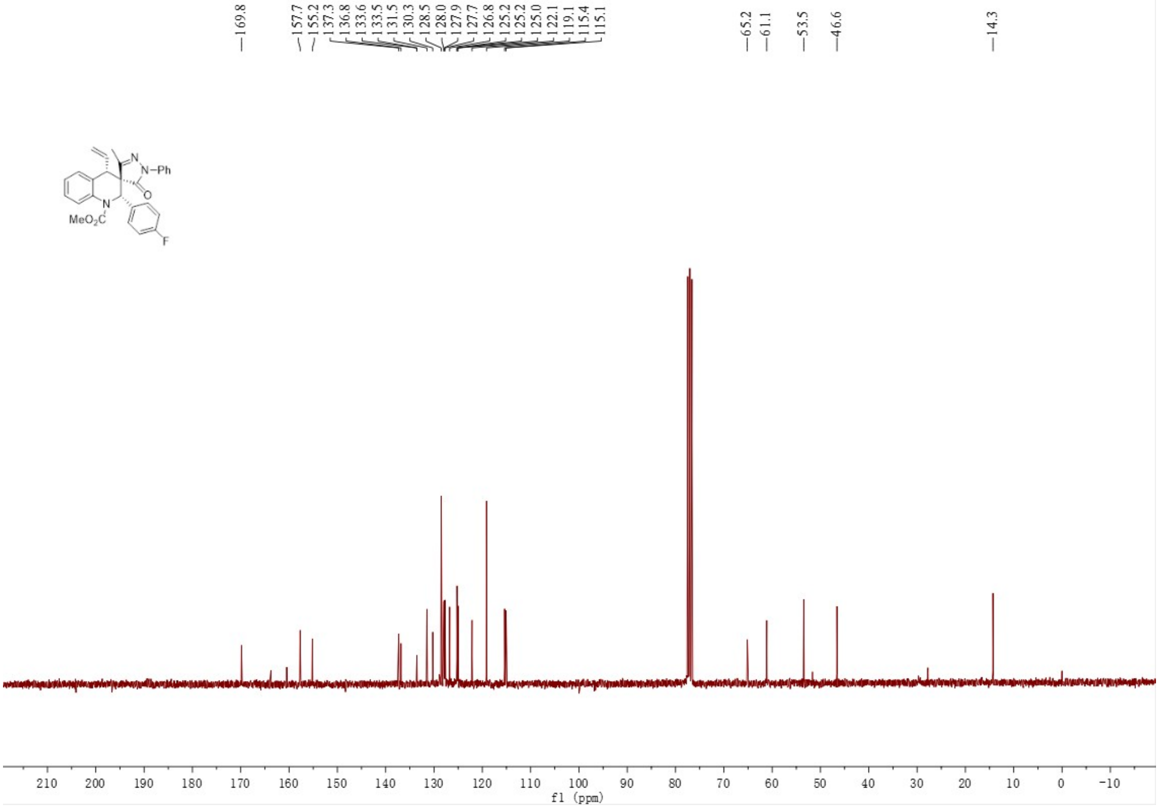

^{13}C ($CDCl_3$, 75 MHz) NMR of compound **4p**

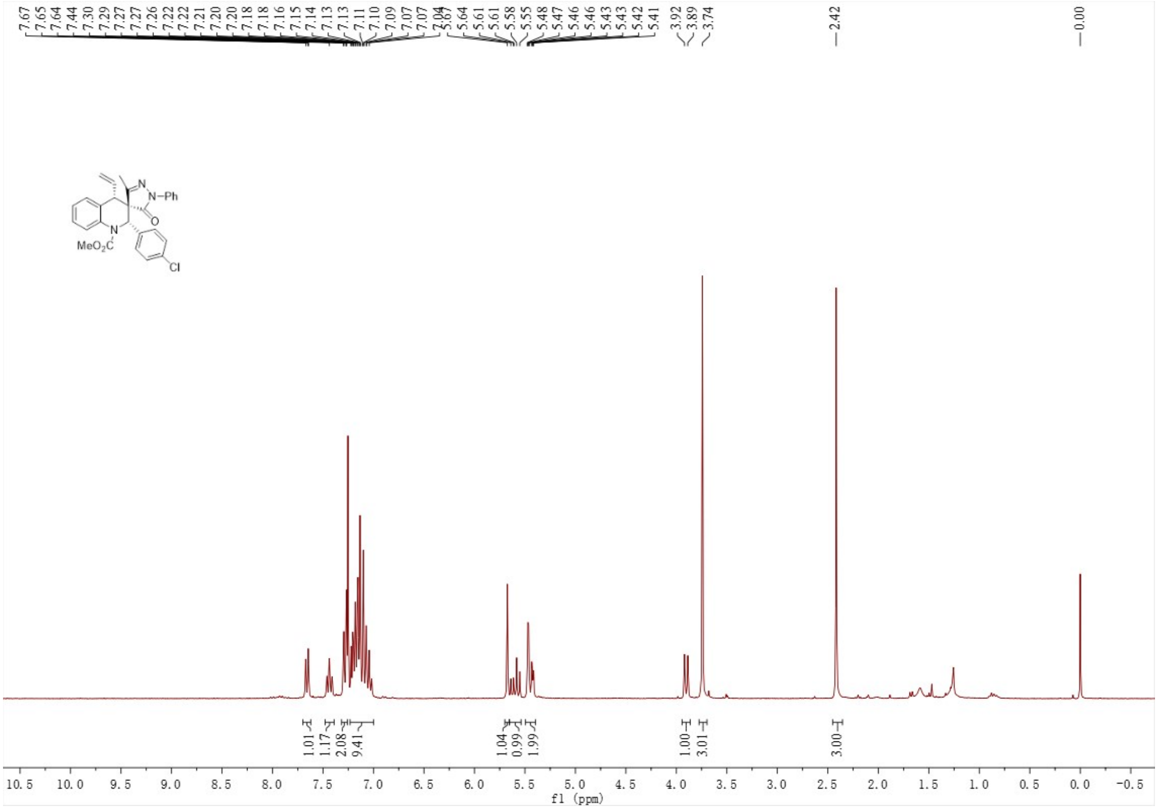

^{1}H ($CDCl_3$, 300 MHz) NMR of compound **4q**

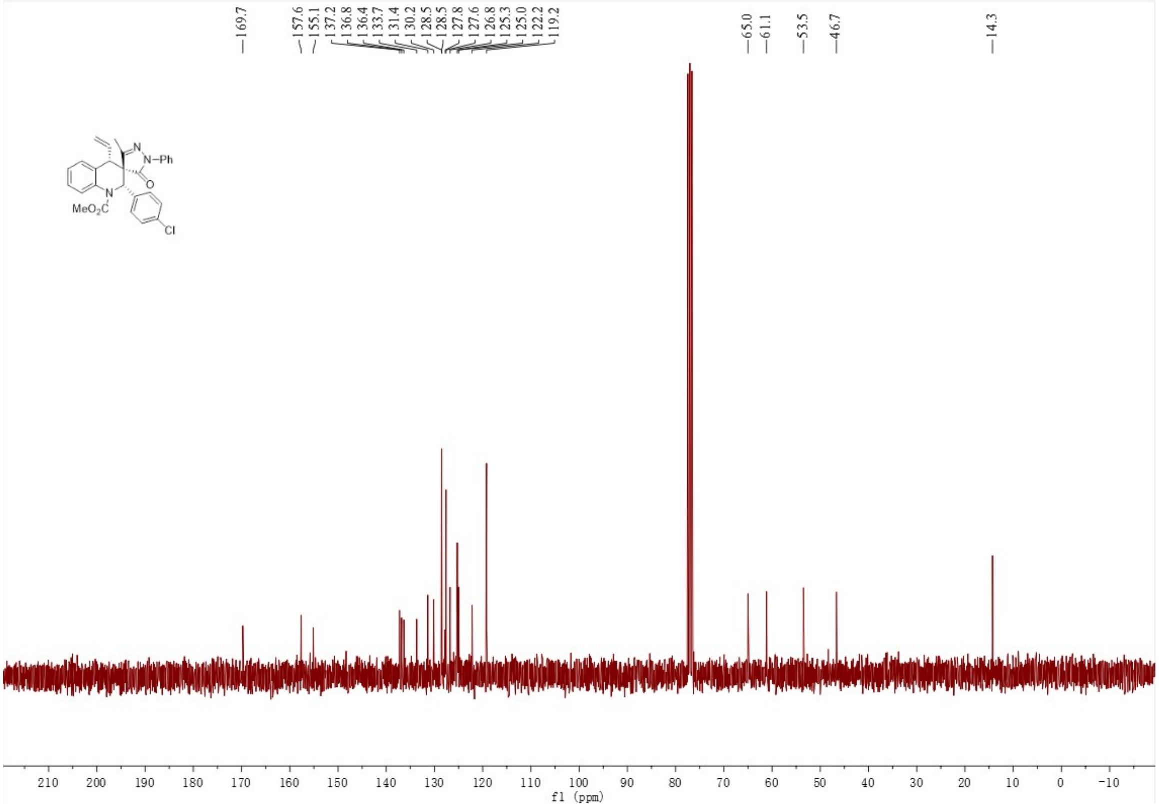

^{13}C ($CDCl_3$, 75 MHz) NMR of compound **4q**

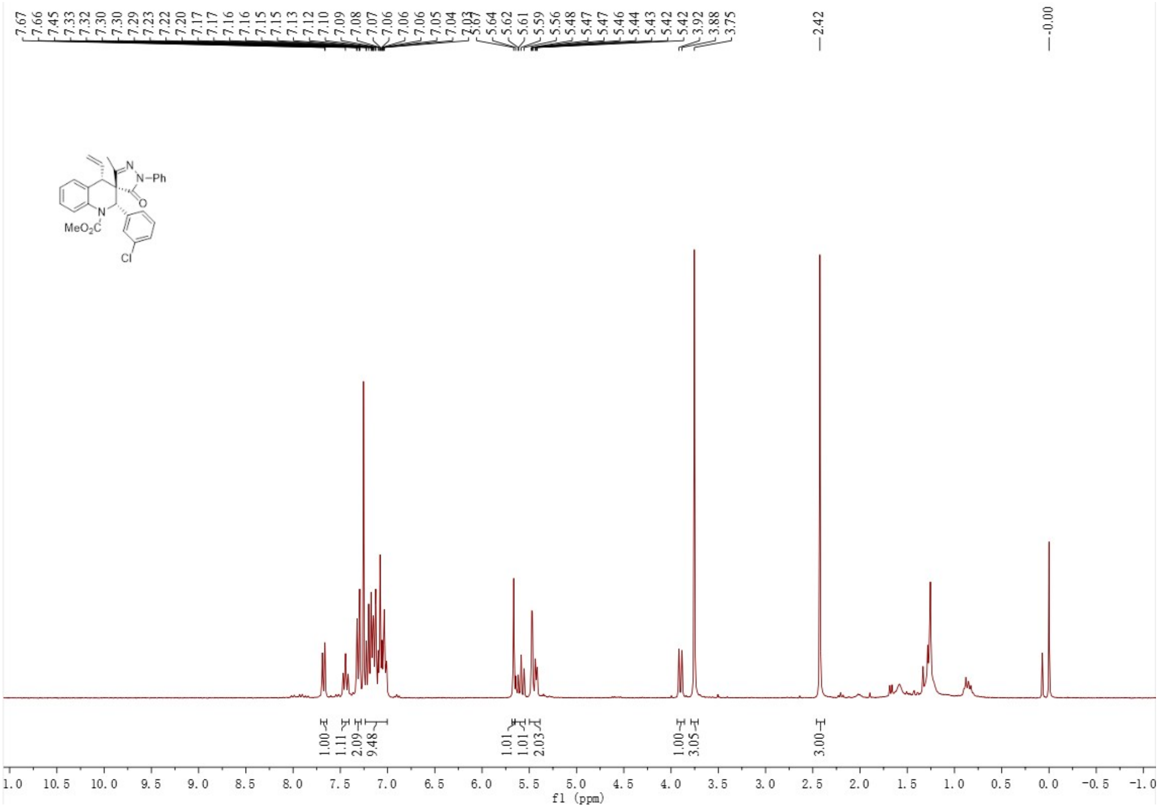

^{1}H ($CDCl_3$, 300 MHz) NMR of compound **4r**

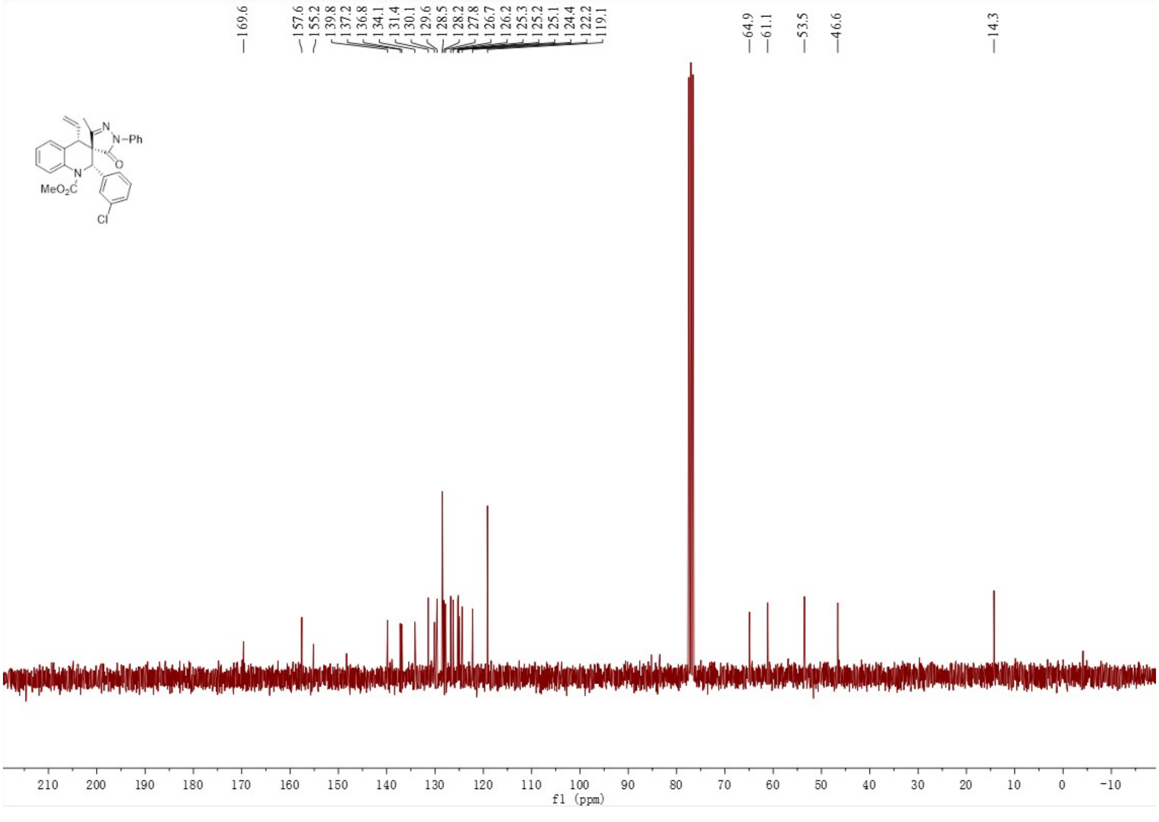

^{13}C ($CDCl_3$, 75 MHz) NMR of compound **4r**

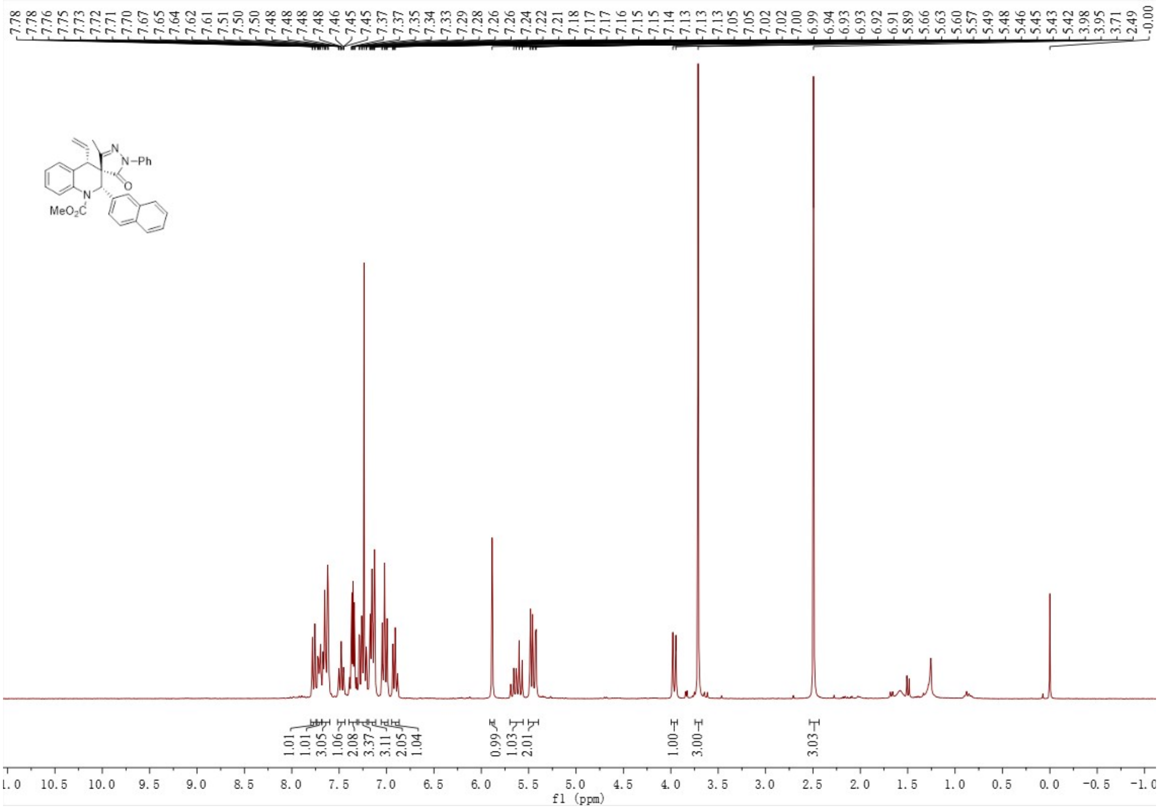

^{1}H ($CDCl_3$, 300 MHz) NMR of compound **4s**

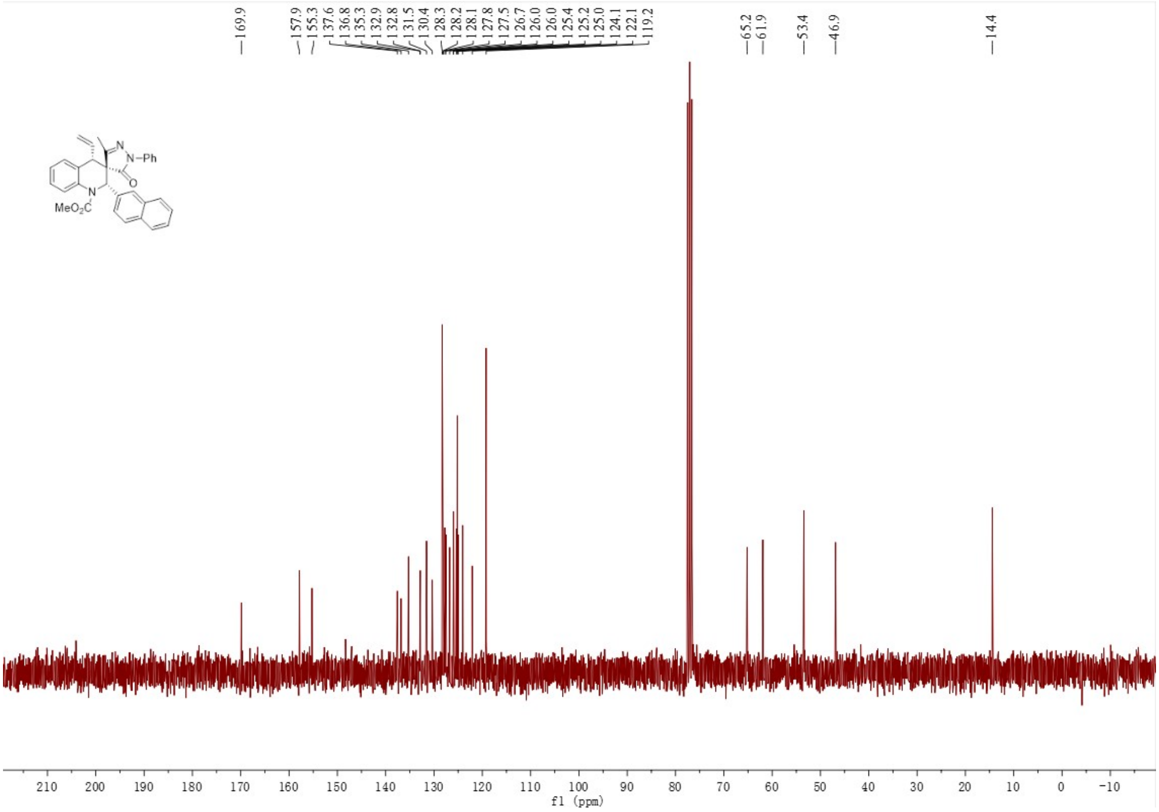

^{13}C ($CDCl_3$, 75 MHz) NMR of compound **4s**

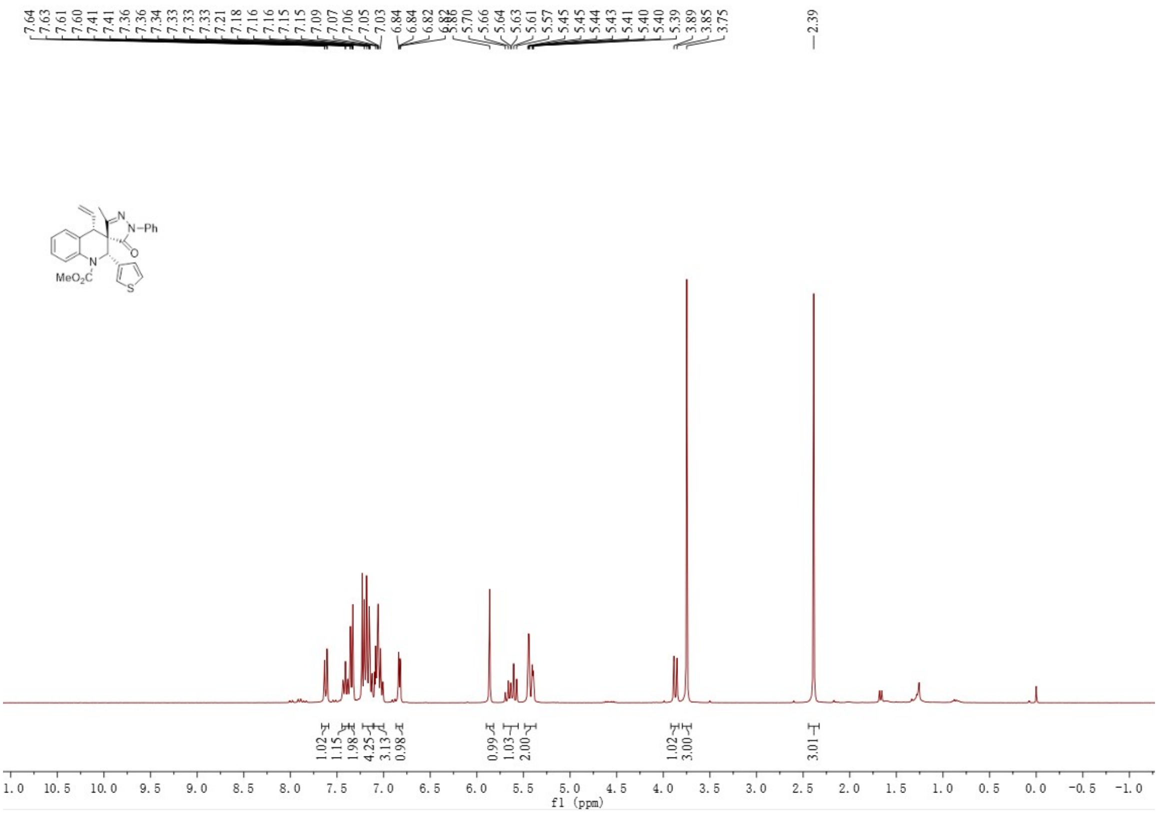

^{1}H ($CDCl_3$, 300 MHz) NMR of compound **4t**

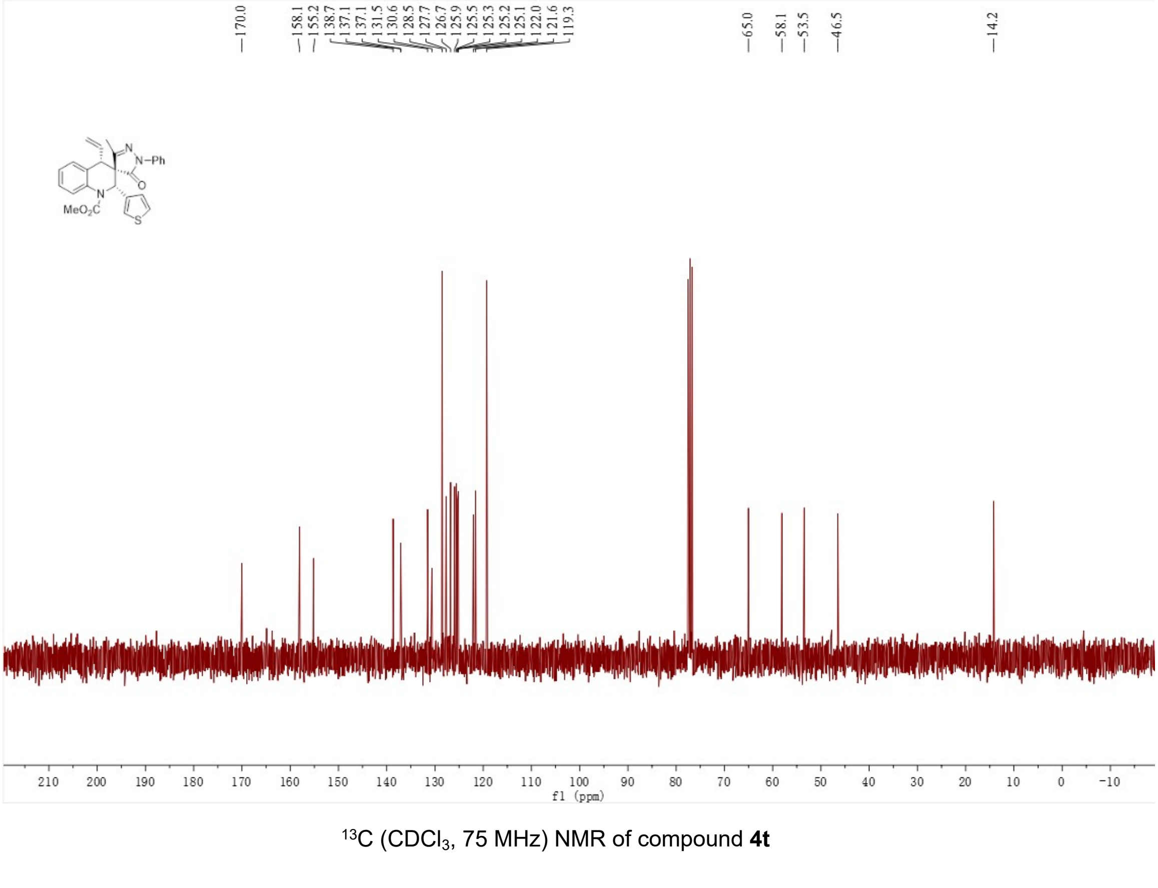

^{13}C ($CDCl_3$, 75 MHz) NMR of compound **4t**

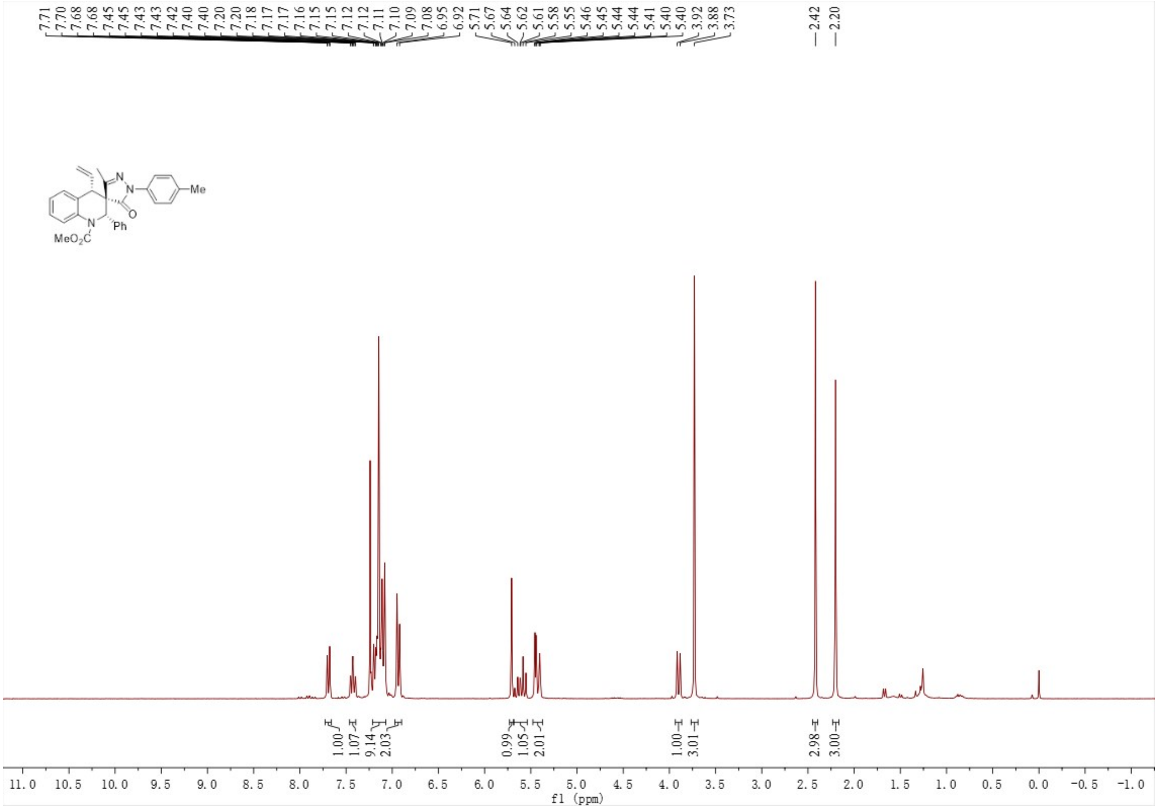

^{1}H ($CDCl_3$, 300 MHz) NMR of compound **4u**

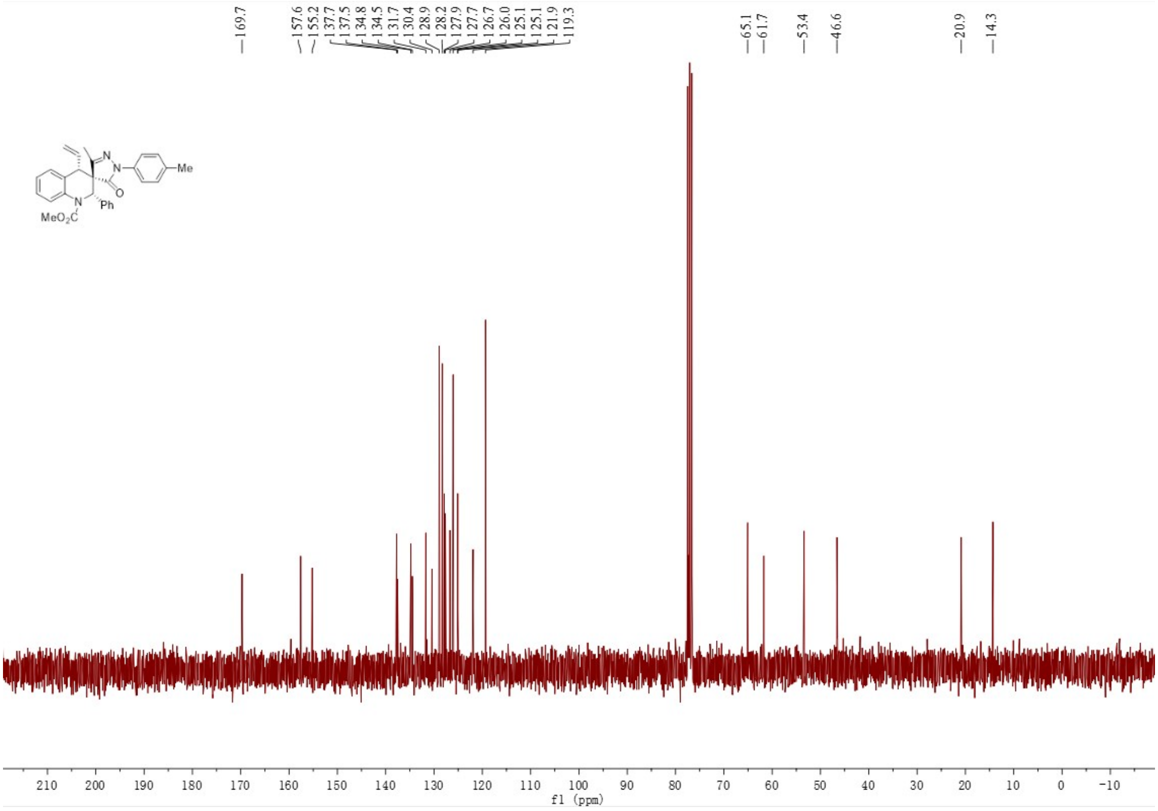

^{13}C ($CDCl_3$, 75 MHz) NMR of compound **4u**

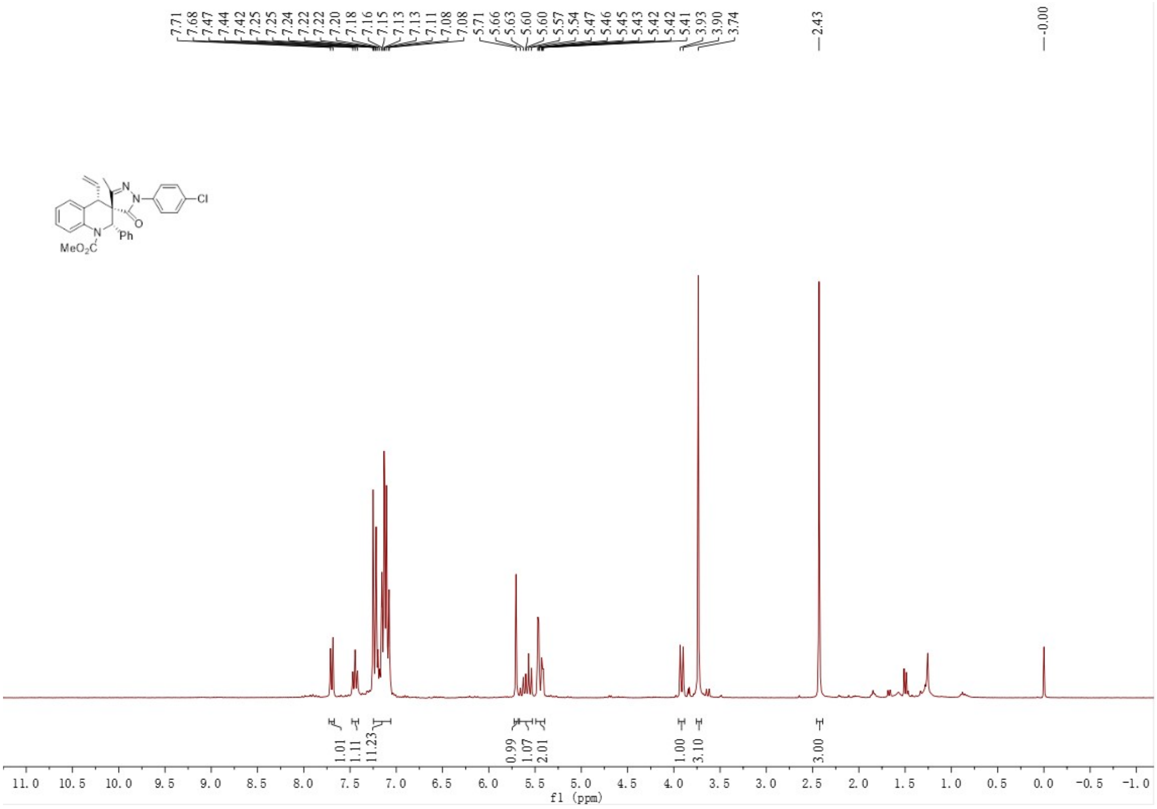

^{1}H (CDCl$_3$, 300 MHz) NMR of compound **4v**

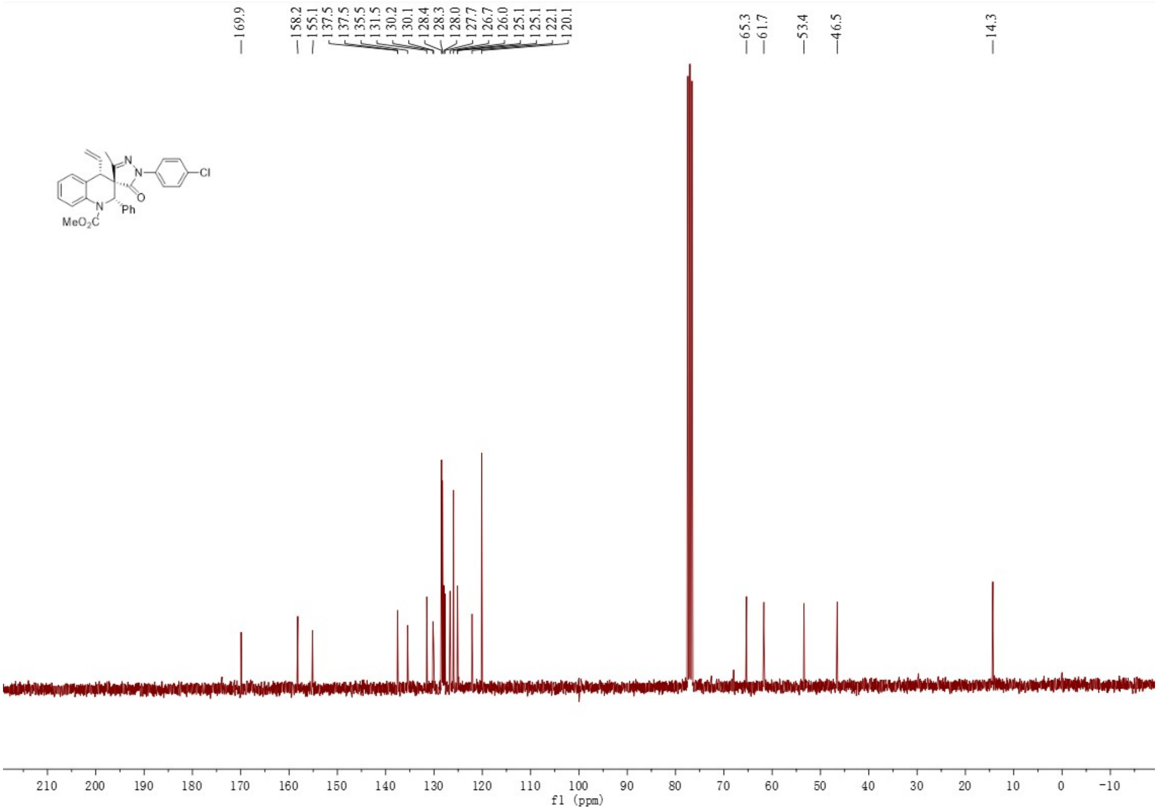

^{13}C (CDCl$_3$, 75 MHz) NMR of compound **4v**

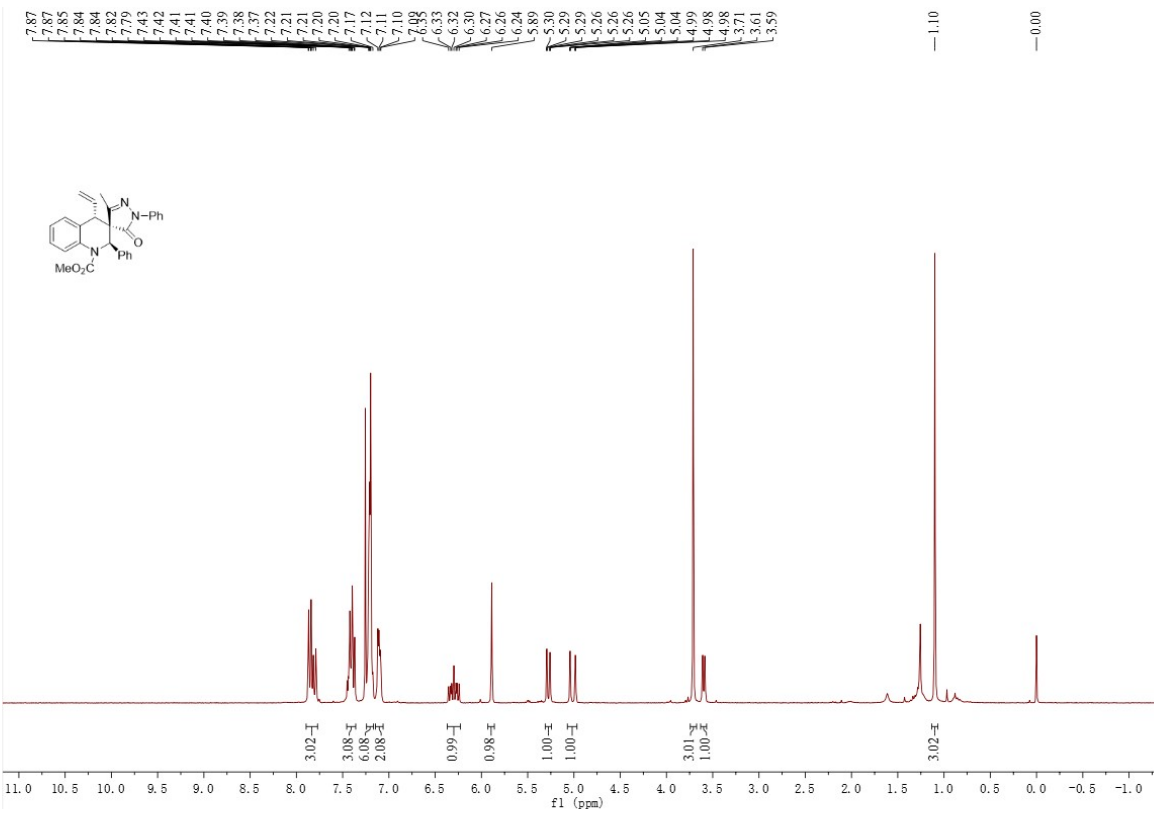

^1H ($CDCl_3$, 300 MHz) NMR of compound **5a**

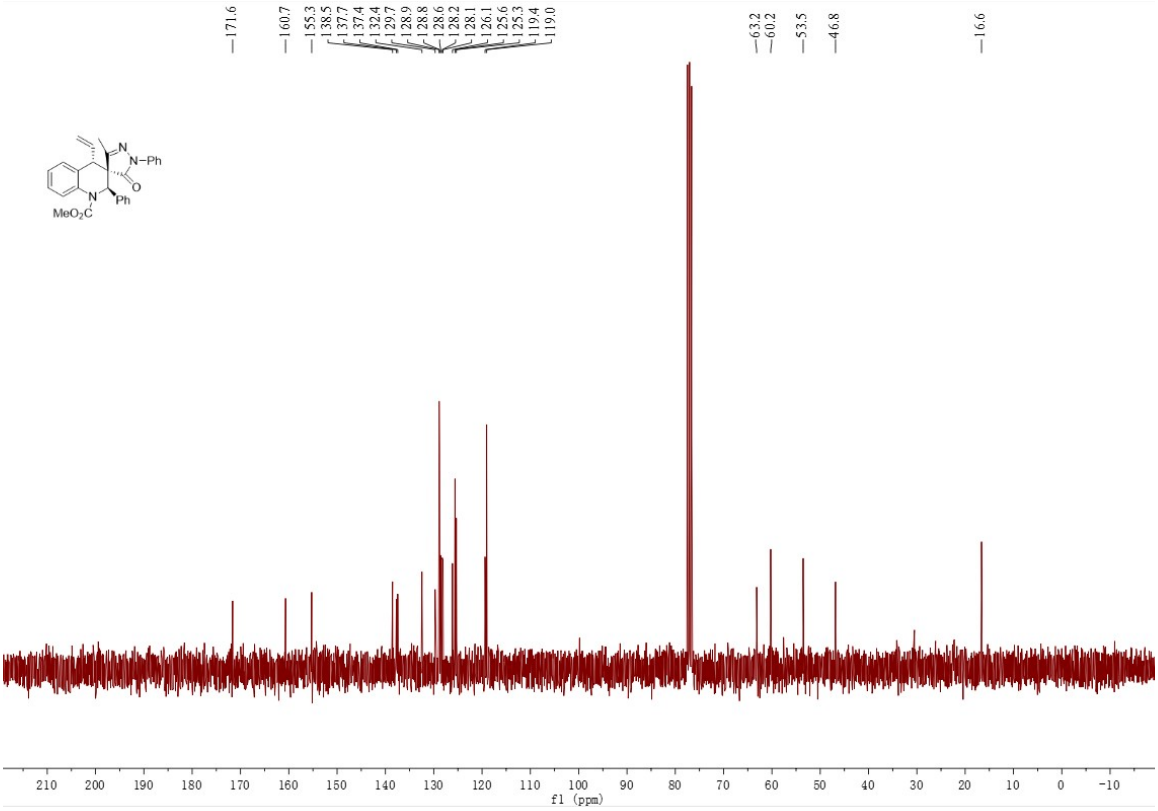

^{13}C ($CDCl_3$, 75 MHz) NMR of compound **5a**

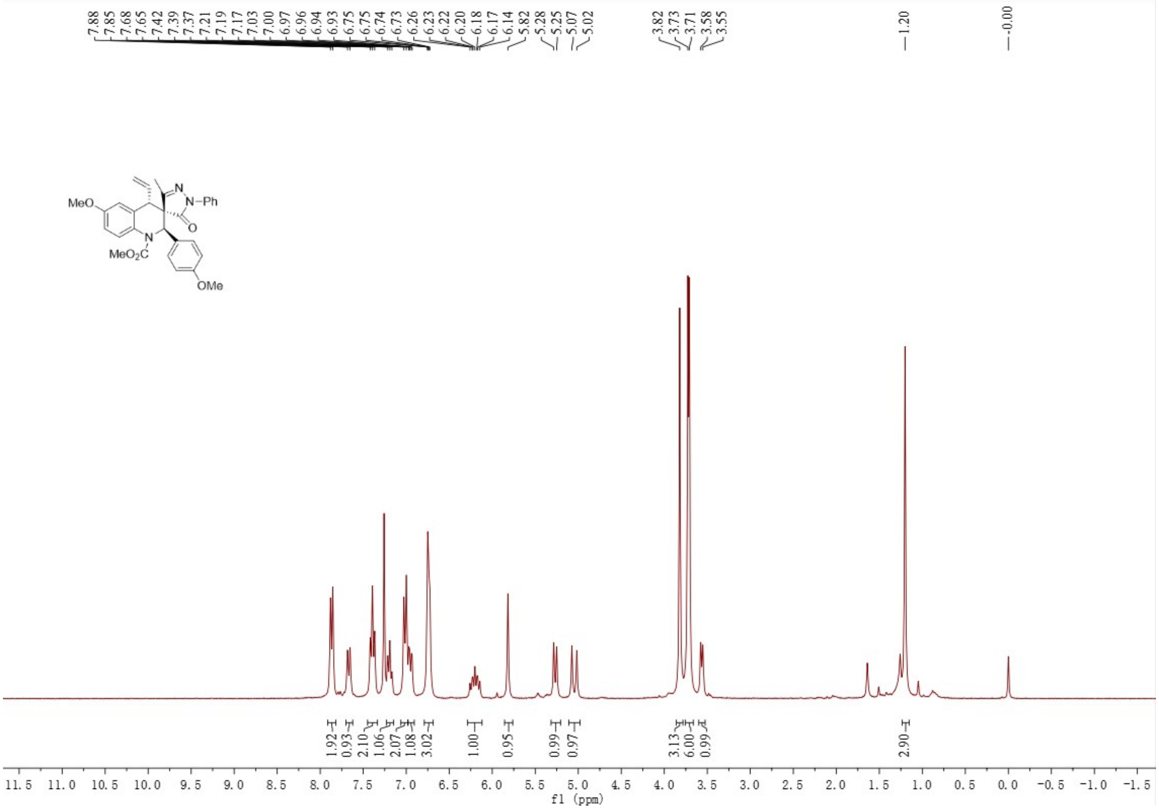

^1H ($CDCl_3$, 300 MHz) NMR of compound **5b**

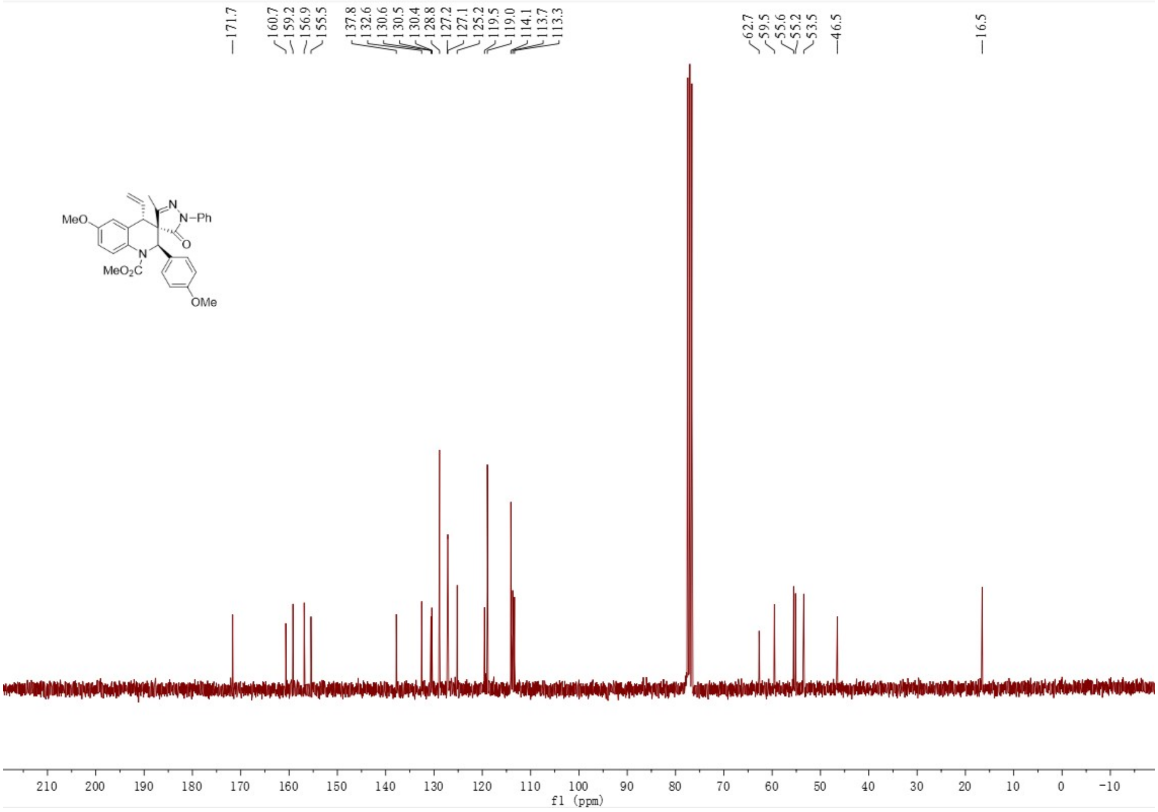

^{13}C ($CDCl_3$, 75 MHz) NMR of compound **5b**

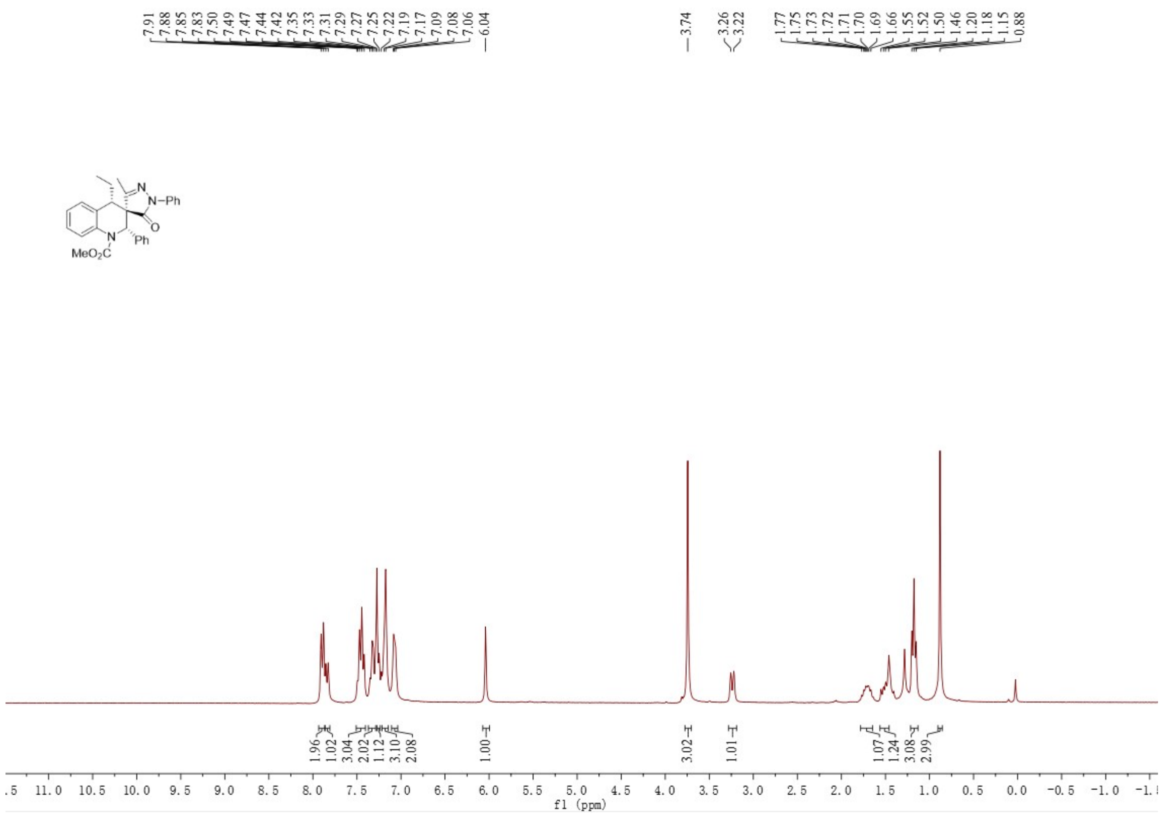

^{1}H ($CDCl_3$, 300 MHz) NMR of compound **6**

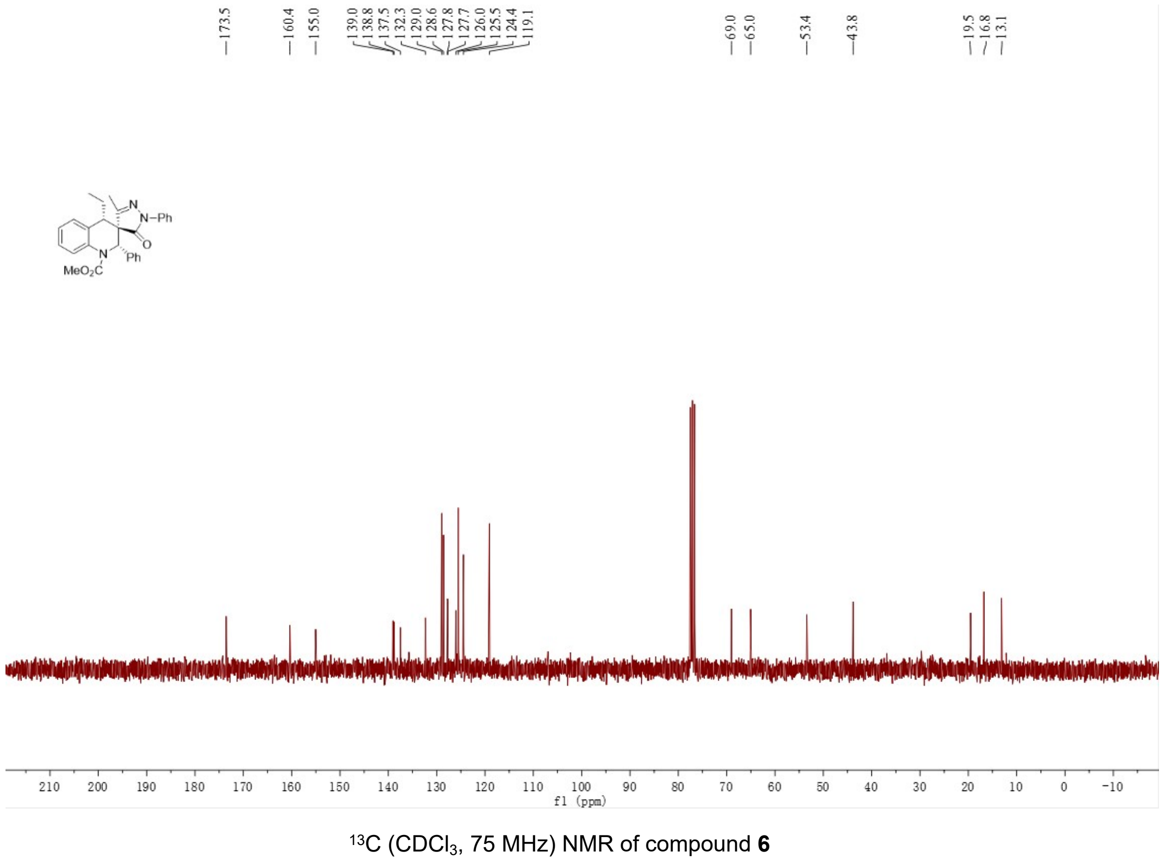

^{13}C ($CDCl_3$, 75 MHz) NMR of compound **6**

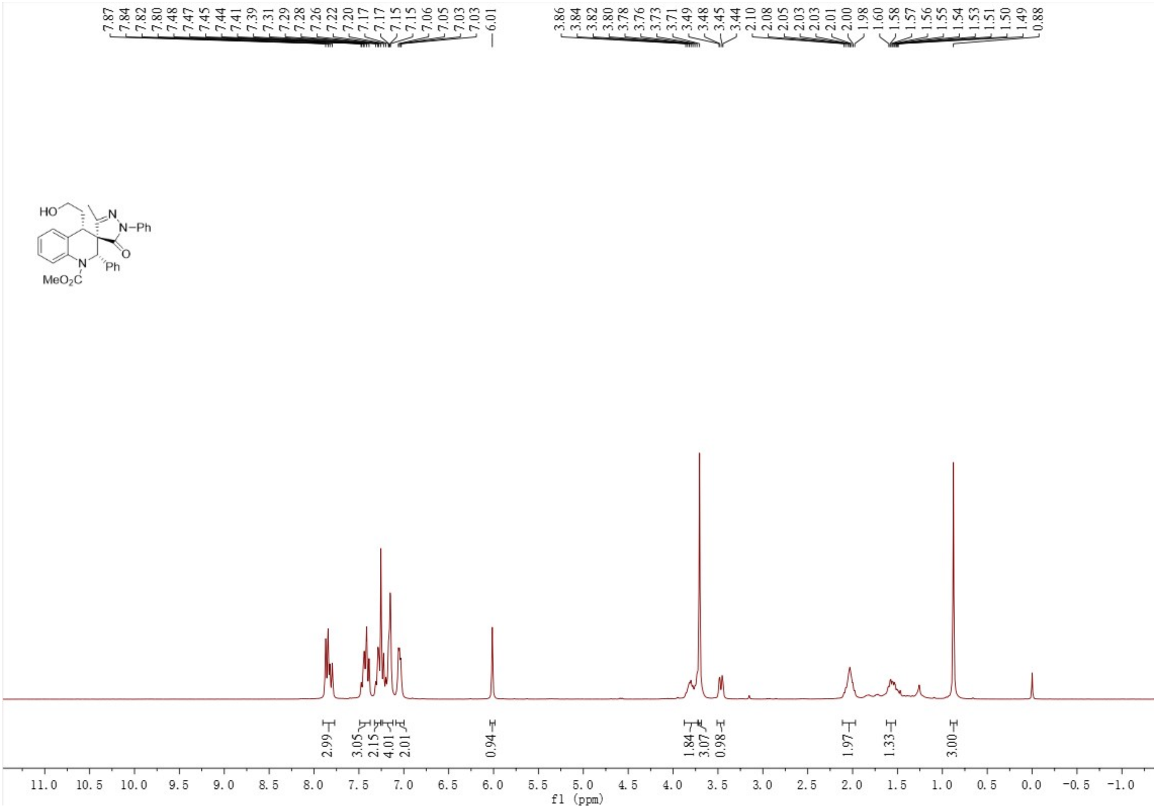

^{1}H (CDCl$_3$, 300 MHz) NMR of compound **7**

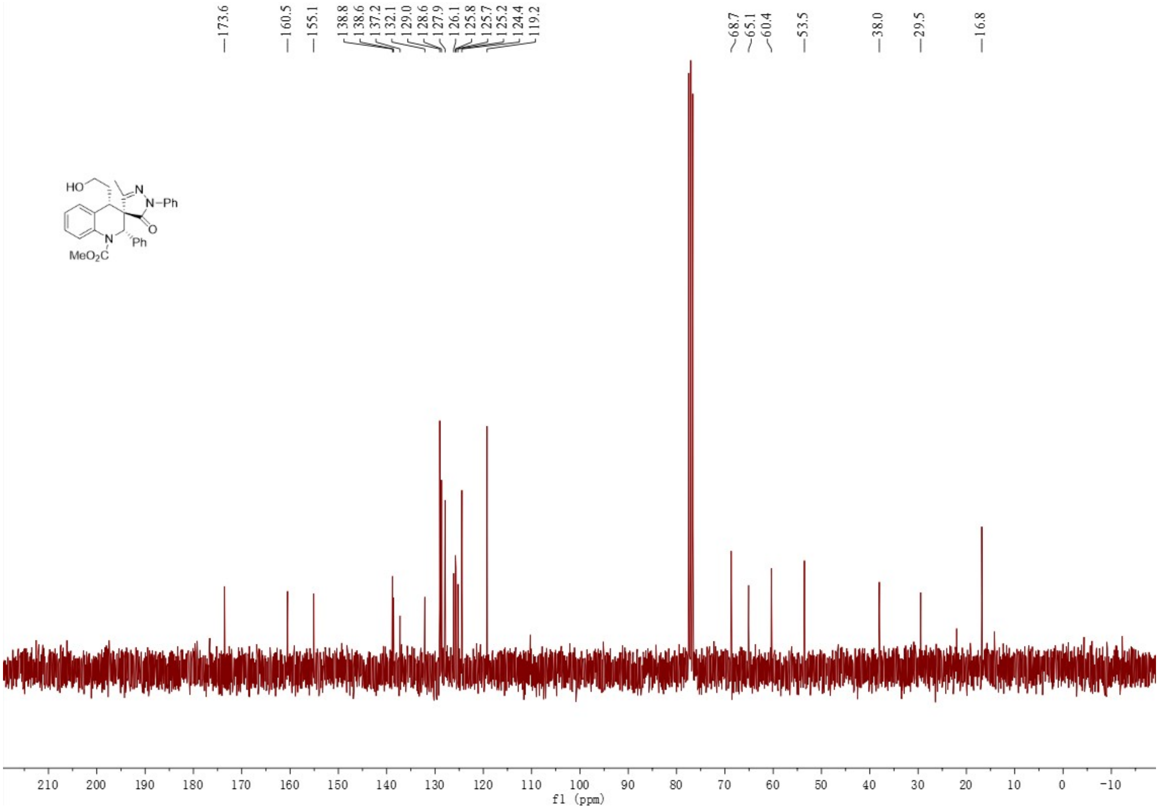

^{13}C (CDCl$_3$, 75 MHz) NMR of compound **7**

6. Copies of HPLC Chromatograms

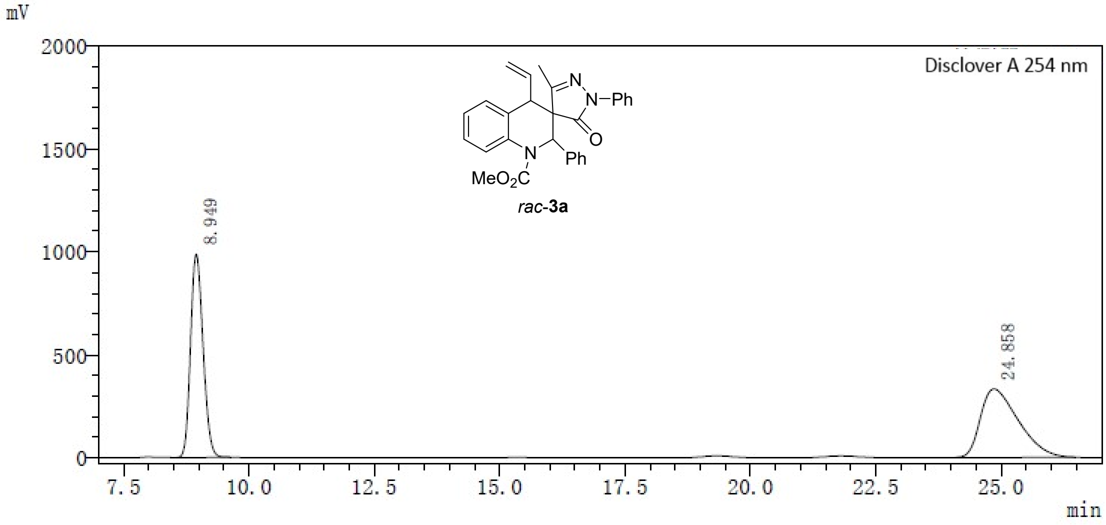

Discloser A 254 nm

Peak	Reten time (min)	Area(%)
1	8.949	49.475
2	24.858	50.525
total		100

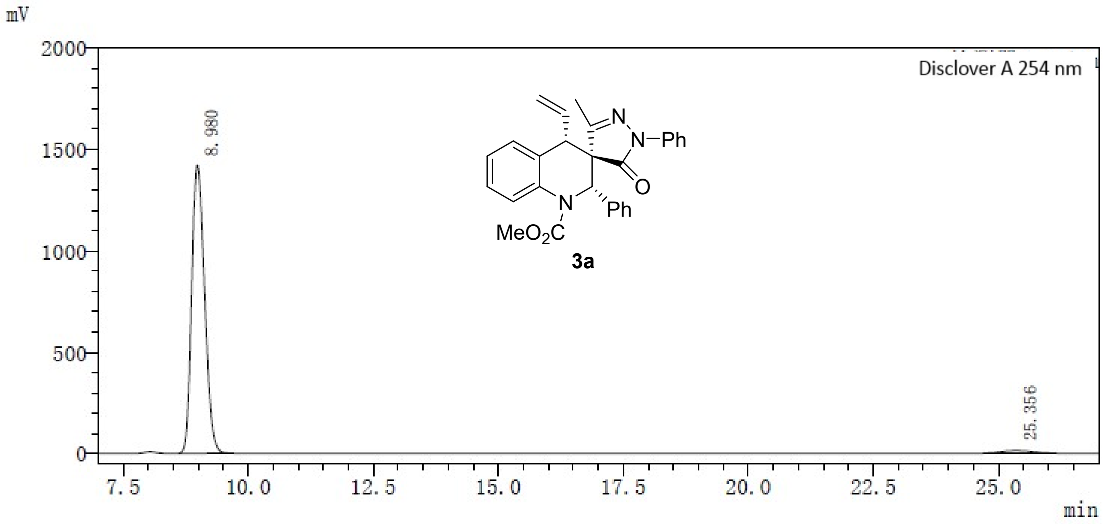

Discloser A 254 nm

Peak	Reten time (min)	Area(%)
1	8.980	97.848
2	25.356	2.152
total		100

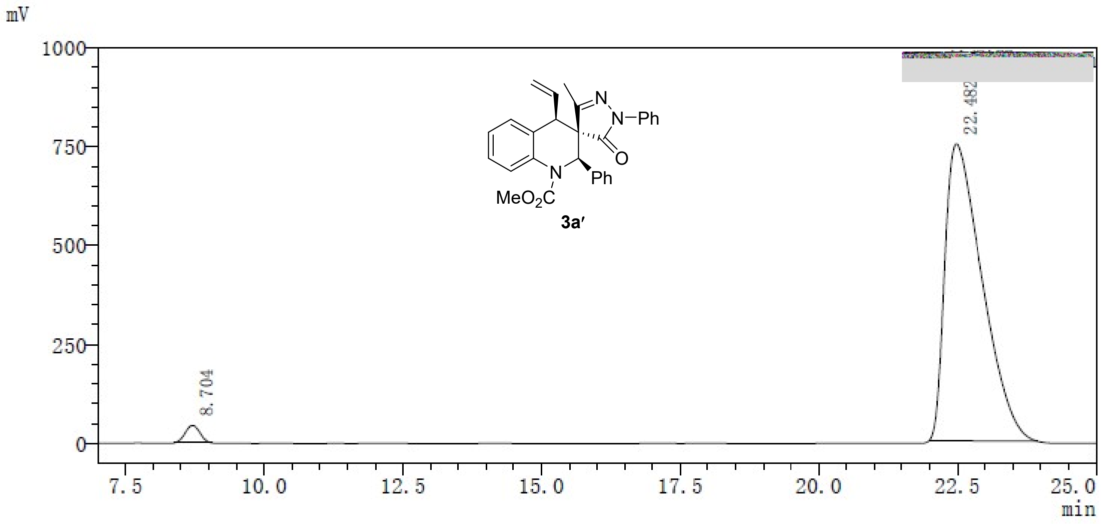

Discloser A 254 nm

Peak	Reten time (min)	Area(%)
1	8.704	2.254
2	22.482	97.746
total		100

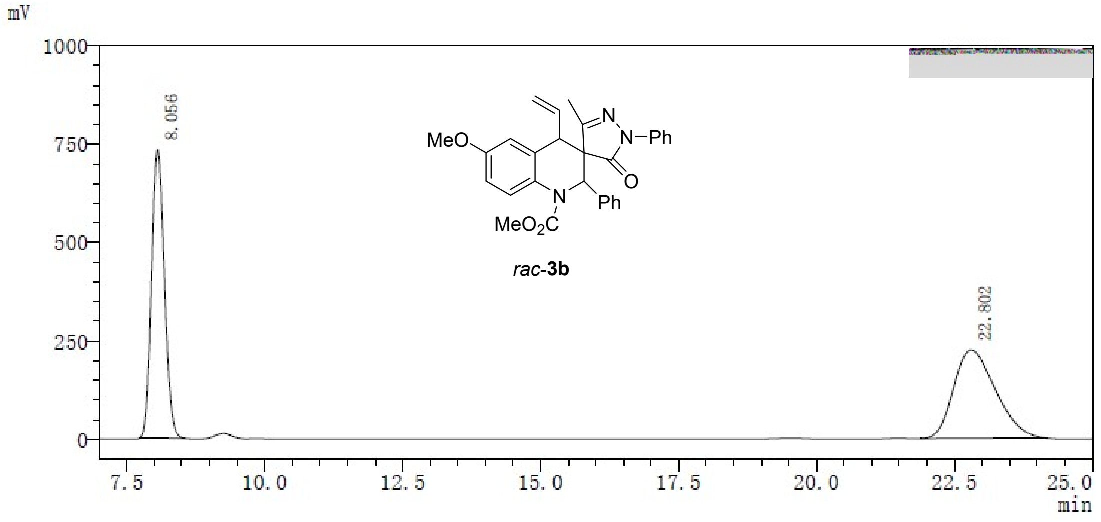

Discloser A 254 nm

Peak	Reten time (min)	Area(%)
1	8.056	49.911
2	22.802	50.089
total		100

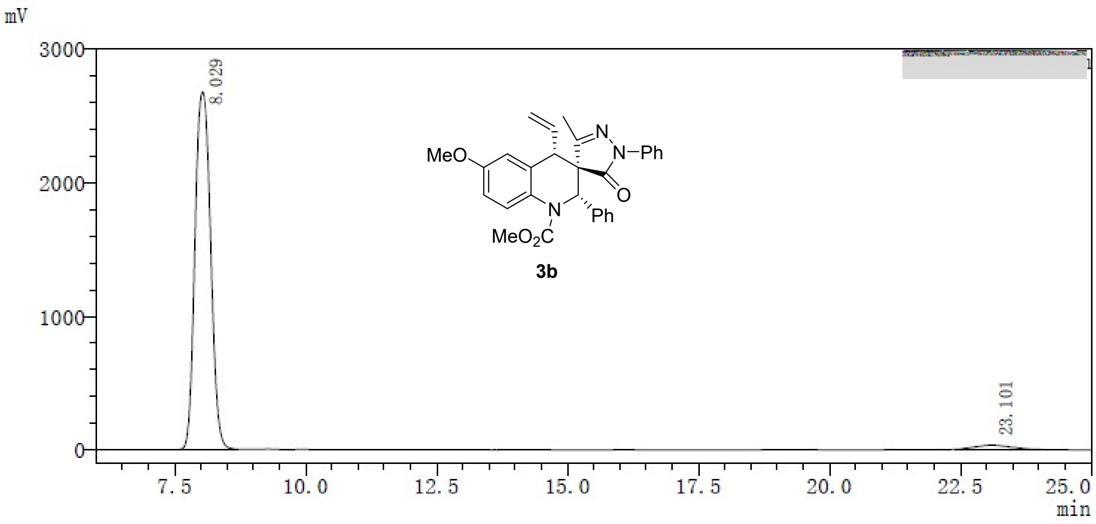

Discloser A 254 nm

Peak	Reten time (min)	Area(%)
1	8.029	97.446
2	23.101	2.554
total		100

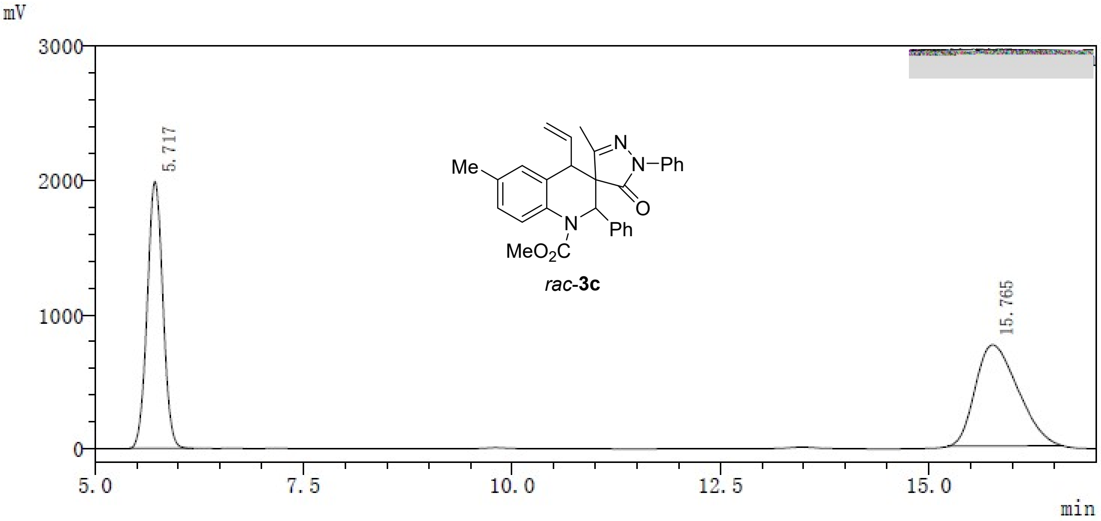

Discloser A 254 nm

Peak	Reten time (min)	Area(%)
1	5.717	49.063
2	15.765	50.937
total		100

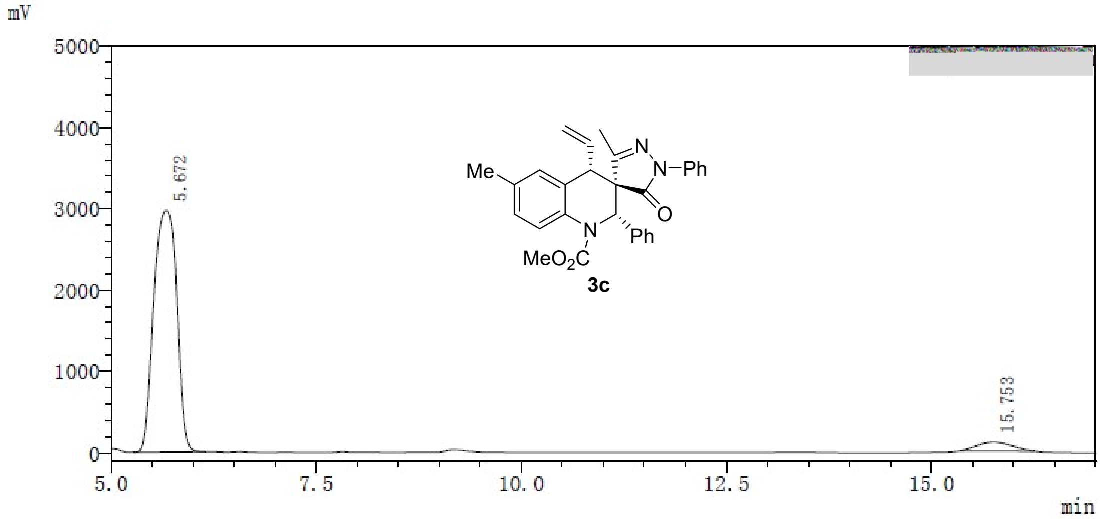

Discloser A 254 nm

Peak	Reten time (min)	Area(%)
1	5.672	94.828
2	15.753	5.172
total		100

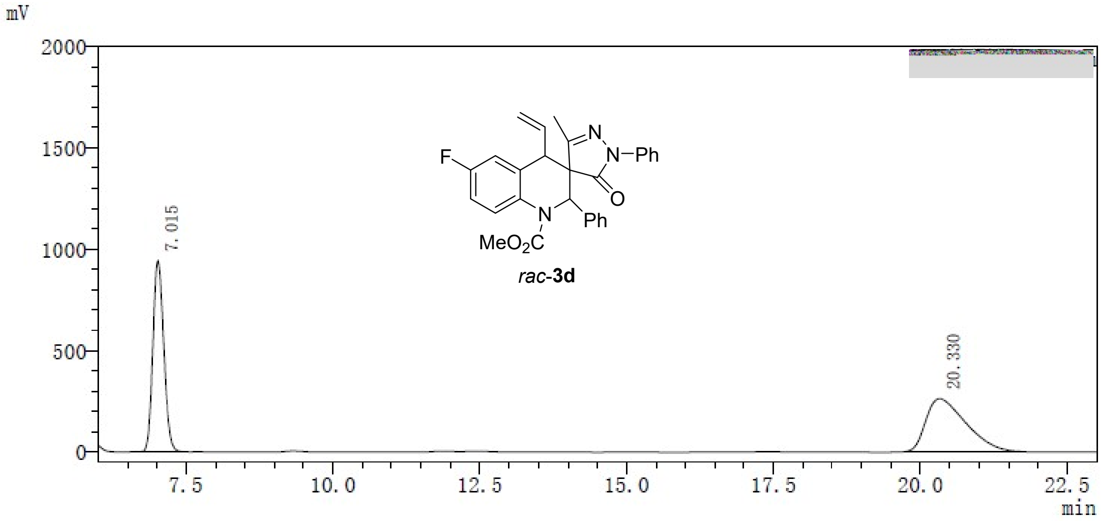

Discloser A 254 nm

Peak	Reten time (min)	Area(%)
1	7.015	49.552
2	20.330	50.448
total		100

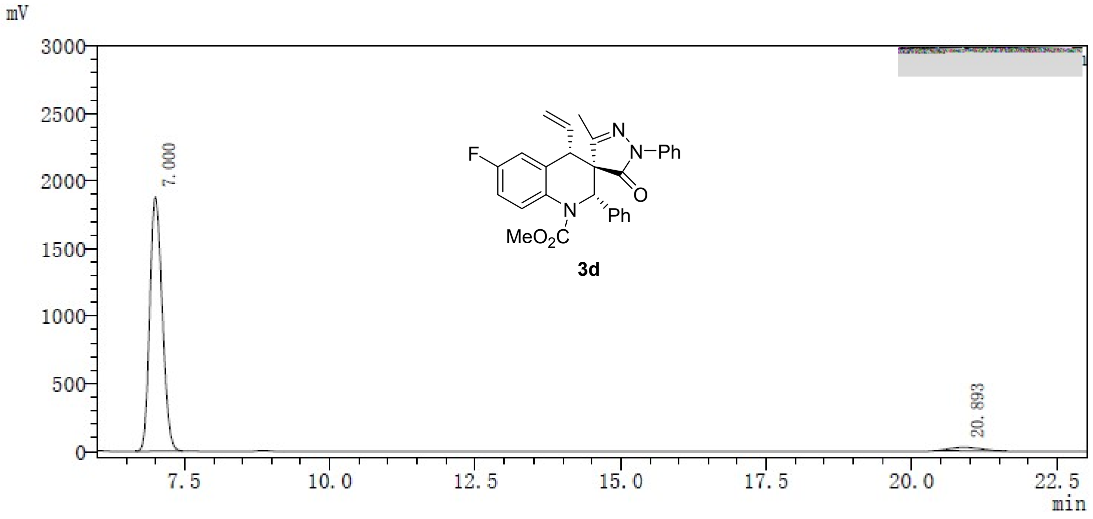

Discloser A 254 nm

Peak	Reten time (min)	Area(%)
1	7.000	96.667
2	20.893	3.333
total		100

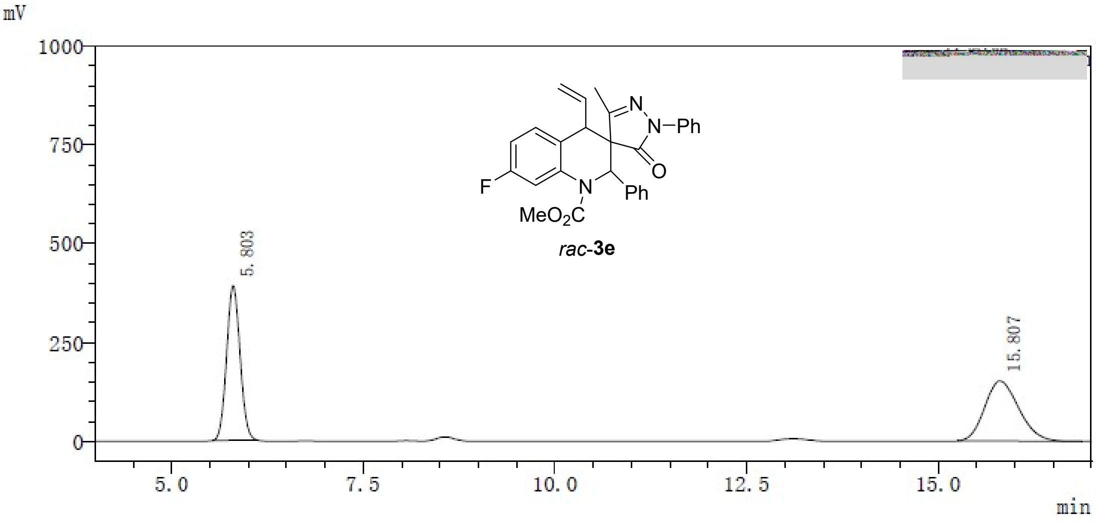

Discloser A 254 nm

Peak	Reten time (min)	Area(%)
1	5.803	49.978
2	15.807	50.022
total		100

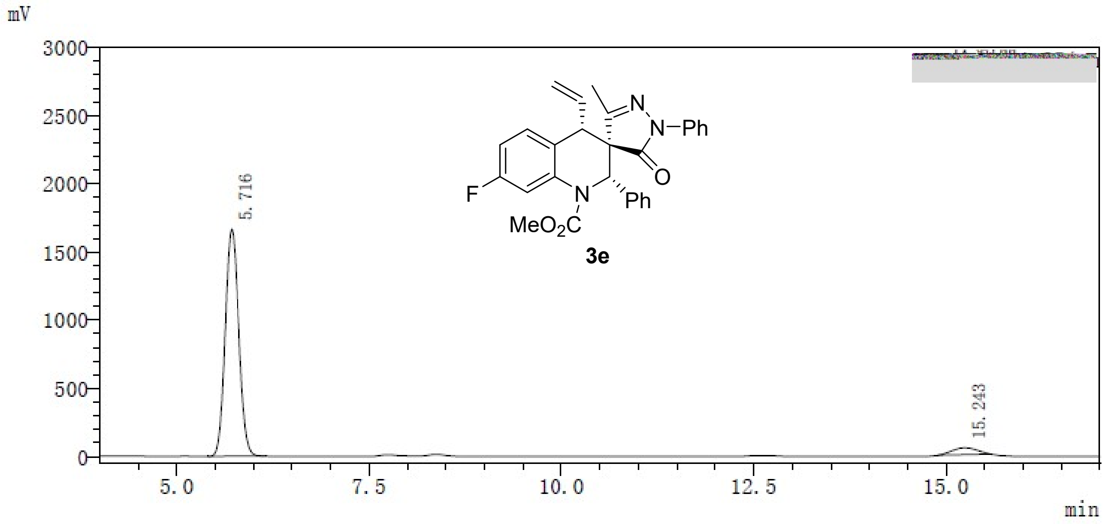

Discloser A 254 nm

Peak	Reten time (min)	Area(%)
1	5.716	94.799
2	15.243	5.201
total		100

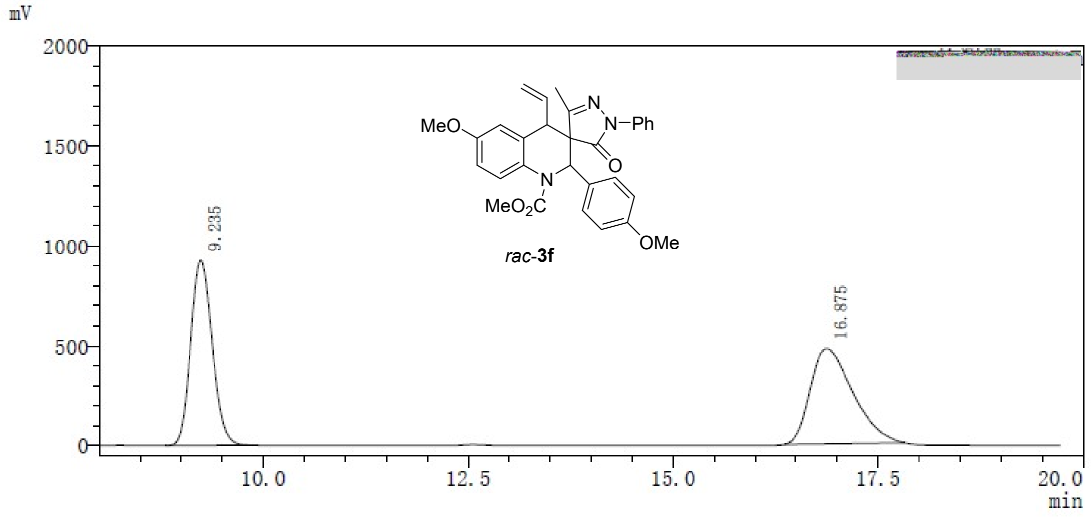

Discloser A 254 nm

Peak	Reten time (min)	Area(%)
1	9.235	49.550
2	16.875	50.450
total		100

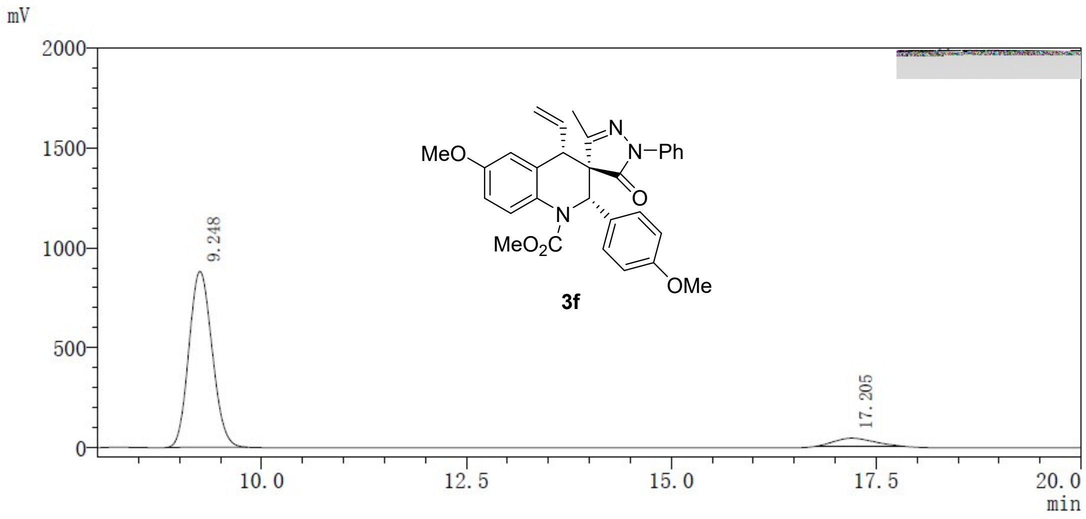

Discloser A 254 nm

Peak	Reten time (min)	Area(%)
1	9.248	92.841
2	17.205	7.159
total		100

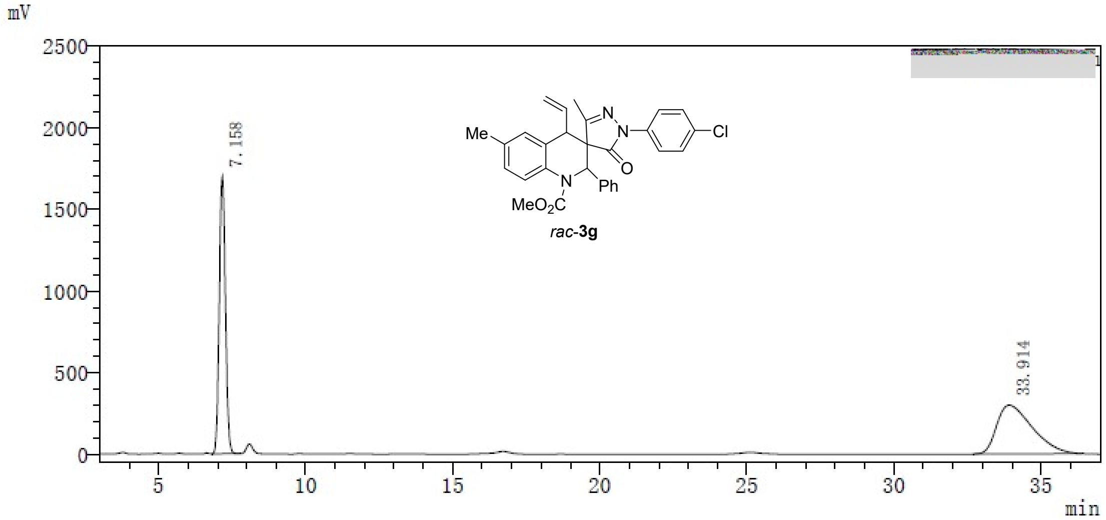

Discloser A 254 nm

Peak	Reten time (min)	Area(%)
1	.7..158	49.360
2	33.914	50.640
total		100

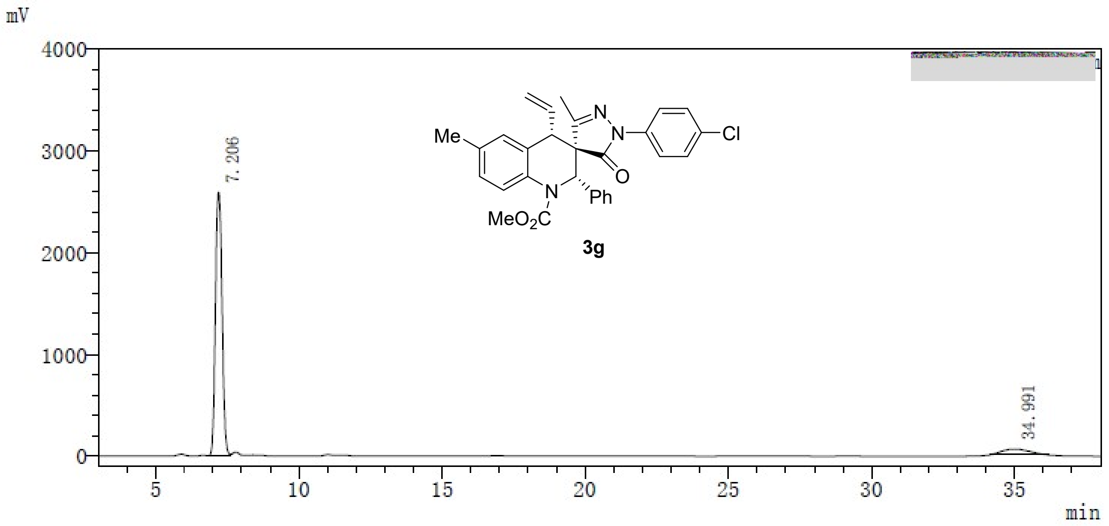

Discloser A 254 nm

Peak	Reten time (min)	Area(%)
1	7.206	91.497
2	34.991	8.503
total		100

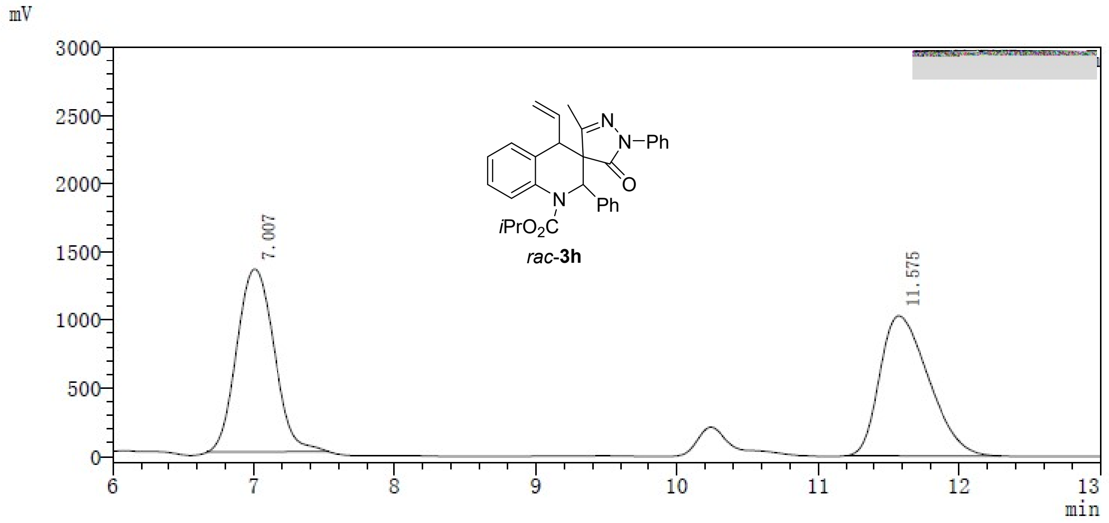

Discloser A 254 nm

Peak	Reten time (min)	Area(%)
1	7.007	50.046
2	11.575	49.954
total		100

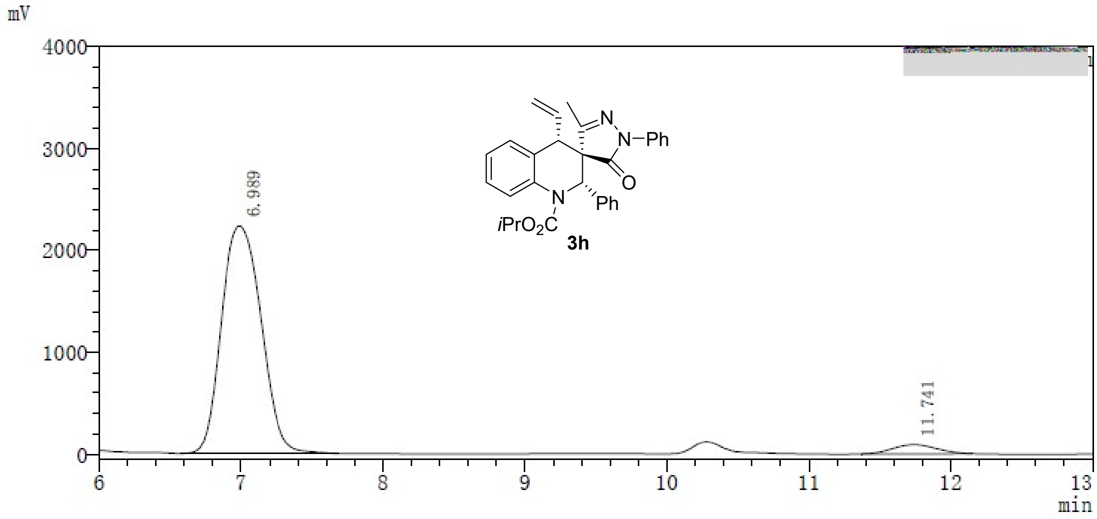

Discloser A 254 nm

Peak	Reten time (min)	Area(%)
1	6.989	95.788
2	11.741	4.212
total		100

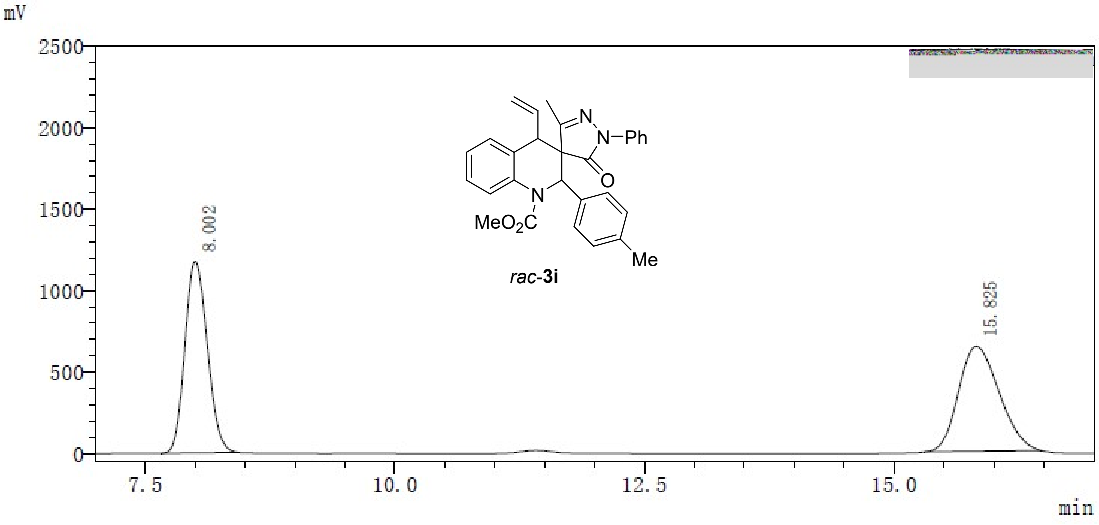

Discloser A 254 nm

Peak	Reten time (min)	Area(%)
1	8.002	50.072
2	15.825	49.828
total		100

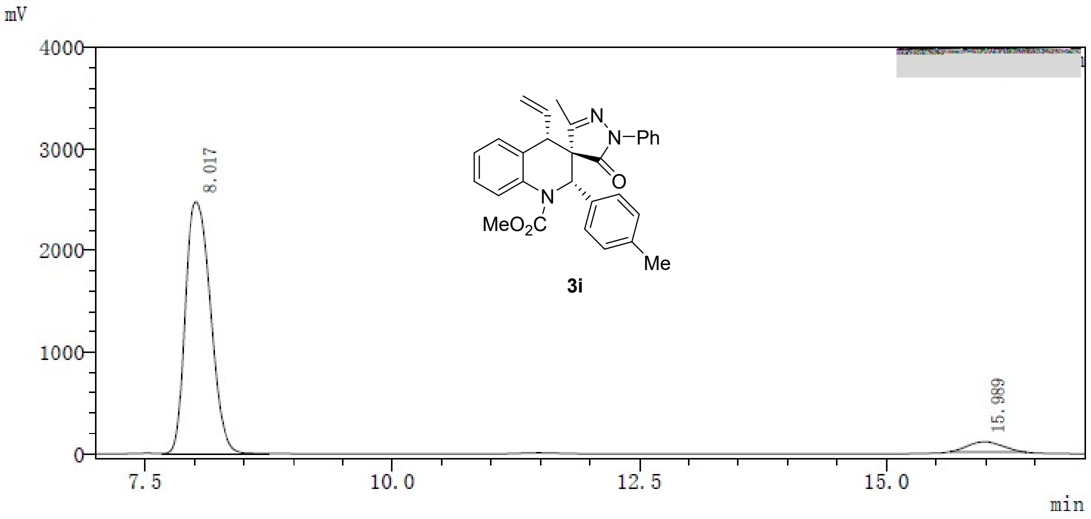

Discloser A 254 nm

Peak	Reten time (min)	Area(%)
1	8.017	94.782
2	15.989	5.218
total		100

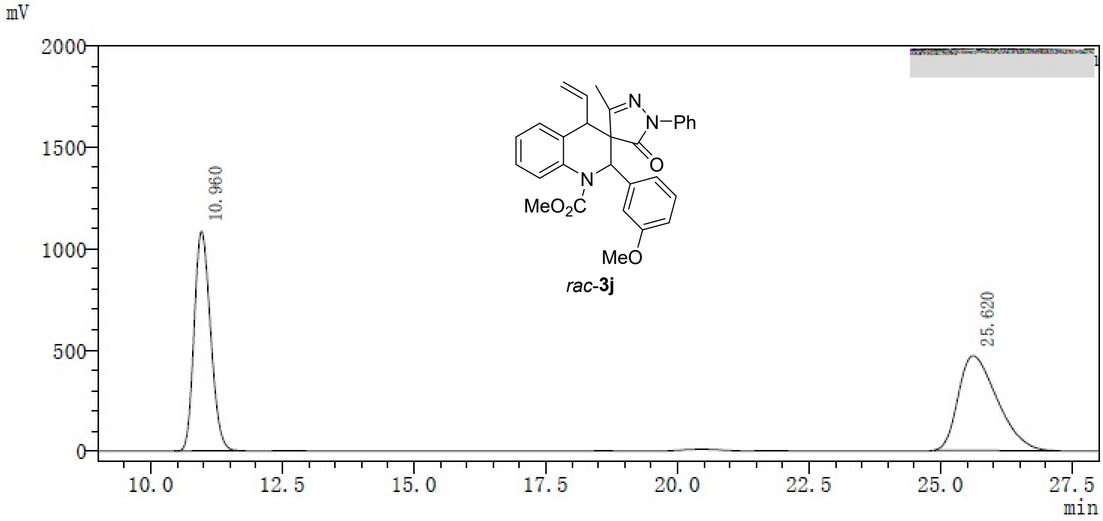

Discloser A 254 nm

Peak	Reten time (min)	Area(%)
1	10.960	49.343
2	25.620	50.657
total		100

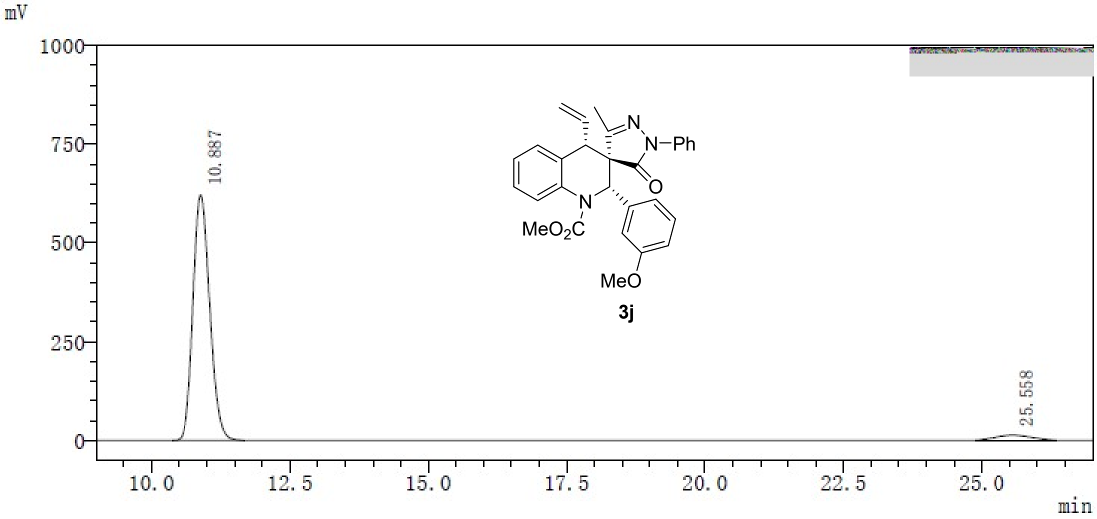

Discloser A 254 nm

Peak	Reten time (min)	Area(%)
1	10.887	96.095
2	25.558	3.905
total		100

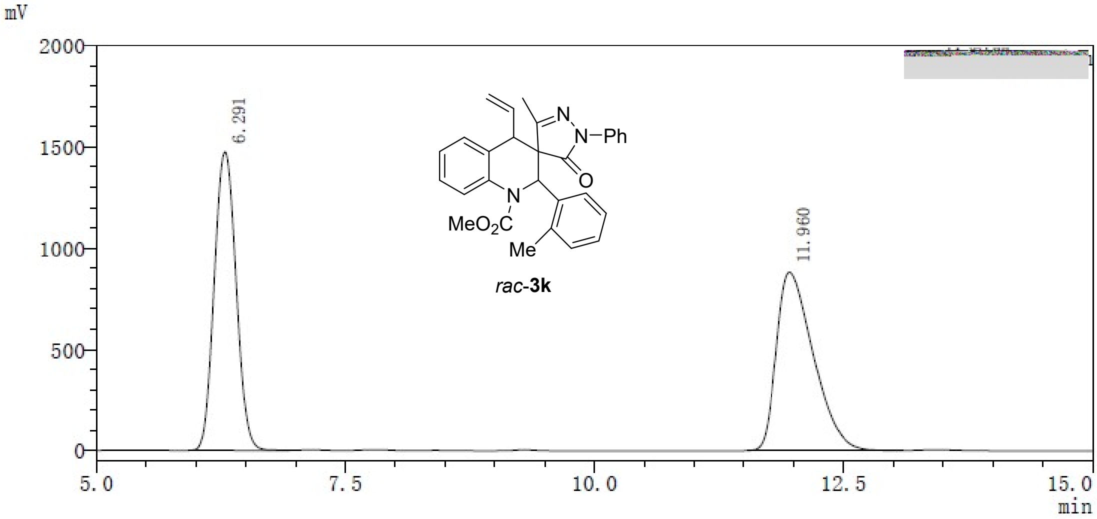

Discloser A 254 nm

Peak	Reten time (min)	Area(%)
1	6.291	49.623
2	11.960	50.377
total		100

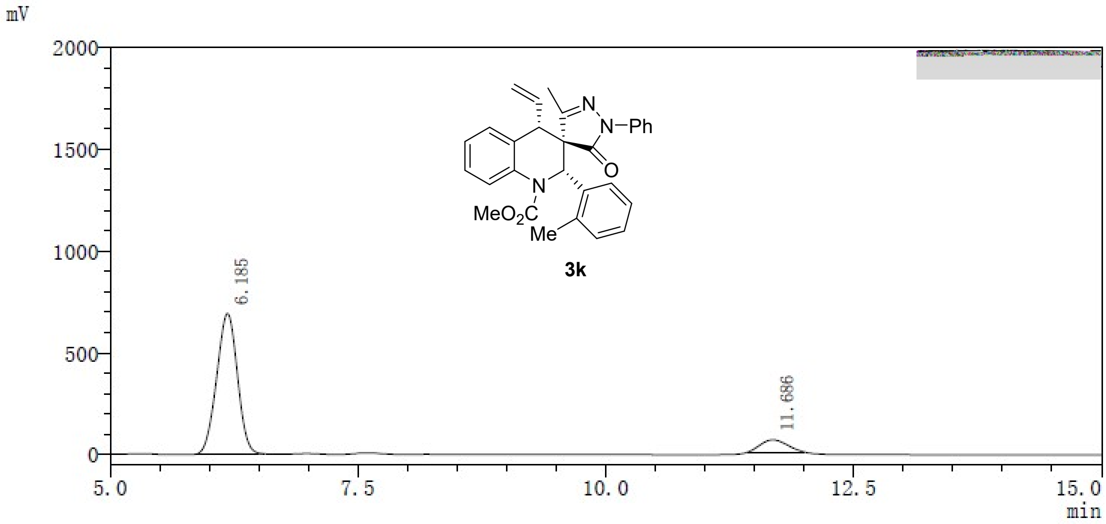

Discloser A 254 nm

Peak	Reten time (min)	Area(%)
1	6.185	90.109
2	11.686	9.891
total		100

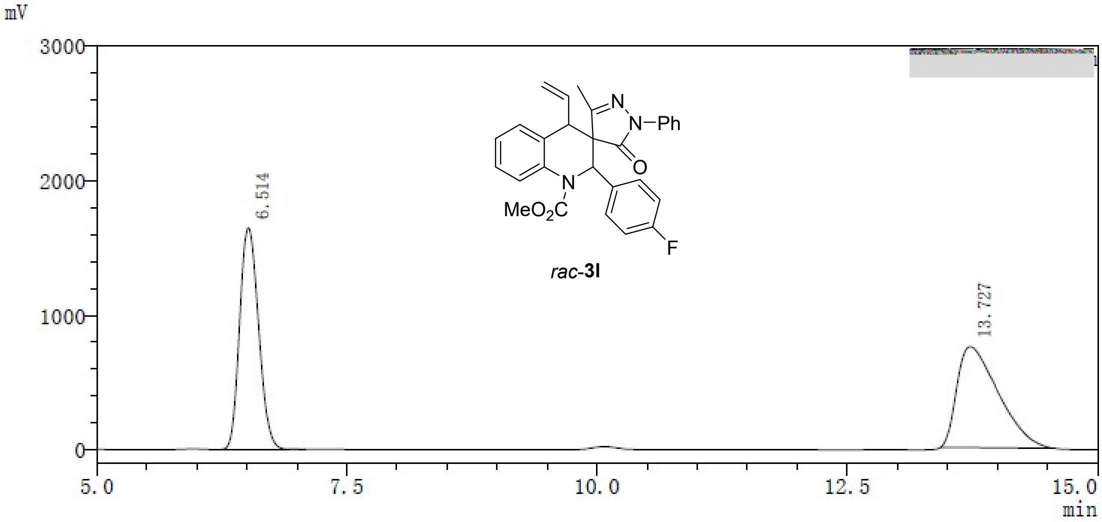

Discloser A 254 nm

Peak	Reten time (min)	Area(%)
1	6.514	50.060
2	13.727	49.940
total		100

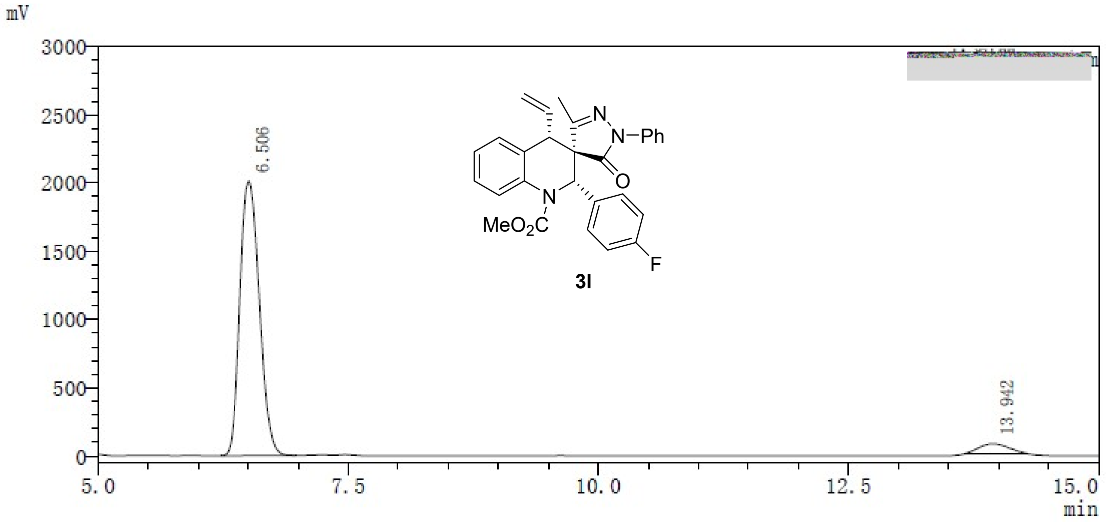

Discloser A 254 nm

Peak	Reten time (min)	Area(%)
1	6.506	95.128
2	13.942	4.872
total		100

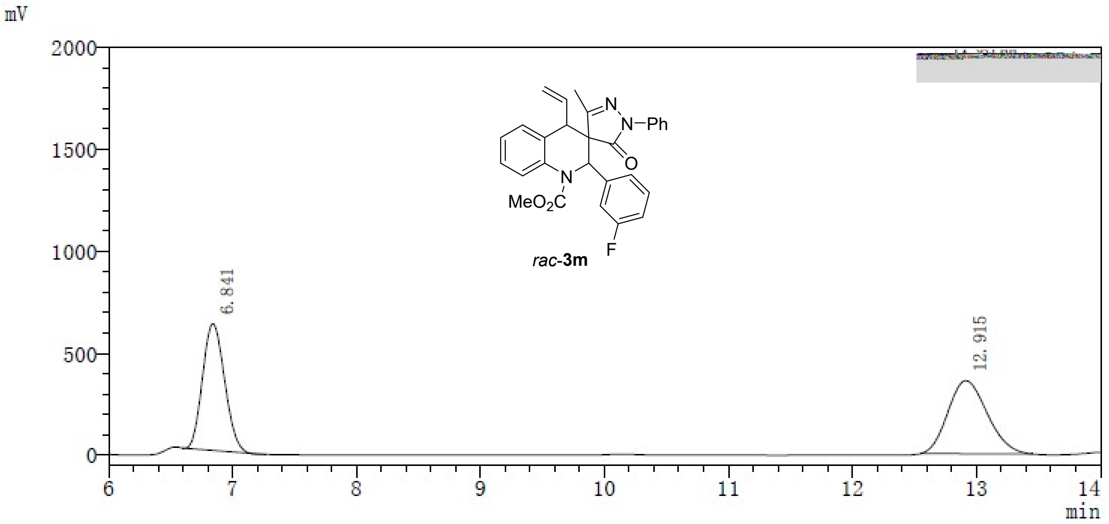

Discloser A 254 nm

Peak	Reten time (min)	Area(%)
1	6.841	49.290
2	12.915	50.710
total		100

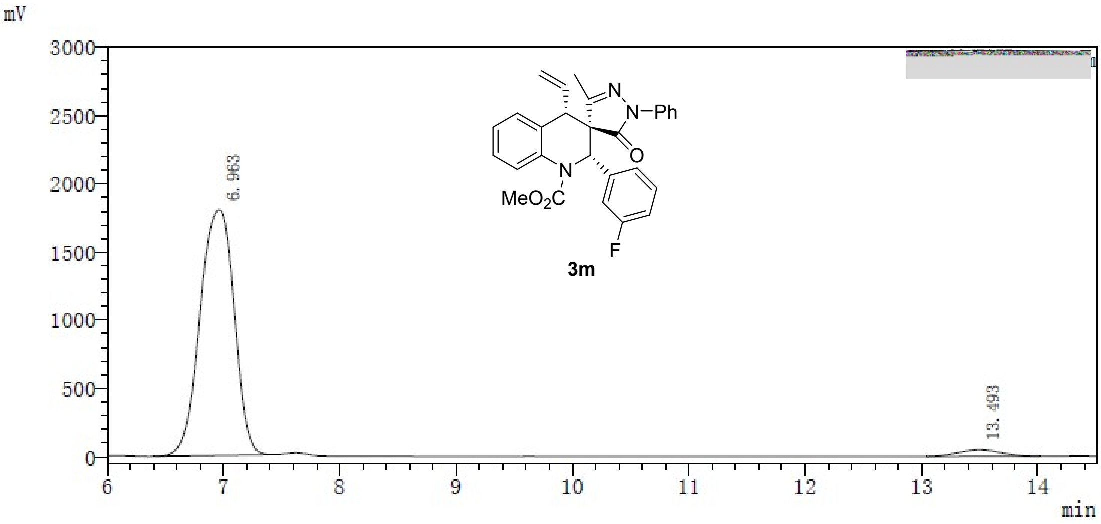

Discloser A 254 nm

Peak	Reten time (min)	Area(%)
1	6.963	96.653
2	13.493	3.347
total		100

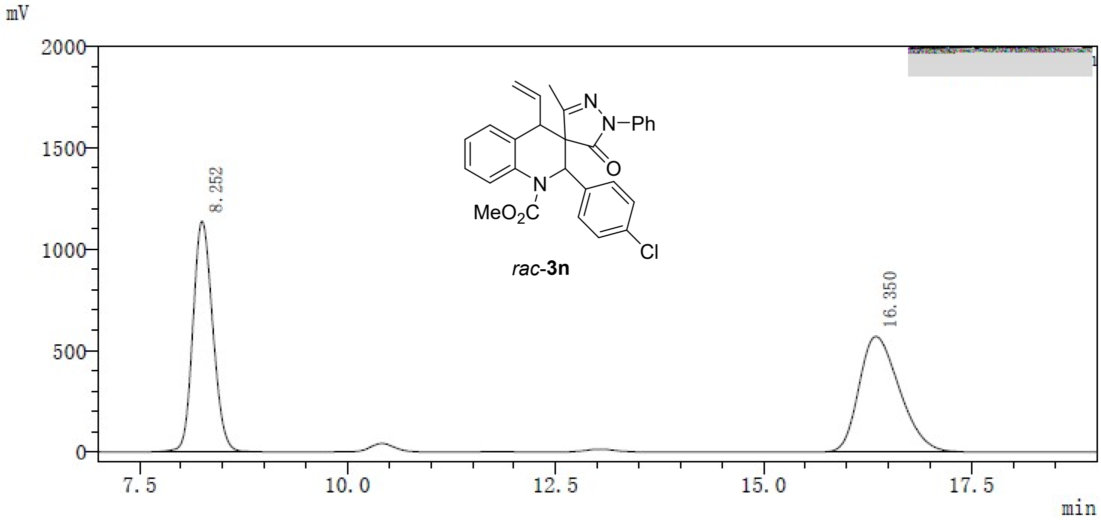

Discloser A 254 nm

Peak	Reten time (min)	Area(%)
1	8.252	49.589
2	18.618	50.411
total		100

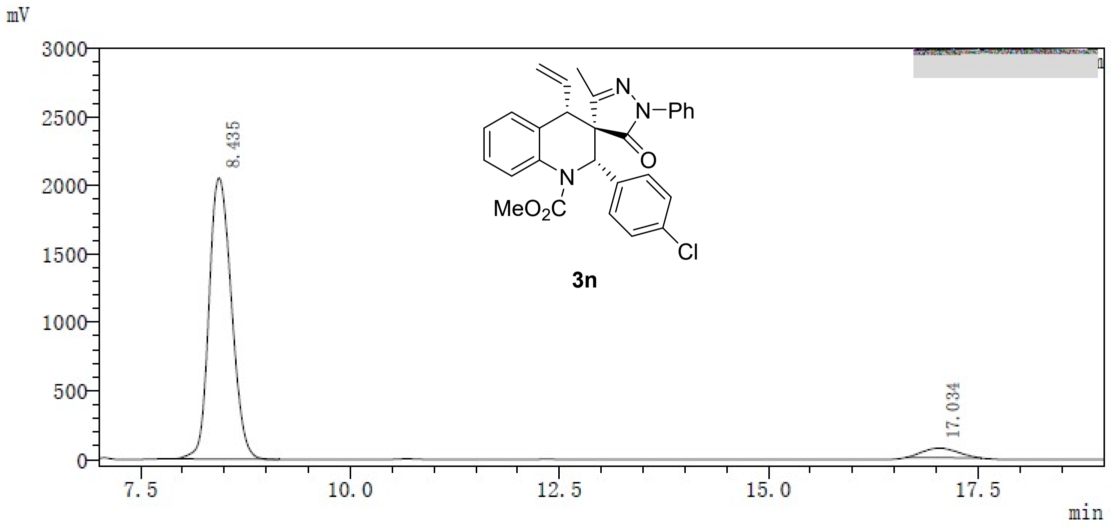

Discloser A 254 nm

Peak	Reten time (min)	Area(%)
1	8.435	95.045
2	17.034	4.955
total		100

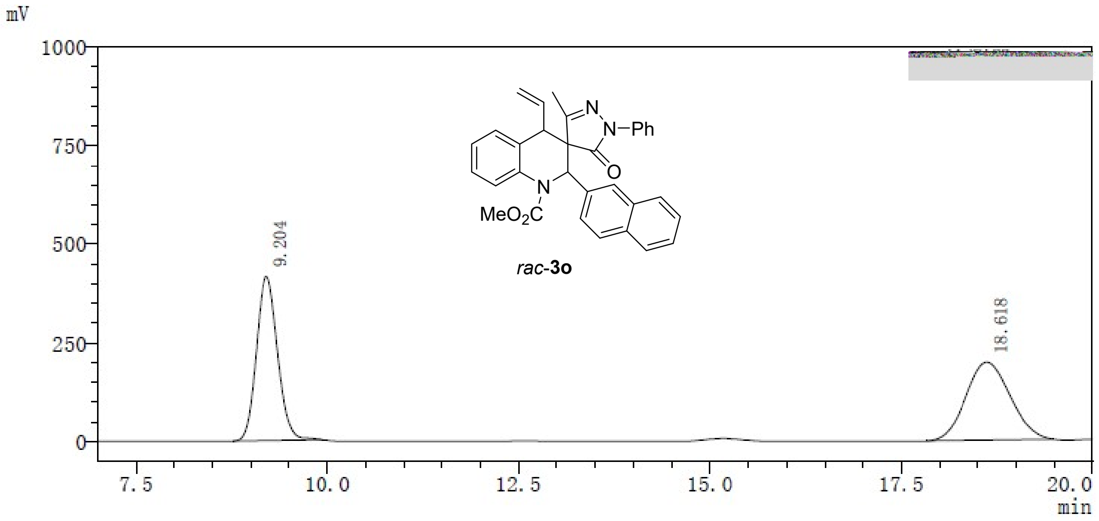

Discloser A 254 nm

Peak	Reten time (min)	Area(%)
1	9.204	50.292
2	18.618	49.708
total		100

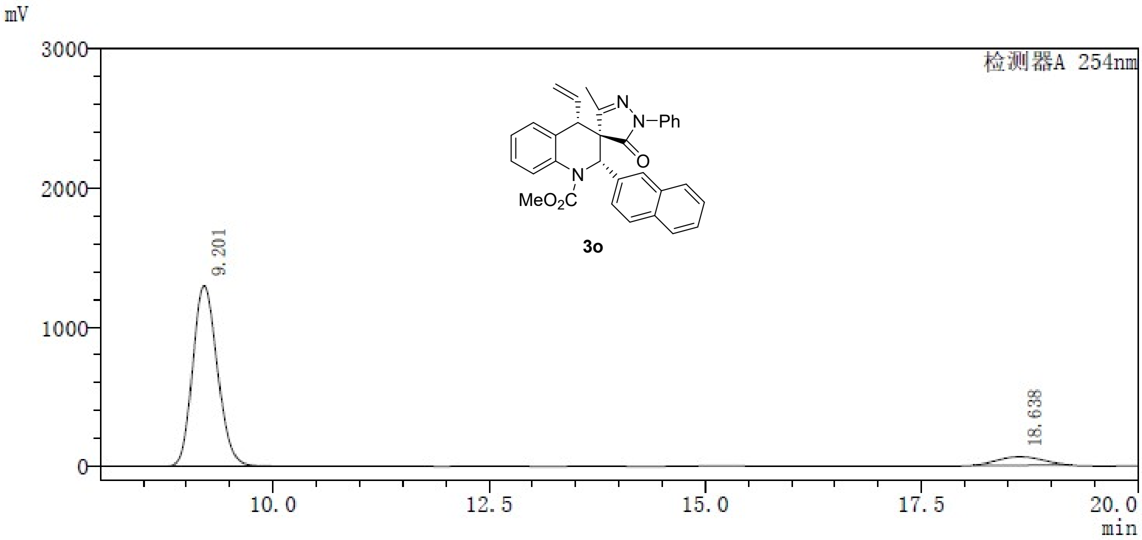

Discloser A 254 nm

Peak	Reten time (min)	Area(%)
1	9.201	92.401
2	18.638	7.599
total		100

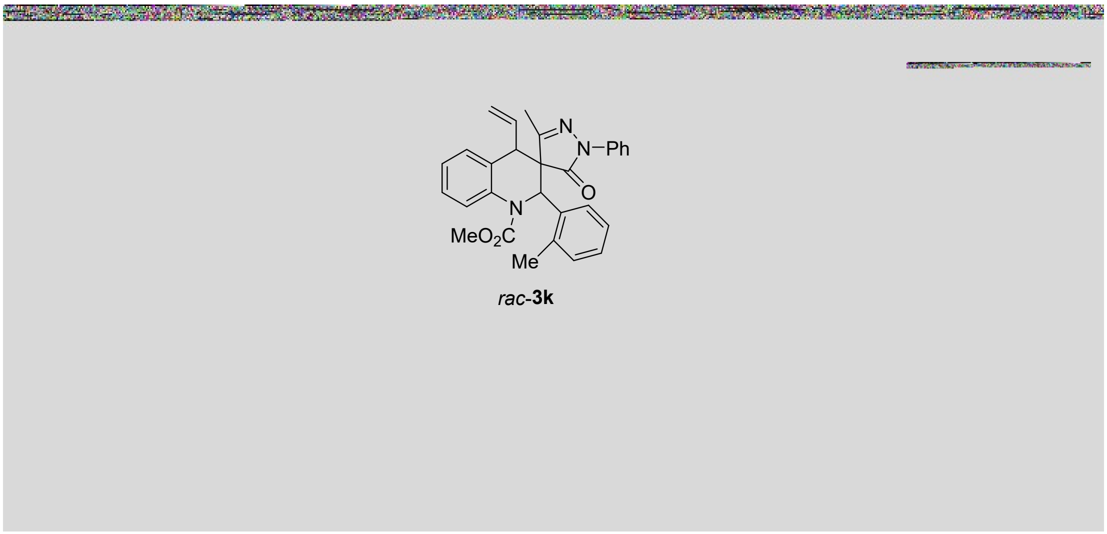

Discloser A 254 nm

Peak	Reten time (min)	Area(%)
1	6.291	49.623
2	11.960	50.377
total		100

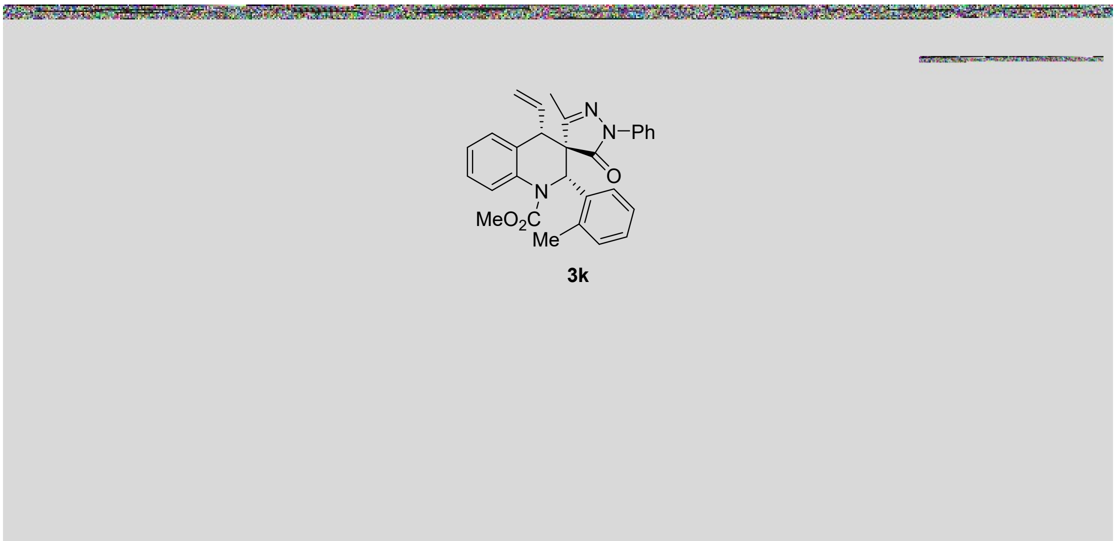

Discloser A 254 nm

Peak	Reten time (min)	Area(%)
1	6.185	90.109
2	11.686	9.891
total		100

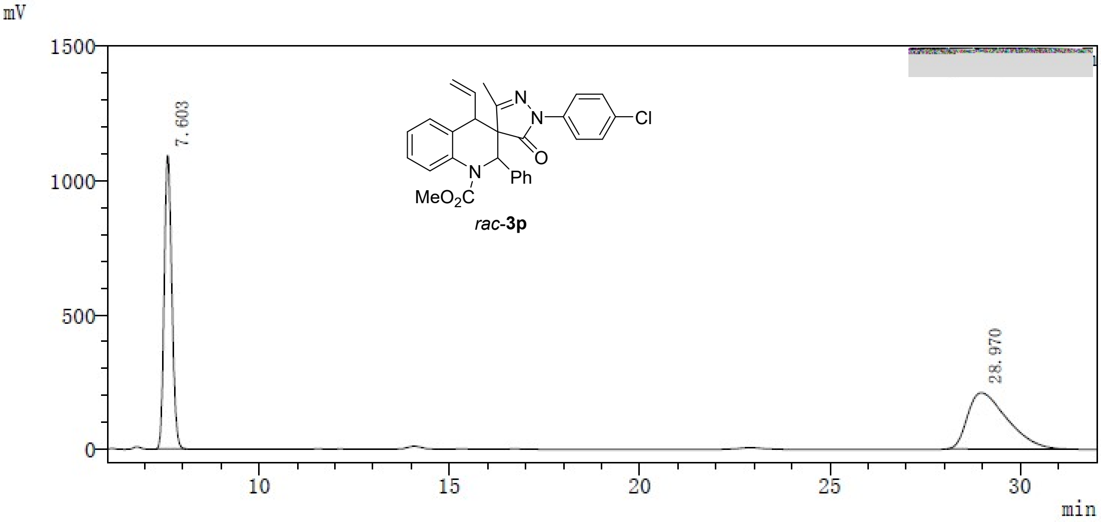

Discloser A 254 nm

Peak	Reten time (min)	Area(%)
1	7.603	49.978
2	28.970	50.022
total		100

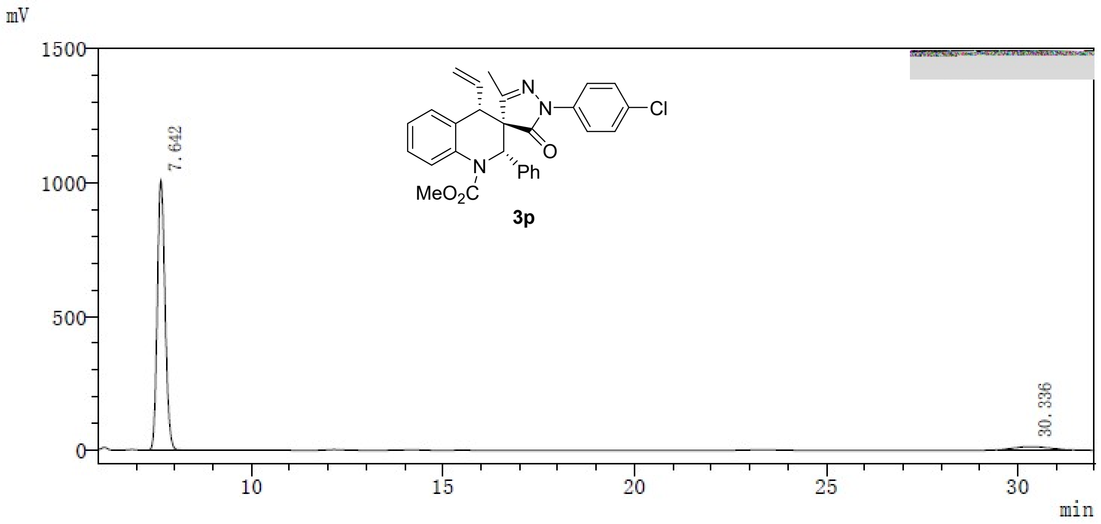

Discloser A 254 nm

Peak	Reten time (min)	Area(%)
1	7.642	95.116
2	30.336	4.884
total		100

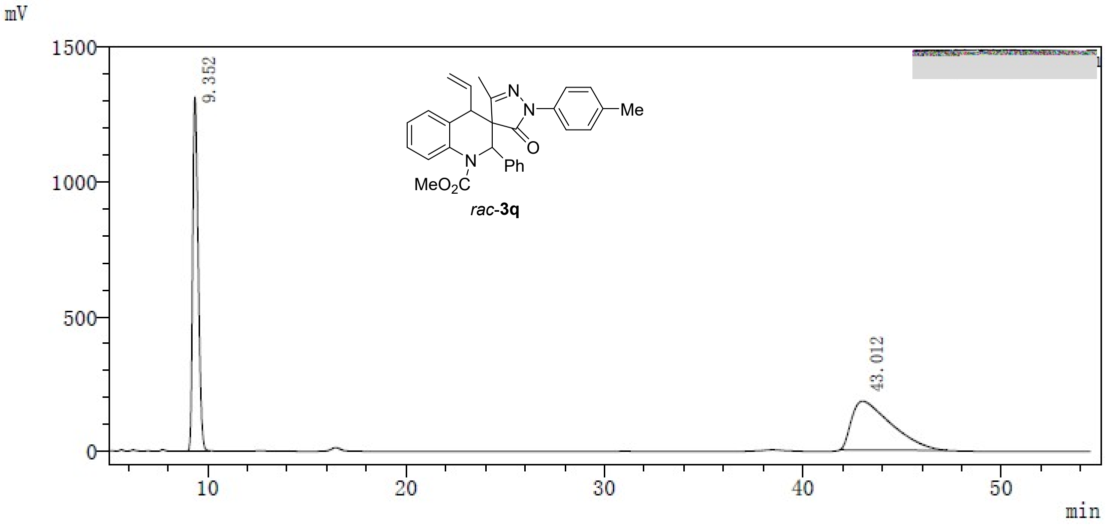

Discloser A 254 nm

Peak	Reten time (min)	Area(%)
1	9.352	50.031
2	43.012	49.969
total		100

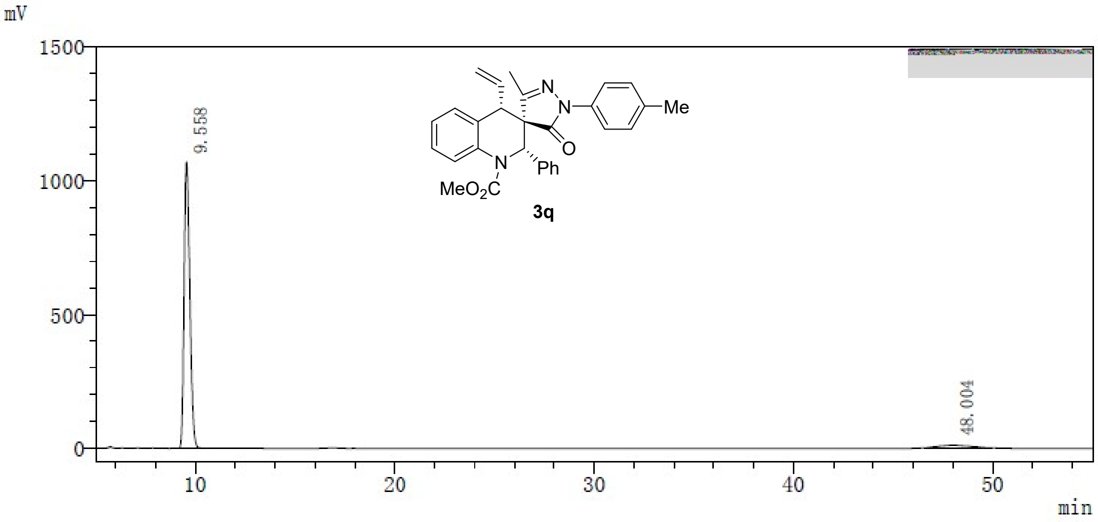

Discloser A 254 nm

Peak	Reten time (min)	Area(%)
1	.9.558	95.180
2	48.004	4.820
total		100

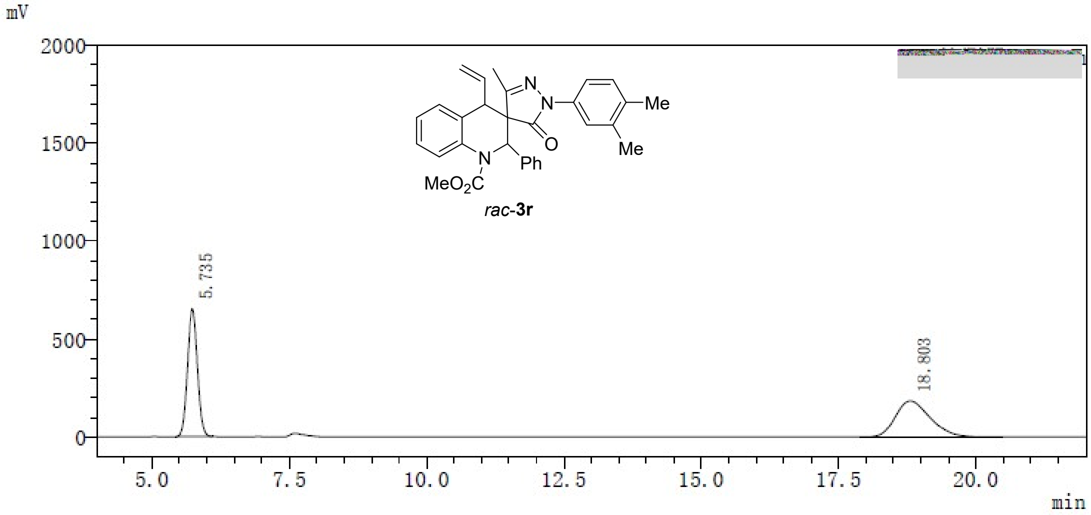

Discloser A 254 nm

Peak	Reten time (min)	Area(%)
1	5.735	50.571
2	18.803	49.429
total		100

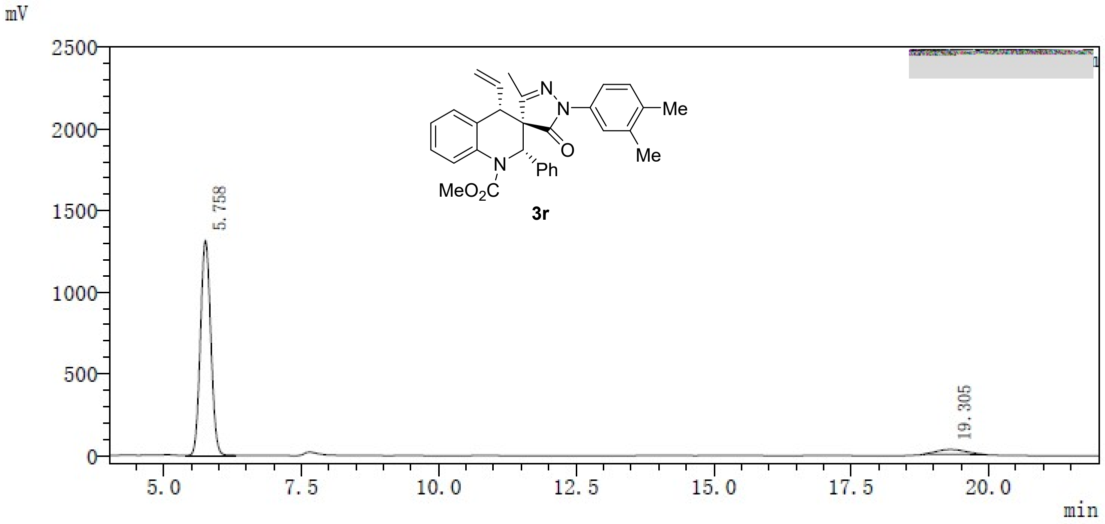

Discloser A 254 nm

Peak	Reten time (min)	Area(%)
1	5.758	94.274
2	19.305	5.726
total		100

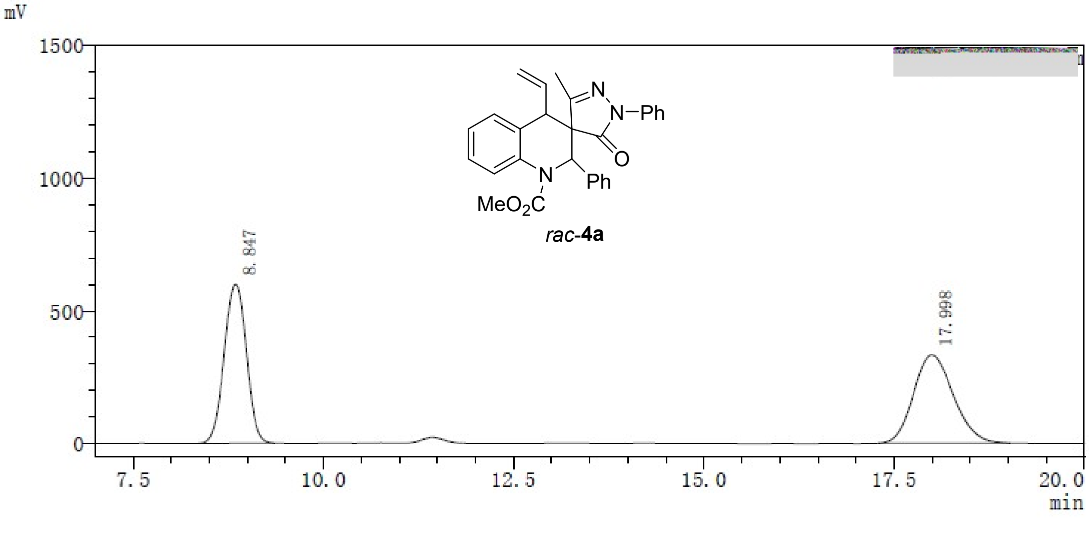

Discloser A 254 nm

Peak	Reten time (min)	Area(%)
1	8.847	50.076
2	17.998	49.824
total		100

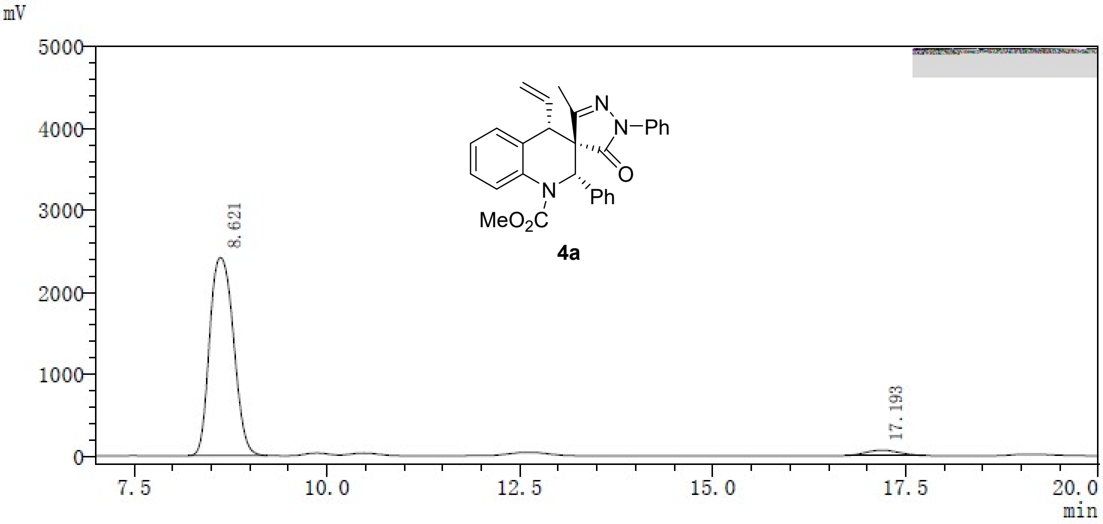

Discloser A 254 nm

Peak	Reten time (min)	Area(%)
1	8.621	96.284
2	17.193	3.716
total		100

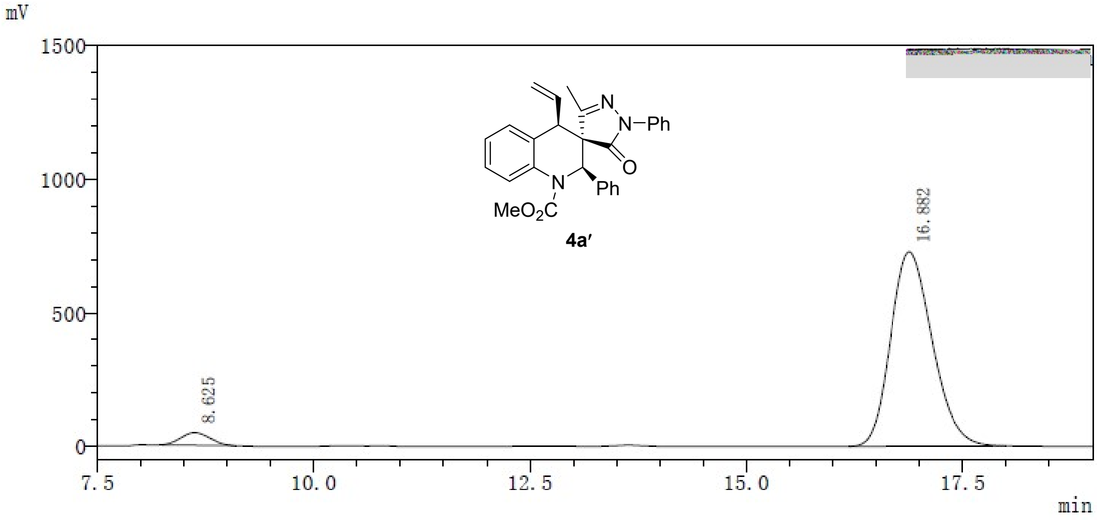

Discloser A 254 nm

Peak	Reten time (min)	Area(%)
1	8.625	4.302
2	16.882	95.698
total		100

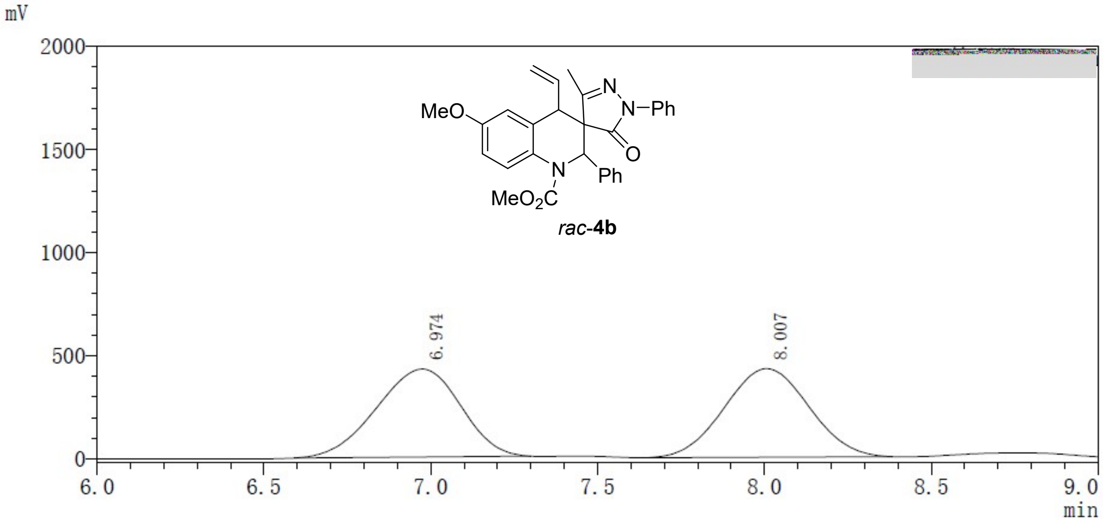

Discloser A 254 nm

Peak	Reten time (min)	Area(%)
1	6.974	49.734
2	8.007	50.266
total		100

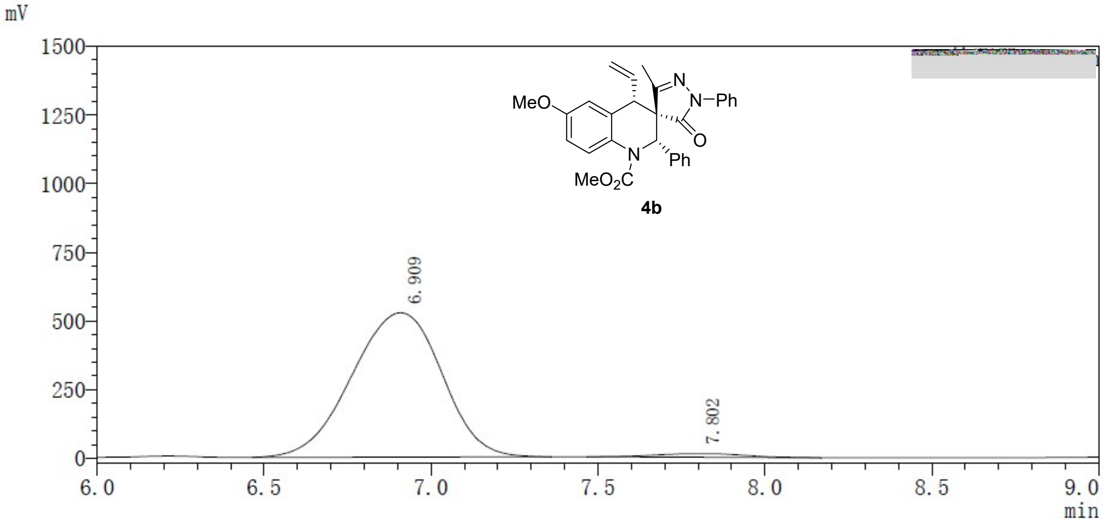

Discloser A 254 nm

Peak	Reten time (min)	Area(%)
1	6.909	97.631
2	7.802	2.369
total		100

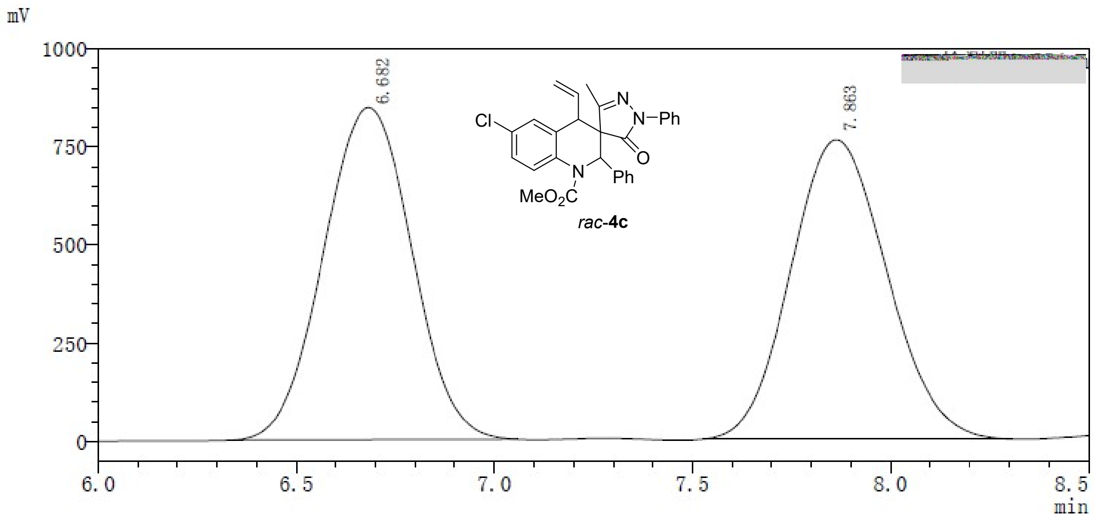

Discloser A 254 nm

Peak	Reten time (min)	Area(%)
1	6.682	50.489
2	7.863	49.511
total		100

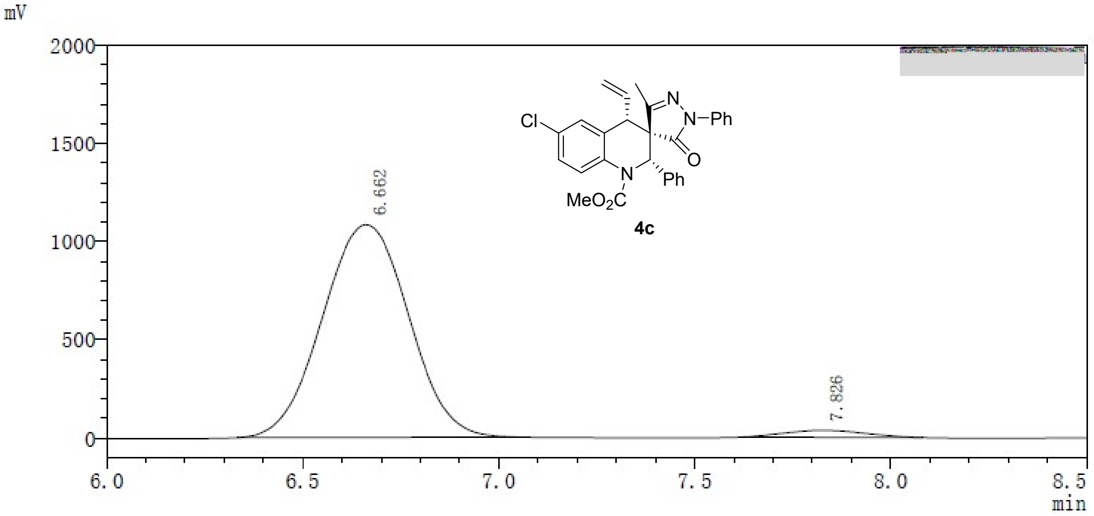

Discloser A 254 nm

Peak	Reten time (min)	Area(%)
1	6.662	97.049
2	7.826	2.951
total		100

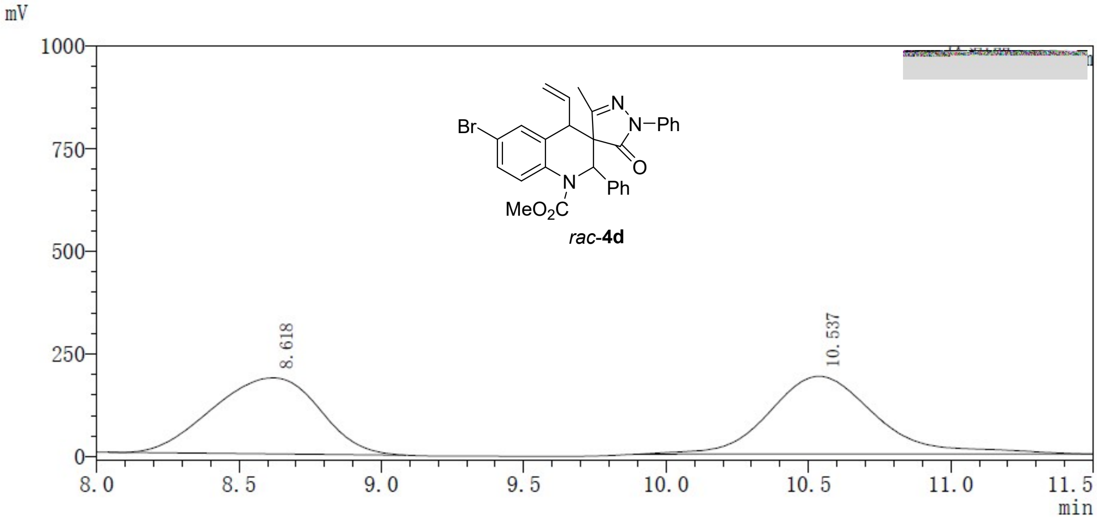

Discloser A 254 nm

Peak	Reten time (min)	Area(%)
1	8.618	49.477
2	10.537	50.523
total		100

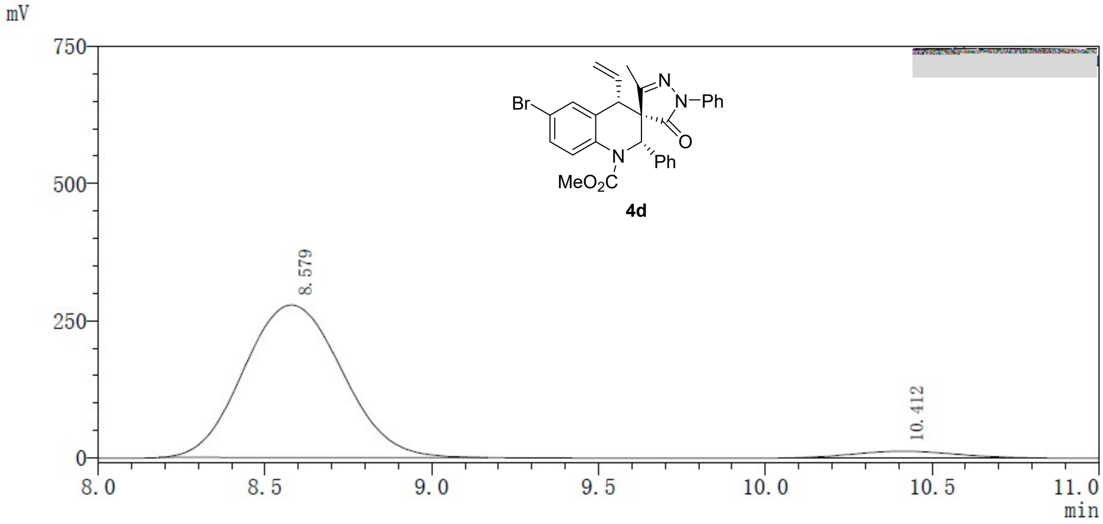

Discloser A 254 nm

Peak	Reten time (min)	Area(%)
1	8.579	95.650
2	10.412	4.350
total		100

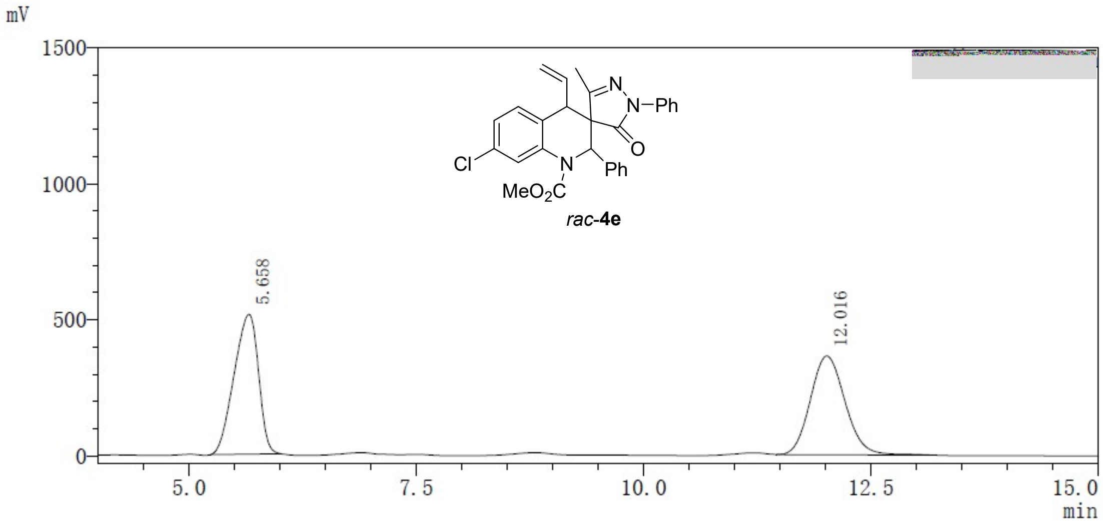

Discloser A 254 nm

Peak	Reten time (min)	Area(%)
1	5.658	49.942
2	12.016	50.058
total		100

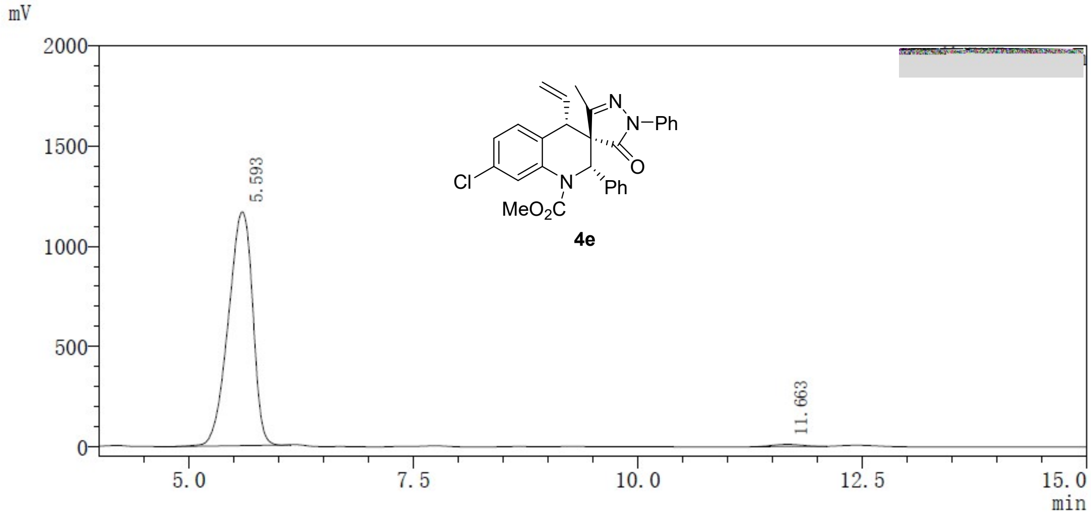

Discloser A 254 nm

Peak	Reten time (min)	Area(%)
1	5.593	99.063
2	11.663	0.937
total		100

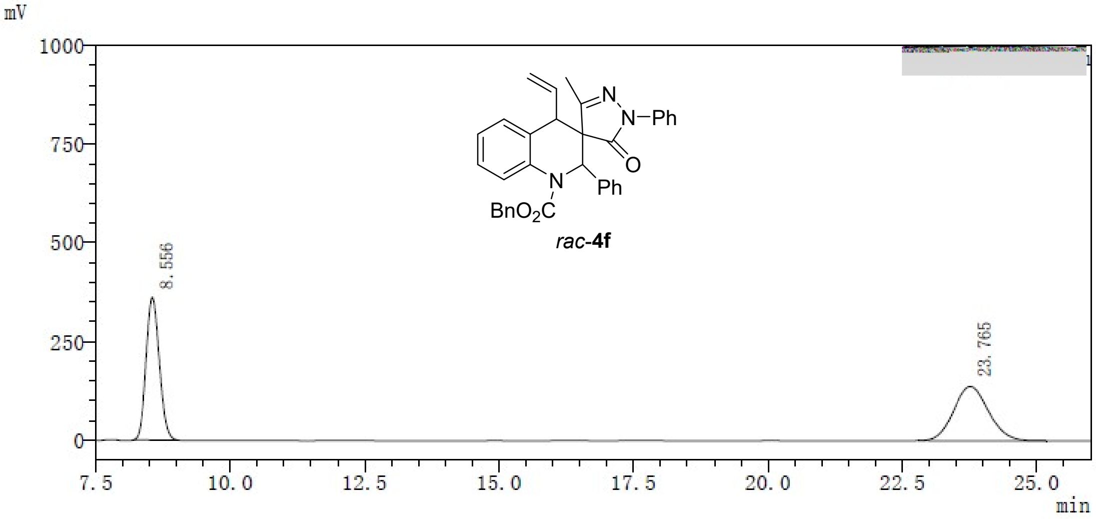

Discloser A 254 nm

Peak	Reten time (min)	Area(%)
1	8.556	49.795
2	22.765	50.205
total		100

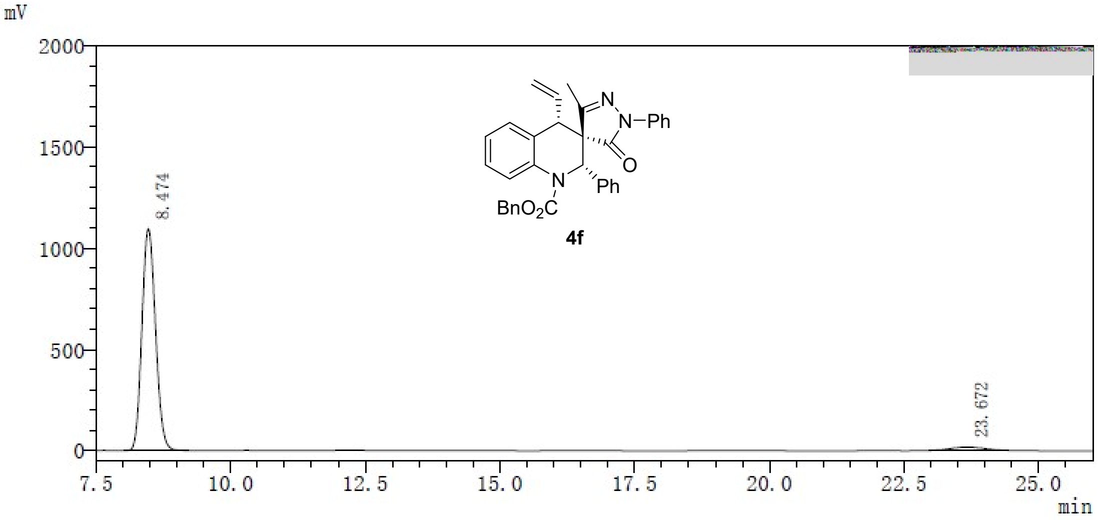

Discloser A 254 nm

Peak	Reten time (min)	Area(%)
1	8.474	96.429
2	23.672	3.571
total		100

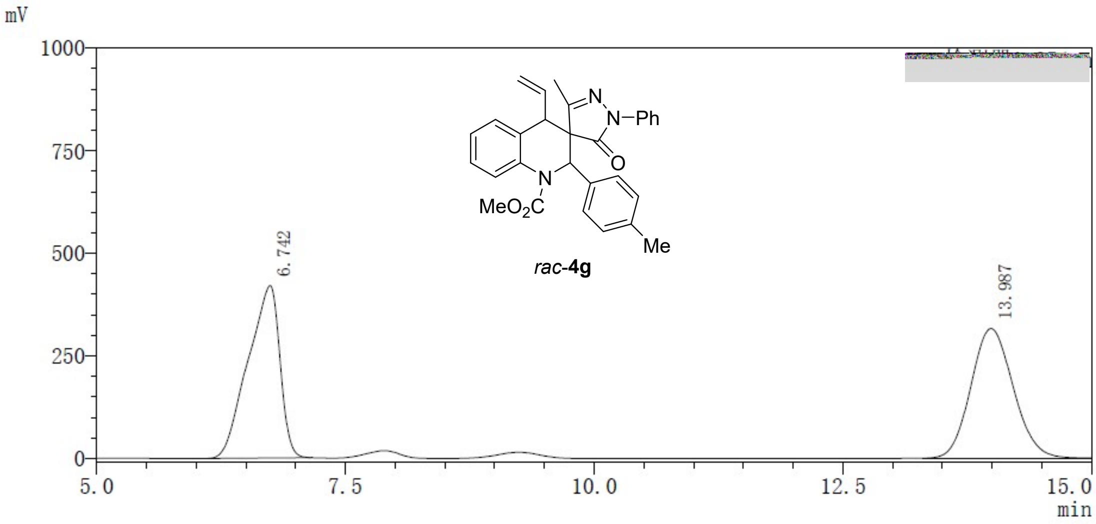

Discloser A 254 nm

Peak	Reten time (min)	Area(%)
1	6.742	49.974
2	13.987	50.026
total		100

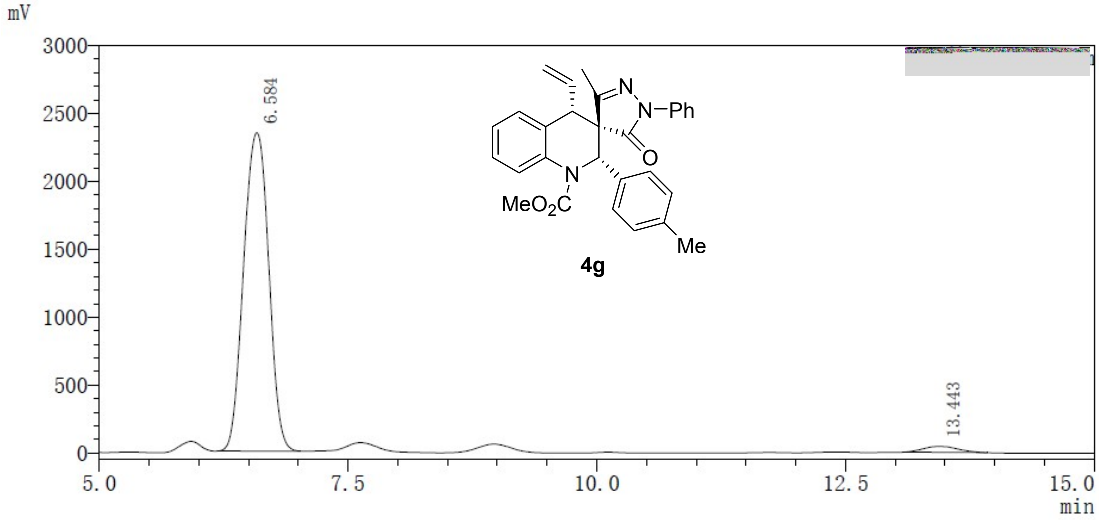

Discloser A 254 nm

Peak	Reten time (min)	Area(%)
1	6.584	97.522
2	13.443	2.478
total		100

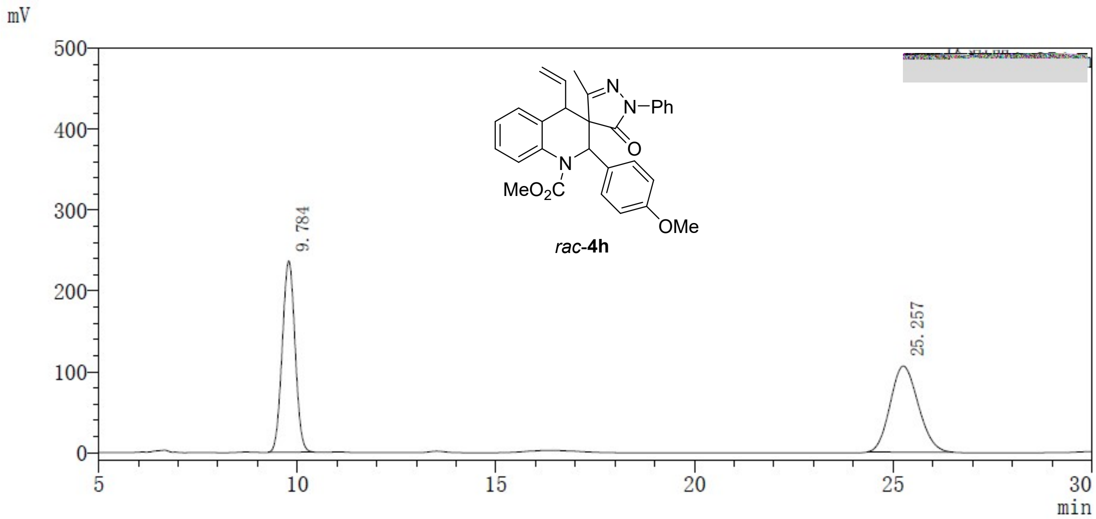

Discloser A 254 nm

Peak	Reten time (min)	Area(%)
1	9.784	50.278
2	25.257	49.722
total		100

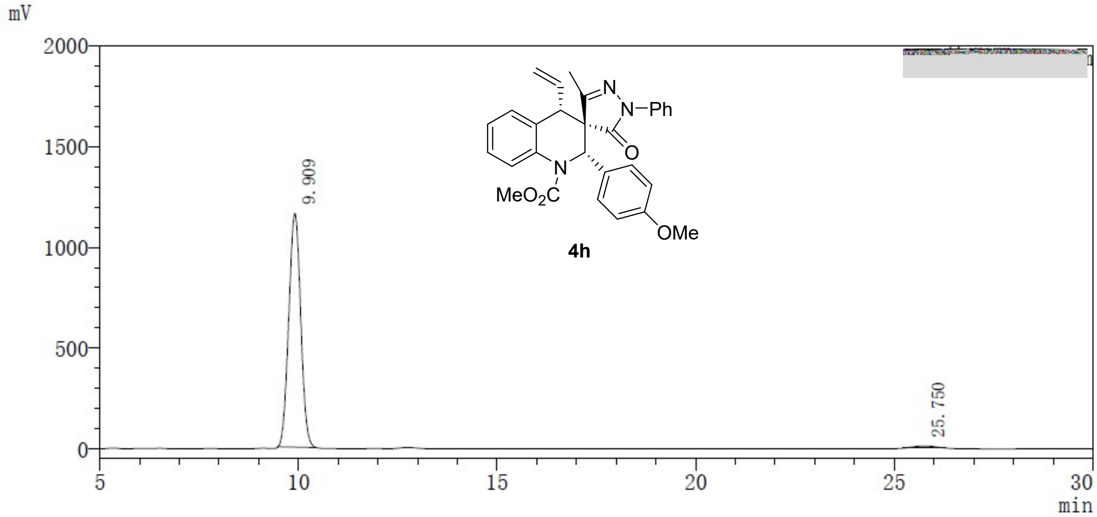

Discloser A 254 nm

Peak	Reten time (min)	Area(%)
1	9.909	98.515
2	25.750	1.485
total		100

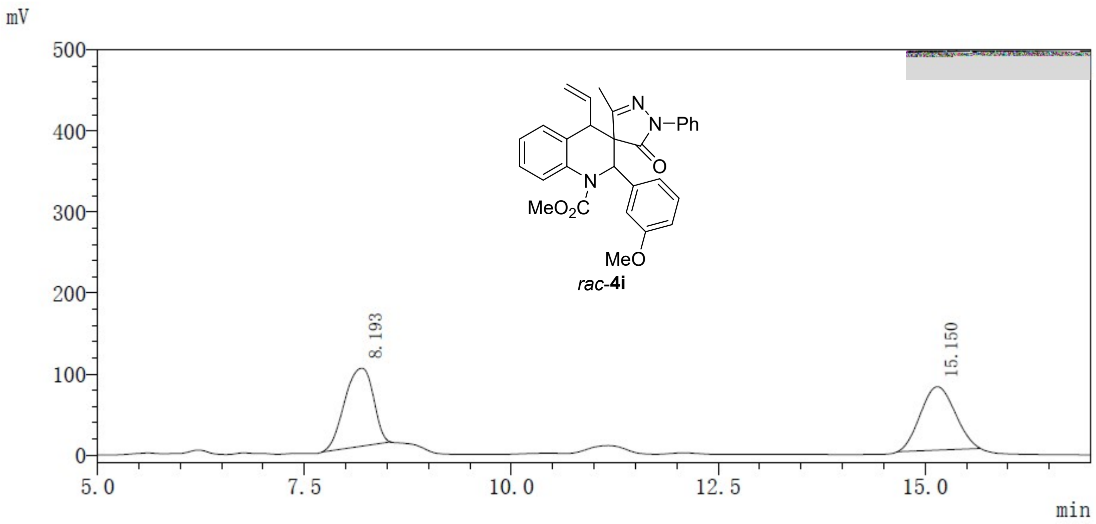

Discloser A 254 nm

Peak	Reten time (min)	Area(%)
1	8.193	50.588
2	15.150	49.412
total		100

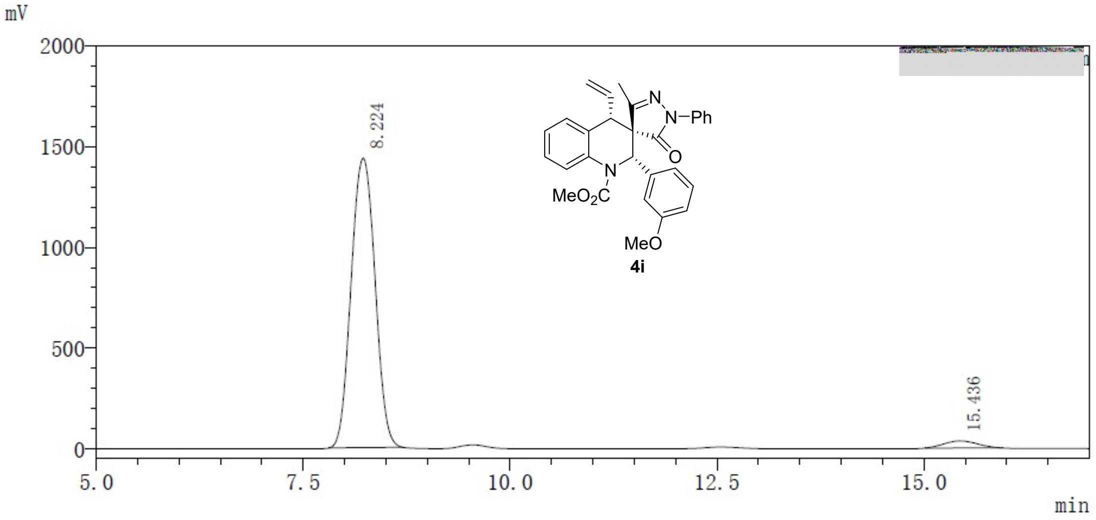

Discloser A 254 nm

Peak	Reten time (min)	Area(%)
1	8.224	96.700
2	15.436	3.300
total		100

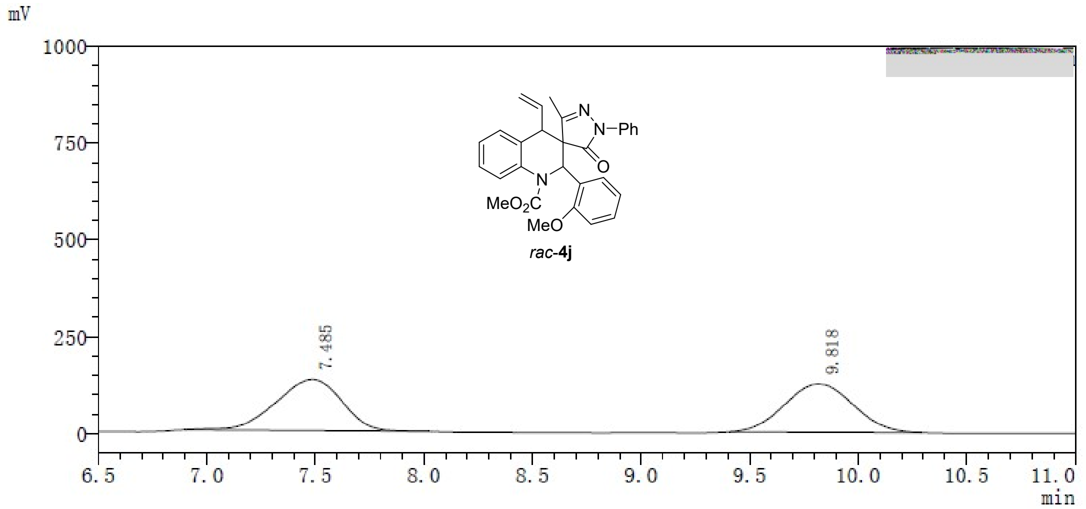

Discloser A 254 nm

Peak	Reten time (min)	Area(%)
1	7.485	50.493
2	9.818	49.507
total		100

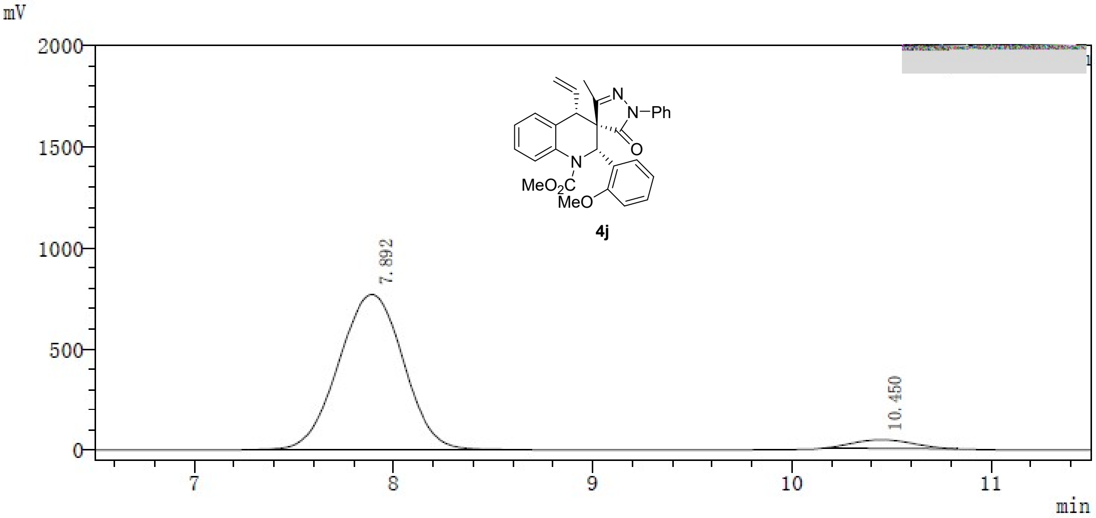

Discloser A 254 nm

Peak	Reten time (min)	Area(%)
1	7.892	95.042
2	10.450	4.958
total		100

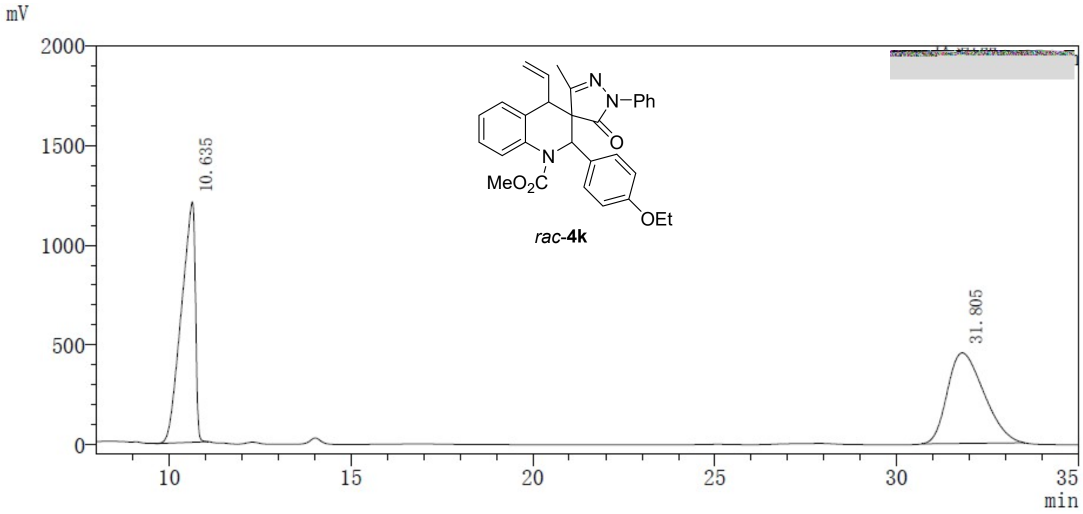

Discloser A 254 nm

Peak	Reten time (min)	Area(%)
1	10.635	50.355
2	31.805	49.645
total		100

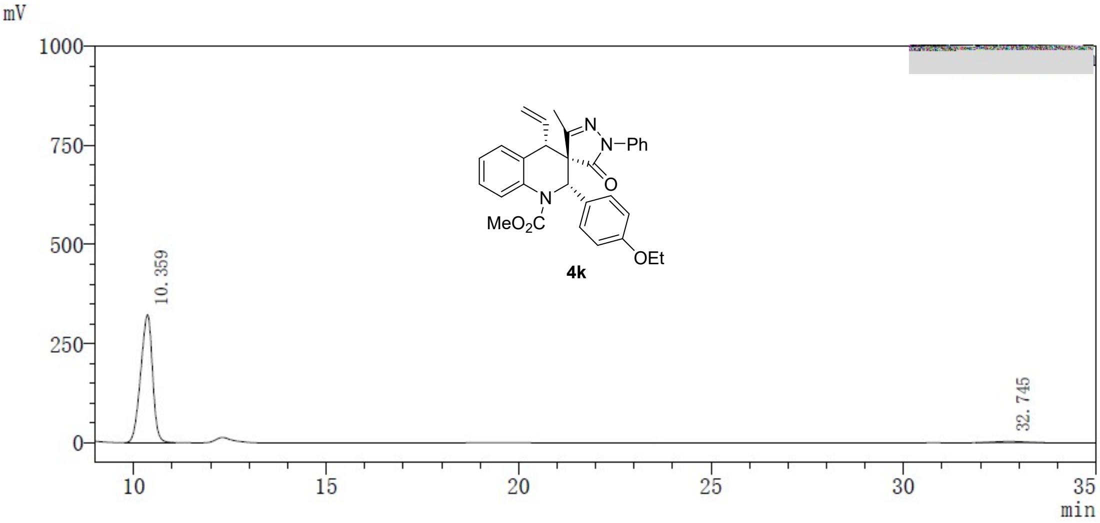

Discloser A 254 nm

Peak	Reten time (min)	Area(%)
1	10.359	97.393
2	32.745	2.607
total		100

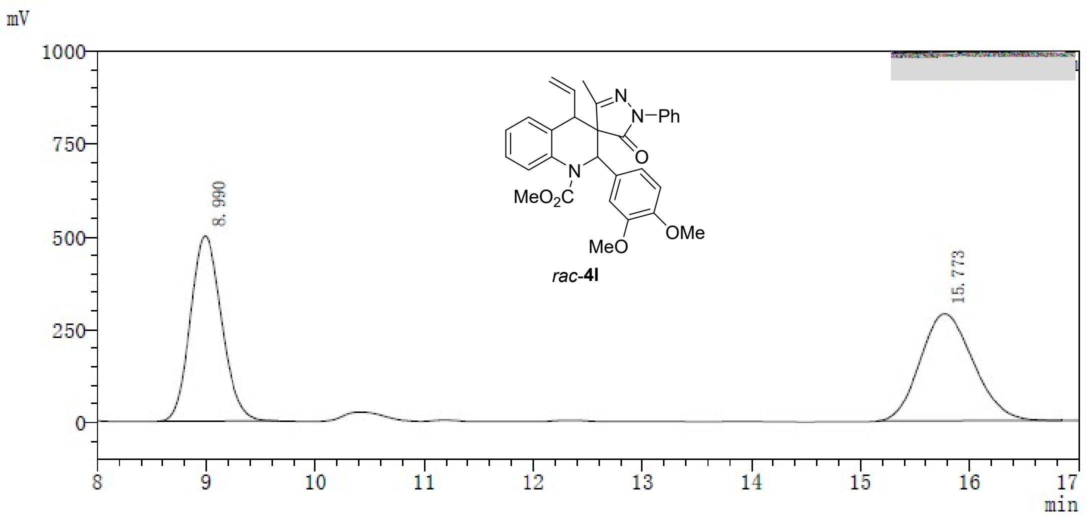

Discloser A 254 nm

Peak	Reten time (min)	Area(%)
1	8.990	50.519
2	15.773	49.481
total		100

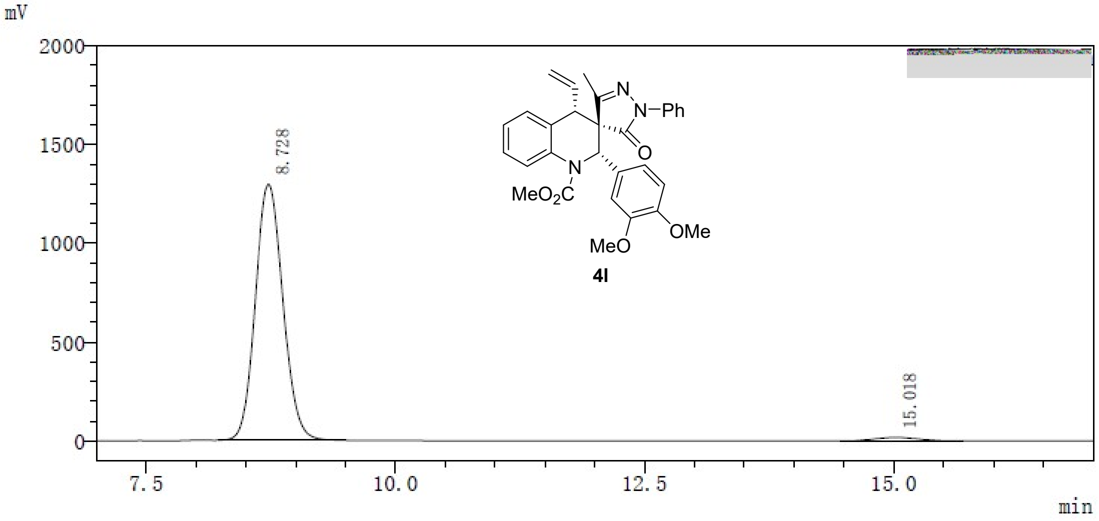

Discloser A 254 nm

Peak	Reten time (min)	Area(%)
1	8.728	97.930
2	15.018	2.070
total		100

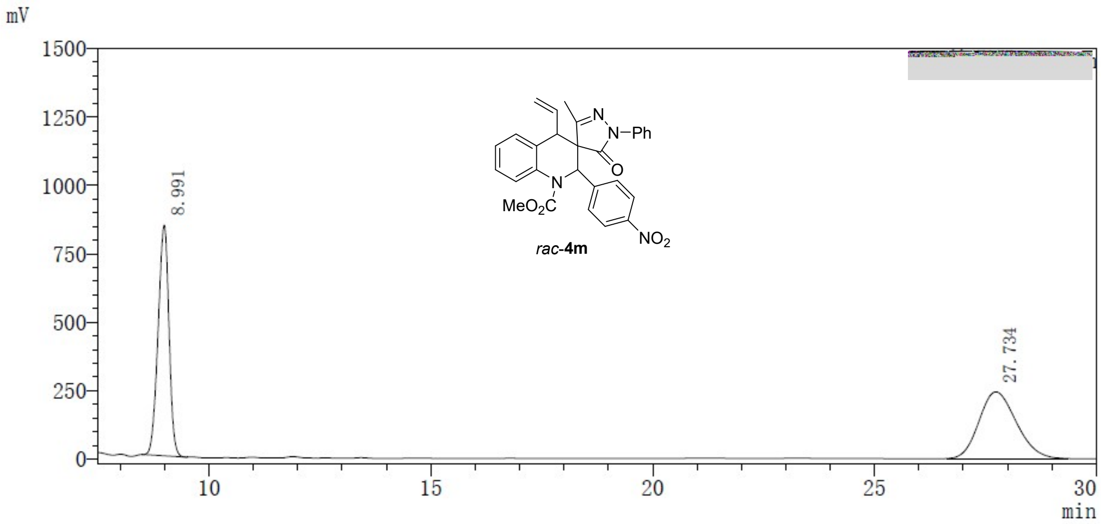

Discloser A 254 nm

Peak	Reten time (min)	Area(%)
1	8.991	50.274
2	27.734	49.726
total		100

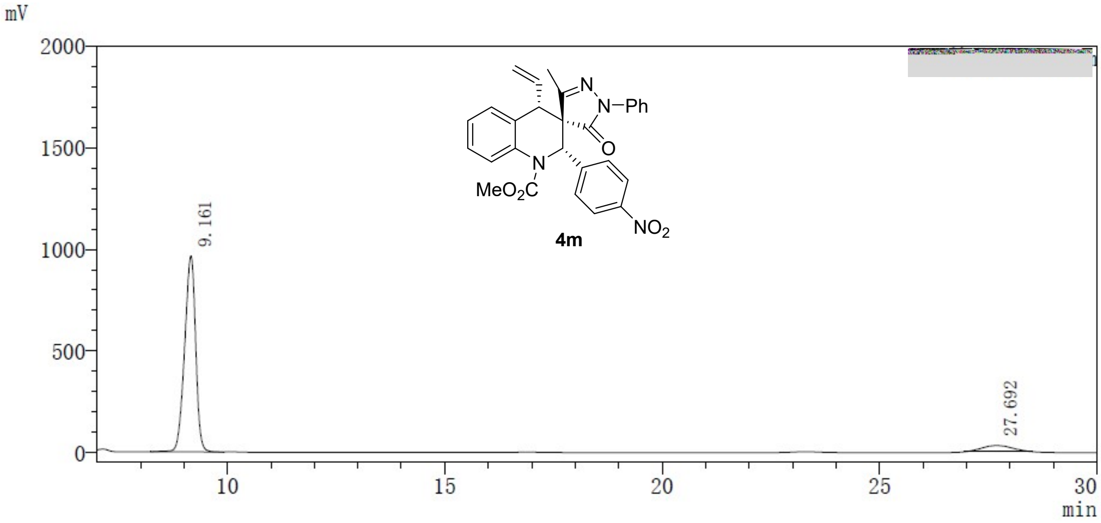

Discloser A 254 nm

Peak	Reten time (min)	Area(%)
1	9.161	91.969
2	27.692	8.031
total		100

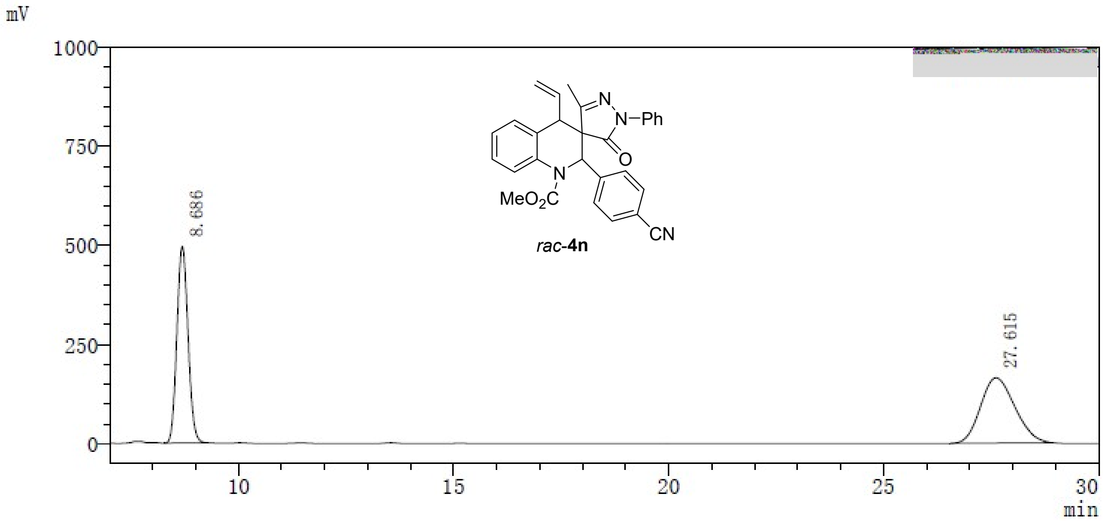

Discloser A 254 nm

Peak	Reten time (min)	Area(%)
1	8.686	49.328
2	27.615	50.672
total		100

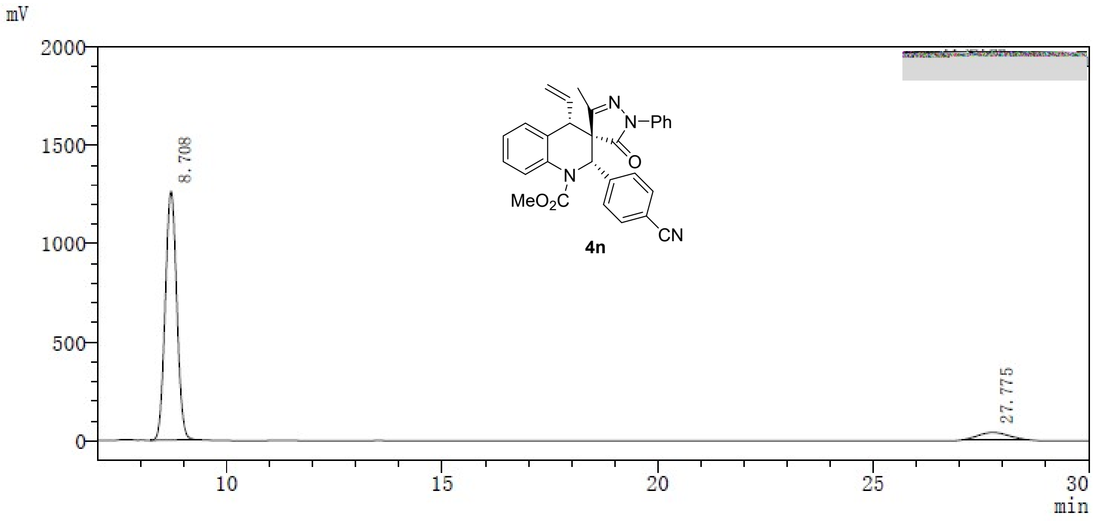

Discloser A 254 nm

Peak	Reten time (min)	Area(%)
1	8.708	92.957
2	27.775	7.043
total		100

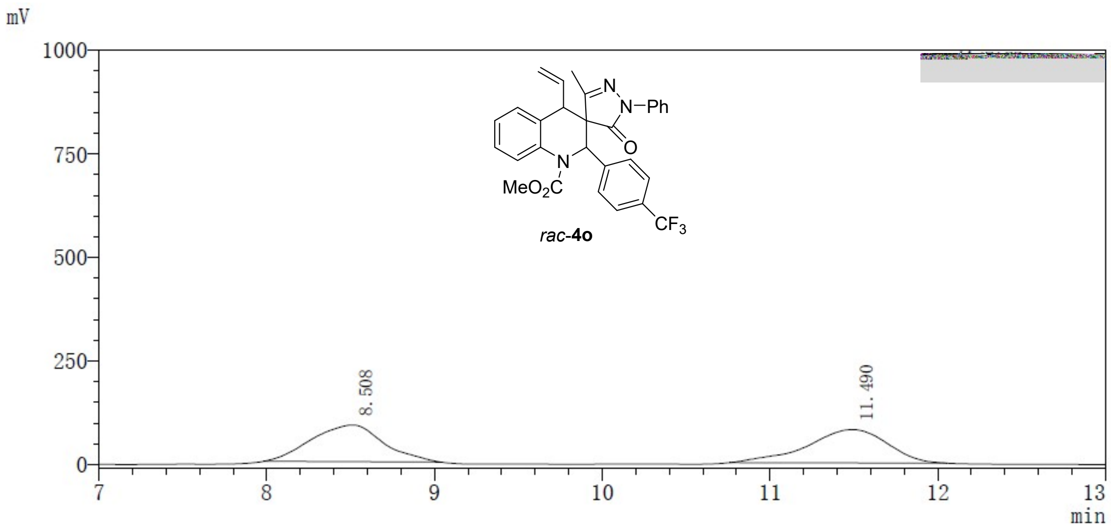

Discloser A 254 nm

Peak	Reten time (min)	Area(%)
1	8.508	49.537
2	11.490	50.463
total		100

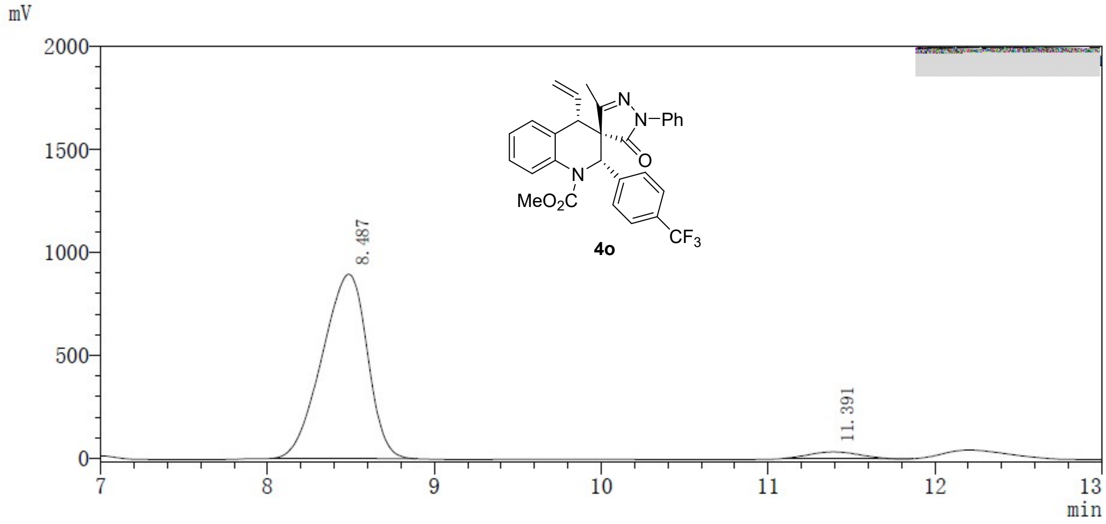

Discloser A 254 nm

Peak	Reten time (min)	Area(%)
1	8.487	96.338
2	11.391	3.662
total		100

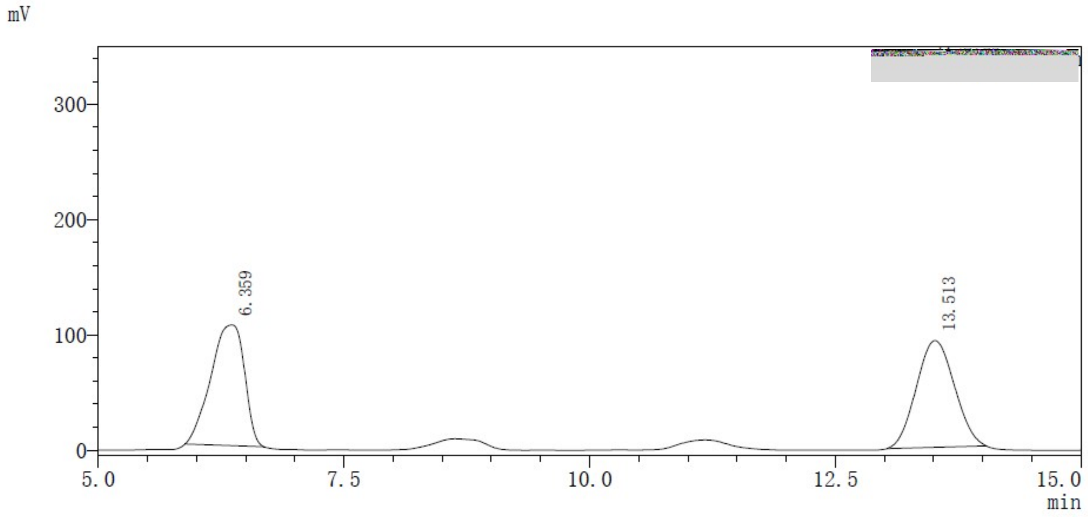

Discloser A 254 nm

Peak	Reten time (min)	Area(%)
1	6.359	49.939
2	13.513	50.061
total		100

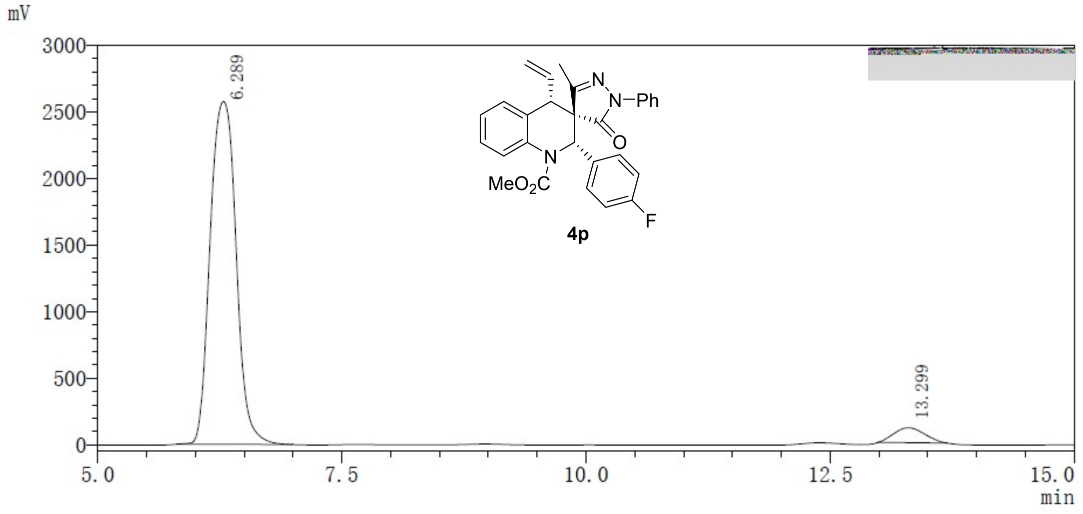

Discloser A 254 nm

Peak	Reten time (min)	Area(%)
1	6.289	95.088
2	13.299	4.912
total		100

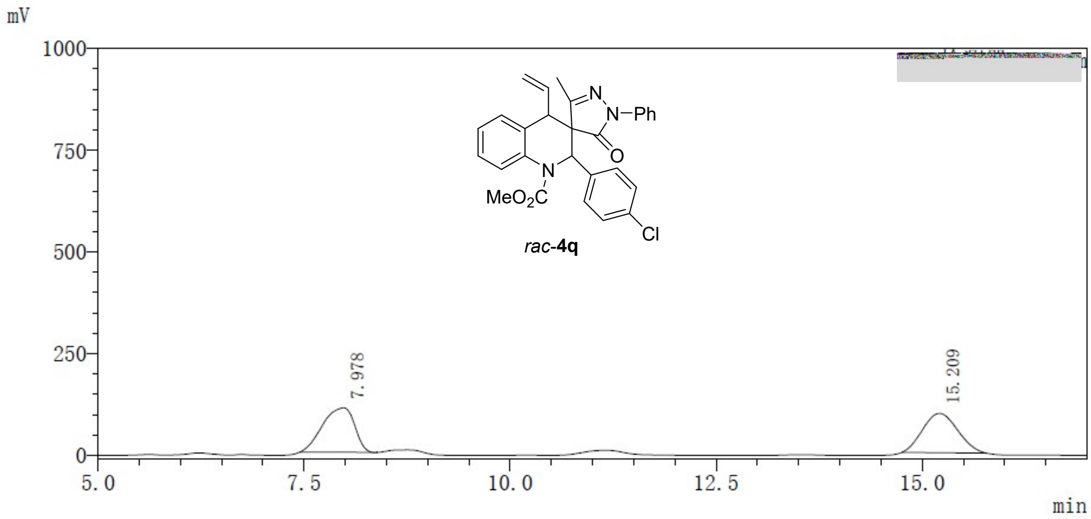

Discloser A 254 nm

Peak	Reten time (min)	Area(%)
1	7.978	50.913
2	15.209	49.087
total		100

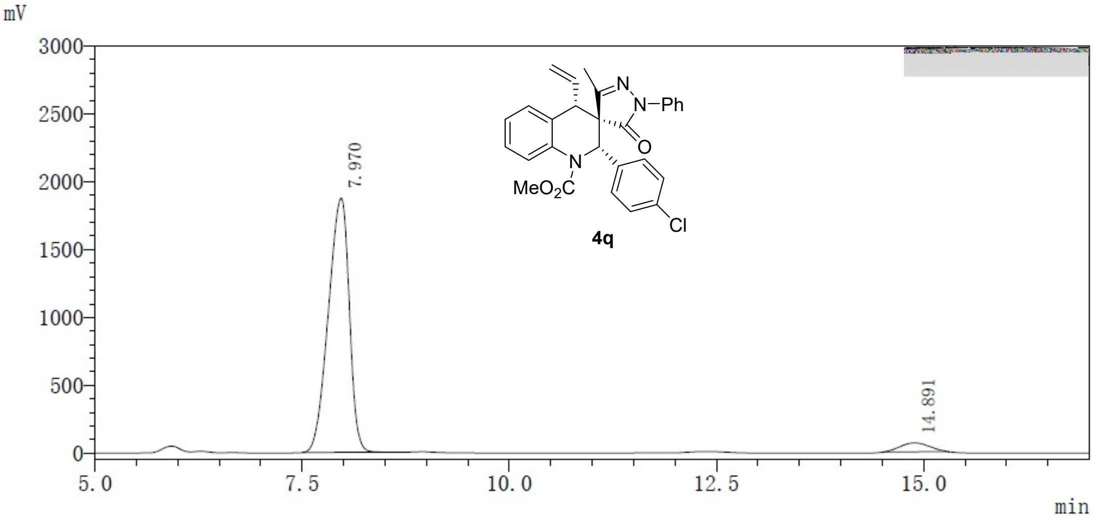

Discloser A 254 nm

Peak	Reten time (min)	Area(%)
1	7.970	95.295
2	14.891	4.705
total		100

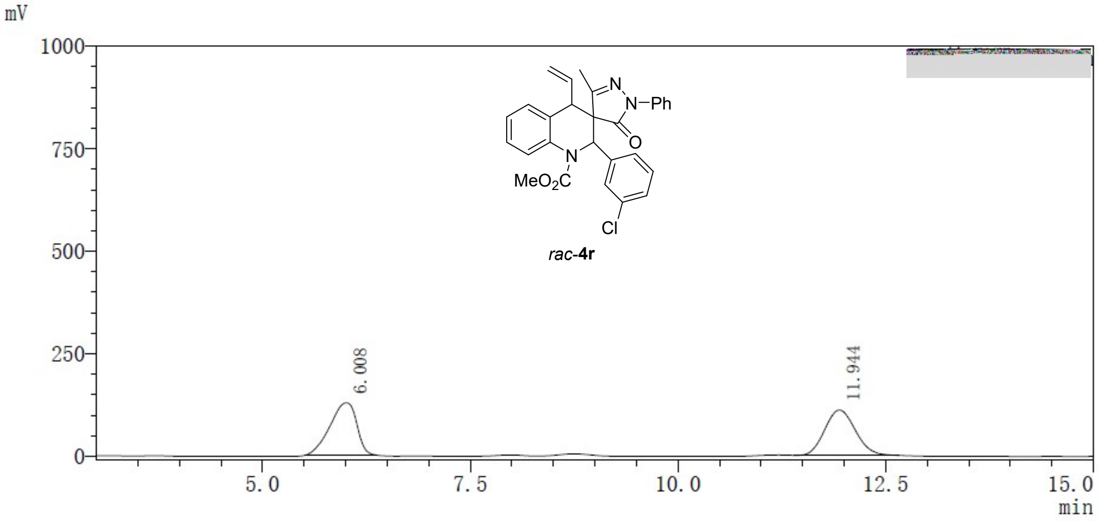

Discloser A 254 nm

Peak	Reten time (min)	Area(%)
1	6.008	49.821
2	11.944	50.179
total		100

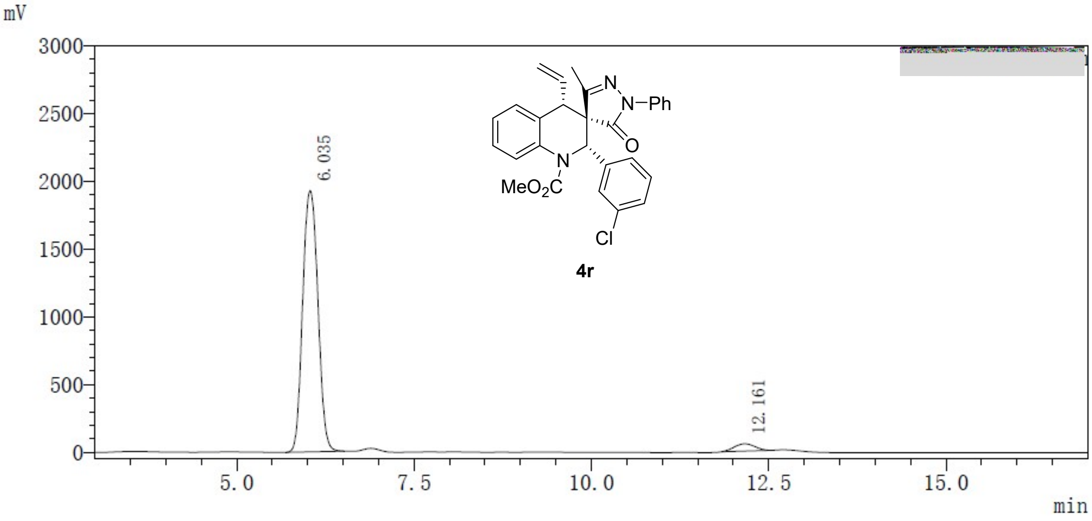

Discloser A 254 nm

Peak	Reten time (min)	Area(%)
1	6.035	96.431
2	12.161	3.569
total		100

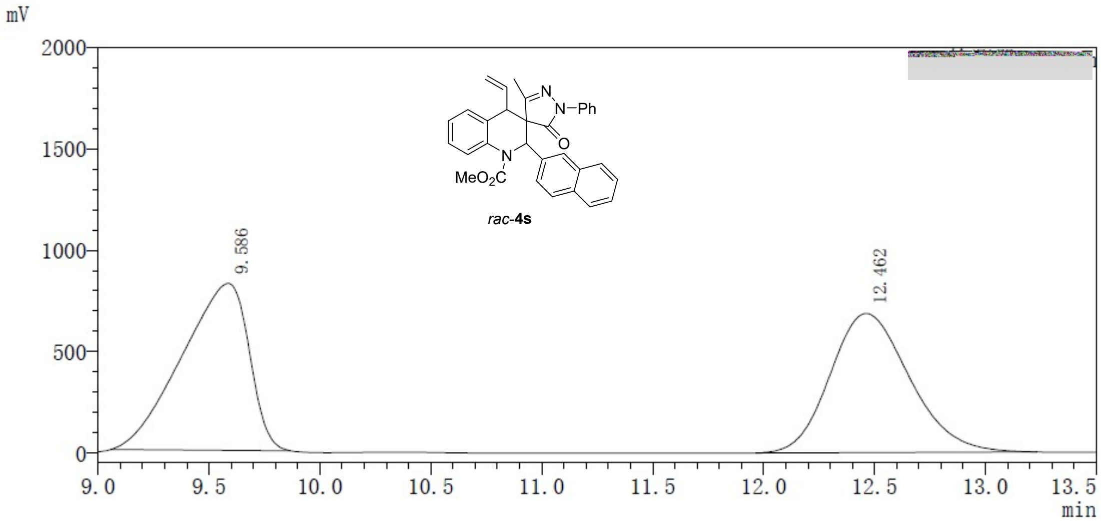

Discloser A 254 nm

Peak	Reten time (min)	Area(%)
1	9.586	50.463
2	12.462	49.537
total		100

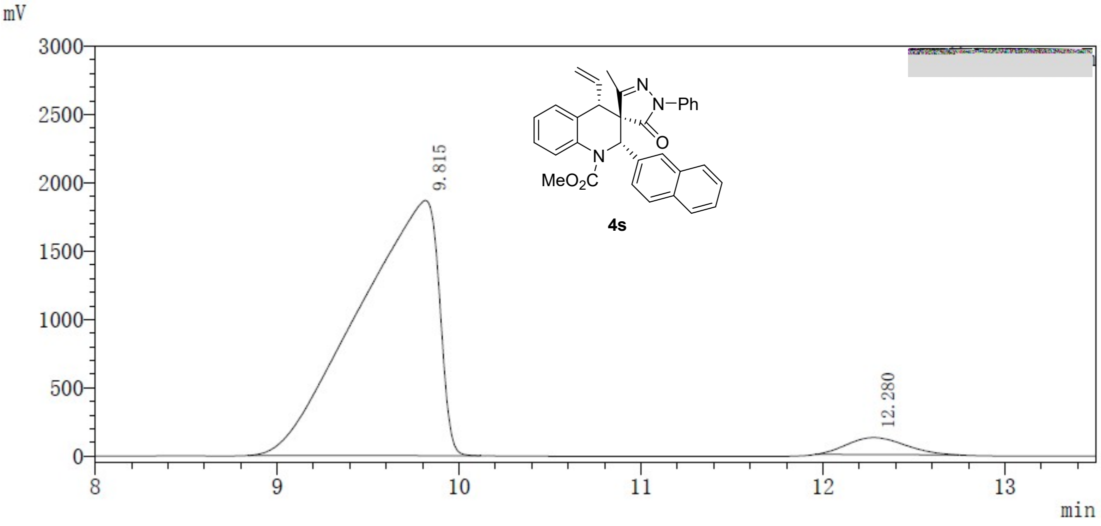

Discloser A 254 nm

Peak	Reten time (min)	Area(%)
1	9.815	95.382
2	12.280	4.618
total		100

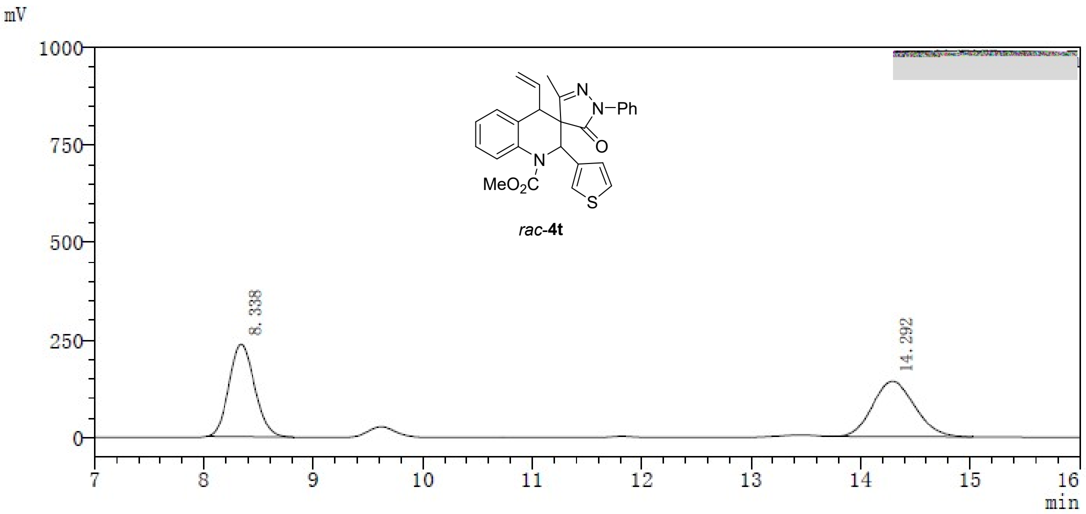

Discloser A 254 nm

Peak	Reten time (min)	Area(%)
1	8.338	50.377
2	14.292	49.623
total		100

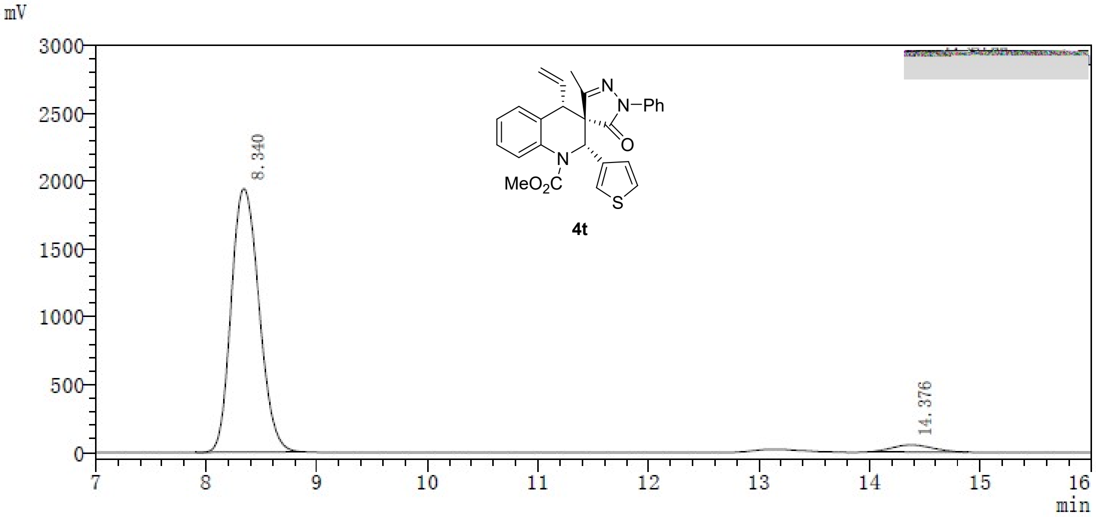

Discloser A 254 nm

Peak	Reten time (min)	Area(%)
1	8.340	96.364
2	14.376	3.636
total		100

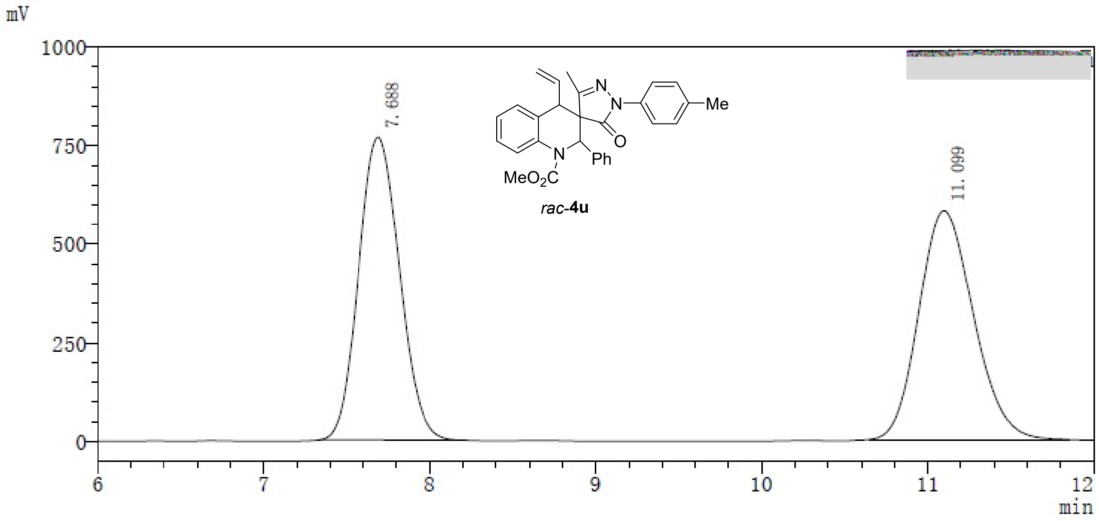

Discloser A 254 nm

Peak	Reten time (min)	Area(%)
1	7.688	49.765
2	11.099	50.235
total		100

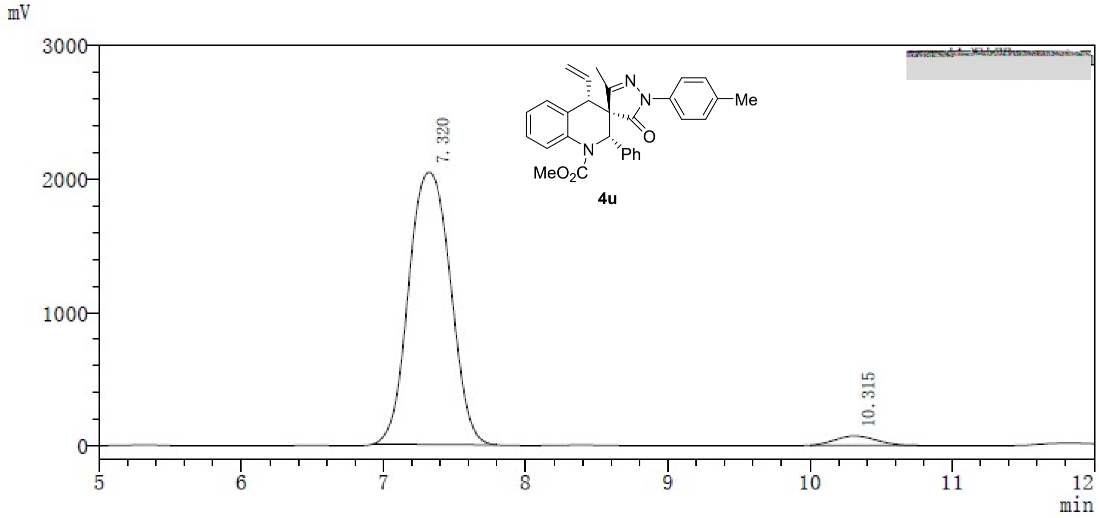

Discloser A 254 nm

Peak	Reten time (min)	Area(%)
1	7.320	96.717
2	10.315	3.283
total		100

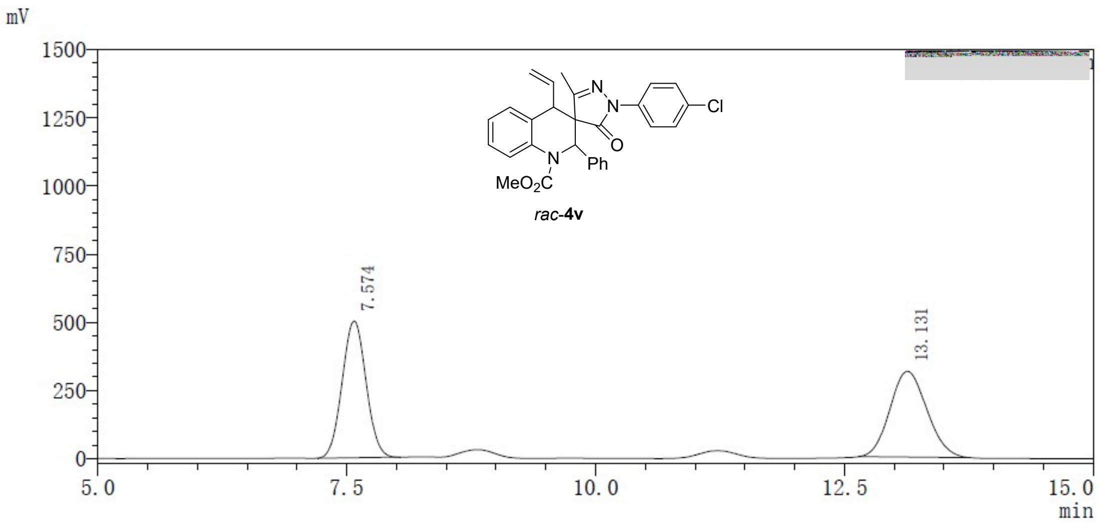

Discloser A 254 nm

Peak	Reten time (min)	Area(%)
1	7.574	50.806
2	13.131	49.184
total		100

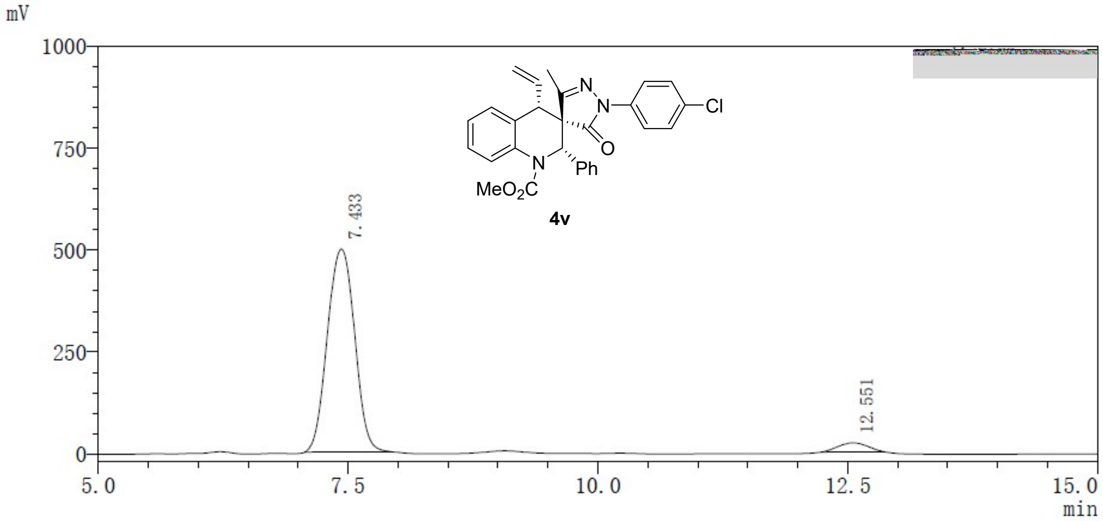

Discloser A 254 nm

Peak	Reten time (min)	Area(%)
1	7.433	95.351
2	12.551	4.649
total		100

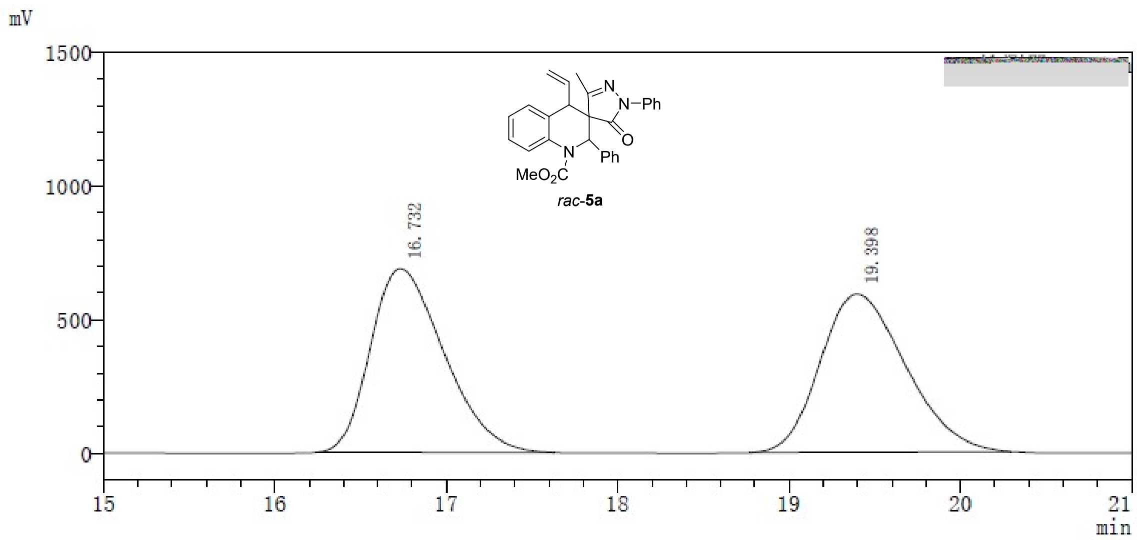

Discloser A 254 nm

Peak	Reten time (min)	Area(%)
1	16.732	50.035
2	19.398	49.965
total		100

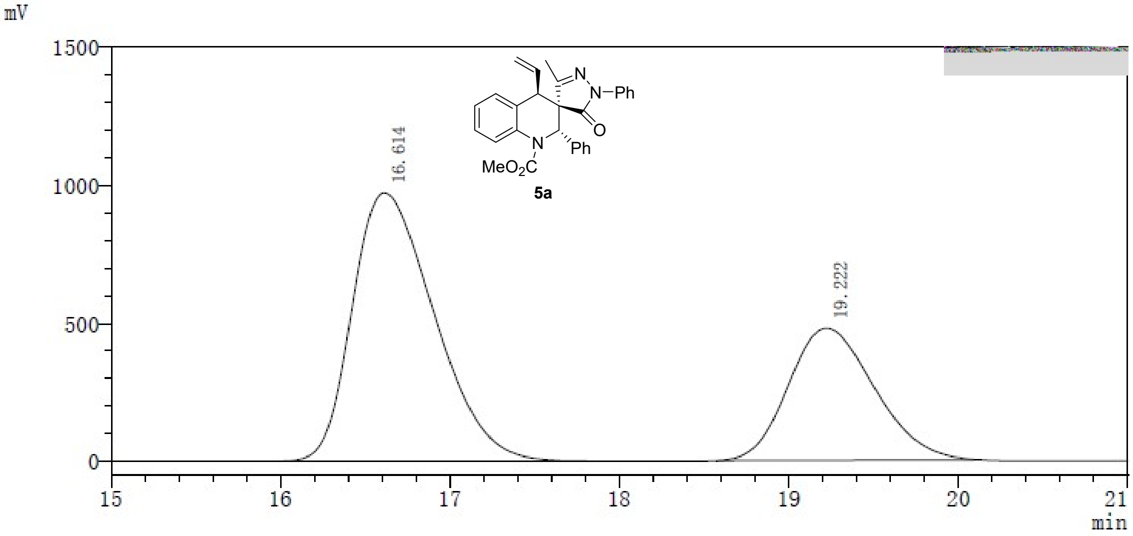

Discloser A 254 nm

Peak	Reten time (min)	Area(%)
1	16.614	65.894
2	19.222	34.106
total		100

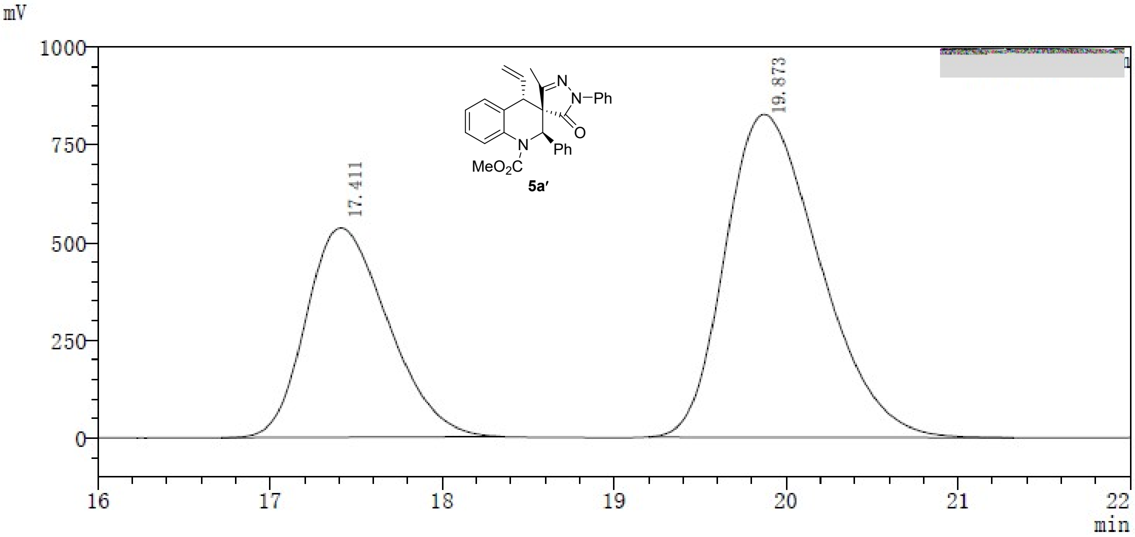

Discloser A 254 nm

Peak	Reten time (min)	Area(%)
1	17.411	36.014
2	19.873	63.986
total		100

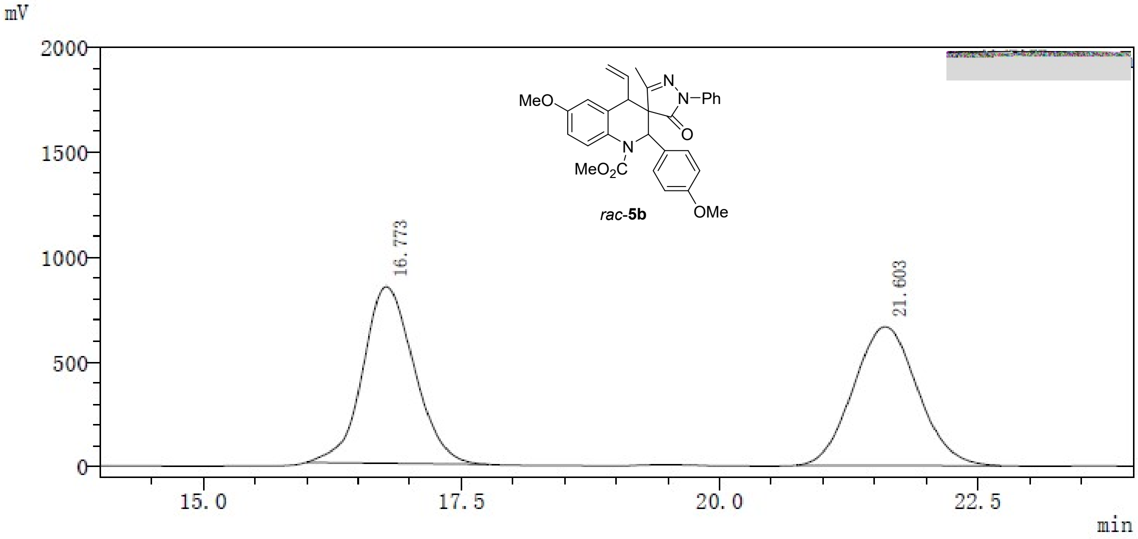

Discloser A 254 nm

Peak	Reten time (min)	Area(%)
1	16.773	50.392
2	21.603	49.608
total		100

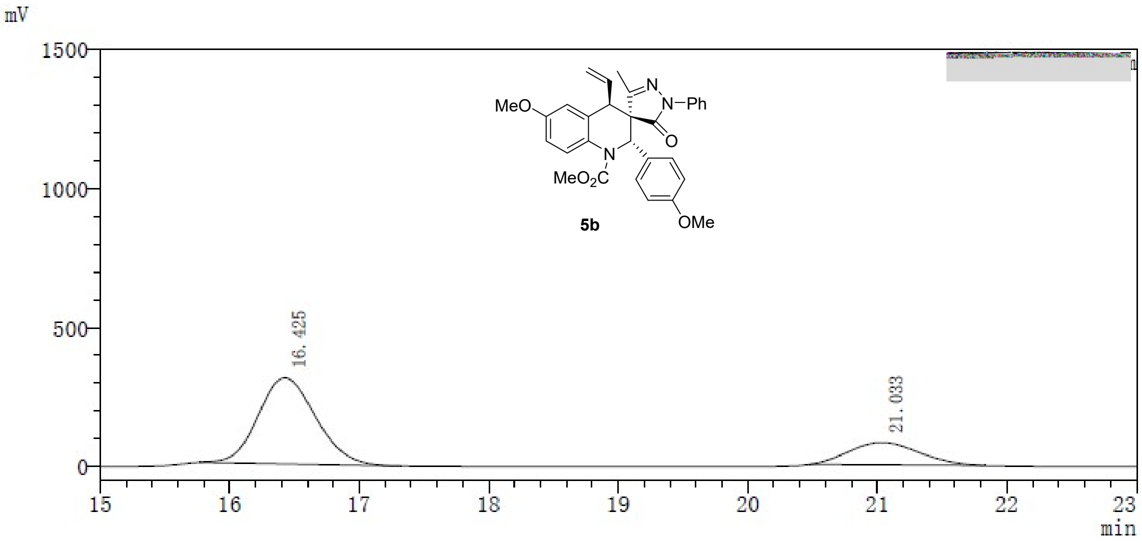

Discloser A 254 nm

Peak	Reten time (min)	Area(%)
1	16.425	75.932
2	21.033	24.068
total		100

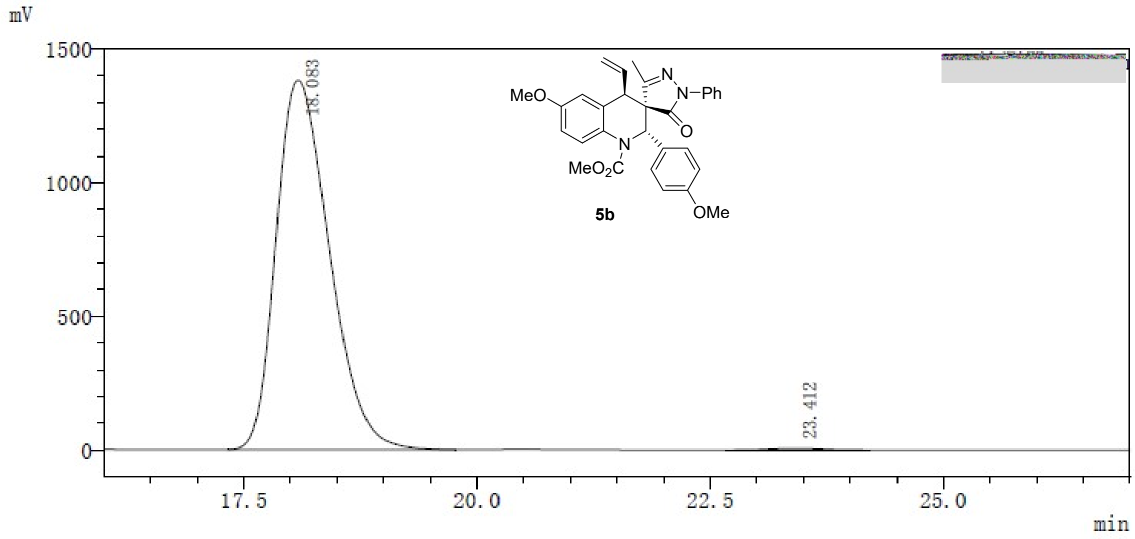

Discloser A 254 nm

Peak	Reten time (min)	Area(%)
1	18.083	99.556
2	23.412	0.444
total		100

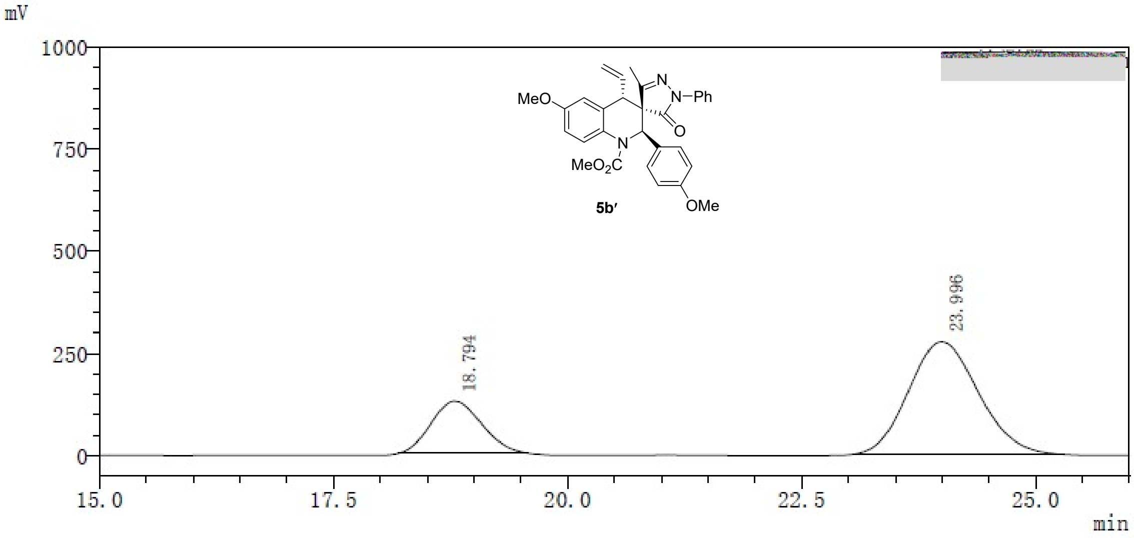

Discloser A 254 nm

Peak	Reten time (min)	Area(%)
1	18.794	25.048
2	23.996	74.952
total		100

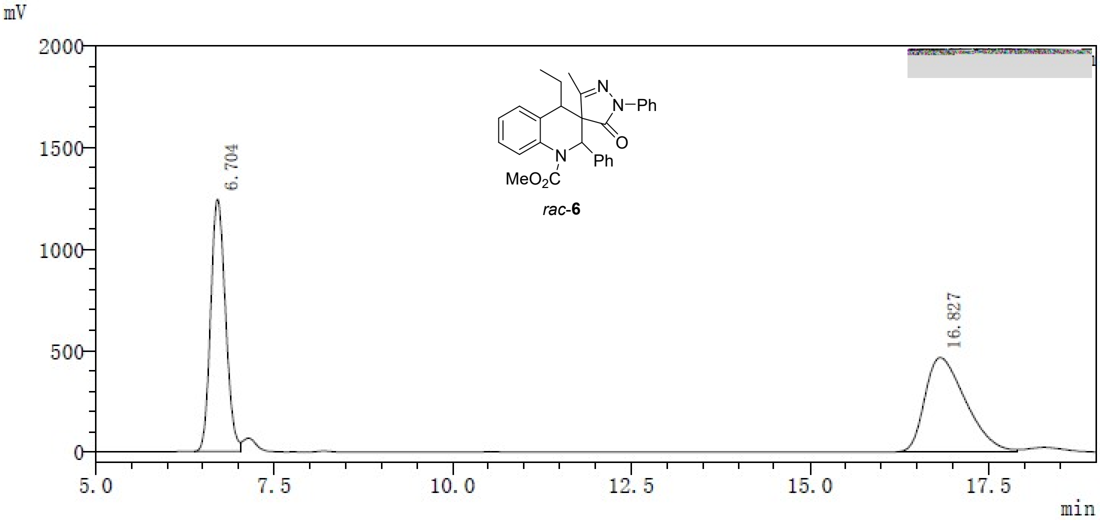

Discloser A 254 nm

Peak	Reten time (min)	Area(%)
1	6.704	49.292
2	16.827	50.708
total		100

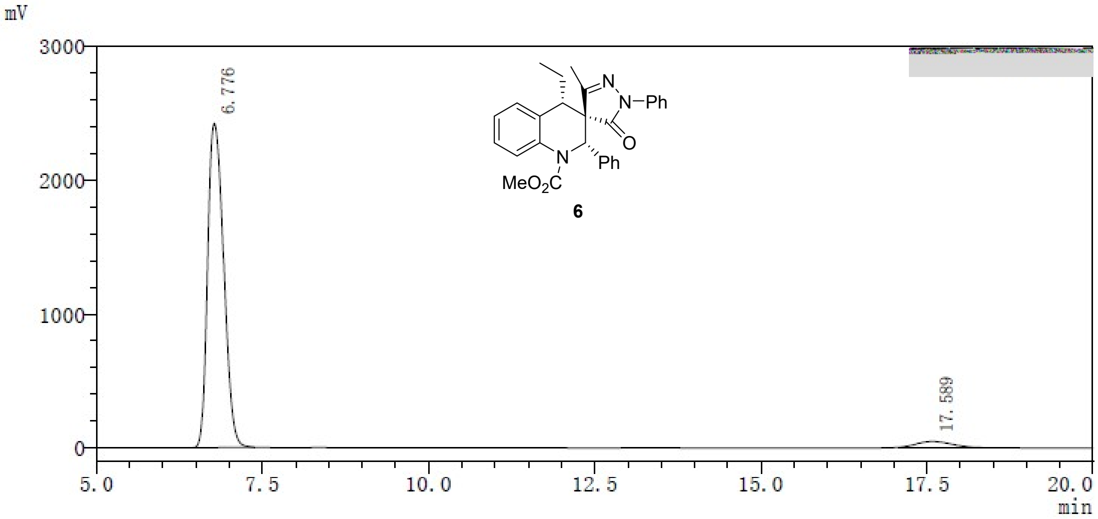

Discloser A 254 nm

Peak	Reten time (min)	Area(%)
1	6.776	96.191
2	17.589	3.809
total		100

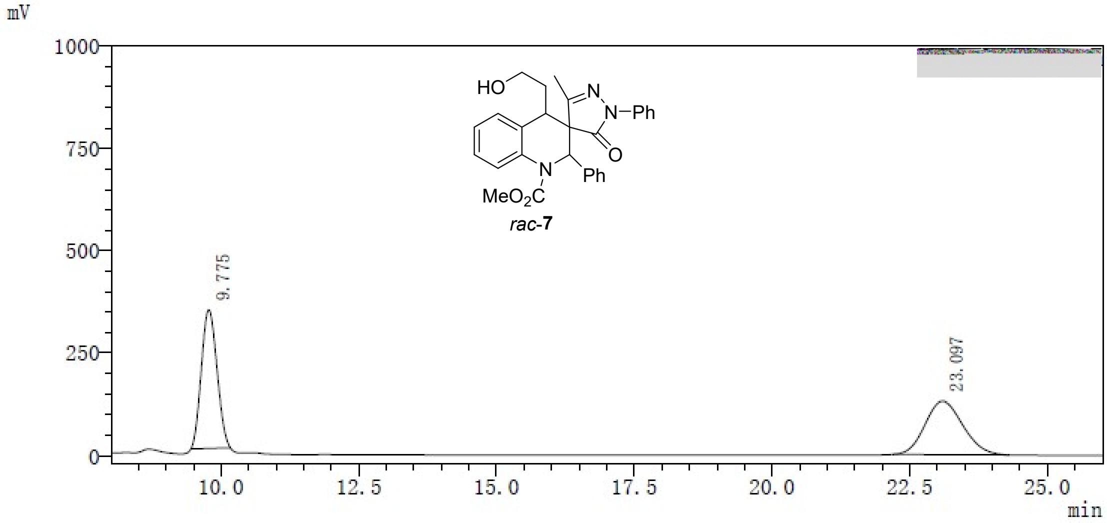

Discloser A 254 nm

Peak	Reten time (min)	Area(%)
1	9.775	51.750
2	23.097	48.250
total		100

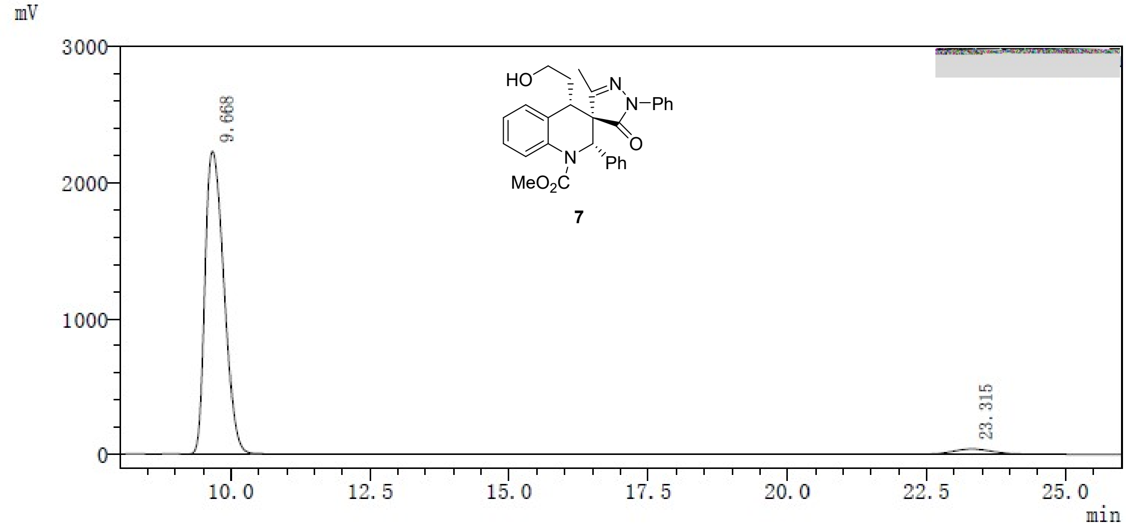

Discloser A 254 nm

Peak	Reten time (min)	Area(%)
1	9.668	96.682
2	23.315	3.318
total		100

7. X-ray crystal structure

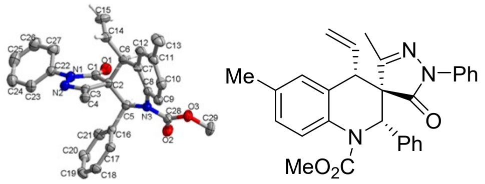

Crystal structure of **3c (CCDC: 2132601)**

Table S6 Crystal data and structure refinement for **3c**

Identification code	2132601
Empirical formula	$C_{29}H_{27}N_3O_3$
Formula weight	465.53
Temperature/K	293(2)
Crystal system	orthorhombic
Space group	$P2_12_12_1$
a/Å	9.75496(19)
b/Å	13.6686(3)
c/Å	18.7757(4)
α/°	90

β/°	90
γ/°	90
Volume/Å^3	2503.48(8)
Z	4
ρ_{calc}g/cm^3	1.235
μ/mm^{-1}	0.648
F(000)	984.0
Crystal size/mm^3	0.16 × 0.1 × 0.09
Radiation	CuKα (λ = 1.54184)
2Θ range for data collection/°	8 to 141.926
Index ranges	-11 ≤ h ≤ 5, -12 ≤ k ≤ 16, -22 ≤ l ≤ 21
Reflections collected	9409
Independent reflections	4720 [R_{int} = 0.0319, R_{sigma} = 0.0437]
Data/restraints/parameters	4720/0/320
Goodness-of-fit on F^2	1.025
Final R indexes [I>=2σ (I)]	R_1 = 0.0474, wR_2 = 0.1211
Final R indexes [all data]	R_1 = 0.0622, wR_2 = 0.1354
Largest diff. peak/hole / e Å^{-3}	0.13/-0.13

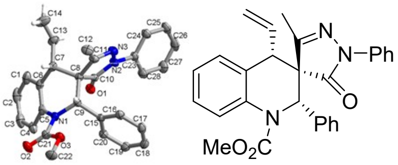

Crystal structure of **4a (CCDC: 2132611)**

Table S7 Crystal data and structure refinement for **4a**

Identification code	2132611
Empirical formula	$C_{28}H_{25}N_3O_3$
Formula weight	451.51
Temperature/K	293(2)
Crystal system	monoclinic
Space group	P2$_1$/c
a/Å	12.6477(6)
b/Å	12.7165(3)
c/Å	19.2801(10)
α/°	90
β/°	130.968(8)
γ/°	90
Volume/Å^3	2341.4(3)
Z	4
ρ_{calc}g/cm^3	1.281

μ/mm^{-1}	0.677
F(000)	952.0
Crystal size/mm^3	0.15 × 0.1 × 0.08
Radiation	CuKα (λ = 1.54184)
2Θ range for data collection/°	9.234 to 134.148
Index ranges	$-15 \leq h \leq 15, -15 \leq k \leq 11, -23 \leq l \leq 20$
Reflections collected	11047
Independent reflections	4178 [R_{int} = 0.0320, R_{sigma} = 0.0351]
Data/restraints/parameters	4178/0/310
Goodness-of-fit on F^2	1.030
Final R indexes [I>=2σ (I)]	R_1 = 0.0460, wR_2 = 0.1212
Final R indexes [all data]	R_1 = 0.0584, wR_2 = 0.1326
Largest diff. peak/hole / e $Å^{-3}$	0.25/-0.19

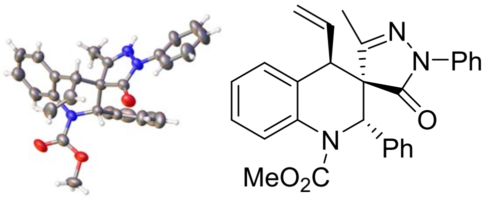

Relative configuration of **5b** and **5b' (CCDC: 2132592)**

Table S8 Crystal data and structure refinement for **5b(5b')**

Identification code	2132592
Empirical formula	$C_{28}H_{24}N_3O_3$
Formula weight	450.50
Temperature/K	298.0
Crystal system	monoclinic
Space group	$P2_1$
a/Å	8.647(3)
b/Å	20.509(7)
c/Å	8.8540(18)
α/°	90
β/°	101.501(13)
γ/°	90
Volume/$Å^3$	1538.7(8)
Z	2
$\rho_{calc}g/cm^3$	0.972
μ/mm^{-1}	0.064
F(000)	474.0
Crystal size/mm^3	0.1 × 0.1 × 0.1
Radiation	MoKα (λ = 0.71073)

2Θ range for data collection/°	3.972 to 55.04
Index ranges	$-11 \le h \le 11$, $-26 \le k \le 26$, $-10 \le l \le 11$
Reflections collected	25970
Independent reflections	7033 [R_{int} = 0.0321, R_{sigma} = 0.0291]
Data/restraints/parameters	7033/1/310
Goodness-of-fit on F^2	1.176
Final R indexes [I>=2σ (I)]	R_1 = 0.0831, wR_2 = 0.2058
Final R indexes [all data]	R_1 = 0.1035, wR_2 = 0.2212
Largest diff. peak/hole / e Å^{-3}	1.17/-0.79

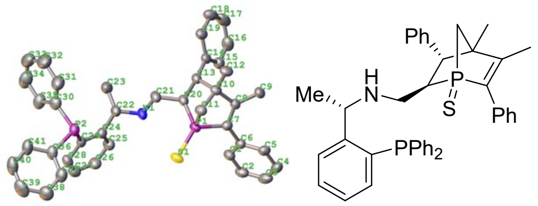

Crystal structure of **Y1' (CCDC: 2150889)**

Table S9 Crystal data and structure refinement for **Y1'**

Identification code	2150889
Empirical formula	$C_{41}H_{41}NP_2S$
Formula weight	235.23
Temperature/K	200.00(10)
Crystal system	monoclinic
Space group	$P2_1$
a/Å	8.80910(10)
b/Å	14.29220(10)
c/Å	15.6275(2)
α/°	90
β/°	97.2740(10)
γ/°	90
Volume/Å^3	1951.69(4)
Z	8
ρ_{calc}g/cm^3	1.601
μ/mm^{-1}	4.231
F(000)	976.0
Crystal size/mm^3	0.3 × 0.1 × 0.1
Radiation	Cu Kα (λ = 1.54184)
2Θ range for data collection/°	5.702 to 147.44
Index ranges	$-10 \le h \le 10$, $-15 \le k \le 17$, $-18 \le l \le 19$
Reflections collected	20108
Independent reflections	6966 [R_{int} = 0.0321, R_{sigma} = 0.0346]

Data/restraints/parameters	6966/1/409
Goodness-of-fit on F^2	1.055
Final R indexes [I>=2σ (I)]	R_1 = 0.0411, wR_2 = 0.1134
Final R indexes [all data]	R_1 = 0.0436, wR_2 = 0.1146
Largest diff. peak/hole / e $Å^{-3}$	0.37/-0.29
Flack parameter	0.036(9)
